# Phosphorylation of an HP1-like Protein Regulates Heterochromatin Body Assembly for DNA Elimination

**DOI:** 10.1016/j.devcel.2015.11.017

**Published:** 2015-12-21

**Authors:** Kensuke Kataoka, Kazufumi Mochizuki

**Affiliations:** 1 Institute of Molecular Biotechnology of the Austrian Academy of Sciences (IMBA), Dr. Bohr-Gasse 3, 1030 Vienna, Austria

## Abstract

Heterochromatic loci are often assembled into higher-order heterochromatin bodies in diverse eukaryotes. However, the formation and biological roles of heterochromatin bodies are poorly understood. In the ciliated protozoan *Tetrahymena*, de novo heterochromatin body formation is accompanied by programmed DNA elimination. Here, we show that the heterochromatin body component Jub1p promotes heterochromatin body formation and dephosphorylation of the Heterochromatin Protein 1-like protein Pdd1p. Through the mutagenesis of the phosphorylated residues of Pdd1p, we demonstrate that Pdd1p dephosphorylation promotes the electrostatic interaction between Pdd1p and RNA in vitro and heterochromatin body formation in vivo. We therefore propose that heterochromatin body is assembled by the Pdd1p-RNA interaction. Pdd1p dephosphorylation and Jub1p are required for heterochromatin body formation and DNA elimination but not for local heterochromatin assembly, indicating that heterochromatin body plays an essential role in DNA elimination.

## Highlights

• Jub1p facilitates dephosphorylation of the HP1-like protein Pdd1p
• Pdd1p dephosphorylation promotes its RNA binding and heterochromatin body formation
• Jub1p and Pdd1p dephosphorylation is required for DNA elimination
• Heterochromatin body has function beyond local heterochromatin maintenance

## Graphical Abstract

**Figure:**
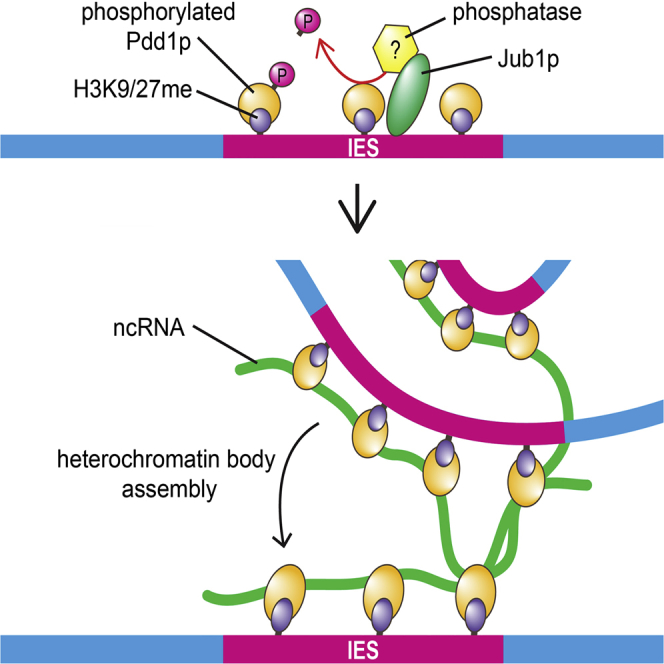

## Teaser

Heterochromatic loci are often assembled into higher-order heterochromatin bodies. Kataoka and Mochizuki provide evidence that developmentally programmed dephosphorylation of an HP1-like protein in *Tetrahymena* promotes its interaction with RNA, heterochromatin body assembly, and DNA elimination without altering local heterochromatic states, suggesting that heterochromatin body has an essential biological function.

## Introduction

Heterochromatin is a closed and mostly transcriptionally repressed state of chromatin, which is dictated by a set of posttranslational histone modifications (Jenuwein and Allis, 2001, Kouzarides, 2007). Hypoacetylation of histone tails maintains closed configuration of heterochromatin by exposing positively charged lysine (Lys) and stabilizing histone-DNA interactions (Mutskov et al., 1998). Methylated histone H3 at Lys 9 (H3K9me) recruits Heterochromatin Protein 1 (HP1), which self-oligomerizes to facilitate compaction of nucleosome arrays (Canzio et al., 2011, Cowieson et al., 2000). Similarly, methylated histone H3 at Lys 27 (H3K27me) attracts Polycomb Repressive Complex 1 (PRC1), and PRC1-DNA and PRC1-PRC1 interactions compact chromatin (Eskeland et al., 2010, Grau et al., 2011).

In certain cell types, multiple heterochromatic loci are assembled into aggregated higher order structures called heterochromatin bodies (Politz et al., 2013). In mammals to plants, constitutive heterochromatin loci at centromeric and other repetitive sequences are organized into heterochromatin bodies called chromocenters (Fransz and de Jong, 2002, Probst and Almouzni, 2011). Chromocenters are condensed their underlying sequences that are tightly silenced in differentiated mammalian cells, whereas they are more dispersed and transcribed in embryonic stem cells and in some cancer cells (Carone and Lawrence, 2013, Efroni et al., 2008, Zhu et al., 2011). In female mammalian somatic cells, a whole X chromosome forms a heterochromatin body, called the Barr body, which is suggested to be important for X inactivation (Deng et al., 2014). Moreover, in human senescent cells, heterochromatic loci are reorganized into senescence-associated heterochromatin foci (SAHF), which are proposed to be a part of the tumor suppressor pathway (Narita, 2007). Because SAHF are formed without detectable alterations of underlying histone modifications (Chandra et al., 2012), SAHF formation per se is likely involved in gene regulation. These examples indicate that, in addition to the compaction of individual heterochromatic loci, their assembly into heterochromatin bodies may play important roles in regulating chromatin activities. However, because there is no intervention system in which heterochromatin body formation is disturbed without altering local heterochromatin, whether and to what extent heterochromatin body contributes to the regulation of the underlying sequences remain unknown.

**Figure 1:**
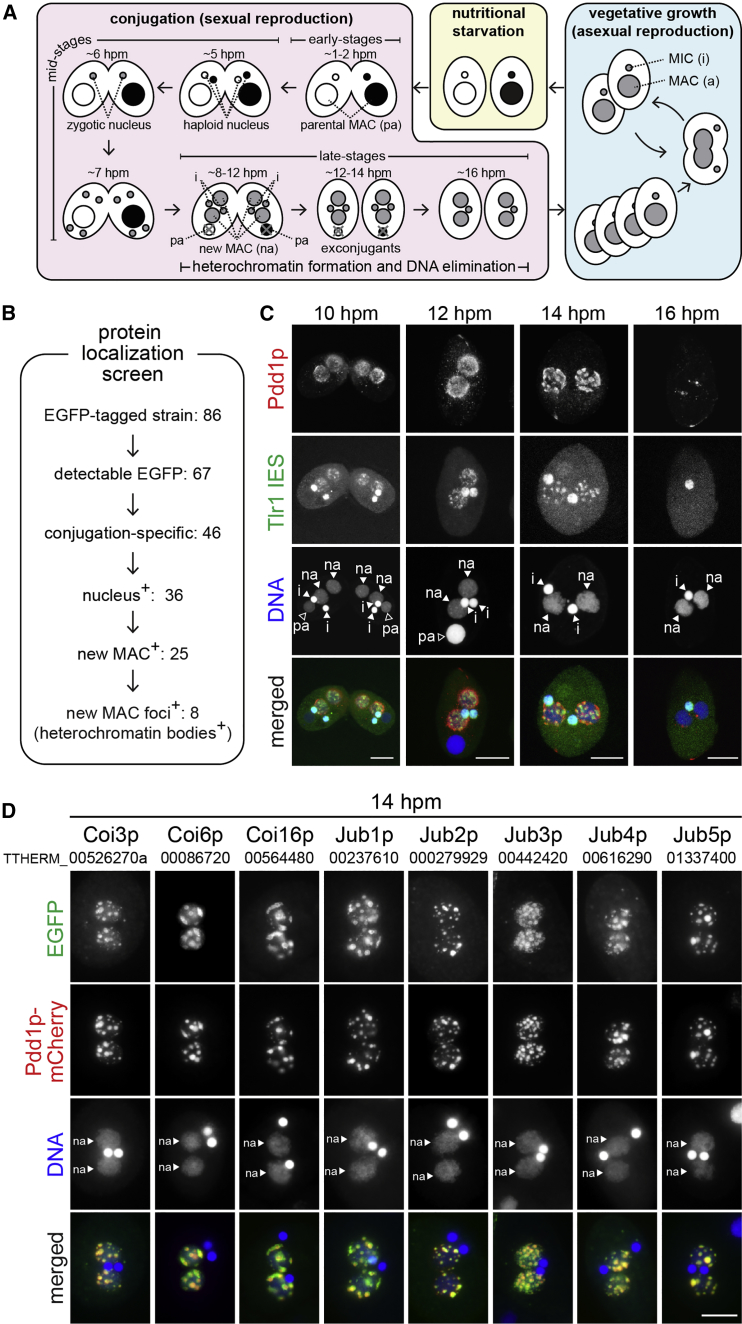
Identification of Heterochromatin Body Components (A) A single *Tetrahymena thermophila* cell possesses a MAC (a) and a MIC (i). During vegetative growth, these nuclei divide and are segregated independently into daughter cells. Nutritional starvation induces the conjugation of two cells carrying different mating types. In the early conjugation stage (∼1–4 hpm), the MICs undergo meiosis. In the mid-stage, one of the meiotic products is exchanged between the cells (∼5 hpm) and fuses with the stationary meiotic product to form a zygotic nucleus (∼6 hpm), which then divides twice to form two new MACs and two MICs (∼7 hpm). At the late-stage, the new MACs (na) are enlarged (∼8 hpm). The pair is dissolved and the parental MAC (pa) and one of the MICs are degraded in the exconjugants (∼12–16 hpm). The exconjugants resume vegetative growth when nutrients are available. (B) Summary of the screen for heterochromatin body components. (C) WT cells at 10, 12, 14, and 16 hpm were hybridized with a probe complementary to the Tlr1 element (green) and stained with an anti-Pdd1p antibody (red). DNA was stained with DAPI (blue). Arrowheads indicate the MIC (i), the new MAC (na), and the parental MAC (pa). The scale bars represent 10 μm. (D) Exconjugants expressing the indicated proteins tagged with EGFP (green) and Pdd1p-mCherry (red) were counterstained with DAPI (blue). Arrowheads indicate the new MACs (na). All pictures share the scale bar, representing 10 μm. See also Figure S1.

Heterochromatin and heterochromatin bodies are formed during the process of programmed DNA elimination in *Tetrahymena thermophila* (Chalker, 2008). Like most ciliated protozoans, *Tetrahymena* harbors two types of nuclei in a single cell: the transcriptionally inactive germline micronucleus (MIC) and the transcriptionally active somatic macronucleus (MAC). Nutritional starvation induces sexual reproduction, called conjugation (Figure 1A), in which the MIC undergoes meiosis and its zygotic products produce both new MIC and new MAC for progeny, whereas the parental MAC is degraded. In the new MAC, more than 8,000 internal eliminated sequences (IESs), which consist of one-third (∼50 Mb) of the MIC genome, many of which are related to transposons, are removed by programmed DNA elimination (Chalker and Yao, 2011, Coyne et al., 2012, Kataoka and Mochizuki, 2011). An RNAi-related mechanism recruits the H3K9 and H3K27 dual-specific methyltransferase Ezl1p to IESs, resulting in the accumulation of H3K9/K27me and their binding HP1-like protein Pdd1p (Aronica et al., 2008, Liu et al., 2007, Taverna et al., 2002). During or prior to DNA elimination, thousands of heterochromatinized IES loci are organized into several electron-dense heterochromatin bodies (Madireddi et al., 1996) (see also Figure 1C). The heterochromatinized IESs are eventually excised by the domesticated transposase Tpb2p (Cheng et al., 2010, Vogt and Mochizuki, 2013).

Because DNA elimination in *Tetrahymena* occurs during the course of inducible conjugation, it serves as an ideal model to analyze the process of heterochromatin body formation. Here, we show identification and functional analyses of the heterochromatin body component Jub1p, which suggest that the heterochromatin body is assembled by phosphorylation-mediated electrostatic regulation of RNA-Pdd1p interaction and is essential for DNA elimination.

## Results

### Identification of Proteins that Localize to Nuclear Foci in the New MAC

To identify proteins involved in the assembly of heterochromatin bodies in *Tetrahymena*, we performed a protein localization screen to identify heterochromatin body components. Because most of the reported heterochromatin body components are exclusively expressed during conjugation (Chalker, 2008), we analyzed 86 genes with expressed sequence tags only in conjugating cells. Each gene was engineered to express a fusion protein with EGFP from its endogenous MAC locus (Figure S1A, top), and its localization was observed in exponentially growing, starved, and conjugating cells.

The screen is summarized in Figure 1B, and detailed data can be found in Data S1. For 67 genes, EGFP-tagged proteins were detected at least in one of the stages analyzed, and 46 of them showed conjugation-specific expression. Among them, 36 were detected in at least one nucleus, and 25 of these localized to the new MAC. Importantly, 8 of the new MAC-localizing proteins were detected in nuclear foci at the late (14 hr post-mixing [hpm]) stage.

### Nuclear Foci Proteins Identified Are Heterochromatin Body Components

To localize heterochromatin body, we first validated the HP1-like protein Pdd1p as a marker. Pdd1p, which was immunofluorescently stained by an anti-Pdd1p antibody, and the moderately repeated IES element Tlr1 (Wuitschick et al., 2002), which was detected by DNA fluorescence in situ hybridization (DNA-FISH), were first distributed homogeneously in the new MAC at the onset of new MAC differentiation (Figure 1C, 10 hpm). They then gradually accumulated into several foci at later stages (Figure 1C, 12–14 hpm) and eventually disappeared (Figure 1C, 16 hpm). Because Pdd1p and the Tlr1 IESs co-localized in the same foci (Figure 1C, 14 hpm), Pdd1p can be used as a marker for IES-containing heterochromatin bodies. Counterintuitively, the heterochromatin bodies were poorly stained with DAPI (Figure 1C, 14–16 hpm). However, because the heterochromatin bodies were stained intensely with an anti-DNA antibody (Figure S1B), we believe that DNA is concentrated in the heterochromatin bodies but DNA there has a low affinity for DAPI for an unknown reason.

We then compared the localization of mCherry-tagged Pdd1p to those of the eight foci-forming proteins tagged with EGFP (Figure S1A). All of these foci-forming proteins co-localized with Pdd1p-mCherry at 14 hpm (Figure 1D), indicating that they are heterochromatin body components. Three of them, Coi3p, Coi6p, and Coi16p, were partially characterized previously (Woehrer et al., 2015). The other five proteins were named Junk Buster 1–5 (Jub1p–5p) (Figure 1D). Jub5p was reported during the course of this study and also called Tcd1p (Xu et al., 2015). Coi6p and Jub5p are HP1-like proteins. Jub3p is a WD40 repeat protein similar to a component of PRC2 in metazoans. The other proteins show no detectable similarities with any known proteins outside of the genus *Tetrahymena*. In this study, we report the further characterization of Jub1p.

### Jub1p Is a Heterochromatin Body Component

**Figure 2:**
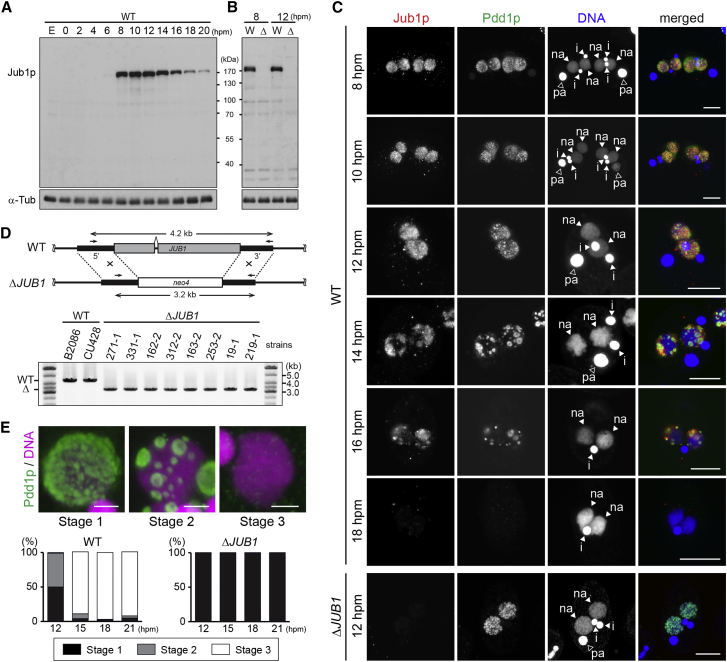
Jub1p Is Required for Heterochromatin Body Formation (A and B) Exponentially growing (E), starved (0 hpm), or conjugating (2–20 hpm) WT cells (A) and WT (W) and *JUB1* KO (Δ) cells at late-conjugation stages (8–12 hpm) (B) were analyzed by western blot using an anti-Jub1p and an anti-α-Tubulin (α-Tub) antibody. (C) WT and *JUB1* KO (Δ*JUB1*) cells at the indicated time points were stained with an anti-Jub1p (red) and an anti-Pdd1p (green) antibody. DNA was stained with DAPI (blue). Arrowheads indicate the MIC (i), new MAC (na), and parental MAC (pa). The scale bars represent 10 μm. (D) The *JUB1* locus in WT and *JUB1* KO (Δ*JUB1*) cells are schematically shown at the top. Replacement of the *JUB1* coding with *neo4* was confirmed by genomic PCR using the primers (arrows). (E) Three stages of heterochromatin body formation (stage 1, pre-heterochromatin body; stage 2, heterochromatin body; stage 3, post-heterochromatin body) according to Pdd1p localization (green). DNA was stained with DAPI (magenta). The scale bars represent 2 μm. Exconjugants (n = 200) at 12, 15, 18, and 21 hpm from WT and *JUB1* KO cells were analyzed, and the averaged fractions from two independent experiments are shown. See also Figure S2.

We raised an antibody against Jub1p. By western blot, the antibody detected a protein appearing only during the late conjugation stages in wild-type cells (Figure 2A). This expression pattern is consistent with that of *JUB1* mRNA (Figure S2A). Moreover, the protein was not detected in *JUB1* knockout (KO) cells (see below for construction of *JUB1* KO cells) by both western blot (Figure 2B) and immunofluorescent staining (Figure 2C, Δ*JUB1*). We therefore conclude that the antibody specifically recognizes Jub1p.

Next, the localizations of Jub1p and Pdd1p were compared by immunofluorescent staining using the anti-Jub1p and an anti-Pdd1p antibody in wild-type (WT) cells (Figure 2C, WT). Jub1p and Pdd1p localized to the new MACs (“na” in Figure 2C) right after MAC enlargement (8 hpm). They were first distributed uniformly in the new MAC (8–10 hpm) but then gradually accumulated into foci at later stages (12–14 hpm). Subsequently, the Jub1p foci became smaller (16 hpm) and disappeared when the Pdd1p-positive heterochromatin body was eliminated (18 hpm).

### Jub1p Is Required for Heterochromatin Body Assembly

We established gene KO strains for *JUB1*, in which the entire *JUB1* protein-coding sequence in both the MAC and the MIC were replaced with a drug resistance gene, which was confirmed by genomic PCR (Figure 2D) and by northern blot (Figure S2B).

We analyzed heterochromatin body formation and the turnover process by categorizing exconjugants (progeny dissolved pairing) into three stages on the basis of the localization of Pdd1p (Figure 2E, top): in stage 1, Pdd1p is localized either homogeneously or in small puncta with continuous localization throughout the new MAC; in stage 2, Pdd1p is localized in discrete foci (heterochromatin bodies) with no detectable Pdd1p in the space between them; in stage 3, Pdd1p disappears completely. Approximately half of the exconjugants from WT cells were either in stage 1 or stage 2 at 12 hpm, and more than 80% of the exconjugants were in stage 3 at 15 hpm (Figure 2E, WT). By contrast, all exconjugants from the *JUB1* KO cells were in stage 1 even at 21 hpm (Figure 2E, Δ*JUB1*). Therefore, we conclude that Jub1p is required for heterochromatin body formation.

### Jub1p Is Dispensable for the Establishment of Heterochromatin on IESs

Heterochromatin body formation can be disrupted by inhibiting either the establishment of heterochromatin on individual IESs or the aggregation of the multiple heterochromatinized IES loci. To determine which of these processes is disrupted in the absence of Jub1p, heterochromatin assembly on IESs was analyzed.

**Figure 3:**
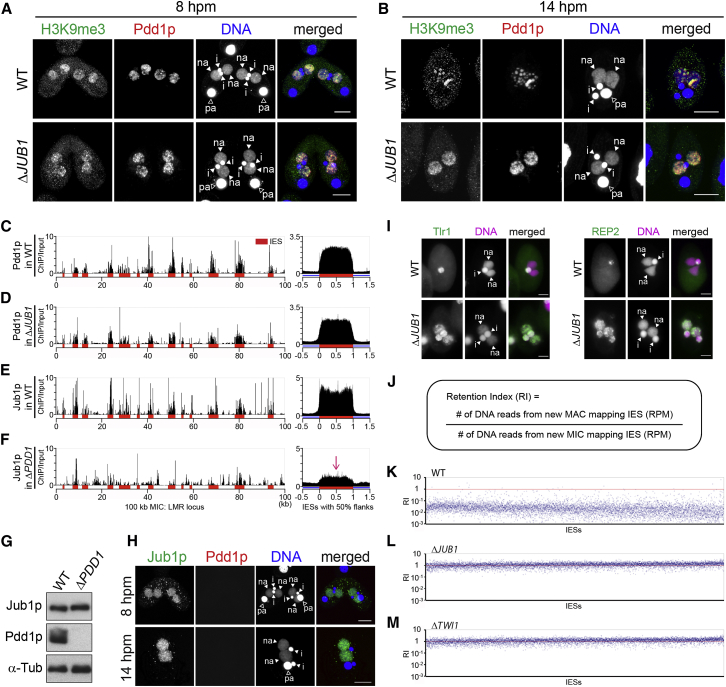
Jub1p Is Required for DNA Elimination but Not for Local Heterochromatin Establishment (A and B) WT and *JUB1* KO (Δ*JUB1*) cells at 8 hpm (A) and 14 hpm (B) were stained with an anti-H3K9me3 (green) and an anti-Pdd1p (red) antibody. DNA was stained with DAPI (blue). Arrowheads indicate the MIC (i), new MAC (na), and parental MAC (pa). The scale bars represent 10 μm. (C–F) Fragmented chromatin from the new MACs from WT (C and E), *JUB1* KO (Δ*JUB1*) (D) or *PDD1* KO (*ΔPDD1*) (F) cells at 12 hpm was immunoprecipitated with an anti-Pdd1p (C and D) or an anti-Jub1p (E and F) antibody. ChIP-seq reads were mapped to a 100 kb representative MIC locus (left; LMR locus) or to a modeled IES locus (right), which consisted of all predicted 1 to 5 kb IESs (red) and their flanks (blue). Fold enrichment relative to input is shown. (G) Proteins from WT and *PDD1* KO (Δ*PDD1*) cells at 12 hpm were analyzed by western blot with an anti-Jub1p, anti-Pdd1p, and anti-α-Tubulin antibody. (H) *PDD1* KO cells at 8 and 14 hpm were stained with an anti-Jub1p (green) and an anti-Pdd1p (red) antibody, and DNA was stained with DAPI (blue). Arrowheads indicate the MIC (i), new MAC (na), and parental MAC (pa). The scale bar represents 10 μm. (I) WT and *JUB1* KO (Δ*JUB1*) cells at 36 hpm were hybridized with probes complementary to Tlr1 or REP2 (green). DNA was stained with DAPI (magenta). Arrowheads indicate the MIC (i) and new MAC (na). The scale bar represents 5 μm. (J–M) RIs were calculated (J) for individual IESs in the new MACs from WT (K), *JUB1* KO (L), and *TWI1* KO (M) at 36 hpm. Red horizontal line indicates RI = 1 (no elimination). See also Figure S3.

We first analyzed two heterochromatin-associated histone modifications, H3K9me3 and H3K27me3, by immunofluorescent staining using an anti-H3K9me3 and an anti-H3K27me3 antibody, respectively. These modifications were accumulated similarly in the new MACs of WT and *JUB1* KO cells at 8 hpm (Figures 3A and S3A). At 14 hpm, these modifications were localized in the Pdd1p-positive heterochromatin bodies in WT cells, but remained homogeneously distributed in the new MACs in *JUB1* KO cells (Figures 3B and S3B). These results indicate that in the new MAC of *JUB1* KO cells, heterochromatin is formed but not assembled into heterochromatin bodies.

We further analyzed the heterochromatin formation by chromatin immunoprecipitation (ChIP) followed by high-throughput DNA sequencing (ChIP-seq). We purified new MACs by fluorescence-activated cell sorting (FACS) from cells at 12 hpm, when most IESs remain in the new MAC chromosomes in WT cells (Austerberry et al., 1984). As previously shown for a few IESs by ChIP-PCR (Chung and Yao, 2012, Liu et al., 2007, Taverna et al., 2002), our ChIP-seq analysis using an anti-Pdd1p antibody showed that in WT cells, Pdd1p was accumulated on most of the IESs in a representative 100 kb MIC genome locus (Figure 3C, left) as well as on a modeled IES in which all predicted 1–5 kb IES loci (5,606 loci total) were compiled (Figure 3C, right). In *JUB1* KO cells, Pdd1p was enriched normally on IESs (Figure 3D). Altogether, we conclude that Jub1p is not required for the proper formation of heterochromatin on IESs but is involved directly in heterochromatin body formation.

### Heterochromatin Is Important for the IES Localization of Jub1p

We performed ChIP-seq using the anti-Jub1p antibody and found that Jub1p was enriched on IESs in WT cells (Figure 3E). Because Jub1p has no obvious chromatin binding domains, it probably localizes on IESs through interaction with other heterochromatin components. Heterochromatin is mostly disrupted in the absence of the core heterochromatin component Pdd1p, which interacts with H3K9me3 and H3K27me3 and is required for the stable accumulation of these histone modifications (Liu et al., 2007, Taverna et al., 2002). We found that Jub1p was less enriched on IESs in *PDD1* KO cells than in WT cells (Figure 3F). This was not due to an overall reduction or aberrant cellular localization of Jub1p, because Jub1p accumulated normally (Figure 3G) and localized to the new MAC (Figure 3H) in *PDD1* KO cells. Therefore, we conclude that Pdd1p or a Pdd1p-dependent heterochromatin structure is important for the efficient recruitment of Jub1p to IESs.

### Jub1p Is Required for DNA Elimination and the Production of Viable Sexual Progeny

We next analyzed DNA elimination by DNA-FISH using probes complementary to the two moderately repeated IESs Tlr1 and REP2 (Fillingham et al., 2004, Wuitschick et al., 2002). At 36 hpm, both of the IESs were detected in the new MICs but not in the new MACs in the exconjugants from WT cells (Figure 3I, WT). By contrast, all exconjugants from *JUB1* KO cells showed staining for these IESs in the new MACs (Figure 3I, Δ*JUB1*), indicating that Jub1p is indispensable for DNA elimination of at least the Tlr1 and REP2 IESs.

To assess DNA elimination genome wide, we purified the new MACs from exconjugants at 36 hpm by FACS and analyzed their genomic DNA by high-throughput sequencing. As a reference, we also analyzed purified MICs from vegetative WT cells. As a measure for DNA elimination, a retention index (RI) (Figure 3J) was calculated for each IES by dividing the normalized number of reads mapping to an IES from the new MAC sample by those from the reference MIC samples. In the WT new MACs, RIs of most of the IESs were 0.001—0.1 (Figure 3K). Although, in theory, RIs of all IESs should be 0 in WT cells, MIC contamination (∼2%–10%) in our new MAC preparations probably made the RI higher. By contrast, in the new MACs from *JUB1* KO cells, most of the IESs had RIs of approximately 1 (Figure 3L), indicating that most, if not all, IESs are retained in the new MAC in the absence of Jub1p. A similar IES elimination defect was detected in *TWI1* KO cells (Figure 3M), in which the RNAi-mediated pathway required for heterochromatin formation is disrupted (Liu et al., 2004, Mochizuki et al., 2002). Altogether, we conclude that Jub1p is essential for the elimination of IESs genome wide.

Consistent with previous reports that DNA elimination is required for the production of viable sexual progeny (Cheng et al., 2010, Horrell and Chalker, 2014, Mochizuki et al., 2002, Nikiforov et al., 1999), *JUB1* KO cells produced no viable sexual progeny, whereas approximately 60% of WT mating pairs produced viable progeny (Figure S3C).

### Jub1p Facilitates Dephosphorylation of Pdd1p

**Figure 4:**
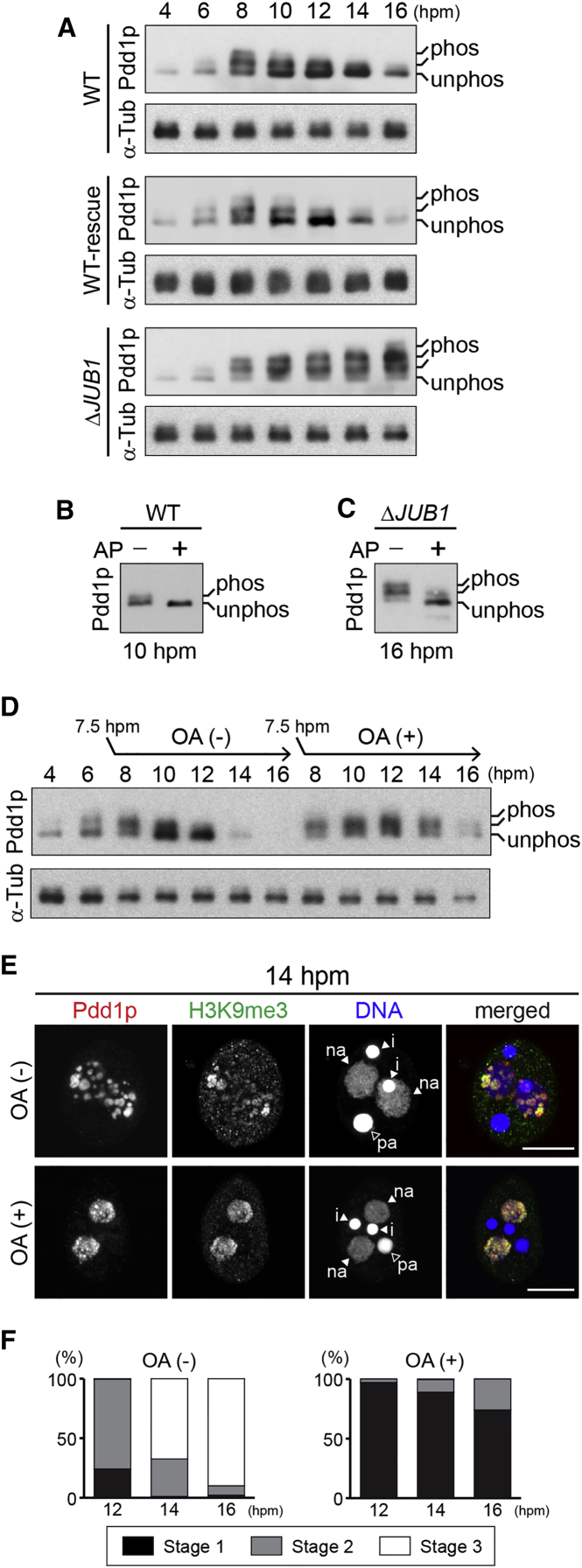
Pdd1p Dephosphorylation Is Inhibited in *JUB1* KO Cells (A) Proteins from WT, WT-rescue, and *JUB1* KO (Δ*JUB1*) cells in conjugation (4–16 hpm) were analyzed by western blot with an anti-Pdd1p antibody. Phosphorylated (phos) and unphosphorylated (unphos) Pdd1p are indicated. α-Tubulin (α-Tub) was analyzed as a control. (B and C) Lysates from WT cells at 10 hpm (B) and *JUB1* KO (Δ*JUB1*) cells at 16 hpm (C) were treated with (+) or without (−) alkaline phosphatase and analyzed as in (A). (D) Conjugating WT cells were treated from 7.5 hpm with (+) or without (−) OA, harvested at indicated time points, and analyzed as in (A). (E) Cells were treated as in (D) and stained at 14 hpm with anti-Pdd1p (red) and anti-H3K9me3 (green) antibody. DNA was stained with DAPI (blue). Arrowheads indicate the MIC (i), new MAC (na), and parental MAC (pa). The scale bars represent 10 μm. (F) Cells were treated as in (D), and heterochromatin body in exconjugants (n = 200) were analyzed as in Figure 2E. See also Figure S4.

In WT cells, at least three differently migrating Pdd1p species were detected by western blot using an anti-Pdd1p antibody at 8 hpm (Figure 4A, WT). As previously reported (Madireddi et al., 1996), all slower migrating Pdd1p disappeared after alkaline phosphatase treatment (Figure 4B), indicating that they were phosphorylated Pdd1p. From 10–14 hpm, these phosphorylated Pdd1p gradually diminished and unphosphorylated Pdd1p increased (Figure 4A, WT). We determined whether this phosphorylated-to-unphosphorylated (phos-unphos) transition is attributable to dephosphorylation of Pdd1p or instead to degradation of phosphorylated Pdd1p accompanied by de novo synthesis of unphosphorylated Pdd1p. To this aim, we analyzed Pdd1p from the WT *PDD1* gene that was introduced into the parental MAC of *PDD1* KO cells (WT-rescue). Although *PDD1* mRNA was expressed until 12 hpm in WT cells (Figure S4A), it was barely detected after 10 hpm in the WT-rescue cells (Figure S4B, WT-rescue). Even in these WT-rescue cells, in which little de novo Pdd1p synthesis was expected after 10 hpm, the phos-unphos transition of Pdd1p occurred between 10 and 12 hpm without a significant reduction in total Pdd1p (Figure 4A, WT-rescue). We therefore conclude that the phos-unphos transition of Pdd1p is mainly caused by dephosphorylation of Pdd1p.

Because the Pdd1p dephosphorylation coincides with heterochromatin body formation (Figures 1C and 2C) and because Jub1p is required for the formation of heterochromatin bodies (Figures 2E and 3B), we hypothesized that Jub1p directs heterochromatin body formation by promoting Pdd1p dephosphorylation. We therefore determined if Jub1p is required for the dephosphorylation of Pdd1p. At 8 hpm, similar amounts of slower migrating Pdd1p species were detected in WT and *JUB1* KO cells (Figure 4A). However, in later stages, these slower migrating Pdd1p species did not decline in *JUB1* KO cells, and additional slower migrating species appeared (Figure 4A, Δ*JUB1*; 10–16 hpm). All the slower migrating species in *JUB1* KO cells disappeared after alkaline phosphatase treatment (Figure 4C), confirming that they were phosphorylated Pdd1p. Jub1p has no identifiable phosphatase-related domain. Therefore Jub1p facilitates the dephosphorylation of Pdd1p most likely by recruiting some phosphatase(s).

### Inhibition of Pdd1p Dephosphorylation Disturbs Heterochromatin Body Formation

To test the importance of the dephosphorylation of Pdd1p, we treated cells with Okadaic acid (OA), an inhibitor for serine (Ser)/threonine (Thr) protein phosphatase 1 and 2A. From 7.5 hpm, just before the highest level of Pdd1p phosphorylation was observed (Figure 4A), conjugating WT cells were incubated with OA. The phosphorylated Pdd1p species remained at least till 16 hpm with OA (Figure 4D, OA+), while they were mostly disappeared by 12 hpm without OA (Figure 4D, OA−), indicating that OA inhibits the dephosphorylation of Pdd1p. In the OA-treated cells, Pdd1p and H3K9me3 were accumulated in the new MAC (Figure 4E), but cells showing heterochromatin body (stage 2) were greatly reduced (Figure 4F). Moreover DNA-FISH analyses for Tlr1 and REP2 IESs showed that most (63% for Tlr1, 87% for REP2) of the OA-treated cells did not finish DNA elimination (Figure S4C). These results are consistent with the idea that Pdd1p dephosphorylation promotes heterochromatin body formation.

### Pdd1p Is Mostly Phosphorylated in Unconserved Regions

**Figure 5:**
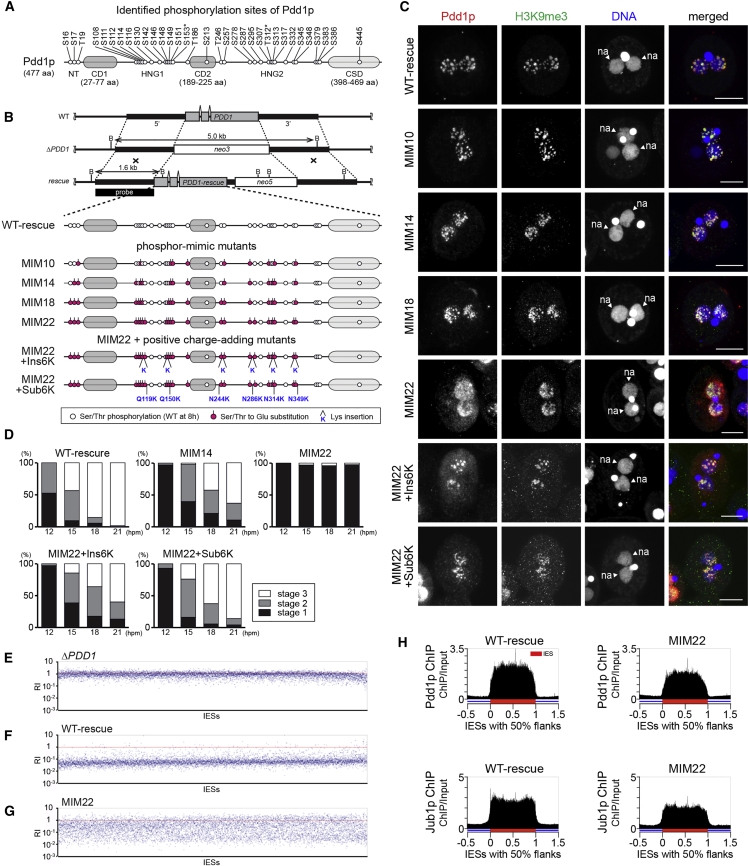
Phosphor-Mimic Pdd1p Mutants Inhibit Heterochromatin Body Formation (A) Thirty-one phosphorylated Ser (S)/Thr (T) residues of Pdd1p in WT cells at 8 hpm identified in this study and additional 2 residues (marked by asterisks) identified by Tian et al. (2014) are shown as open circles. HNG1 and HNG2 indicate the non-conserved hinge regions between CD1 and CD2 and between CD2 and CSD, respectively. (B) The WT, KO (Δ*PDD1*), and rescued loci (top) and the proteins expressed from the rescue constructs (bottom). Magenta circles indicate the introduced phosphor-mimic mutations (Ser/Thr to Glu). Lys insertions are indicated with “K,” and substitutions from Gln (Q) or Asn (N) to Lys (K) are indicated with the positions. (C) Cells at 14 hpm were stained with anti-Pdd1p (red) and anti-H3K9me3 (green) antibody, and DNA was stained with DAPI (blue). The new MACs are marked with an arrowhead (na). The scale bars represent 10 μm. (D) Heterochromatin body in the exconjugants (n = 200) from the indicated *PDD1* mutants was analyzed as in Figure 2E. (E–G) The RI of each IES in *PDD1* KO (E), WT-rescue (F), and MIM22 (G) strains was calculated as in Figure 3J. Red horizontal line indicates RI = 1 (no elimination). (H) Localization of Pdd1p and Jub1p in the new MACs of WT-rescue and MIM22 cells at 12 hpm were analyzed by ChIP-seq. See also Figure S5.

Although the data above suggest that the dephosphorylation of Pdd1p facilitates heterochromatin body formation, it is also possible that the loss of Jub1p and the OA treatment inhibit heterochromatin body formation independently of Pdd1p dephosphorylation. Therefore, we aimed to analyze the role of Pdd1p dephosphorylation by directly mutating the phosphorylated residues of Pdd1p. For this purpose, we identified the phosphorylated residues of Pdd1p. Pdd1p was immunoprecipitated from WT cells at 8 hpm, when the highest phosphorylation level of Pdd1p was observed (Figure 4A), and mass spectrometry analyses detected 31 phosphorylated Ser/Thr residues in Pdd1p. Another study has identified 10 phosphorylated Ser/Thr residues in Pdd1p (Tian et al., 2014), 2 of which were not identified in our analysis. Therefore, in total, 33 phosphorylated residues of Pdd1p have been identified (Figure 5A, open circles). Pdd1p has 2 chromodomains (CD1 and CD2) and a chromoshadow domain (CSD) (Callebaut et al., 1997), and most (31 of 33) of the identified residues are located in the N-terminal and hinge regions outside of these domains.

### Experimental System to Analyze In Vivo Function of Pdd1p

To analyze the function of different Pdd1p mutants in vivo, we established a system in which the KO loci in the MAC of *PDD1* KO cells were replaced by constructs expressing *PDD1* genes from the endogenous *PDD1* promoter (Figure 5B). We validated this system by expressing WT Pdd1p (WT-rescue; Figures 5B and S5A). These WT-rescue strains formed heterochromatin bodies containing Pdd1p and H3K9me3 in the new MACs at 14 hpm (Figure 5C, WT-rescue). A time course study (Figure 5D, WT-rescue) showed that approximately half of the exconjugants from the WT-rescue strains at 12 hpm had heterochromatin bodies (stage 2), and the heterochromatin bodies disappeared (stage 3) from most of the exconjugants by 21 hpm. Whole-genome sequencing of the new MACs at 36 hpm indicated that the DNA elimination defect of the *PDD1* KO cells (Figure 5E) was rescued in the WT-rescue strains (Figure 5F). Consistently, DNA-FISH analysis at 36 hpm indicated that although Tlr1 IESs remained in the new MACs of *PDD1* KO cells, they mostly disappeared in the new MACs of the WT-rescue strains (Figure S5B). Moreover, the WT-rescue cells produced viable sexual progeny (Figure S5C). Altogether, the expression of WT Pdd1p from the parental MAC in the *PDD1* KO background was sufficient to restore all essential processes for DNA elimination, including the formation of heterochromatin bodies. Therefore, the rescue system can be used to analyze the functionalities of Pdd1p mutants in vivo.

### Pdd1p Dephosphorylation Promotes Heterochromatin Body Formation by Reducing Net Negative Charge

We generated a series of constructs to express Pdd1p carrying phosphor-mimic mutations, in which 10, 14, 18, or 22 of the identified phosphorylated Ser/Thr residues in the N-terminal and hinge regions (NT, HNG1 and 2, respectively; Figure 5A) were substituted with glutamic acid (Glu) (MIM10, MIM14, MIM18, and MIM22, respectively; Figure 5B), and introduced them into *PDD1* KO cells. Cells expressing MIM10, MIM14, and MIM18 formed exconjugants with heterochromatin bodies at 14 hpm (Figure 5C). Time course analyses (Figure 5D) revealed that although the formation of heterochromatin bodies was delayed in the MIM14 strain, approximately half of the exconjugants from this strain formed heterochromatin bodies (stage 2) at 15 hpm, and the heterochromatin bodies disappeared (stage 3) in some of the exconjugants at later stages. By contrast, in the cells expressing MIM22, no heterochromatin bodies were detected at any of the time points examined (Figures 5C and 5D, MIM22). Nonetheless, Pdd1p and Jub1p accumulated similarly in the WT-rescue and MIM22 strains (Figure S5D) and localized properly on the IESs (Figure 5H). Moreover, in vitro peptide pull-down assay showed that bacterially expressed full-length WT (WT_FL) and the MIM22 mutant (MIM22_FL) Pdd1p similarly bound to the peptides corresponding to H3K9me3 and H3K27me3 (Figure S5E). Therefore, phosphor-mimic mutations in the unconserved regions do not affect the interaction of Pdd1p with the methylated histones. We conclude that the failure of the MIM22 mutant to restore heterochromatin body formation is due neither to instabilities in Pdd1p or Jub1p nor to their absence from chromatin but to a functional disturbance in Pdd1p’s heterochromatin body-forming activity. Although the correlative occurrence of the dephosphorylation of Pdd1p and heterochromatin body has been suggested (Madireddi et al., 1996, Shieh and Chalker, 2013), this is the first direct demonstration showing the functional link between the two events.

The results above indicated that the more phosphor-mimic mutations in Pdd1p, the stronger the defects in heterochromatin body formation. We therefore hypothesized that the lack of heterochromatin body-forming ability in the phosphor-mimic Pdd1p mutants was due to the increase in their net negative charge. If this was the case, additional mutations supplying positive charges to the phosphor-mimic Pdd1p mutant should restore heterochromatin body formation. To test this idea, we expressed MIM22 with six Lys insertions (MIM22+Ins6K; Figure 5B) or MIM22 with six substitutions of glutamine and asparagine for Lys (MIM22+Sub6K; Figure 5B) in the *PDD1* KO background. In these strains, the formation of heterochromatin bodies was restored to a level similar to the strains expressing MIM14 (Figures 5C and 5D), indicating that the lack of heterochromatin body-forming ability of MIM22 was due not to structural disturbances, if any, caused by the 22 amino acid substitutions but to the constitutive increase in the net negative charge. Altogether, we conclude that the dephosphorylation of Pdd1p facilitates heterochromatin body formation by reducing the net negative charge of Pdd1p.

### Pdd1p Dephosphorylation Is Important for DNA Elimination and Progeny Viability

DNA-FISH analyses showed that Tlr1 IESs were completely eliminated in the exconjugants from WT-rescue and MIM10 cells. In contrast, only 40%, 19%, and 1% of the exconjugants from MIM14, MIM18, and MIM22 cells, respectively, completed DNA elimination of the Tlr1 IESs (Figure S5B). The DNA elimination defects of these phosphor-mimic mutants correlated with their abilities to produce viable sexual progeny: 71%, 10%, and 0.3% of mating pairs from WT-rescue, MIM10, and MIM14 cells, respectively, produced viable progeny, and no viable progeny were obtained from MIM18 and MIM22 cells (Figure S5C). Moreover, in the cells expressing the positive charge-added MIM22 mutants (MIM22+Ins6K and MIM22+Sub6K), both IES elimination and progeny production were restored to the level of MIM14 cells (Figures S5B and S5C).

We next assessed genome-wide DNA elimination of the new MACs of MIM22 cells at 36 hpm, as described above. RI scores of different IESs were variable, ranging from 0.01—1 (Figure 5G), indicating that different IESs were affected differently by the MIM22 mutation. This is in contrast with *JUB1* KO cells, in which eliminations of most of the IESs were inhibited (Figure 3L). Therefore, although the MIM22 mutation phenocopied *JUB1* KO in terms of heterochromatin body formation (Figures 2E, 3B, 5C, and 5D) and progeny viability (Figures S3C and S5C), the DNA elimination defect in MIM22 was less severe than that in *JUB1* KO cells. This difference might be because Jub1p can still regulate phosphorylation of the non-mutated Ser/Thr residues of MIM22.

### Phosphorylation Does Not Affect the Self-Interaction of Pdd1p

**Figure 6:**
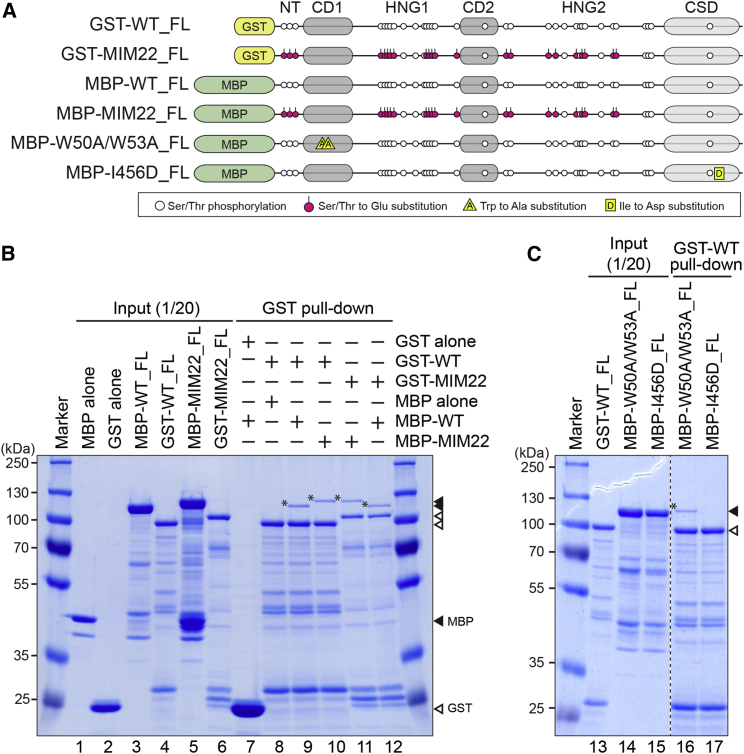
Phosphor-Mimetic Mutations Do Not Inhibit Self-Interaction of Pdd1p (A) The recombinant Pdd1p proteins used for GST pull-down assays. Magenta circles indicate the introduced phosphor-mimic mutations (Ser/Thr to Glu). Trp to Ala and Ile to Asp substitutions are indicated as yellow triangles and squares, respectively. (B and C) Input proteins (lanes 1–6 and 13–15) and proteins co-precipitated with the indicated GST-tagged proteins (lanes 7–12 and 16–17) were analyzed by SDS-PAGE followed by Coomassie blue staining. Filled and open arrowheads indicate MBP- and GST-tagged proteins, respectively. Asterisks indicate co-precipitated MBP-tagged proteins.

We hypothesized that Pdd1p dephosphorylation might facilitate heterochromatin body formation by promoting direct interaction between the hinge regions of two Pdd1p molecules, which is otherwise inhibited by the negatively charged phosphate groups. To test this idea, we performed pull-down assays using bacterially expressed, WT Pdd1p (WT_FL) and the MIM22 phosphor-mimic Pdd1p mutant (MIM22_FL) (Figure 6A). Maltose binding protein-tagged WT_FL (MBP-WT_FL) and MIM22_FL (MBP-MIM22_FL) were similarly co-precipitated with glutathione S-transferase-tagged WT-FL (GST-WT_FL) (Figure 6B, lanes 9 and 10, asterisks). MBP-WT_FL and MBP-MIM22_FL were also co-precipitated with GST-MIM22_FL (Figure 6B, lanes 11 and 12, asterisks). These results indicate that the phosphor-mimic mutations do not prevent association of two Pdd1p molecules.

Many HP1 family proteins form homodimers through their CSDs (Cowieson et al., 2000). In addition, Swi6, the fission yeast HP1, also multimerizes through its CD (Canzio et al., 2013). We therefore speculated that Pdd1p might also multimerize through a CD-CD or CSD-CSD interaction, which might compensate for the effect of the phosphor-mimic mutations on a hinge-hinge interaction in vitro. To test this possibility, we performed pull-down assays using MBP-Pdd1p carrying either two amino acid substitutions (W50A/W53A) in CD1 or a substitution (I456D) in CSD (Figure 6A) that inhibit the CD-CD and the CSD-CSD interaction in Swi6, respectively (Canzio et al., 2013, Cowieson et al., 2000). The I456D mutation inhibited the co-precipitation of MBP-Pdd1p with GST-WT_FL (Figure 6C, lane 17), but the W50A/W53A mutations did not (Figure 6C, lane 16). Therefore, the in vitro self-interaction of Pdd1p is mediated solely by CSD, and the other domains, including the hinge regions, do not support self-interaction. We conclude that the dephosphorylation of Pdd1p induces heterochromatin body formation not by regulating the multimerization of Pdd1p but through some other mechanism.

The dimerization of HP1 proteins through their CSDs facilitates local heterochromatin compaction (Canzio et al., 2011, Cowieson et al., 2000). Because Pdd1p also homo-multimerizes through its CSD and the phosphor-mimic mutations of Pdd1p do not affect this homo-multimerization, dephosphorylation of Pdd1p is not likely involved in the local compaction of heterochromatin but rather specifically involved in heterochromatin body formation. It has been reported that the Pdd1p CSD mutation I456D causes a defect in heterochromatin body formation in vivo (Schwope and Chalker, 2014). This defect may arise because Pdd1p homo-multimerization is important for the local compaction of heterochromatin, which is a prerequisite for heterochromatin body formation.

### Phosphor-Mimic Mutations Inhibit RNA Binding of Pdd1p

The hinge regions of mammalian HP1α and yeast Swi6 bind to non-coding RNAs (ncRNAs) (Keller et al., 2012, Maison et al., 2011), and RNase treatment of permeabilized mouse cells disassembles the chromocenter (Maison et al., 2002). Because ncRNAs are transcribed from IESs in the new MAC in *Tetrahymena* (Aronica et al., 2008). We thought that ncRNAs might mediate the Pdd1p-Pdd1p interaction and thus the formation of heterochromatin bodies.

**Figure 7:**
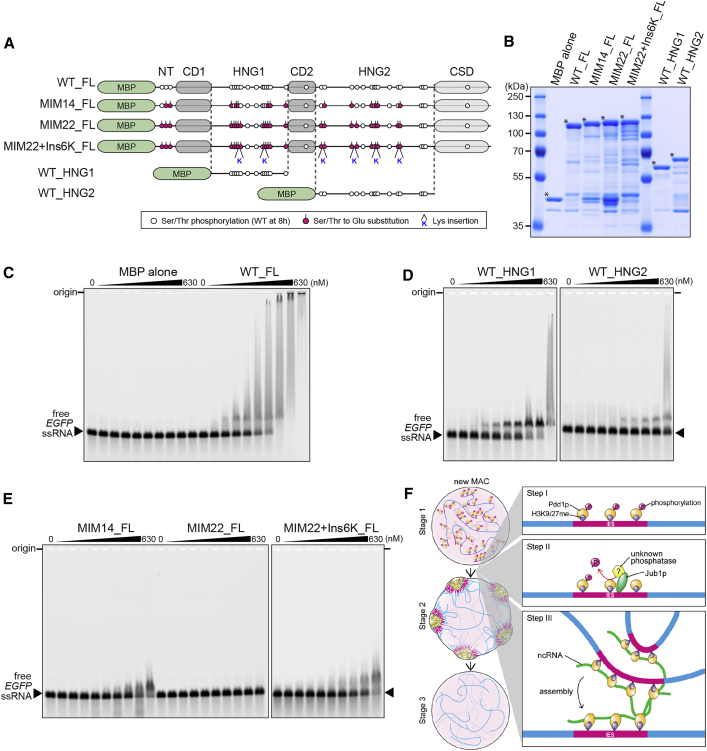
Hinge Regions of Pdd1p Bind to RNA through Their Net-Positive Charge (A) The recombinant Pdd1p proteins used for EMSA. Magenta circles indicate the introduced phosphor-mimic mutations (Ser/Thr to Glu). The Lys insertions are indicated with “K.” (B) Proteins used were analyzed by SDS-PAGE followed by Coomassie blue staining. Asterisks indicate MBP-tagged Pdd1p proteins or MBP alone. (C–E) A 723-nt *EGFP* ssRNA (10.4 nM) was titrated with the indicated proteins (0, 10.4, 26.3, 52.5, 78.3, 104, 158, 210, 420, and 630 nM) and separated by agarose gel electrophoresis. (F) A model for heterochromatin body formation. See also Figure S6.

We performed an electrophoretic mobility shift assay (EMSA) to test this possibility. The recombinant WT Pdd1p (WT_FL; Figures 7A and 7B) bound to a 723-nt single-stranded RNA (ssRNA) complementary to *EGFP* (*EGFP* ssRNA) (*K*_d_ = 65 ± 15 nM) (Figure 7C). Multiple shifts were detected in this assay, indicating that more than one Pdd1p molecule interacts with a single *EGFP* ssRNA. WT_FL also bound to a 1,305-nt ssRNA complementary to Cal IES (Cal IES ssRNA) (Figure S6A). Furthermore, the isolated two hinge regions of Pdd1p (WT_HNG1 and WT_HNG2; Figures 7A and 7B) also bound to the *EGFP* ssRNA (*K*_d_ = 130 ± 20 nM, *K*_d_ > 630 nM, respectively) (Figure 7D). We conclude that Pdd1p interacts with RNA through its hinge regions in a sequence-independent manner.

Similar EMSA experiments were performed with Pdd1p harboring 14 or 22 phosphor-mimic mutations (MIM14_FL, MIM22_FL; Figures 7A and 7B). Note that the MIM14 and MIM22 show mild and severe heterochromatin body formation defects in vivo, respectively (Figure 5D). MIM14_FL exhibited weaker interaction with the *EGFP* ssRNA (*K*_d_ = 340 ± 14 nM) compared with WT-FL, and MIM22_FL showed no detectable interaction with the RNA (Figure 7E). Therefore, the phosphor-mimic mutations disrupt the RNA-Pdd1p interaction. Importantly, MIM22+Ins6K_FL, which had insertions of 6 Lys residues into MIM22_FL (Figures 7A and 7B), interacted with the *EGFP* ssRNA with the affinity (*K*_d_ = 347 ± 31 nM) similar to that of MIM14_FL (Figure 7E), indicating that Pdd1p interacts with RNA not via its specific residues but electrostatically through the global positive charge of its hinge regions.

## Discussion

In this study, we showed that the heterochromatin body component Jub1p facilitates the dephosphorylation of Pdd1p. Both *JUB1* KO and phosphor-mimic mutations of Pdd1p severely compromised heterochromatin body formation and DNA elimination without affecting local heterochromatin assembly. As far as we know, these mutants are the first experimental system in any eukaryote by which we can genetically separate roles of heterochromatin body from roles of the underlying local heterochromatin. This study therefore provides the first evidence that heterochromatin body per se has an essential biological function.

### RNA-Glue Model for Heterochromatin Body Formation in *Tetrahymena*

We propose a model for the molecular mechanism of heterochromatin body assembly during new MAC differentiation in *Tetrahymena* (Figure 7F). First, Pdd1p is deposited onto IESs through its interaction with H3K9/27me to form heterochromatin (step I). We believe Pdd1p is phosphorylated prior to its chromatin deposition because Pdd1p was phosphorylated in *EZL1* and *TWI1* KO cells (Figure S6B), which are defective in H3K9/27me accumulation (Liu et al., 2004, Liu et al., 2007). At this stage (stage 1), heterochromatinized IESs are distributed homogeneously in the new MAC. Then, Jub1p localizes to heterochromatin and recruits a phosphatase (or phosphatases) to trigger Pdd1p dephosphorylation (step II). This dephosphorylation reduces the net negative charge of the hinge regions of Pdd1p and restores its RNA binding activity (step III). Because one RNA molecule can interact with multiple Pdd1p molecules (Figure 7C), we propose that the Pdd1p-RNA interaction “glues” multiple IESs into a heterochromatin body (step III, stage 2). Finally, IESs are excised within the heterochromatin body compartments (stage 3).

Although this study clearly demonstrates that the dephosphorylation of Pdd1p plays a pivotal role in heterochromatin body formation, why Pdd1p must be phosphorylated in the first place remains unclear. ChIP-seq analysis indicated that a phosphorylation-defective Pdd1p mutant, in which 26 phosphorylated Ser/Thr residues were substituted with alanine, localized normally on IESs (unpublished data). Therefore, Pdd1p phosphorylation is unlikely to be required for its chromatin deposition but might only be required to regulate the timing of heterochromatin body formation. Alternatively, it may establish a chromatin environment for some downstream event.

Our efforts to identify the phosphatase(s) of Pdd1p have been unsuccessful. It has been proposed that Pdd1p dephosphorylation and heterochromatin body formation are triggered by DNA elimination, based on the observation that UV irradiation induced both of these events in the DNA elimination-defective *LIA5* KO cells (Shieh and Chalker, 2013). Although this notion is seemingly contradictory to the fact that elimination of most IESs occurs after heterochromatin body formation (Austerberry et al., 1984), some IESs might be eliminated prior to heterochromatin body formation, and DNA damage signaling caused by such DNA elimination might upregulate phosphatase(s) for Pdd1p.

Currently, it is unclear which RNA species interact with Pdd1p in vivo. We previously showed that at least some IESs in the new MAC are transcribed to produce ncRNAs (Aronica et al., 2008). Although these ncRNAs were suggested to be nascent transcripts required for the interaction between the RNAi-machinery and chromatin (Aronica et al., 2008), they might also interact with Pdd1p. Future work should comprehensively identify Pdd1p-associated RNAs in vivo.

### Does RNA Glue Heterochromatin Bodies in Other Eukaryotes?

The involvement of ncRNAs in heterochromatin body dynamics is not a new concept but has been reported in other eukaryotes: the mouse HP1α binds to ncRNA from major satellites, which serves as a structural platform for recruiting heterochromatin modulators for the assembly of chromocenters (Maison et al., 2002, Maison et al., 2011); similarly, Xist coats the inactive X chromosome and plays an important role in recruiting factors required for Barr body formation (Hall and Lawrence, 2010). However, it is unclear whether these ncRNAs are directly involved in heterochromatin body formation or indirectly through regulation of local heterochromatin. In *Tetrahymena*, an RNA binding-defective Pdd1p mutant (MIM22) inhibits heterochromatin body formation without affecting local heterochromatin formation in vivo (Figures 5C, 5D, and 5H) and Pdd1p homo-multimerization in vitro (Figure 6B). Therefore, we believe this study provides the first clear demonstration that RNA interaction with a heterochromatin component plays a direct role in heterochromatin body formation.

As for *Tetrahymena* Pdd1p, positively charged residues in the hinge region of HP1 proteins in yeast (Swi6) and mammals (HP1α) are critical for their electrostatic interactions with RNA (Keller et al., 2012, Muchardt et al., 2002), and accumulating evidences from phosphoproteomics suggest that most of the phosphorylated residues of HP1 proteins are in the unconserved region (Dephoure et al., 2008, Shimada et al., 2009, Wilson-Grady et al., 2008, Zhai et al., 2008). Therefore, phosphorylation of HP1 in many eukaryotes might also downregulate their RNA-binding activities as it does in Pdd1p. In this context, it will be interesting to study whether phosphorylations of the hinge regions of HP1 proteins affect their RNA binding and whether such regulation also plays a role in heterochromatin body formation.

## Experimental Procedures

For detailed experimental procedures, see Supplemental Experimental Procedures.

### Protein and IES Localization Analyses

Immunofluorescent staining and DNA-FISH were performed as described (Loidl and Scherthan, 2004, Noto et al., 2010). For immuno-DNA-FISH, fixed cells were first hybridized with a Cy3-labeled Tlr1 probe and then used for immunofluorescent staining.

### ChIP-Seq and DNA Elimination Analysis

For ChIP-seq, the new MACs at 12 hpm were fixed with Di(N-succinimidyl) glutarate and paraformaldehyde and purified by FACS. DNA library was produced from immunoprecipitated chromatin. For genome-wide DNA elimination analysis, DNA libraries were generated from the new MACs from 36 hpm exconjugants and the MICs from starved WT cells. For the both analyses, 50-nt single sequence reads were generated by a HiSeq2000 platform. The MIC genome sequence (version 2) was obtained from the *Tetrahymena* Comparative Sequencing Project (Broad Institute of MIT and Harvard).

### Progeny Viability Test

For *JUB1* KO strains, a blasticidin S (bs)-resistance marker was introduced into the MAC. Conjugating pairs were isolated at 8 hpm and bs-sensitive cells were determined as sexual progeny. For the *PDD1* mutants, isolated cells were examined for their resistance to paromomycin without CdCl_2_.

### Identification of Pdd1p Phosphorylation Sites

Pdd1p in WT cells at 8 hpm was immunoprecipitated with an anti-Pdd1p antibody, digested with trypsin, chymotrypsin or subtilisin, and analyzed by mass spectrometry.

### Pull-Down Assays

For peptide pull-down assay, MBP-tagged proteins were precipitated with beads coupled with peptides corresponding to the histone H3 N-terminal tail and analyzed by western blot with an anti-MBP antibody. Mean enrichment was calculated from three independent experiments. For GST pull-down assay, GST- and MBP-tagged proteins were mixed, purified with glutathione beads, and analyzed by SDS-PAGE followed by Coomassie blue staining.

### EMSA

Fluorescein-12 labeled ssRNA complementary to *EGFP* (723 nt) or Cal IES (1,305 nt) was incubated with MBP-tagged proteins and separated by agarose gel electrophoresis, and the RNA was quantified. Means of dissociation constant were calculated from more than two independent experiments.

## Accession Numbers

The accession number for the sequence data reported in this paper is GEO: GSE70083.

## Supplemental Information

Document S1. Supplemental Experimental Procedures and Figures S1–S6

Data S1. Summary of the Protein Localization Screen, Nuclear Events during the Life Cycle of *Tetrahymena thermophila*, and Localizations of EGFP-Tagged Proteins

Document S2. Article plus Supplemental Information
