# Supplementary material for: Phosphorylation of an HP1-like Protein Regulates Heterochromatin Body Assembly for DNA Elimination
Source: Dev Cell. 2015 Dec 21;35(6):775–88. doi: 10.1016/j.devcel.2015.11.017 (PMC4695338; doi:10.1016/j.devcel.2015.11.017)
Supplement: Document S1. Supplemental Experimental Procedures and Figures S1–S6 [file mmc1.pdf]

Developmental Cell

Supplemental Information

Phosphorylation of an HP1-like Protein Regulates Heterochromatin Body Assembly for DNA Elimination

Kensuke Kataoka and Kazufumi Mochizuki

Figure S1

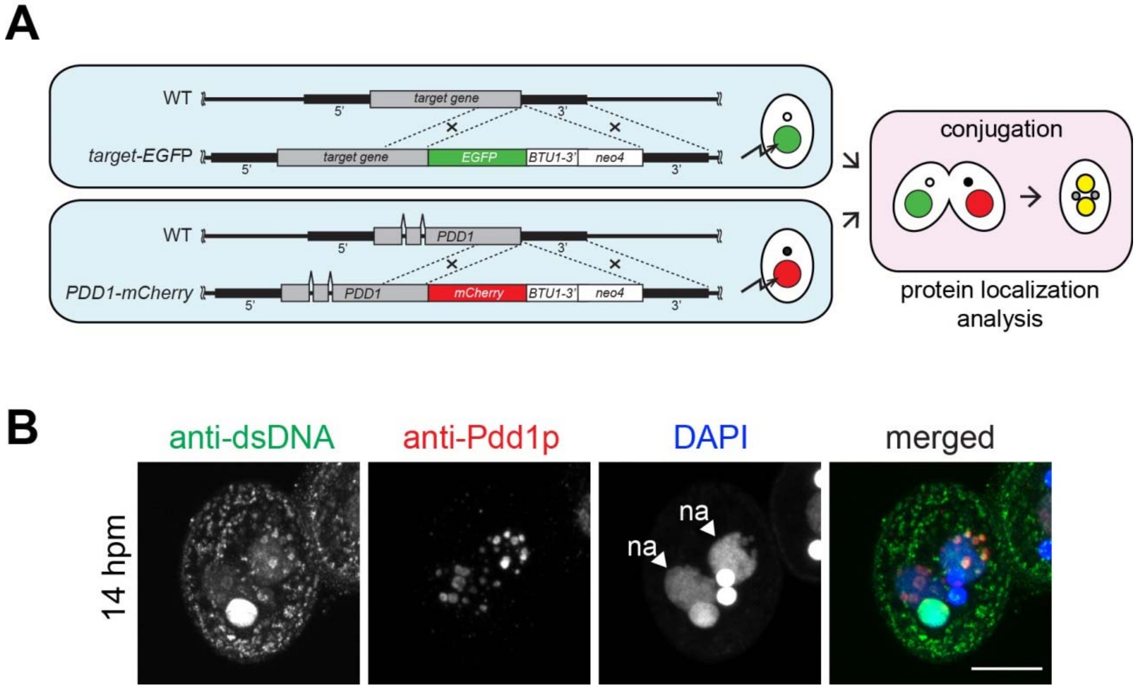

Figure S1, related to Figure 1. Experimental design for the protein localization screen
(A) A schematic representation of the protein localization screen. (Left top) The *EGFP* gene followed by the *BTU1* 3'UTR and the *neo4* drug-resistance cassette were inserted before the stop codon of an endogenous target locus in the MAC by homologous recombination. To visualize heterochromatin bodies, cells expressing an EGFP-tagged protein were crossed with cells expressing Pdd1p-mCherry (Left bottom). The localizations of the EGFP-tagged protein and Pdd1p-mCherry were observable within the same cell because the cytoplasm of conjugating cells is connected. **(B)** A wild-type cell at 14 hpm was immunostained with an anti-double-stranded DNA (dsDNA) antibody (green) and an anti-Pdd1p antibody (red). DNA was counter-stained with DAPI (blue). The new MACs are marked by arrowheads with “na”. The scale bar represents 10 μm.

Figure S2

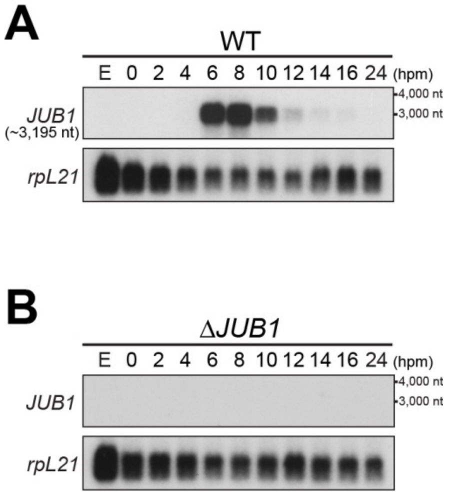

Figure S2, related to Figure 2. Northern blot analyses of *JUB1* mRNA
(A, B) *JUB1* mRNA (~3,195 nt) from exponentially growing (E), starved (0 hpm) or conjugating (2-24 hpm) wild-type (WT) (A) and *JUB1* KO (Δ*JUB1*) (B) cells were analyzed by northern blot. Constitutively expressed *rpL21* was analyzed as a control.

Figure S3

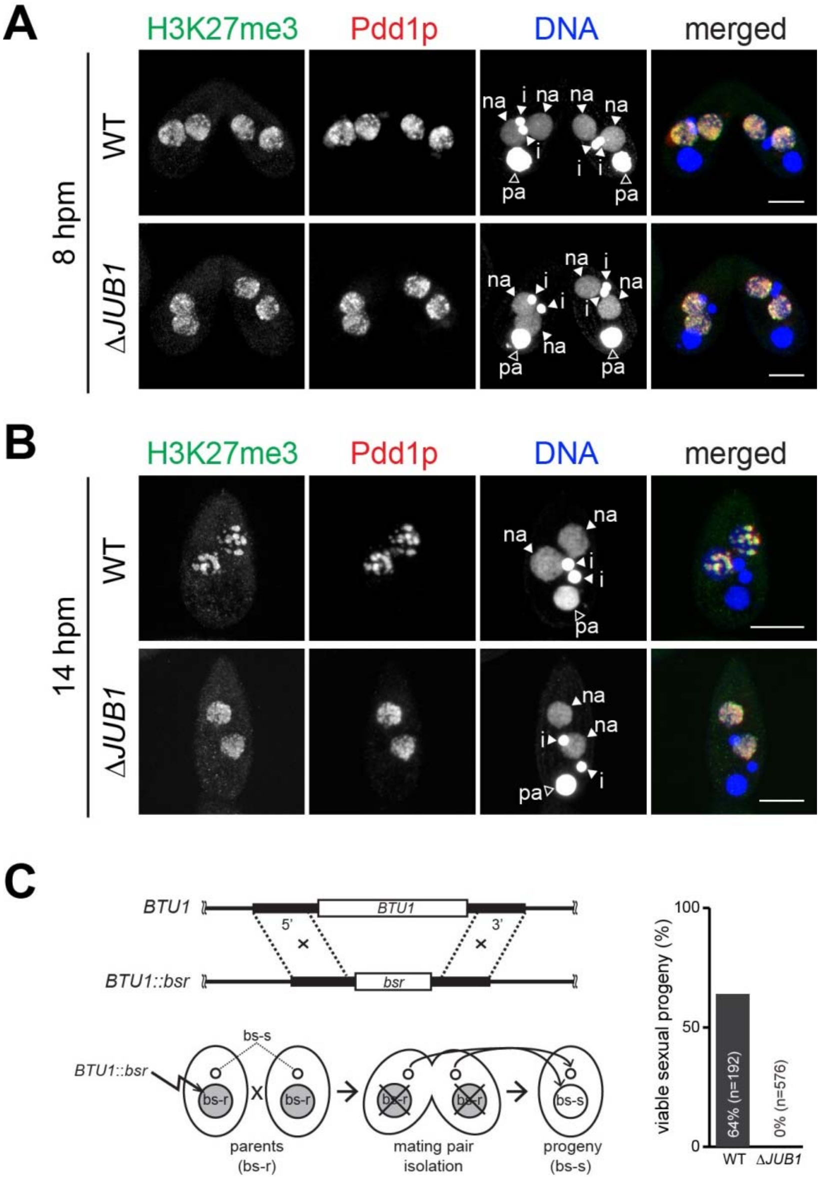

Figure S3, related to Figure 3. Analyses of methylated histones and progeny viability
(A, B) Wild-type (WT) and *JUB1* KO (Δ*JUB1*) cells at 8 hpm (A) and 14 hpm (B) were immunostained with anti-H3K27me3 (green) and anti-Pdd1p (red) antibodies. DNA was counter-stained with DAPI (blue). Arrowheads indicate the MIC (i), new MAC (na) and parental MAC (pa). Scale bars represent 10 µm. **(C)** Viability tests for the sexual progeny of *JUB1* KO cells. (Left top) A schematic representation of the *BTU1* locus. MAC loci encoding the non-essential *BTU1* gene in *JUB1* KO (Δ*JUB1*) and wild-type (WT) cells were replaced with the Blasticidin S (bs) resistance marker cassette *bsr* by homologous recombination. (Left bottom) A schematic representation of the experimental design of the assay. The bs-resistant (bs-r) cells (parents) were induced to conjugate, and the bs sensitivity of the cells grown from the isolated mating pair was assessed. The progeny are bs sensitive (bs-s) because the parental MAC carrying the *bsr* cassette is destroyed during conjugation (See Supplementary Experimental Procedures for details). (Right) The average percentages of isolated mating pairs that produced bs-s progeny from a wild-type cross and 3 independent *JUB1* KO crosses are shown.

Figure S4

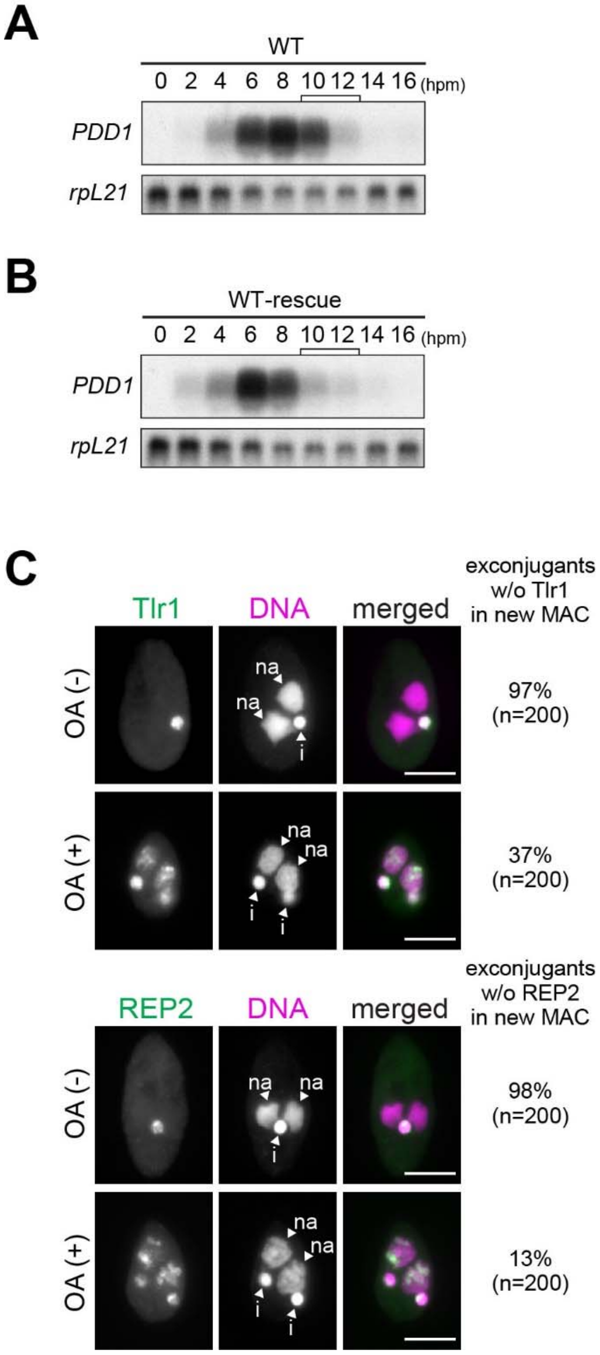

Figure S4, related to Figure 4. Northern blot analyses of *PDD1* mRNA in wild-type and WT-rescue cells, and DNA FISH analyses in wild-type cells treated with a phosphatase inhibitor

(A, B) *PDD1* mRNA from starved (0 hpm) or conjugating (2-16 hpm) wild-type (WT) (A) and WT-rescue (B) cells was analyzed by northern blot. Constitutively expressed rpL21 was analyzed as a control. **(C)** (Left) Exconjugants from wild-type treated with or without Okadaic acid [OA (+) or OA (-)] at 36 hpm were hybridized with probes complementary to Tlr1 or REP2 (green). DNA was counter-stained with DAPI (magenta). Arrowheads indicate the MIC (i), new MAC (na). Scale bar represents 10 µm. (Right) the average percentages of exconjugants that do not have Tlr1 or REP2 signal in the new MAC from 2 independent crosses (n=200) are shown.

Figure S5

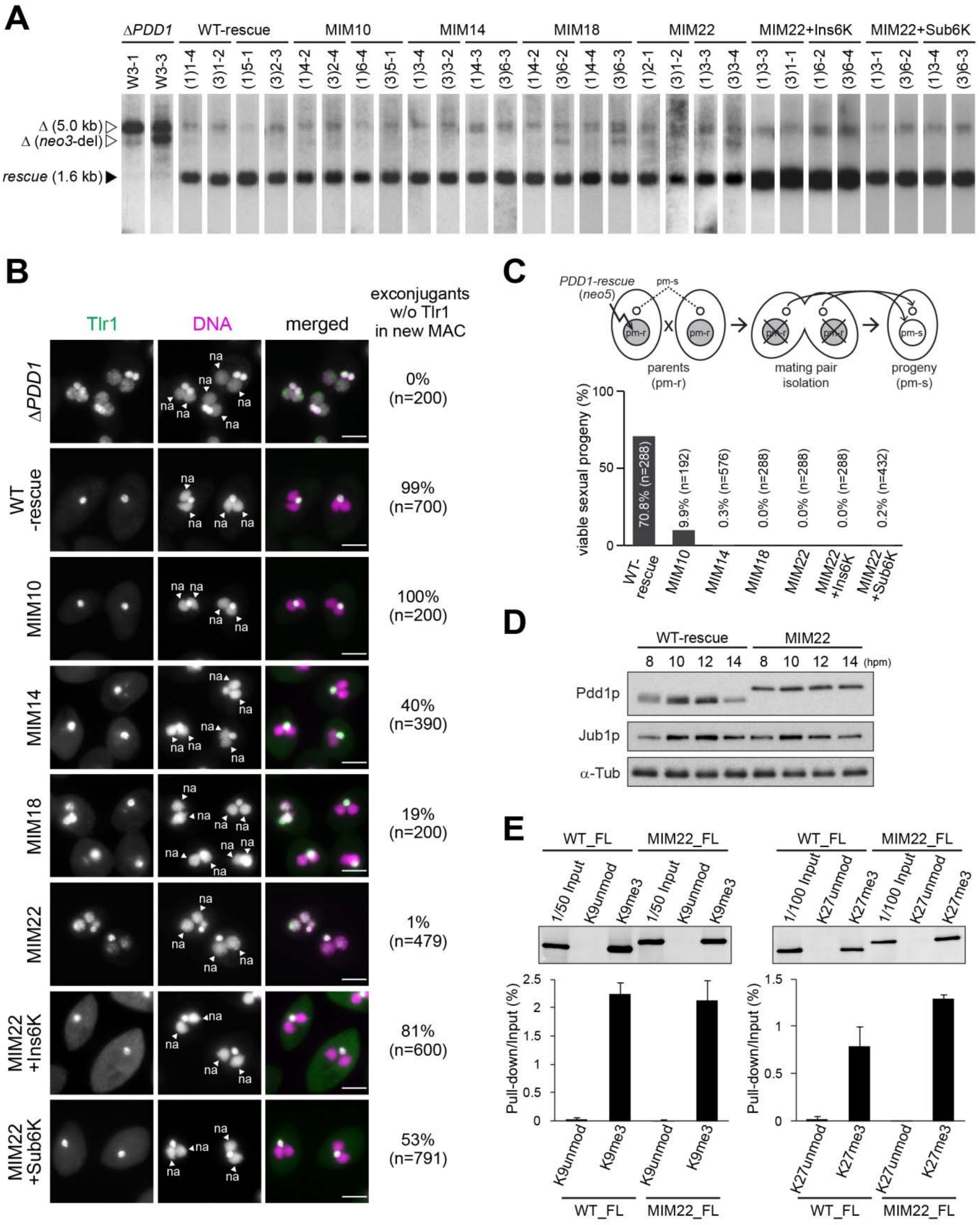
A
ΔPDD1
WT-rescue
MIM10
MIM14
MIM18
MIM22
MIM22+Ins6K
MIM22+Sub6K
Δ (5.0 kb)
Δ (neo3-del)
rescue (1.6 kb)
B
Tlr1
DNA
merged
exconjugants w/o Tlr1 in new MAC
0% (n=200)
99% (n=700)
100% (n=200)
40% (n=390)
19% (n=200)
1% (n=479)
81% (n=600)
53% (n=791)
C
PDD1-rescue (neo5)
pm-s
pm-r
parents (pm-r)
mating pair isolation
progeny (pm-s)
viable sexual progeny (%)
70.8% (n=288)
9.9% (n=192)
0.3% (n=576)
0.0% (n=288)
0.0% (n=288)
0.0% (n=288)
0.2% (n=432)
D
WT-rescue
MIM22
8 10 12 14 8 10 12 14 (hpm)
Pdd1p
Jub1p
α-Tub
E
WT_FL
MIM22_FL
1/50 Input
K9unmod
K9me3
1/100 Input
K27unmod
K27me3
Pull-down/Input (%)

Figure S5, related to Figure 5. In vivo and in vitro analyses of phosphor-mimic mutants of Pdd1p
(A) Genomic DNA extracted from the rescued strains (two pairs in each rescue strain) was digested with BglII (indicated as "B" in Figure 5B), and the replacements were analyzed by southern blot using the probe depicted in Figure 5B. Background *PDD1* KO strains were also analyzed as a control. The loci from KO and *neo3*-deletion in the background strains are marked with open arrowheads with "Δ (5.0 kb)" and "Δ (*neo3*-del)" (Liu et al., 2005), respectively. The replaced loci (1.6 kb) are marked by filled arrowheads with "rescue". **(B)** Exconjugants at 36 hpm from *PDD1* KO and rescued cells as indicated were hybridized with Tlr1 probe (green). DNA was counter-stained with DAPI (magenta). Arrowheads indicate new MACs (na). Scale bar represents 10 µm. The average percentages of exconjugants that did not show Tlr1 signals in the new MAC from more than 2 independent mating pairs are given. **(C)** Viability tests for the sexual progeny of the rescued cells. (Top) A schematic representation of the experimental design of the assay. The rescued cells (parents), which are paromomycin resistant (pm-r), were induced to conjugate. The mating pairs were isolated, and the pm sensitivity of the cells grown from the isolated mating pair was assessed. The progeny are pm sensitive (pm-s) in the absence of cadmium ions because the parental MAC carrying the *neo5* cassette is destroyed during conjugation (See Supplementary Experimental procedures for details). (Bottom) The average percentages of isolated mating pairs that produced pm-s progeny from more than 2 independent crosses are shown. **(D)** Proteins from WT-rescue and MIM22 cells during late conjugation (8-14 hpm) stages were analyzed by western blot with an anti-Pdd1p antibody and an anti-Jub1p antibody. The western blot using an anti-α-Tubulin (α-Tub) antibody is also shown as a control. **(E)** Histone peptide pull-down assay. MBP-tagged full-length recombinant Pdd1p (WT_FL and MIM22_FL) was pull-downed with beads coupled with peptide corresponding to N-terminal tail of histone H3, that contains unmodified or methylated Lys at 9 (K9unmod or K9me3), or at 27 (K27unmod or K27me3), and was analyzed by western blot using anti-MBP antibody (top). The mean value (±standard deviation) of enrichment relative to Input (Pull-down/Input) form 3 independent experiments is shown (bottom).

Figure S6

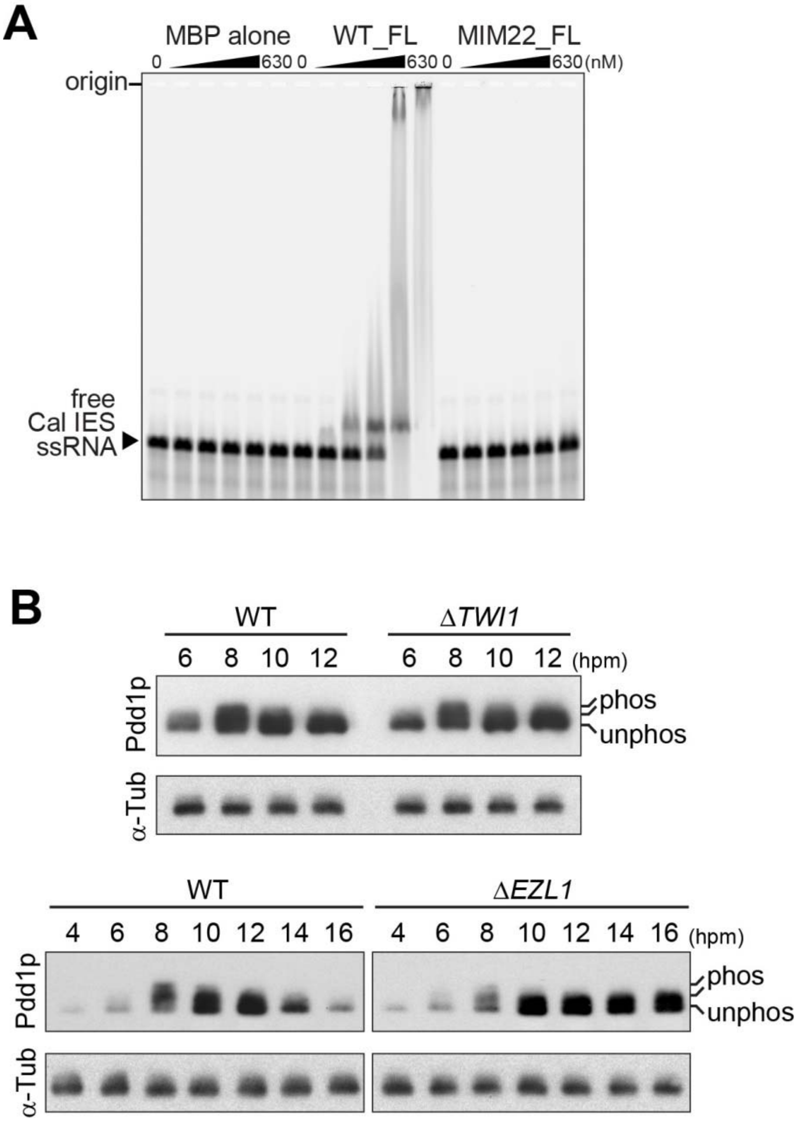

Figure 6S, related to Figure 7. EMSA with Cal IES ssRNA and western blot analyses of Pdd1p phosphorylation in *TWI1* and *EZL1* KO cells

(A) The 1,305-nt Cal IES ssRNA (10.4 nM) was titrated with a recombinant protein as indicated (0, 10.5, 52.5, 104, 420 and 630 nM) and analyzed by agarose gel electrophoresis. **(B)** Proteins from conjugating wild-type (WT), *TWI1* KO (Δ*TWI1*; 6-12 hpm) and *EZL1* KO (Δ*EZL1*; 4-16 hpm) cells were analyzed by western blot with an anti-Pdd1p antibody. Phosphorylated (phos) and unphosphorylated (unphos) Pdd1p are indicated. α-Tubulin (α-Tub) was analyzed as a control.

Supplementary Data S1, related to Figure 1:
1. Nuclear events during the life cycle of *Tetrahymena thermophila* (related to Figure 1)
2. Summary of the protein localization screen (related to Figure 1)
3. Localizations of EGFP-tagged proteins (related to Figure 1): Cells expressing EGFP-tagged proteins (green), which were growing, starved or conjugating with wild-type cells, were fixed and DNA was stained with DAPI (magenta). For conjugation stages (E1-1~L3), see "1. Nuclear events during the life cycle of *Tetrahymena thermophila*"

Supplemental Experimental Procedures

Strains and culture conditions

The wild-type *Tetrahymena thermophila* strains B2086, CU427, CU428 and SB1969 were provided by Dr. P. J. Bruns (Cornell University, USA). The *EZL1* KO strains, *PDD1-mCherry* strain and *TWI1* KO strains were described previously (Aronica et al., 2008; Kataoka et al., 2010; Noto et al., 2010). The germline *PDD1* KO strains (Motl and Chalker, 2011) were provided by Dr. Yifan Liu (University of Michigan, USA). Cells were grown in SPP medium (Gorovsky et al., 1975) containing 2% proteose peptone at 30ºC overnight. To induce conjugation, exponentially growing cells (~3-5x 10^5/ml) of two different mating types were starved in 10 mM Tris-HCl (pH 7.5) at 30°C for 8-14 h and mixed for conjugation (7x 10^5/ml) at 30°C.

Protein localization screen

The coding and 3' flanking sequences (~0.5-1 kb) of the target genes (listed in Supplementary Data S1) were amplified from the genomic DNA of the wild-type strain B2086 or CU428 by PCR using PrimeSTAR HS DNA polymerase (Takara) with the primers listed below. Targeting constructs were generated by connecting these genomic DNA fragments and an *EGFP-neo4* module, which was amplified from the plasmid pEGFP-neo4, by overlapping PCR using the primers 5'RACE Outer and 3'RACE Outer as previously described (Kataoka et al., 2010). The construct was introduced into the endogenous MAC locus of a wild-type strain (B2086 or CU428) by biolistic transformation as previously described (Cassidy-Hanley et al., 1997), and a partial replacement was performed by stepwise selection in increasing concentrations up to 25 mg/ml of paromomycin sulfate (pm, Sigma) in the presence of 1 µg/ml $CdCl_2$. These transgenic cells, which were in exponentially growing, starved and conjugating with a wild-type strain (B2086 or CU428) at 2, 4, 6, 8, 10, 12 and 14 hpm, were fixed with 10% formalin and 25% methanol in PBS at 4°C overnight. Localization of the EGFP-tagged proteins was observed in cells counter-stained with 10 ng/ml *4',6-Diamidino-2-phenylindole* (DAPI), and the conjugating cells were categorized by their nuclear morphology according to the developmental stages (see Supplementary Data S1). To compare the localizations of the EGFP-tagged proteins and Pdd1p, cells expressing an EGFP-tagged protein were mated with *PDD1*-mCherry cells.

Primary antibodies

To generate an anti-Jub1p antibody, rabbits were immunized with a peptide (CRSFDKQAQGKKNSS) corresponding to amino acids 838-851 (underlined) of Jub1p (DDBJ/EMBL/GenBank EAS04546.2). The antibody was affinity purified with the peptide, dialyzed against PBS and used for all the analyses of Jub1p performed in this study. To generate an anti-Pdd1p antibody, a guinea pig was immunized with a peptide (CLGSKGQSQQVEKEQATNS) corresponding to amino acids 315-332 (underlined) of Pdd1p (DDBJ/EMBL/GenBank XP_001016207.1). The antibody was purified with protein A, dialyzed against PBS and used for all immunofluorescent staining analyses of Pdd1p and the western blot analysis shown in Figure S5D. For the other western blot analyses, immuno-DNA-FISH and ChIP-Seq, a rabbit anti-Pdd1p antibody (ab5338, Abcam) was used. The mouse anti-dsDNA antibody HYB331-01 (ab27156, Abcam) and rabbit antibodies anti-H3K9me3 (07-442, Merck Millipore) and anti-H3K27me3 (07-449, Merck Millipore) were used for immunofluorescent staining. The rabbit antibody anti-H3K4me3 (ab8580, Abcam) was used for immuno-DNA-FISH. The mouse anti-alpha-tubulin antibody 12G10 was obtained from the Developmental Studies Hybridoma Bank (University of Iowa, USA) and used for western blot analyses.

Immunofluorescent staining

Cells were fixed and processed as previously described (Loidl and Scherthan, 2004) with modifications. Briefly, cells were fixed with 10% formalin in 10 mM Tris-HCl (pH 7.5) at room temperature for 10 min. Subsequently, Triton X-100 was added (final concentration of 0.25%), and the cells were incubated at room temperature for 20 min. The fixed cells were resuspended in 10% formalin containing 3.4% sucrose and air-dried on Superfrost Ultra Plus slides (Thermo Scientific). For immunofluorescent staining, the fixed cells were incubated with the mouse 1:500 anti-dsDNA antibody, rabbit antibodies (1:1000 anti-Jub1p, 1:500 anti-H3K9me3 or 1:1000 anti-H3K27me3) or 1:1000 guinea pig anti-Pdd1p antibody at 4°C overnight and then incubated with secondary antibodies (1:1000 anti-mouse, anti-rabbit or anti-guinea pig IgG conjugated with Alexa-488, -568 or -647

[Invitrogen]) at room temperature for 2 h. They were then counter-stained with 40 ng/ml DAPI and observed using an epifluorescence microscope and a confocal laser scanning microscope.

DNA-FISH and immuno-DNA-FISH

Cells were fixed and processed as described above, and DNA-FISH was performed as previously described (Loidl and Scherthan, 2004). Cy3-labeled probes for Tlr1 and REP2 IESs were produced as previously described (Noto et al., 2010). For immuno-DNA-FISH, the fixed cells were first hybridized with a Cy3-labeled Tlr1 probe. Subsequently, immunofluorescent staining was performed with a rabbit primary antibody and with an anti-rabbit IgG secondary antibody conjugated with Alexa-488 (Invitrogen) as described above.

Establishment of germ line *JUB1* KO strains

To create the knockout construct for *JUB1*, 5' and 3' flanking regions of the *JUB1* gene were amplified by PCR using the primer sets 00237610_KO5Fw/00237610_KO5Rv and 00237610_KO3Fw/3Am-00237610_Rv, respectively (primer sequences are listed below). The pm-resistant cassette *neo4* was isolated from the pNeo4 plasmid (Mochizuki, 2008) by SmaI digestion. The PCR products and the *neo4* cassette were connected by overlapping PCR using the PCR Extender System (5 PRIME) with the primers 00237610_KO5Fw and 3Am-00237610_Rv. The resulting targeting construct was introduced into the MIC of mating cells (B2086 and CU428) by biolistic transformation as described previously (Cassidy-Hanley et al., 1997), and a heterozygous progeny was selected with pm and 6-methylpurine in the presence of 1 μg/ml $CdCl_2$. To obtain heterozygous strains expressing different mating types, the heterozygous strain was mated with the wild-type strain CU427 or SB1969, and the heterozygous sexual progeny were selected with 0.1 mg/ml pm and 15 μg/ml cycloheximide in the presence of 1 μg/ml $CdCl_2$. The resulting heterozygous strains were cultured for 10 passages without pm, and pm-sensitive (pm-s) heterozygous strains were isolated. The pm-s heterozygous cells expressing different mating types were mated, and the sexual progeny were selected with 0.1 mg/ml pm in the presence of 1 μg/ml $CdCl_2$. The *JUB1* locus of the sexual progeny was amplified by PCR with the primers 00237610_Fw9 and 3Am-00237610_Rv, and complete homozygous KO strains were selected. The losses of the *JUB1* mRNA and Jub1p in the KO strains were confirmed by northern blot using a radiolabeled probe, which was generated from DNA amplified from cDNA by PCR with the primers 00237610_Fw1 and 00237610_Rv4 and by western blot analysis using the anti-Jub1p antibody.

ChIP-Seq

Nuclei from cells at 12 hpm were dissociated and stained with DAPI in TMSN buffer (0.25 M sucrose, 10 mM Tris-HCl pH 7.5, 10 mM $MgCl_2$, 3 mM $CaCl_2$, 0.016% NP-40, 1 mM PMSF, 1x complete proteinase inhibitor cocktail (Roche), 0.1 μg/ml DAPI) using a dounce homogenizer with 15 strokes. The nuclear fraction was collected by centrifugation at 4,500 g at 4°C for 5 min and then washed with TMSN buffer once and with TMSN(-) buffer (TMSN buffer without complete proteinase inhibitor cocktail) twice. The nuclei were fixed with 1.25 mg/ml Di(N-succinimidyl) glutarate (DSG) (Sigma) in PBSN buffer (PBS with 0.016% NP-40) at room temperature for 30 min, collected by centrifugation at 9,000 g at 4°C for 5 min, and washed with PBSN buffer twice. The DSG-fixed nuclei were re-fixed with 1% paraformaldehyde (Sigma) in PBSN buffer at room temperature for 10 min. The paraformaldehyde was quenched with 0.25 M glycine at room temperature for 5 min. The nuclei were washed with PBSN buffer containing 0.1 μg/ml DAPI twice and then with PBSN(+) buffer (PBSN with 1x complete proteinase inhibitor cocktail and 0.1 μg/ml DAPI) once, and the nuclear pellet was stored at -80°C. The nuclei were resuspended in PBSN(+), and the new MACs were collected according to their size and DAPI intensity using a FACSAria III (BD Biosciences). The purity of the collected new MACs was assessed by immuno-DNA-FISH using a Cy3-labeled probe against Tlr1 and an anti-Pdd1p antibody (Abcam) for wild-type, *JUB1* KO, WT-rescue and MIM22 cells or an anti-H3K4me3 (Abcam) antibody for *PDD1* KO cells: Tlr1-negative nuclei were counted as parental (old) MACs; Tlr1-positive, and Pdd1p- or H3K4me3-positive nuclei were counted as new MACs; Tlr1-positive but Pdd1p- or H3K4me3-negative nuclei were counted as MICs. Contamination of the parental MACs was rare (<2%), and most of the contaminants in our new MAC preparations were MICs. Chromatin immunoprecipitation was performed with the new MACs only when their purity was above 80%. Chromatin from the new MACs was sheared into 150-500 bp fragments (peak ~300 bp) in Sonication buffer (50 mM Tris-HCl pH 8.0, 10 mM EDTA, 0.1% SDS, 1x complete protease inhibitor cocktail, 1 mM PMSF) using a probe sonicator. For ChIP, 20 mg of Protein A Sepharose CL-4B beads (GE Healthcare) was washed with TE twice and with ChIP buffer (25 mM Tris-HCl pH 8.0, 167 mM NaCl, 5 mM EDTA, 0.05% SDS, 1% Triton X-100, 1x complete protease inhibitor cocktail, 1 mM PMSF) 3 times and was then pre-incubated with 90 μl of ChIP buffer containing 10 μl of 10 mg/ml BSA (NEB) (total ~200 μl suspension) at 4°C for 1 h. The fragmented chromatin from 2 million new MACs in Sonication buffer was diluted with an equal volume of 2x ChIP dilution buffer (334 mM NaCl, 2% Triton X-100, 1x complete protease inhibitor cocktail), adjusted to 1 ml with ChIP buffer and incubated with a 25 μl suspension of the beads at 4°C for 2 h for the pre-absorption. To couple an antibody to the beads, the 10 μl suspension of the beads pre-incubated with BSA was incubated with an antibody (50 μl anti-Jub1p or 5 μg anti-Pdd1p) in 0.5 ml of ChIP buffer at 4°C for 2 h and was then washed with ChIP buffer 3 times. The pre-absorbed chromatin was incubated with the antibody-coupled beads at 4°C overnight. The beads were washed once with Low-salt wash buffer (20 mM Tris-HCl pH 8.0, 150 mM NaCl, 2 mM EDTA, 0.1% SDS, 1% Triton X-100) and

twice each with High-salt buffer (20 mM Tris-HCl pH 8.0, 500 mM NaCl, 2 mM EDTA, 0.1% SDS, 1% Triton X-100), LiCl wash buffer (10 mM Tris-HCl pH 8.0, 0.25 M LiCl, 1 mM EDTA, 1% sodium deoxycholate, 1% NP-40) and TE. The immunoprecipitated chromatin was eluted with Elution buffer (0.1 M NaHCO3, 1% SDS). For cross-linking reversal, the NaCl concentration of the sample was adjusted to 200 mM, and the sample was incubated at 65°C for ~12 h. The DNA was extracted, and DNA libraries were generated from the immunoprecipitated DNA and 20 ng of the fragmented chromatin DNA without immunoprecipitation (Input) using the NEBNext DNA Library Prep Reagent Set for Illumina (NEB) and the KAPA Real-Time Library Amplification Kit (KAPA Biosystems). The libraries were sequenced using the HiSeq2000 platform (Illumina) with 50-nt single-end reads. The first 36 nucleotides of sequence from the reads were used in the downstream analyses. We obtained more than 18 million reads from the ChIP samples and more than 35 million reads from the Input samples that uniquely mapped to the draft MIC genome. The raw and processed sequence data sets have been deposited at the NCBI Gene Expression Omnibus (www.ncbi.nih.gov/geo/) as GSE70083. The numbers of normalized sequence reads (RPMs) from the ChIP sample that mapped to each 100-bp bin were divided by the numbers of reads from the Input sample, and these normalized scores were mapped to a representative 100-kb MIC locus, the LMR, as previously described, (Schoeberl et al., 2012) and to a modeled IES consisting of the 5,606 predicted IESs that range from 1-5 kb in length along with their franking sequences.

Genome-wide DNA elimination analysis
Nuclei from exconjugants at 36 hpm and from wild-type vegetative cells (B2086 and CU428) that were starved overnight at 30°C were stained with DAPI and dissociated as described in the ChIP-Seq procedures above. After the nuclear dissociation, the nuclear fraction was collected by centrifugation at 4,500 g at 4°C for 5 min, washed with TMSN(-) buffer without PMSF 3 times, and stored at -80˚C. The nuclear fraction was resuspended in TMSN buffer containing 2 mM EDTA without PMSF, and the MICs from vegetative cells and the new MACs from exconjugants were collected according to their size and DAPI intensity using a FACSAria III (BD Biosciences). The purities of the collected new MAC and MIC samples were assessed by immunofluorescent staining using an anti-H3K4me3 antibody and DAPI staining: H3K4me3-negative nuclei were counted as MICs; H3K4me3-positive, DAPI-poor small nuclei were counted as new MACs; and H3K4me3-positive, DAPI-rich large nuclei were counted as parental MACs. The purity of the MIC sample was >98%, and the rare contamination was from fragmented MACs. Contamination of the parental MACs in the isolated new MAC sample was rare (<1%), and most of the contaminants in our new MAC preparations were MICs. Genomic DNA libraries were constructed from the MIC samples and the new MAC samples only when the purity was above 90%. Genomic DNAs were extracted from the MICs and the new MACs and sonicated into ~250 bp fragments. Genomic DNA libraries were generated from 20 ng of the fragmented DNA using the NEBNext DNA Library Prep Reagent Set for Illumina (NEB) and the KAPA Real-Time Library Amplification Kit (KAPA Biosystems). The libraries were sequenced using the HiSeq2000 platform with 50-nt single-end reads. The first 36 nucleotides of the sequence of reads were used in the downstream analyses. We obtained more than 48 million reads that uniquely mapped to the draft MIC genome. The raw and processed sequence data sets have been deposited at the NCBI Gene Expression Omnibus (www.ncbi.nih.gov/geo/) as GSE70083. Of the 8,752 previously predicted IESs (Schoeberl et al., 2012), 8,074 are longer than 300 nt and contain more than 100-nt A/C/G/T bases that were used to map the sequence reads. The numbers of normalized sequence reads (RPMs) from the purified new MACs that mapped to each IES were calculated and divided by the average numbers of reads from the purified, wild-type-derived MICs (from B2086 and CU428) that mapped to the corresponding IES to give the Retention Indexes (RIs) of the IESs.

Viability test for sexual progeny
To introduce a drug resistance marker into the MAC of the *JUB1* KO strains to distinguish sexual progeny from parental cells, the 5' and 3' flanking regions of the non-essential *BTU1* gene were amplified from wild-type genomic DNA using the primer sets BTU1_5Fw/BTU1_5bsrRv and BTU1_3bsrFw/BTU1_3Rv, respectively (primer sequences are listed below). The Blasticidin S (bs)-resistance marker cassette *bsr*, which consists of the *HHF1* promoter followed by the bs resistance gene and the *BTU2* 3' UTR (Iwamoto et al., 2009), was amplified from the pBla1 plasmid (a gift from Dr. Masaaki Iwamoto and Dr. Tokuko Haraguchi, KARC, NICT, Japan) with T3 and T7 primers. These PCR products, the 5' and 3' flanking regions of *BTU1* and *bsr*, were connected by overlapping PCR with the primers BTU1_5Fw and BTU1_3Rv. The resulting targeting construct was introduced into the MAC of wild-type (B2086 and CU428) and *JUB1* KO strains by biolistic transformation as previously described (Cassidy-Hanley et al., 1997). The targeted loci were replaced into the transgene by stepwise selection with increasing concentrations of bs (0.3-10 mg/ml, Invivogen) in the absence of $CdCl_2$. The partial assortment was confirmed by genomic PCR using the primer set BTU1_5Fw/BTU1_3Rv. Conjugation of the transformants was induced, and 192 conjugating pairs were isolated in SPP medium at 8 hpm. The cells grown from the isolated pairs were examined for their resistance to 0.3 mg/ml bs in SPP medium, and the bs-sensitive cells were determined as sexual progeny. For the phosphor-mimic mutants, conjugating pairs were isolated from each mating cross at 8 hpm in SPP medium, and the cells that grew were examined for their resistance to 0.5 mg/ml pm in SPP medium without $CdCl_2$. The parental cells were pm-resistant under this condition (without cadmium ions), and the pm-sensitive cells were determined as sexual progeny.

Dephosphorylation of Pdd1p for western blots

Wild-type cells (B2086 and CU428) at 10 hpm and *JUB1* KO cells at 16 hpm were lysed by sonication in 20 mM Tris-HCl pH 7.5, 100 mM NaCl, 2 mM $MgCl_2$, 2 mM $CaCl_2$, 0.1% Tween-20, 0.05 mM PMSF and 1x complete protease inhibitor cocktail (Roche). Pre-cleared lysate was incubated with or without 0.15 U/µl FastAP (Thermo Scientific) at 37°C for 20 min. An equal volume of 2x SDS sample buffer was added to the lysate, which was then incubated at 95°C for 10 min and analyzed by western blotting with a rabbit anti-Pdd1p antibody (Abcam).

Phosphatase inhibitor treatment

Okadaic acid (495604, Merck Millipore) was dissolved in DMSO (1 mM). Conjugating wild-type cells (B2086 and CU428) were incubated from 7.5 hpm with 10 µM Okadaic acid in 10 mM Tris-HCl (pH 7.5) at 30°C. As a control, the cells were treated with the same amount of DMSO without Okadaic acid.

Identification of Pdd1p phosphorylation sites

Conjugating wild-type cells (B2086 and CU428) (5.6x 10^7) at 8 hpm were lysed by sonication in 20 mM Tris-HCl pH 7.5, 100 mM NaCl, 2 mM $MgCl_2$, 2 mM $CaCl_2$, 0.1% Tween-20, 0.05 mM PMSF, 1.25x complete protease inhibitor cocktail (Roche) and 2x PhosSTOP phosphatase inhibitor cocktail (Roche), and the lysate was cleared by centrifugation at 20,000 g at 4°C for 30 min. To immunoprecipitate Pdd1p, a 100 µl suspension of Affi-Prep Protein A Support beads (Bio-Rad) were incubated with 28.5 µg of anti-Pdd1p antibody (Abcam) at 4°C overnight in PBST (PBS containing 0.04% Triton X-100) and washed 3 times each with PBST and with 0.2 M sodium borate pH 9.2. The antibody was cross-linked to the beads by incubating with 20 mM DMP (dimethyl pimelimidate dihydrochloride) in 0.2 M sodium borate pH 9.2 at room temperature for 30 min, and the beads were washed twice each with 0.2 M Tris-HCl pH 8.0 and with PBST. To remove uncross-linked antibody, the beads were washed twice with 0.1 M glycine pH 2.0 followed by washing with PBST three times. The beads were incubated with the pre-cleaned lysate at 4°C for 4.5 h and washed eight times with Washing buffer (20 mM Tris-HCl pH 7.5, 500 mM NaCl, 2 mM $MgCl_2$, 2 mM $CaCl_2$, 1% Triton X-100), three times with Washing buffer without Triton X-100 and once with 150 mM NaCl. The immunopurified Pdd1p was eluted with 0.1 M glycine pH 2. After adjusting the pH to 8, the immunoprecipitated Pdd1p was digested with trypsin, chymotrypsin or subtilisin. The digested peptides were analyzed by an LTQ Orbitrap XL mass spectrometer (Thermo Scientific) coupled with the UltiMate 3000 HPLC system (Dionex). The resulting molecular masses were searched against the Pdd1p sequence using Mascot (Matrix Science) with the additional mass of phosphorylation. The tandem mass spectra showing Mascot scores >10 and those showing Mascot scores from 5-9 and probability >90% as analyzed by phosphoRS (Taus et al., 2011) were selected for phosphorylation site assignment.

Phosphor-mimic *PDD1* mutant strains

To create a plasmid backbone for the rescue construct, the 5' and 3' franking regions of the *PDD1* gene were amplified from CU428 genomic DNA by PCR with the primer sets BX-5FNK_PDD1_FwL/SSB-5FNK_PDD1_Rv and SSS-3FNK_PDD1_Fw/KX-3FNK_PDD1_RvL, respectively (primer sequences are listed below). These two PCR products were connected by overlapping PCR using the primers BX-5FNK_PDD1_FwL and KX-3FNK_PDD1_RvL, digested with BlnI and KpnI and inserted into the SpeI and KpnI sites of pBluescript SK(+). The pm resistance cassette *neo5* franked by loxP sequences (Busch et al., 2010) was inserted into the plasmid at the SalI and SmaI sites followed by the insertion of the *PDD1* 3' UTR, which was amplified from CU428 genomic DNA by PCR using the primer set SpeI-PDD1_3UTR_Fw/SalI-PDD1_3UTR_Rv, at the SpeI and SalI sites. The resulting plasmid backbone was named pPPLNP1 (see below for the sequence). *PDD1* coding sequences were amplified from CU428 genomic DNA for the *WT-rescue* construct or amplified from synthesized DNA for the phosphor-mimic mutants *MIM10*, *14*, *18* and *22* (Genscript) and for the positive charge-added mutants *MIM22+Ins6K* and *MIM22+Sub6K* (Genscript) by PCR with the primer set BamHI-PDD1ORF_Fw/SpeI-PDD1ORF_Rv2. The sequences of *MIM10*, *MIM14*, *MIM18*, *MIM22*, *MIM22+Ins6K* and *MIM22+Sub6K* are listed below. The PCR products were inserted into the pPPLNP plasmid at the BamHI and SpeI sites. The resulting plasmids containing the targeting constructs were linearized by XhoI and transformed into the MACs of *PDD1* KO strains by biolistic transformation as previously described (Cassidy-Hanley et al., 1997). *PDD1* KO loci in the MAC were replaced almost completely by the transgene through stepwise selection with increasing concentrations of pm (0.5-60 mg/ml) in the absence of $CdCl_2$. The replacement was analyzed by southern blot in which genomic DNA from isolated transformants was digested with BglII and hybridized to a radiolabeled probe that was produced from wild-type genomic DNA amplified by PCR with the primers BX-5FNK_PDD1_FwL and SSB-5FNK_PDD1_Rv. The expression of *PDD1* mRNA in wild-type and WT-rescue cells was analyzed by northern blot using a radio-labeled probe that was generated from wild-type genomic DNA amplified by PCR with the primers 5Am-Pdd1_Fw and SpeI-PDD1ORF_Rv2.

Recombinant proteins

Full-length wild-type *PDD1*, *MIM14*, *MIM22* and *MIM22+Ins6K* genes that were codon-optimized for *E. coli* expression and named *opt.WT_FL*, *opt.MIM14_FL*, *opt.MIM22_FL* and *opt.MIM22+Ins6K_FL,* respectively,

were synthesized (Genscript and IDT). The sequences are listed below. Amplified DNA corresponding to the *PDD1* mutants *opt.W50A/W53A_FL* and *opt.I456D_FL* were produced from *opt.WT_FL* by PCR. For *opt.I456D_FL*, the primer set EcoRI-opt.PDD1_Fw/PstI-TGA-opt.PDD1_I478D_RvMAL was used (primer sequences are listed below). For *opt.W50A/W53A_FL*, two DNA fragments were first amplified from *opt.WT-FL* with the primer sets EcoRI-opt.PDD1_Fw/opt.PDD1_W72,75A_Rv1 and opt.PDD1_W72,75A_Fw1/PstI-TGA-opt.PDD1_Rv2. These fragments were connected by overlapping PCR with the primer set EcoRI-opt.PDD1_Fw/PstI-TGA-opt.PDD1_Rv2. Amplified DNA corresponding to the hinge regions of wild-type Pdd1p, *opt.WT_HNG1*: 78-188 aa and *opt.WT_HNG2*: 226-397 aa, were produced from *opt.WT_FL* by PCR with the primer sets ERI-opt.PDD1_HNG1_Fw/PstI-TGA-opt.PDD1_HING1_Rv and ERI-opt.PDD1_HNG2_Fw/PstI-TGA-opt.PDD1_HING2_Rv. For the MBP-fusion proteins, *opt.WT_FL*, *opt.WT_HNG1*, *opt.WT_HNG2*, *opt.MIM14_FL*, *opt.MIM22_FL*, *opt.MIM22+Ins6K_FL*, *opt.W50A/W53A_FL* and *opt.I456D_FL* were cloned into the EcoRI and PstI sites of pMAL-c2X (NEB). To express MBP alone, a stop codon-containing sequence (5'-TGAGCATAACGTGCCTTGC-3') was inserted into the EcoRI and PstI sites of pMAL-c2X. For the GST-fusion proteins, *opt.WT_FL* and *opt.MIM22_FL* were amplified by PCR with the primer sets EcoRI-opt.PDD1_FwpGEX/XhoI-opt.PDD1_RvpGEX and ERI-opt.MIM22_FwGEX/XhoI-opt.MIM22_RvGEX, respectively, and cloned into the EcoRI and XhoI sites of a variant of pGEX-4T-1 containing a TEV cleavable site between an N-terminal GST tag and the protein coding sequence (pGEX-4T-1-TEV, a gift from Dr. Tim Clausen, IMP, Austria). For GST alone, empty pGEX-4T-1-TEV was used. All recombinant proteins were expressed in the *E. coli* strain BL21 (DE3). Bacterial cultures were grown at 37°C in LB with ampicillin to an OD600 of ~0.8. The cultures were cooled on ice for 20 min, and protein expression was induced with 0.5 mM IPTG at 18°C overnight. The recombinant proteins were purified as described previously (Woehrer et al., 2015), dialyzed against Interaction buffer (20 mM HEPES-NaOH pH 7.5, 100 mM KCl, 1 mM EDTA, 0.1 mM DTT, 10% glycerol) at 4°C overnight, and stored at -80°C. The protein concentrations were estimated by SDS-PAGE followed by PageBlue staining (Thermo Scientific) using BSA (NEB) as a reference.

Histone peptide pull-down assay

Biotinylated Peptides corresponding to the N-terminal tail of *Tetrahymena* histone H3 (K9unmod: ARTKQTARKSTGAKAPRKQ, K9me3: ARTKQTAR[Kme3]STGAKAPRKQ, K27unmod: PRKQLASKAARKSAPATGG and K27me3: PRKQLASKAAR[Kme3]SAPATGG) were synthesized as described (Vogt and Mochizuki, 2013). Peptide (4 nmol) was incubated with 20 µl (bed volume) of Dynabeads M-280 Streptavidin (Invitrogen) in PBS at room temperature for 3 h and excess peptide was washed-out with PBS containing 0.1% Tween-20. The peptide-coupled beads were pre-incubated with Interaction buffer (20 mM HEPES-NaOH pH 7.5, 1 mM EDTA, 0.1 mM DTT, 10% glycerol, and KCl at 400 mM for K27 peptides or 500 mM for K9 peptides) containing 5% BSA at 4°C for 1 h. Recombinantly expressed MBP-WT_FL or MBP-MIM22_FL (30 pmol) was incubated with the beads in 300 µl Interaction buffer (with corresponding KCl concentration) containing 2.5% BSA at 4°C overnight. The beads were washed with Washing buffer (20 mM HEPES-NaOH pH 7.5, 1 mM EDTA, 0.1% Tween-20, and KCl at 400 mM for K27 peptides or 500 mM for K9 peptides) at room temperature 6 times for 10 min each. The bound protein was eluted with 1x SDS-PAGE sample buffer by incubation at 95°C for 10 min, resolved in 8% SDS-PAGE gel and analyzed by western blot using anti-MBP primary antibody (NEB) and anti-rabbit IgG secondary antibody conjugated with IRDye 800CW (LI-COR). The images were quantified by Odyssey CLx (LI-COR). The mean value of enrichment relative to corresponding input and the standard deviation of the mean were calculated from 3 independent experiments.

GST pull-down assays

GST and MBP fusion proteins (350 pmol each) were mixed, and the volume was adjusted to 400 µl with Interaction buffer (20 mM HEPES-NaOH pH 7.5, 100 mM KCl, 1 mM EDTA, 0.1 mM DTT, 10% glycerol). GST pull-down buffer (933.3 µl) (20 mM Tris pH 7.5, 100 mM NaCl, 0.1 mM EDTA, 0.1% Triton X-100) was added, and the proteins were incubated at 4°C for 1 h. The protein complex containing the GST-tag was affinity purified by incubation with a 20 µl suspension of Glutathione Sepharose 4B beads (GE-Healthcare) at 4°C for 1 h followed by four washes with GST pull-down buffer at 4°C. The protein was eluted from the beads by incubation with 25 µl of 1x SDS PAGE buffer at 95°C for 10 min ("GST pull-down" sample). Then, 17.5 pmol (1/20) of the input samples and 10 µl of the GST pull-down samples were analyzed by SDS-PAGE followed by PageBlue staining (Thermo Scientific).

EMSA

To produce EGFP ssRNA (723 nt) and Cal IES ssRNA (1305 nt) as substrates for EMSA, DNA templates containing the T7 promoter were generated from the plasmids pEGFP-neo4 (Kataoka et al., 2010) and pCaM_MDS-IES, which contains the Cal IES sequence from the B2086 wild-type strain (see below for the sequence), by PCR using PrimeSTAR HS DNA polymerase and the primer sets T7-opt.EGFP_Fw1/opt.EGFP_Rv1 and T7-CaM-IES_Fw1/CaM-IES_Rv1, respectively (primer sequences are listed below). The PCR products were purified with the QIAquick PCR Purification Kit (Qiagen) and Illustra MicroSpin S-400 HR Columns (GE Healthcare). In vitro transcription was performed on the PCR products using the

MEGAscript T7 transcription kit (Ambion) according to the manufacturer's instructions. To label the RNA, 1.9 mM fluorescein-12 UTP (Roche) and 5.6 mM non-labeled UTP were used in the reaction. The reaction (20 µl) was carried out at 37°C for 3 h followed by incubation with 1 µl of TURBO DNase (Ambion) at 37°C for 15 min. The labeled RNAs were purified by phenol-chloroform-isoamyl alcohol extraction followed by isopropanol precipitation. The RNAs were dissolved in nuclease-free water, and the unincorporated nucleotides were removed using mini Quick Spin RNA columns (Roche). EMSAs were performed as described previously (Keller et al., 2012) with modifications. Two microliters containing the desired amount (0.125-7.5 pmol) of protein dialyzed against Interaction buffer was incubated with 8 µl of EMSA buffer (20 mM HEPES-NaOH pH7.5, 100 mM KCl, 0.05% NP-40) at room temperature for 10 min. Two microliters of fluorescein-labeled *EGFP* ssRNA or Cal IES ssRNA (125 fmol) was added as a substrate and the mixture was incubated at room temperature for 30 min, and the samples were analyzed by 1x TBE agarose (0.5% for the FL and 1% for the HNG1 and HNG2 proteins) gel electrophoresis. Fluorescently labeled RNA was detected using a Typhoon Trio imaging system (GE Healthcare) and quantified with ImageQuant TL (GE Healthcare). The mean dissociation constant (Kd) and the standard deviation of the mean were calculated from more than 2 independent experiments.

Primers used for C-terminal EGFP tagging constructs

a) Primers to amplify coding and 3' flanking regions

TTHERM_#	amplicon	name	sequence (5' -> 3')
TTHERM_00006160	coding	5Am-00006160_Fw	GCTGATGGCGATGAATGAACACTGGCTATCAAATATCGATAATATTGATGTTTC
		00006160-EGFP_Rv	AAGTTCTTCACCCTTAGAAACCATGGATCCGTTAAATATATCGTTGACAGTAAGGTTAGC
	3' flanking	Neo4-00006160_Fw	CCCGGGGGATCTGAATTCGATATCAAGCTTGAAGAAGTTACTCAAGTATGTATGAACTTG
		3Am-00006160_Rv	GCGAGCACAGAATTAATACGACTAAATAAAACTTTAATATTGTACTAAGTTAC
TTHERM_00008690	coding	5Am-00008690_Fw	GCTGATGGCGATGAATGAACACTGATGAATAACATAGAAGAAAGTGCTTTCTAC
		00008690-EGFP_Rv	AAGTTCTTCACCCTTAGAAACCATGGATCCGTTTTTTTAATTCTTTTTACTTGATTATTAAATG
	3' flanking	Neo4-00008690_Fw	CCCGGGGGATCTGAATTCGATATCAAGCTTATATAATATACCACATTTTATACTTAGTCC
		3Am-00008690_Rv	GCGAGCACAGAATTAATACGACTTCTAAGATCATCATTTGCTGCCTTTCCT
TTHERM_00013110	coding	5Am-00013110_Fw	GCTGATGGCGATGAATGAACACTGATGTTGATAGTGATGGTATGTATATTGTCA
		00013110-EGFP_Rv	AAGTTCTTCACCCTTAGAAACCATGGATCCAAGTTCTTGAAGTACCTGTTAATTAATTAAT
	3' flanking	Neo4-00013110_Fw	CCCGGGGGATCTGAATTCGATATCAAGCTTCATAACCACAAACAAACACATAAAAAGCCA
		3Am-00013110_Rv	GCGAGCACAGAATTAATACGACTACAATTAAAGTTTGGACATCAATCAAAGCT
TTHERM_000245499	coding	5Am-00245500_Fw	GCTGATGGCGATGAATGAACACTGCAAGCTATGTATTCCTTAGCGTGCCAGCAC
		00245500-EGFP_Rv	AAGTTCTTCACCCTTAGAAACCATGGATCCATCTTAAATTCCTCTTTAAAATTATTTAAA
	3' flanking	Neo4-00245500_Fw	CCCGGGGGATCTGAATTCGATATCAAGCTTCTAAAATAGCTTTAAAGTACACAGTAGAATC
		3Am-00245500_Rv	GCGAGCACAGAATTAATACGACTTTCTAGAATTATTAATTCAAAATGTATGTG
TTHERM_000279929	coding	5Am-00279930_Fw	GCTGATGGCGATGAATGAACACTGGTAATGAGATGAGTTTAGATGAAGCATCAG
		00279930-EGFP_Rv	AAGTTCTTCACCCTTAGAAACCATGGATCCTTTTTTTATTTTTTTGGTATACTTTTATTTATATTC
	3' flanking	Neo4-00279930_Fw	CCCGGGGGATCTGAATTCGATATCAAGCTTTAAATCAAATGTTTGTTTCAACTACAATTC
		3Am-00279930_Rv	GCGAGCACAGAATTAATACGACTTATGTCTTATATTCTCACTAAATTATCATC
TTHERM_00028580	coding	5Am-00028580_Fw	GCTGATGGCGATGAATGAACACTGAATAATGTAGGACTTATCCTTGACATCTCT
		00028580-EGFP_Rv	AAGTTCTTCACCCTTAGAAACCATGGATCCATTATTAGCATAATAAGAAGGAGAGTTTATG
	3' flanking	Neo4-00028580_Fw	CCCGGGGGATCTGAATTCGATATCAAGCTTGATAAAATCTAAATATTCAGCCACCATAATG
		3Am-00028580_Rv	GCGAGCACAGAATTAATACGACTATCCTCTCTATCTTATTAGATAAAGAAGAG
TTHERM_000295779	coding	5Am-00295780_Fw	GCTGATGGCGATGAATGAACACTGGTAATAGGGAAATCAATATTTGCTTTAAAG
		00295780-EGFP_Rv	AAGTTCTTCACCCTTAGAAACCATGGATCCGCAATAAGACTTTTTTTTTGAAAATTTATTG
	3' flanking	Neo4-00295780_Fw	CCCGGGGGATCTGAATTCGATATCAAGCTTTATAGATAGATATATATGTGCATACTTTAC
		3Am-00295780_Rv	GCGAGCACAGAATTAATACGACTTTCTATATTCCAACTCTCTTTGGACATATC
TTHERM_00034970	coding	5Am-00034970_Fw	GCTGATGGCGATGAATGAACACTGTACATGAACTTAAAGAATGCACTTGACGAC
		00034970-EGFP_Rv	AAGTTCTTCACCCTTAGAAACCATGGATCCCTTTTCTTTTTAGTTATTGAGTGATTTTTG
	3' flanking	Neo4-00034970_Fw	CCCGGGGGATCTGAATTCGATATCAAGCTTTGCTTATTGGATAAAATATTAATCCATAGC
		3Am-00034970_Rv	GCGAGCACAGAATTAATACGACTATAATTAGCTTGTCCAATTTCTTTACAAAC
TTHERM_00036890	coding	5Am-00036890_Fw	GCTGATGGCGATGAATGAACACTGCTCGAACTCTAATCAGATTCATTTAGTGTG
		00036890-EGFP_Rv	AAGTTCTTCACCCTTAGAAACCATGGATCCTTCTAATTCTATTTAATTAATCTAATTTTC
	3' flanking	Neo4-00036890_Fw	CCCGGGGGATCTGAATTCGATATCAAGCTTCCACTTTACAAACATTCATATTTAGCTTTC
		3Am-00036890_Rv	GCGAGCACAGAATTAATACGACTTCAAAACTTATAAAATCCAGCTGAAATATC
TTHERM_00046930	coding	5'Am-00046930_Fw	GCTGATGGCGATGAATGAACACTGACAGACAACAGTTGTGCTGAAAAAGCTGTTT
		00046930-EGFP_Rv2	AAGTTCTTCACCCTTAGAAACCATGGATCCTAAATTTTAGGAATAATTCTAATTCAAAGA
	3' flanking	Neo4-00046930_3'FLNK_Fw3	CCCGGGGGATCTGAATTCGATATCAAGCTTTGGTTATATCATTGCATTTTATCTTTCTAA
		3'Am-00046930_Rv3	GCGAGCACAGAATTAATACGACTTGATATGGGAGTGGGCACCTATTCTTTCGT
TTHERM_00049220	coding	5Am-00049220_Fw	GCTGATGGCGATGAATGAACACTGTGAAATATCAGTGAGGAGAATGAGCAACAC
		00049220-EGFP_Rv	AAGTTCTTCACCCTTAGAAACCATGGATCCTTTGCTTAATTTTGGATTGTATTAGTCAAT
	3' flanking	Neo4-00049220_Fw	CCCGGGGGATCTGAATTCGATATCAAGCTTTTAAAATATATAGAATGGATTGATTACGAG
		3Am-00049220_Rv	GCGAGCACAGAATTAATACGACTTACAAGTCTCCAACAACTCACTATGGCTGA
TTHERM_000549648	coding	5Am-00549670_Fw	GCTGATGGCGATGAATGAACACTGAGCATTTGTTGTTGTTGCTGCTGCTAGTTC
		00549670-EGFP_Rv	AAGTTCTTCACCCTTAGAAACCATGGATCCATTAATTAATTATTAAATATACTGG
	3' flanking	Neo4-00549670_Fw	CCCGGGGGATCTGAATTCGATATCAAGCTTTTCAGTTTAATTTACATTCTTTTTAAGTTG
		3Am-00549670_Rv	GCGAGCACAGAATTAATACGACTTTTACAAGTTATGGAAACAAATAGATGCTC
TTHERM_00079530	coding	5'Am-00079530_Fw	GCTGATGGCGATGAATGAACACTGCACTTCTGTCGTGAAATTGCTCATCAAGCT
		00079530-EGFP_Rv	AAGTTCTTCACCCTTAGAAACCATGGATCCATTCAAGATAAACTTTTAGAATTTATCTCT
	3' flanking	Neo4-00079530_3'FLNK_Fw	CCCGGGGGATCTGAATTCGATATCAAGCTTCTATATAAATTGTGATTGACTTTTAATTAG
		3'Am-00079530_Rv	GCGAGCACAGAATTAATACGACTCATCCATTCCTATAAAGCTGATGAAGTGCT
TTHERM_00086720	coding	5'Am-00086720_Fw	GCTGATGGCGATGAATGAACACTGGTCAACTTCAATTTAGAGATCCAGTTCTGG
		00086720-EGFP_Rv	AAGTTCTTCACCCTTAGAAACCATGGATCCGATATTTATTCTATTTTCTTTCCCGTTTACC
	3' flanking	Neo4-00086720_3'FLNK_Fw	CCCGGGGGATCTGAATTCGATATCAAGCTTCTCTTCATTCTATATATAAATAAAATCAACT
		3'Am-00086720_Rv	GCGAGCACAGAATTAATACGACTAAAAAATCTATTAGAATATACTATCAGCCT
TTHERM_00091510	coding	5Am-00091510_Fw	GCTGATGGCGATGAATGAACACTGGTAAAAGTACTATGACGACTTGCTACCTGT
		00091510-EGFP_Rv	AAGTTCTTCACCCTTAGAAACCATGGATCCTTATTTAGGAGCCATAATGTTTAAGAGC
	3' flanking	Neo4-00091510_Fw	CCCGGGGGATCTGAATTCGATATCAAGCTTTGTTAATTAAATTTAGTAGGTTTTAACTGT
		3Am-00091510_Rv	GCGAGCACAGAATTAATACGACTGTGTATTTAGCTATAAGTATTAGCGTTGA
TTHERM_00092800	coding	5Am-00092800_Fw	GCTGATGGCGATGAATGAACACTGAACAACTGGTACCAACAGGGAAAGTGTATC
		00092800-EGFP_Rv	AAGTTCTTCACCCTTAGAAACCATGGATCCAAGTATTTGGCTATTTAGTTTACTC
	3' flanking	Neo4-00092800_Fw	CCCGGGGGATCTGAATTCGATATCAAGCTTTTCAAATACTACTACTATTACAAATACTAG
		3Am-00092800_Rv	GCGAGCACAGAATTAATACGACTAGTTTAGAAGTGTTTGTTTTAATTACTTAG
TTHERM_00112710	coding	5Am-00112710_Fw	GCTGATGGCGATGAATGAACACTGAGCAGTAGGAAGAAGATAAGGTGATTGGCT
		00112710-EGFP_Rv	AAGTTCTTCACCCTTAGAAACCATGGATCCATTTTATTCAATATTATTTACTTAGATTTTTTGAG
	3' flanking	Neo4-00112710_Fw	CCCGGGGGATCTGAATTCGATATCAAGCTTCAAATAGTTTTGTTCTAATAGAGACAATAG
		3Am-00112710_Rv	GCGAGCACAGAATTAATACGACTCTATAAATATTTTCATTCTATCAACACCTA
TTHERM_00112830	coding	5Am-00112830_Fw	GCTGATGGCGATGAATGAACACTGTGAAAATAACTCATTGTACTAATACTAGGA
		00112830-EGFP_Rv	AAGTTCTTCACCCTTAGAAACCATGGATCCAATTAAAATGACATAGTATTCATC
	3' flanking	Neo4-00112830_Fw	CCCGGGGGATCTGAATTCGATATCAAGCTTTTTTGAAAAATTATGAAATTATATAAAGGC
		3Am-00112830_Rv	GCGAGCACAGAATTAATACGACTGGAATAACATGGATAAAAGTATTACCATGG
TTHERM_00113310	coding	5Am-00113310_Fw	GCTGATGGCGATGAATGAACACTGCAGACTCAGAATACATGATTTAGGCTATTG
		00113310-EGFP_Rv	AAGTTCTTCACCCTTAGAAACCATGGATCCATTATTATCTATAAGTTCTTTGAATTTCTG

	3' flanking	Neo4-00113310_Fw	CCCGGGGGATCTGAATTCGATATCAAGCTTACGAATTAATTTCTCCTAGTTTTTTCTTAC
		3Am-00113310_Rv	GCGAGCACAGAATTAATACGACTATTTCATAAAAAATGTTAGCTGTAGTAATG
TTHERM_00128920	coding	5Am-00128920_Fw	GCTGATGGCGATGAATGAACACTGGGTAAGAAAAGCTAATAAGCTCAAGGTGAC
		00128920-EGFP_Rv	AAGTTCTTCACCCTTAGAAACCATGGATCCATCTTGAGTTTAACTATGTTCATGTTCAGA
	3' flanking	Neo4-00128920_Fw	CCCGGGGGATCTGAATTCGATATCAAGCTTTTCTTTAAAATCTAAAGAAAAGTATCTTGC
		3Am-00128920_Rv	GCGAGCACAGAATTAATACGACTCAAAACATTTAATAAATGTCTCAAGATGCC
TTHERM_00133710	coding	5Am-00133710_Fw	GCTGATGGCGATGAATGAACACTGGTTAACATACAAGACACTCCAATCTTTCTC
		00133710-EGFP_Rv	AAGTTCTTCACCCTTAGAAACCATGGATCCAAAAGCACTCTTTCTGAAGCTAAAACAACT
	3' flanking	Neo4-00133710_Fw	CCCGGGGGATCTGAATTCGATATCAAGCTTATAAAGAACTTACTTAATTATCAGGTTAAC
		3Am-00133710_Rv	GCGAGCACAGAATTAATACGACTGTTTAAAGTAACCTTCATCCATCATACCAA
TTHERM_00133730	coding	5Am-00133730_Fw2	GCTGATGGCGATGAATGAACACTGAACCTAAAGTAATCACAATTGTTTCTTCC
		00133730-EGFP_Rv2	AAGTTCTTCACCCTTAGAAACCATGGATCCATTATTTTAAGTTTAGGTTGCCTTTTTCC
	3' flanking	Neo4-00133730_Fw2	CCCGGGGGATCTGAATTCGATATCAAGCTTTTTTAGTTATATTTTTGTGGATTGTAC
		3Am-00133730_Rv2	GCGAGCACAGAATTAATACGACTCAAATAGAATATGATGATATCAAATTACTG
TTHERM_00155590	coding	5'Am-00155590_Fw	GCTGATGGCGATGAATGAACACTGGAAGAAGCTCTAAGTAAATGCATGCAGAAAC
		00155590-EGFP_Rv	AAGTTCTTCACCCTTAGAAACCATGGATCCTTGTTCAGCATTCATTTATTGTAAGCCATT
	3' flanking	Neo4-00155590_3'FLNK_Fw	CCCGGGGGATCTGAATTCGATATCAAGCTTACCTATCTATATTTTTCCACTAATTACCCA
		3'Am-00155590_Rv	GCGAGCACAGAATTAATACGACTGTATTCTAATAACTATCCTATCAGTCAGTC
TTHERM_00185640	coding	5Am-00185640_Fw2	GCTGATGGCGATGAATGAACACTGTTCACTCACTCAATCTTCGAGAGTTCTAAG
		00185640-EGFP_Rv	AAGTTCTTCACCCTTAGAAACCATGGATCCGTTTGAGCTATTAGTAATTTATTTTAAGTT
	3' flanking	Neo4-00185640_Fw	CCCGGGGGATCTGAATTCGATATCAAGCTTAAACGAACTTTTTACTTCTAACAAACCAAC
		3Am-00185640_Rv	GCGAGCACAGAATTAATACGACTACCTTTTGGAATTGAAAGCTCAAATTCTGC
TTHERM_00189440	coding	5'Am-00189440_Fw	GCTGATGGCGATGAATGAACACTGCTGCATCTCCATTTGATTATGTAGATAATC
		00189440-EGFP_Rv	AAGTTCTTCACCCTTAGAAACCATGGATCCAAATGATTATTTGCTATTATTTGCAGAATG
	3' flanking	Neo4-00189440_3'FLNK_Fw	CCCGGGGGATCTGAATTCGATATCAAGCTTTTGTATTTATTTGTGTGTATGTGTGTTTGA
		3'Am-00189440_Rv	GCGAGCACAGAATTAATACGACTAGTTATTTGAGGGTGGTTACCATCAAATGC
TTHERM_00193969	coding	5Am-00193970_Fw	GCTGATGGCGATGAATGAACACTGGTTTAACATTCCTTCGTTTCTACTAAACCT
		00193970-EGFP_Rv	AAGTTCTTCACCCTTAGAAACCATGGATCCAATTTCCTAATCTAAGTCTTTCACTTCTAAG
	3' flanking	Neo4-00193970_Fw	CCCGGGGGATCTGAATTCGATATCAAGCTTTGTTTGTTATCTTTTATAAAAGTATTCTTG
		3Am-00193970_Rv	GCGAGCACAGAATTAATACGACTAAGCCAATTTAAATTGTCCATAGTTGGTCT
TTHERM_00197670	coding	5Am-00197670-2_Fw	GCTGATGGCGATGAATGAACACTGAATTAACCAAGAAGACCAATCATCCTTGCT
		00197670-2-EGFP_Rv	AAGTTCTTCACCCTTAGAAACCATGGATCCTTTGTAAACCTTCTTTTCTTATTATTTAAG
	3' flanking	Neo4-00197670-2_Fw	CCCGGGGGATCTGAATTCGATATCAAGCTTGTTGGATTTTTGATTGAATTTTAGAAAATC
		3Am-00197670-2_Rv	GCGAGCACAGAATTAATACGACTAAAGTTAGTTTCTGACTAATTATTAGTAGT
TTHERM_00204150	coding	5Am-00204150_Fw	GCTGATGGCGATGAATGAACACTGTGATTGATATAAAGATATATAGAAAATGGA
		00204150-EGFP_Rv	AAGTTCTTCACCCTTAGAAACCATGGATCCTTAGATAAATAATTTTTATGGAACATTATC
	3' flanking	Neo4-00204150_Fw	CCCGGGGGATCTGAATTCGATATCAAGCTTTTCTATATTGGAATCTGCTACAATTCTAAG
		3Am-00204150_Rv	GCGAGCACAGAATTAATACGACTATTAATCTAATTCAAAAATTAGACCTATTC
TTHERM_00219320	coding	5'Am-00219320_Fw	GCTGATGGCGATGAATGAACACTGCGATAAATTAGCAAAAGATTAATTTGAGCA
		00219320-EGFP_Rv2	AAGTTCTTCACCCTTAGAAACCATGGATCCATAAATTATATGATTATTAAATTTTCTTTT
	3' flanking	Neo4-00219320__Fw2	CCCGGGGGATCTGAATTCGATATCAAGCTTACAAAATATTCATCGATAATCAGAAACTTC
		3'Am-00219320_Rv2	GCGAGCACAGAATTAATACGACTAAGTTGATCCTGAGACAAATTATCGAAGCT
TTHERM_00221110	coding	5Am-00221110_Fw	GCTGATGGCGATGAATGAACACTGCAACTAGATGACCTCTTAAATGAAAAATCC
		00221110-EGFP_Rv	AAGTTCTTCACCCTTAGAAACCATGGATCCTTTGCTTGGTTCGCTGCCGTCCTATTAATTTG
	3' flanking	Neo4-00221110_Fw	CCCGGGGGATCTGAATTCGATATCAAGCTTTGTTTCTTGGCAAAACAATATTTTCTATTG
		3Am-00221110_Rv	GCGAGCACAGAATTAATACGACTTCACAAAAACGAAATATATCTGGTTTATAG
TTHERM_00237610	coding	5Am-00237610_Fw	GCTGATGGCGATGAATGAACACTGCAACAGAAAAAATGATGGAAAAGCTGACTG
		00237610-EGFP_Rv	AAGTTCTTCACCCTTAGAAACCATGGATCCAAGAAGCTTCTCAATTCTTGATTTAAATCT
	3' flanking	Neo4-00237610_Fw	CCCGGGGGATCTGAATTCGATATCAAGCTTTCTATTAAAGCTTATTTTATATTTCGATGA
		3Am-00237610_Rv	GCGAGCACAGAATTAATACGACTTCATCTGTTTATAATCGAAGTTAATTTACT
TTHERM_00267890	coding	5Am-00267890_Fw	GCTGATGGCGATGAATGAACACTGGTCACTCAAACATCTCAAGGCTAATTTGCT
		00267890-EGFP_Rv	AAGTTCTTCACCCTTAGAAACCATGGATCCTTATGGATTTCCTTTATAATTAGT
	3' flanking	Neo4-00267890_Fw	CCCGGGGGATCTGAATTCGATATCAAGCTTAAGTATCTATTTACCTATCAATCAATCTCT
		3Am-00267890_Rv	GCGAGCACAGAATTAATACGACTCATGCTTGATAATTGATTATTAACAATCTC
TTHERM_00289290	coding	5Am-00289290_Fw	GCTGATGGCGATGAATGAACACTGCTTGACATCAGATGTAGCTCATGAGCTCAG
		00289290-EGFP_Rv	AAGTTCTTCACCCTTAGAAACCATGGATCCATTATTAAAGTAGCTTTTAGTTGCGTCTTAG
	3' flanking	Neo4-00289290_Fw	CCCGGGGGATCTGAATTCGATATCAAGCTTCGAGATAAATAAATATCTATGAACTCAAAG
		3Am-00289290_Rv	GCGAGCACAGAATTAATACGACTTGGTTTATTCTTCATATTTATGTGTAAGAG
TTHERM_00299879	coding	5Am-00299870_Fw	GCTGATGGCGATGAATGAACACTGGAAAAGTAGTCAATTCTTTGCGAACCCTGT
		00299870-EGFP_Rv	AAGTTCTTCACCCTTAGAAACCATGGATCCTTCATAGGCAGGTATTTTTTGGCAAAAAGA
	3' flanking	Neo4-00299870_Fw	CCCGGGGGATCTGAATTCGATATCAAGCTTGCTTATCCAGAAATTCATCCAAGTTTTTAG
		3Am-00299870_Rv	GCGAGCACAGAATTAATACGACTGAAGGAGTTGTCATATTAGATGAAGCTTAC
TTHERM_00301910	coding	5Am-00301910_Fw	GCTGATGGCGATGAATGAACACTGATGATAGAAAATTTTGACAAATTAATTTAG
		00301910-EGFP_Rv	AAGTTCTTCACCCTTAGAAACCATGGATCCTTCGTCAAATTCTTCTTCATCTTTTTC
	3' flanking	Neo4-00301910_Fw	CCCGGGGGATCTGAATTCGATATCAAGCTTTTTTGAGATGTGATGTATTCTTTCATGGGA
		3Am-00301910_Rv	GCGAGCACAGAATTAATACGACTACAAATATAGAGATTTATAGAAAGATCAAG
TTHERM_00309960*	coding	5Am-00309960_Fw2	GCTGATGGCGATGAATGAACACTGAGTTGACACCTAAAATAAGTAATTAAGGTG
		00309960-EGFP_Rv3	AAGTTCTTCACCCTTAGAAACCATGGATCCTATAATTAAGGTTGAAATGAATTATTAATC
	3' flanking	Neo4-00309960_Fw2	CCCGGGGGATCTGAATTCGATATCAAGCTTTGTTATTCATTTTGTAAGTTTTATTTACTC
		3Am-00309960_Rv2	GCGAGCACAGAATTAATACGACTTTTGCACACTTTTTTGATACATAAATTCTC
TTHERM_00341280	coding	5Am-00341280_Fw2	GCTGATGGCGATGAATGAACACTGGCAAGTTATACAAAACTCAGTTTATCC
		00341280-EGFP_Rv2	AAGTTCTTCACCCTTAGAAACCATGGATCCAGATTATTATGATTAGTAAAAACTGTTGC
	3' flanking	Neo4-00341280_Fw2	CCCGGGGGATCTGAATTCGATATCAAGCTTACTAAATGATGCAAAATAAAATTGTCTTC
		3Am-00341280_Rv2	GCGAGCACAGAATTAATACGACTAATCTAAAACATTTAACAACAGTATCTAG
TTHERM_00370840	coding	5Am-00370840_Fw	GCTGATGGCGATGAATGAACACTGTTATTTGATTGAAACATTTCCTTCTCTTGT
		00370840-EGFP_Rv	AAGTTCTTCACCCTTAGAAACCATGGATCCGTATTCATTAACAGCAAAAAATTCCTGT
	3' flanking	Neo4-00370840_Fw	CCCGGGGGATCTGAATTCGATATCAAGCTTCAGTAAATTATTGATTTCAGTGATATTAAG
		3Am-00370840_Rv	GCGAGCACAGAATTAATACGACTTTACCCTTATAATATAGATTTCAGTTGTTC
TTHERM_00402050	coding	5'Am-00402050_Fw	GCTGATGGCGATGAATGAACACTGTAGTTAAATAGCTCAGAAATCAGAAGAGTT
		00402050-EGFP_Rv	AAGTTCTTCACCCTTAGAAACCATGGATCCTTAAAACAATTTTCTATAAAATGTATTTATTGATTG
	3' flanking	Neo4-00402050_3'FLNK_Fw	CCCGGGGGATCTGAATTCGATATCAAGCTTCTTTATAATAAACTTACAACACAACTTCTA

		3'Am-00402050_Rv	GCGAGCACAGAATTAATACGACTCTTCAGTGTGTTAGACTGACGTGATAACCA
TTHERM_00412010a*	coding	5Am-00412010_Fw2	GCTGATGGCGATGAATGAACACTGGAATAAAAGTCTTTTGTTGCTTGAGGCAACG
		00412010-EGFP_Rv	AAGTTCTTCACCCTTAGAAACCATGGATCCTTAAAAAAAGCATGATTATGGTGGGGATAA
	3' flanking	Neo4-00412010_Fw	CCCGGGGGATCTGAATTCGATATCAAGCTTTATTATATTATTTTTCAATATCAGCCTGTA
		3Am-00412010_Rv	GCGAGCACAGAATTAATACGACTATATATGAAAATAGATTACGTTAGAGCCTA
TTHERM_00412010b*	coding	5Am-00412010_Fw2	GCTGATGGCGATGAATGAACACTGGAATAAAAGTCTTTTGTTGCTTGAGGCAACG
		00412010-EGFP_Rv-2	AAGTTCTTCACCCTTAGAAACCATGGATCCTATTTGATATAATAAAAAGCTGACATAGA
	3' flanking	Neo4-00412010_Fw	CCCGGGGGATCTGAATTCGATATCAAGCTTTATTATATTATTTTTCAATATCAGCCTGTA
		3Am-00412010_Rv	GCGAGCACAGAATTAATACGACTATATATGAAAATAGATTACGTTAGAGCCTA
TTHERM_00420400*	coding	5Am-00420400_Fw2	GCTGATGGCGATGAATGAACACTGAGAACTTAACTCTGAGGTTACTATGCTAAG
		00420400-EGFP_Rv_2	AAGTTCTTCACCCTTAGAAACCATGGATCCAAATTTCTTCTTTTTCTCTGTGGTGGAATA
	3' flanking	Neo4-00420400_Fw	CCCGGGGGATCTGAATTCGATATCAAGCTTATGTTACAAATTTTTATACAGTTATTTAGC
		3Am-00420400_Rv	GCGAGCACAGAATTAATACGACTAGTTACTGTAATATTAGTTCTAGAGGGAGA
TTHERM_00433790	coding	5Am-00433790_Fw	GCTGATGGCGATGAATGAACACTGTTTGAGTGCTGTAAATATCGATCCTAACCA
		00433790-EGFP_Rv	AAGTTCTTCACCCTTAGAAACCATGGATCCTTTATTTTATTTATATTATGACAAAAATCC
	3' flanking	Neo4-00433790_Fw	CCCGGGGGATCTGAATTCGATATCAAGCTTTTTTAATCATACATGAACACAGAAACAAATTTTAC
		3Am-00433790_Rv	GCGAGCACAGAATTAATACGACTATTGATCCATTGAAAAATAAATAGGAATCG
TTHERM_00439300	coding	5Am-00439300_Fw	GCTGATGGCGATGAATGAACACTGGATAGAGGAAAAATAAGTGAGTGAGGATTC
		00439300-EGFP_Rv	AAGTTCTTCACCCTTAGAAACCATGGATCCATCTCCGTATTCGTACTGATCCTCATCATC
	3' flanking	Neo4-00439300_Fw	CCCGGGGGATCTGAATTCGATATCAAGCTTTAGTTTCTACTTTTAAAGTTATATTTCTTG
		3Am-00439300_Rv	GCGAGCACAGAATTAATACGACTGTTTTATATTTAATGTAAGCTCACTTAGCTG
TTHERM_00442420	coding	5Am-00442420_Fw	GCTGATGGCGATGAATGAACACTGTGGAACAATTTAAGTTTGGCAGCTATCAGA
		00442420-EGFP_Rv	AAGTTCTTCACCCTTAGAAACCATGGATCCCTTATTAGGTATAAATGAGAATTTAATTAG
	3' flanking	Neo4-00442420_Fw	CCCGGGGGATCTGAATTCGATATCAAGCTTTTTTCAGTCCGCTTCATATATCAGTGTATC
		3Am-00442420_Rv	GCGAGCACAGAATTAATACGACTGCATTAAATAGATTAGGTAGCTTGCTTATC
TTHERM_00460720	coding	5Am-00460720_Fw	GCTGATGGCGATGAATGAACACTGTGATAATGAAAAGTAATCTCAGAGCAGC
		00460720-EGFP_Rv	AAGTTCTTCACCCTTAGAAACCATGGATCCAATTATTTTTTTAATAATTTATGTAATATC
	3' flanking	Neo4-00460720_Fw	CCCGGGGGATCTGAATTCGATATCAAGCTTTACTGAGTTTGATTTTTCTTAGCTGTTAG
		3Am-00460720_Rv	GCGAGCACAGAATTAATACGACTTTAGCCTTACTCCAAACTTTAACTTGTG
TTHERM_00471000	coding	5Am-00471000_Fw	GCTGATGGCGATGAATGAACACTGGTACTTCATAAATTAGGGTATTTTATAGTC
		00471000-EGFP_Rv	AAGTTCTTCACCCTTAGAAACCATGGATCCCATTTATTTTGATAAAATATTTTTATTC
	3' flanking	Neo4-00471000_Fw	CCCGGGGGATCTGAATTCGATATCAAGCTTTACATGTTTATTTGTTTTATTGGTTGGTTG
		3Am-00471000_Rv	GCGAGCACAGAATTAATACGACTGGCTTGAAGGAGATGACTCATTCAATGCTG
TTHERM_00471720	coding	5Am-00471720_Fw	GCTGATGGCGATGAATGAACACTGAAGGTACAAAAGTTGATATCAGACTGGATG
		00471720-EGFP_Rv2	AAGTTCTTCACCCTTAGAAACCATGGATCCAATGATATTTTATTTGCTTTTTGAAAAAGG
	3' flanking	Neo4-00471720_Fw	CCCGGGGGATCTGAATTCGATATCAAGCTTAAGATTTACAAGTTGTTTATTTTACAC
		3Am-00471720_Rv	GCGAGCACAGAATTAATACGACTCTATTGTACAATTTTTCTTTTACTATTAGC
TTHERM_00471730	coding	5Am-00471730_Fw	GCTGATGGCGATGAATGAACACTGGAGCGCACTTTATGAATTATTCTCTCGTG
		00471730-EGFP_Rv	AAGTTCTTCACCCTTAGAAACCATGGATCCATCATCATTTTCATCATCTTACTATTATC
	3' flanking	Neo4-00471730_Fw	CCCGGGGGATCTGAATTCGATATCAAGCTTCCTCAATTTAATATAAATCAATTCAATTTC
		3Am-00471730_Rv	GCGAGCACAGAATTAATACGACTCTTAATATTAAATCATCATTTATGACATAG
TTHERM_00474360	coding	5Am-00474360_Fw2	GCTGATGGCGATGAATGAACACTGCTCAACAGATTAAATGCCCAGCTAAAGAGC
		00474360-EGFP_Rv	AAGTTCTTCACCCTTAGAAACCATGGATCCATTAGTATATAGGTTGTCTTAGTAATCATCAAC
	3' flanking	Neo4-00474360_Fw	CCCGGGGGATCTGAATTCGATATCAAGCTTATGAATTGTTCATTTTTTATCTATTACCTC
		3Am-00474360_Rv	GCGAGCACAGAATTAATACGACTTATCTTATTAACGTTTCTAATATTAATATC
TTHERM_00474920	coding	5Am-00474920_Fw	GCTGATGGCGATGAATGAACACTGCGAAAATAATATGAATCCTACTAGAGGAGA
		00474920-EGFP_Rv	AAGTTCTTCACCCTTAGAAACCATGGATCCGCTTTGGATATTTTCACAACAGAAAGTG
	3' flanking	Neo4-00474920_Fw	CCCGGGGGATCTGAATTCGATATCAAGCTTGAGTTTATTTTAAGTATTCAAATGAACTTG
		3Am-00474920_Rv	GCGAGCACAGAATTAATACGACTTTGGAAAGTAAATAACATTTGAAGAGAGAG
TTHERM_00487030	coding	5Am-00487030_Fw	GCTGATGGCGATGAATGAACACTGGTAAGAAACACCATGCAGTCAAGTGCTTTG
		00487030-EGFP_Rv	AAGTTCTTCACCCTTAGAAACCATGGATCCGCTCTTTATCTTTATTTATTCTGCAACTTC
	3' flanking	Neo4-00487030_Fw	CCCGGGGGATCTGAATTCGATATCAAGCTTGTGTCCTATATATATTCTATCTATCCACTC
		3Am-00487030_Rv	GCGAGCACAGAATTAATACGACTATAAGGAAAGATTTGGATAATTATTACATG
TTHERM_00497670	coding	5Am-00497670_Fw	GCTGATGGCGATGAATGAACACTGGTAATAAACTAAATATTGGATGTAAGGCCA
		00497670-EGFP_Rv	AAGTTCTTCACCCTTAGAAACCATGGATCCATAAAAACTATCTACATTAAATTATTTAAACC
	3' flanking	Neo4-00497670_Fw	CCCGGGGGATCTGAATTCGATATCAAGCTTGCATCTTAAGAGAAAAGAGTTATACATTAC
		3Am-00497670_Rv	GCGAGCACAGAATTAATACGACTTAATACGTAAATGTTGAAGGTGTTGGAACT
TTHERM_00499370	coding	5Am-00499370_Fw	GCTGATGGCGATGAATGAACACTGATAAAGCAAATGACCTATCTTTTCCT
		00499370-EGFP_Rv2	AAGTTCTTCACCCTTAGAAACCATGGATCCATTTAAAACGTCTTCTTATTTTGC
	3' flanking	Neo4-00499370_Fw2	CCCGGGGGATCTGAATTCGATATCAAGCTTCATAAGTAATAGTTTTTCAAGAATTAGCAG
		3Am-00499370_Rv	GCGAGCACAGAATTAATACGACTAGTTGAAATTTTTCCTAATCCTAAGACCT
TTHERM_00501010	coding	5Am-00501010_Fw	GCTGATGGCGATGAATGAACACTGATTTCATAAATAGAGTAGTTGGCTCTTCCT
		00501010-EGFP_Rv	AAGTTCTTCACCCTTAGAAACCATGGATCCTTAGTTATGTTCTAATTTATATGAGACTGT
	3' flanking	Neo4-00501010_Fw	CCCGGGGGATCTGAATTCGATATCAAGCTTTTAACACAAACTTAATTAACCAACCTGATG
		3Am-00501010_Rv	GCGAGCACAGAATTAATACGACTACAGGACATCCCCATATAGCTTTGCATGAT
TTHERM_00522820	coding	5'Am-00522820_Fw	GCTGATGGCGATGAATGAACACTGAATTTCTAAAACGTTCAAGAGCCTCAAAAGA
		00522820-EGFP_Rv2	AAGTTCTTCACCCTTAGAAACCATGGATCCTTCTTATTTGTGTAATTCACTATTTTGTTT
	3' flanking	Neo4-00522820_3'FLNK_Fw3	CCCGGGGGATCTGAATTCGATATCAAGCTTGAGCTTAAAAATTTGAAGTAAGCTAATAAT
		3'Am-00522820_Rv3	GCGAGCACAGAATTAATACGACTTACGATAAAGTTAATCGCCTGTCCAAGCAG
TTHERM_00526270a*	coding	5Am-00526270_Fw	GCTGATGGCGATGAATGAACACTGACGATGATGATGAAGAATTAGATGATGATG
		00526270-EGFP_Rv	AAGTTCTTCACCCTTAGAAACCATGGATCCCTTCTTCTACAGTAAGTTTTATTTTTTTTATTG
	3' flanking	Neo4-00526270_Fw	CCCGGGGGATCTGAATTCGATATCAAGCTTATCATATTCATAATAAAATCTTTTTTTC
		3Am-00526270_Rv	GCGAGCACAGAATTAATACGACTATCATAAAGTAAACTCTTAGGGAGACTCCA
TTHERM_00526270b*	coding	5Am-00526270-2_Fw	GCTGATGGCGATGAATGAACACTGTAAGGAGGTAAACCAAACAAAGCTCACTAC
		00526270-2-EGFP_Rv	AAGTTCTTCACCCTTAGAAACCATGGATCCAATACAATTCTGATTTTATAATAAGTTACT
	3' flanking	Neo4-00526270-2_Fw	CCCGGGGGATCTGAATTCGATATCAAGCTTTATTGTGATTTGCTCAATAAATTGTCAGTG
		3Am-00526270-2_Rv	GCGAGCACAGAATTAATACGACTAAACAAGGGAAAATTAGGATTAATATTGTG
TTHERM_00529550	coding	5Am-00529550_Fw	GCTGATGGCGATGAATGAACACTGTAATAATGCTAAGCCACTCAAAAGTTAGCT
		00529550-EGFP_Rv	AAGTTCTTCACCCTTAGAAACCATGGATCCTTTATTCTAATTATTGATCACCTGTCTTG
	3' flanking	Neo4-00529550_Fw	CCCGGGGGATCTGAATTCGATATCAAGCTTAGAATGGCATAAATACTCTAAGAAAAAGGA
		3Am-00529550_Rv	GCGAGCACAGAATTAATACGACTTTAATTGTGATATAATCTACTACGAAGGTG

TTHERM_00540090	coding	5Am-00540090_Fw	GCTGATGGCGATGAATGAACACTGACGCAGCACTAATTTTACTAGGTTTTCAAG
		00540090-EGFP_Rv	AAGTTCTTCACCCTTAGAAACCATGGATCCGACTCTTGAAGAGGAATCTGGCTTTTCGAG
	3' flanking	Neo4-00540090_Fw	CCCGGGGGATCTGAATTCGATATCAAGCTTGATGCTAGATTTTCTTAACTAGCTTTAG
		3Am-00540090_Rv	GCGAGCACAGAATTAATACGACTTTTACTTGATTTGCTTTTCACCATTTTCTC
TTHERM_00564480	coding	5Am-00564480_Fw2	GCTGATGGCGATGAATGAACACTGCTTCAAATTCCAATGATGATAACGATGACAG
		00564480-EGFP_Rv	AAGTTCTTCACCCTTAGAAACCATGGATCCATTATACTTCTTGCTTAATTTTTAATCTAAG
	3' flanking	Neo4-00564480_Fw	CCCGGGGGATCTGAATTCGATATCAAGCTTGTATTATATTTCAACTTACCAGTGTTTATGG
		3Am-00564480_Rv	GCGAGCACAGAATTAATACGACTACCACTAAAAGAGCCATCCGAGTCCTAATC
TTHERM_00569460	coding	5Am-00569460_2_Fw	GCTGATGGCGATGAATGAACACTGATTAACATTAAAAGCTATCAAAACCTAGCT
		00569460-EGFP_Rv	AAGTTCTTCACCCTTAGAAACCATGGATCCTATTGAAAACTTCTTAAAGTTCTCATAAGA
	3' flanking	Neo4-00569460_Fw	CCCGGGGGATCTGAATTCGATATCAAGCTTAAAGATTCAATCTATGACTCTTGCTGCAGT
		3Am-00569460_Rv	GCGAGCACAGAATTAATACGACTCTCCAAAAGAGTAAGTGACTATTATTTAGC
TTHERM_00572190	coding	5'Am-00572190_Fw	GCTGATGGCGATGAATGAACACTGGAGGAAAACAAGCAGTTTGTTGAGACGTAT
		00572190-EGFP_Rv	AAGTTCTTCACCCTTAGAAACCATGGATCCATTAAAATATGTTTCGATGATCGATTACAG
	3' flanking	Neo4-00572190_3'FLNK_Fw	CCCGGGGGATCTGAATTCGATATCAAGCTTCTGTATAGATTTAATTAAGATGTTAAACTA
		3'Am-00572190_Rv	GCGAGCACAGAATTAATACGACTGCCATTGACATGCCCTTACTTTTACTTTCT
TTHERM_00616290	coding	5Am-00616290_Fw	GCTGATGGCGATGAATGAACACTGGGTGGTAGTAGAGGAGTAATTACAAGATCA
		00616290-EGFP_Rv	AAGTTCTTCACCCTTAGAAACCATGGATCCACTCTTTACAATTTTTCTTTTTTATATTTTTTATTTTG
	3' flanking	Neo4-00616290_Fw	CCCGGGGGATCTGAATTCGATATCAAGCTTAATACTTCATGTAAAATATTGCTGTAC
		3Am-00616290_Rv	GCGAGCACAGAATTAATACGACTAACTGCTTACTTATCTTTTAGATAAGC
TTHERM_00622830	coding	5Am-00622830_Fw	GCTGATGGCGATGAATGAACACTGCACATACCTTGAGAAAATGAGCAAAGAATC
		00622830-EGFP_Rv	AAGTTCTTCACCCTTAGAAACCATGGATCCTGGCTAGCTAATATTCCTTTCGCTTATATTC
	3' flanking	Neo4-00622830_Fw	CCCGGGGGATCTGAATTCGATATCAAGCTTATCATTTATGTATGTATGTAAAGCTCTATC
		3Am-00622830_Rv	GCGAGCACAGAATTAATACGACTAGCAATTTGAATAAAATTCTAAACAGATGC
TTHERM_00637050	coding	5Am-00637050_Fw	GCTGATGGCGATGAATGAACACTGATAGTTAGAGAGGAATTAGTAATCTAAGCT
		00637050-EGFP_Rv	AAGTTCTTCACCCTTAGAAACCATGGATCCTGCTTCAACAATTTCACTATGGTTTGGAG
	3' flanking	Neo4-00637050_Fw	CCCGGGGGATCTGAATTCGATATCAAGCTTAGAAATTTTTCTTCCAATTCTCCCTAAATC
		3Am-00637050_Rv	GCGAGCACAGAATTAATACGACTATGTCAAATCTTCTCTCCTAAATTAATC
TTHERM_00649180	coding	5Am-00649180_Fw	GCTGATGGCGATGAATGAACACTGAACGATAAGGGTGATATCGATGTTGATAGC
		00649180-EGFP_Rv2	AAGTTCTTCACCCTTAGAAACCATGGATCCGAAGGTAATGGATTCATCGTTATCATTAGG
	3' flanking	Neo4-00649180_Fw	CCCGGGGGATCTGAATTCGATATCAAGCTTTATGTATCACAAATTCCATCTTCTAACACA
		3Am-00649180_Rv	GCGAGCACAGAATTAATACGACTGAAGAAATTATGCATGTTTTGTGAAAATCA
TTHERM_00664050	coding	5'Am-00664050_Fw	GCTGATGGCGATGAATGAACACTGCGAAGACAAACAACAGAGGATAGTATGGGA
		00664050-EGFP_Rv	AAGTTCTTCACCCTTAGAAACCATGGATCCGTATAGTCTACGATAATGTTCATTTATGCC
	3' flanking	Neo4-00664050_3'FLNK_Fw	CCCGGGGGATCTGAATTCGATATCAAGCTTTTTTGTTTGTTTATTTGTTGTCAATTTCTG
		3'Am-00664050_Rv	GCGAGCACAGAATTAATACGACTTAAGTGCGTGCTGGAATGAAATTGAAATAG
TTHERM_00693080	coding	5Am-00693080_Fw	GCTGATGGCGATGAATGAACACTGTTCTTATTGACTGGCTTACTGAAGTTTCCA
		00693080-EGFP_Rv	AAGTTCTTCACCCTTAGAAACCATGGATCCAATATTGGTTAGTTGTTAAGATTATTATTAG
	3' flanking	Neo4-00693080_Fw	CCCGGGGGATCTGAATTCGATATCAAGCTTAATAGAGCCATAAAACTATACTTATTGTTG
		3Am-00693080_Rv	GCGAGCACAGAATTAATACGACTCAATAGGATATTATAACTGTGTGCTCTTAG
TTHERM_00704020*	coding	5Am-00704020_Fw	GCTGATGGCGATGAATGAACACTGCATCCTCATTCAGTAATCATTTAATCTTAC
		00704020-EGFP_Rv	AAGTTCTTCACCCTTAGAAACCATGGATCCTTTTTTCTTTTTACTAAACTTTGCAAAAGC
	3' flanking	Neo4-00704020_Fw	CCCGGGGGATCTGAATTCGATATCAAGCTTACATATTCACTAAATTGAAGTCAAAATACT
		3Am-00704020_Rv	GCGAGCACAGAATTAATACGACTATTCTTCATAAACTTAACACATTTATCAAC
TTHERM_00706330	coding	5Am-00706330_Fw	GCTGATGGCGATGAATGAACACTGTTCAAAGCCTATATCAAAGGGAGTAATAGA
		00706330-EGFP_Rv	AAGTTCTTCACCCTTAGAAACCATGGATCCGTTAATTAATAAAACCTCCAATTCTTAATTTTC
	3' flanking	Neo4-00706330_Fw	CCCGGGGGATCTGAATTCGATATCAAGCTTCTCATTAATAAGTGCTTGATTGATTTATGA
		3Am-00706330_Rv	GCGAGCACAGAATTAATACGACTTGCAAAACACACAGAAAGTATGCTATTTGC
TTHERM_00728900	coding	5Am-00728900_Fw	GCTGATGGCGATGAATGAACACTGTGAATAATTTTGAAACATTATGGAGAAATC
		00728900-EGFP_Rv	AAGTTCTTCACCCTTAGAAACCATGGATCCAACTTATGCTTATTTTGAATCTAATTATG
	3' flanking	Neo4-00728900_Fw	CCCGGGGGATCTGAATTCGATATCAAGCTTTTGAAGAGAGTGGCAGCAAGATTCATCA
		3Am-00728900_Rv2	GCGAGCACAGAATTAATACGACTCTTTCACATTCTACTTTAGAGGAAGTATTC
TTHERM_00775940	coding	5Am-00775940-1_Fw	GCTGATGGCGATGAATGAACACTGCTATATTTGGTGTAGGGATTAGTTTGCTCG
		00775940-1-EGFP_Rv	AAGTTCTTCACCCTTAGAAACCATGGATCCCTATTTCAAGCTACCAAAACCTTGCTTAAT
	3' flanking	Neo4-00775940-1_Fw	CCCGGGGGATCTGAATTCGATATCAAGCTTATGAGCAAGGACTCTTATATCTATCTATAC
		3Am-00775940-1_Rv	GCGAGCACAGAATTAATACGACTACTACTGTCTAAAACAATGTAGATTTATGG
TTHERM_00775949	coding	5Am-00775940-2_2_Fw	GCTGATGGCGATGAATGAACACTGTACTTCCTAAAAAGCACTTAAAATCAGGCT
		00775940-2-EGFP_Rv	AAGTTCTTCACCCTTAGAAACCATGGATCCCAACGTGTTTAGAAAGCTTAAAAGTAATAAAC
	3' flanking	Neo4-00775940-2_Fw	CCCGGGGGATCTGAATTCGATATCAAGCTTTTCATTCTAATTTAAACCTCAGAATTACAC
		3Am-00775940-2_Rv	GCGAGCACAGAATTAATACGACTTCACTTCTTATTCGTTTGCAAACATTCGTG
TTHERM_00825660	coding	5Am-00825660_Fw	GCTGATGGCGATGAATGAACACTGGAATTCGATGATGATGAAGAAGAAGTAGAG
		00825660-EGFP_Rv	AAGTTCTTCACCCTTAGAAACCATGGATCCATTCTTTTTAGTTTTCTTTTAGGATCTG
	3' flanking	Neo4-00825660_Fw	CCCGGGGGATCTGAATTCGATATCAAGCTTTCTATTCATATTATTTGTTTATGTTCG
		3Am-00825660_Rv	GCGAGCACAGAATTAATACGACTCATAACTTAAAACAAGTAACAATGAGTC
TTHERM_00849260	coding	5'Am-00849260_Fw	GCTGATGGCGATGAATGAACACTGGTGAATAATTTAATAATGGTTATCCTCGCT
		00849260-EGFP_Rv	AAGTTCTTCACCCTTAGAAACCATGGATCCTTCTTGCTTCTACTAAATTTAAATAGTATC
	3' flanking	Neo4-00849260_Fw	CCCGGGGGATCTGAATTCGATATCAAGCTTCTAAGTGGATATTTCAAATTACATATCATG
		3'Am-00849260_Rv	GCGAGCACAGAATTAATACGACTATTAATACAATAATATGCTTTGCGTCCACT
TTHERM_00974120	coding	5Am-00974120_Fw	GCTGATGGCGATGAATGAACACTGTGAAATAGTTTAATAATAGGTCTGTGTGTG
		00974120-EGFP_Rv	AAGTTCTTCACCCTTAGAAACCATGGATCCTTGTTTTAAGTATATATCATACTATTTG
	3' flanking	Neo4-00974120_Fw	CCCGGGGGATCTGAATTCGATATCAAGCTTGCTTAAAAGCAATGATTTCAAGATCTCTGC
		3Am-00974120_Rv	GCGAGCACAGAATTAATACGACTGATAATAGAAATAGATTTATATACCCACTG
TTHERM_01014530	coding	5Am-01014530_Fw	GCTGATGGCGATGAATGAACACTGAGAAAAGGAACTTCCTCCATATTGCAAGGA
		01014530-EGFP_Rv	AAGTTCTTCACCCTTAGAAACCATGGATCCTTAAGGGGATTGTTGAGAGTTTGATTTCTG
	3' flanking	Neo4-01014530_Fw	CCCGGGGGATCTGAATTCGATATCAAGCTTATTAAACTATCAAATTAGATTCATTCTCA
		3Am-01014530_Rv	GCGAGCACAGAATTAATACGACTTGAGAACGAACTTGAATACCAGTCGGT
TTHERM_01014770	coding	5Am-01014770_Fw	GCTGATGGCGATGAATGAACACTGACAATCTACCAATTGGTAAAGTGACTATGG
		01014770-EGFP_Rv	AAGTTCTTCACCCTTAGAAACCATGGATCCATCCTTTTTGAGCATTTTATCTGGGTTTCC
	3' flanking	Neo4-01014770_Fw	CCCGGGGGATCTGAATTCGATATCAAGCTTTCAACTTATTTTGATATTATGTCTGAATTG
		3Am-01014770_Rv	GCGAGCACAGAATTAATACGACTGTTGATTTGTAAAAATTTGATCAAAAATAC
TTHERM_01085480	coding	5Am-01085480_Fw	GCTGATGGCGATGAATGAACACTGGATATAGCCCAACGAAGAATCTTTTAATAC

		01085480-EGFP_Rv	AAGTTCTTCACCCTTAGAAACCATGGATCCCACAAAGTATTTTTTGTTTGATCTCTTAAT
	3' flanking	Neo4-01085480_Fw	CCCGGGGGATCTGAATTCGATATCAAGCTTTTATCTTGGAATTACTGTGGAATTTCTCAG
		3Am-01085480_Rv	GCGAGCACAGAATTAATACGACTACAAAATACTCAGGATGAAGAGATTAGTTC
TTHERM_01132870	coding	5Am-01132870_Fw	GCTGATGGCGATGAATGAACACTGGAGGGAAATGATATTGATTAAATTTTGGCA
		01132870-EGFP_Rv2	AAGTTCTTCACCCTTAGAAACCATGGATCCGCTTTTTTAATCTTTTTTTGTTGATGATC
	3' flanking	Neo4-01132870_Fw2	CCCGGGGGATCTGAATTCGATATCAAGCTTAGTTTATAAATAAAAATTCTTCCTTTCATC
		3Am-01132870_Rv2	GCGAGCACAGAATTAATACGACTGATAACTTATTCATATCAGATTTCACTGTG
TTHERM_01276320	coding	5'Am-01276320_Fw	GCTGATGGCGATGAATGAACACTGGAGATTTCCTAAGTTTTATTATGAAAATACATTTATGGGTC
		01276320-EGFP_Rv2	AAGTTCTTCACCCTTAGAAACCATGGATCCGCTTTGTAATCTCTAAGGACATATAGATTT
	3' flanking	Neo4-01276320_3'FLNK_Fw3	CCCGGGGGATCTGAATTCGATATCAAGCTTGAAAACCTTTTAAAAATTGATTTTTGTGAG
		3'Am-01276320_Rv3	GCGAGCACAGAATTAATACGACTCTAATCAATACTTTGATAGGTACATTAGAGACT
TTHERM_01285910	coding	5Am-01285910_Fw	GCTGATGGCGATGAATGAACACTGAGACGAGAGCTTTTTAAATTATCAAAGCCA
		01285910-EGFP_Rv	AAGTTCTTCACCCTTAGAAACCATGGATCCATTAAAGTCAATCAATTCATATAAAATATTCG
	3' flanking	Neo4-01285910_Fw	CCCGGGGGATCTGAATTCGATATCAAGCTTTGATAATATTCAATGTAATATTTTAGTTCG
		3Am-01285910_Rv	GCGAGCACAGAATTAATACGACTAATGTAGTAGACTTTCCTACATTACGCTTG
TTHERM_01337400	coding	5'Am-01337400_Fw	GCTGATGGCGATGAATGAACACTGGATTGTCCCTGAATAAATAGATGTGGACTG
		01337400-EGFP_Rv	AAGTTCTTCACCCTTAGAAACCATGGATCCTTTAGCTATTTATTACTATTATTTCTATATTAG
	3' flanking	Neo4-01337400_Fw	CCCGGGGGATCTGAATTCGATATCAAGCTTTATATCTCTTCATAACTTAAATTGCTATTC
		3'Am-01337400_Rv	GCGAGCACAGAATTAATACGACTTTATCAGAGAGGATCCTGAGGTTGTCAAAG
TTHERM_01358410	coding	5Am-01358410_Fw	GCTGATGGCGATGAATGAACACTGATTTCCATAAGCTGAATGCGATGCGTTCTC
		01358410-EGFP_Rv	AAGTTCTTCACCCTTAGAAACCATGGATCCGTTGTTTTATTAACAAACCCATGGGTGGTT
	3' flanking	Neo4-01358410_Fw	CCCGGGGGATCTGAATTCGATATCAAGCTTTTGTAATTTATTTTATCTCCTATGATTTTG
		3Am-01358410_Rv	GCGAGCACAGAATTAATACGACTTTAGCATTCGCTATTTAAACTTAATATTTG
TTHERM_01367700	coding	5'Am-01367700_Fw	GCTGATGGCGATGAATGAACACTGGATTAATAAGCTGTTAAAAGTGAAAGCAAA
		01367700-EGFP_Rv2	AAGTTCTTCACCCTTAGAAACCATGGATCCTTTTTTAATAATTTTAAAGTGTTTAATAGG
	3' flanking	Neo4-01367700_3'FLNK_Fw	CCCGGGGGATCTGAATTCGATATCAAGCTTTTGTAAATTCTTTTGGTTTTTTAATGGAGT
		3'Am-01367700_Rv	GCGAGCACAGAATTAATACGACTTGAACAGTGTTTTTTATTCCTATTAATGGA

Overlapping and adapter sequences are underlined and dotted underlined, respectively.

b) Primers for the overlapping PCR

name	sequence (5' -> 3')
5’RACE Outer	GCTGATGGCGATGAATGAACACTG
3’RACE Outer	GCGAGCACAGAATTAATACGACT

Primers used for the other experiments

name	sequence (5' -> 3')
00237610_KO5Fw	AACAGCTATTTTGAATACATTAGTGGAG
00237610_KO5Rv	GTCTATCGAATTCCTGCAGCCCATAAATTTAATGAATCTGAATGCTTTC
00237610_KO3Fw	CTGGAAAAATGCAGCCCTCTATTAAAGCTTATTTTATATTTCGATGA
00237610_Fw1	AATTGTCAGAAAAAATGAAAAAAATTATC
00237610_Rv4	GTTAATTAAAATAAATTGATTAATAATAC
00237610_Fw9	GTTAAGCTTAATATAAAATATACGATCC
BTU1_5Fw	AATATGTGAAAATATCAAGCGAACTGAC
BTU1_5bsrRv	CCATACTTTGAAGATATCTGTCGACCTTTTCATCACCCAAATAAATACACGCA
BTU1_3bsrFw	TAATATTTTTTTTTGCCGCGGCCGCAATTCTTACTTCTACATGTTTCCTTTC
BTU1_3Rv	TGCTATAAGTCGCTTTTTAAAGAACTAAG
BX-5FNK_PDD1_FwL	AGGCTCGCCCTAGGTCACTCGAGCACATAAATTTAATCCAAATCATCAATCAC
SSB-5FNK_PDD1_Rv	CAGTGAGTCGACGAGTCCACTAGTACGTCGGGATCCTTTCTTTATTGAAATATTTATTGCTTTTTTAGC
SSS-3FNK_PDD1_Fw	CGACGTACTAGTGGACTCGTCGACTCACTGCCCGGGTTTTATCAAATTAAAATTTAAATAGGATTGATTG
KX-3FNK_PDD1_RvL	AGGCTCGCGGTACCAACCTCGAGTAGACCAATTATCGAGAATGTTGATAATAGC
SpeI-PDD1_3UTR_Fw	CGTCGAACTAGTTAAATTATTTAGTGATTACGGCTTGATTAAGC
SalI-PDD1_3UTR_Rv	CGTCGAGTCGACTATTTTAATTGAATAAATTTTCATAAATTAAAATGTG
BamHI-PDD1ORF_Fw	AGTCAGGATCCATGTCTCAGAAAAAGAGTTTAAAATAAAAGAG
SpeI-PDD1ORF_Rv2	AACACTGACTAGTTCAATGAGTAAGTTATTAATTAGCTTGTC
5Am-Pdd1_Fw	GCTGATGGCGATGAATGAACACTGATGTCTCTTAGCCATAATGGGTAGATGTTG
EcoRI-opt.PDD1_Fw	AGCAGAATTCATGAGCCAAAAAAAATCCCTGAAACAAAAACG
opt.PDD1_W72,75A_Rv1	GGTTGAATCTTCGATCGGGGCGTTCTCGGCTTTCACCAGATACTC
opt.PDD1_W72,75A_Fw1	GAGTATCTGGTGAAAGCCGAGAACGCCCCGATCGAAGATTCAACC
PstI-TGA-opt.PDD1_Rv2	ATCGCTGCAGTCAATGGGTCAGTTGCTGATTTGCCTGGC
PstI-TGA-opt.PDD1_I478D_RvMAL	ATCGCTGCAGTCAATGGGTCAGTTGCTGATTTGCCTGGCGCAGCTGTGACTGATTGGAGTGTTGCAGCAGAAAGTCGTCCAGGACTTGTGG
ERI-opt.PDD1_HNG1_Fw	AGCAGAATTCAAAAAACAGAAAGCCAACGTGATGCCTCAACC
PstI-TGA-opt.PDD1_HING1_Rv	ATCGCTGCAGTCACAGTTCCGTTTCATCGGCATCGGCGTTATTCG
ERI-opt.PDD1_HNG2_Fw	AGCAGAATTCATCAAAGATGACGTGATCGCCTATGAGGAC
PstI-TGA-opt.PDD1_HING2_Rv	ATCGCTGCAGTCAAATTTCAGTCAGTTCAATCGGGCGACGTTTC
EcoRI-opt.PDD1_FwpGEX	CACGAATTCTGATGAGCCAAAAAAAATCCCTGAAACAAAAACG
XhoI-opt.PDD1_RvpGEX	CATCTCGAGTATGGGTCAGTTGCTGATTTGCCTGGC
ERI-opt.MIM22_FwGEX	CAGCGAATTCTGATGTCTCAAAAAAAAAGCCTG
XhoI-opt.MIM22_RvGEX	CAGTCTCGAGTGTGCGTCAACTGCTGGTTGG
T7-opt.EGFP_Fw1	GTAATACGACTCACTATAGGGAGAATGGTTTCTAAGGGTGAAGAAC
opt.EGFP_Rv1	CTTATATAATTCATCCATACCAAGAGTAATACC
T7-CAM-IES_Fw1	GTAATACGACTCACTATAGGGAGAAATTTACAAAAGTTTGAATAATGAATTTTAGCAC
CAM-IES_Rv1	TTATTTTAAGATTTATTTTCAATTTGATTCAAG

A map and the sequence of pPPLNP1

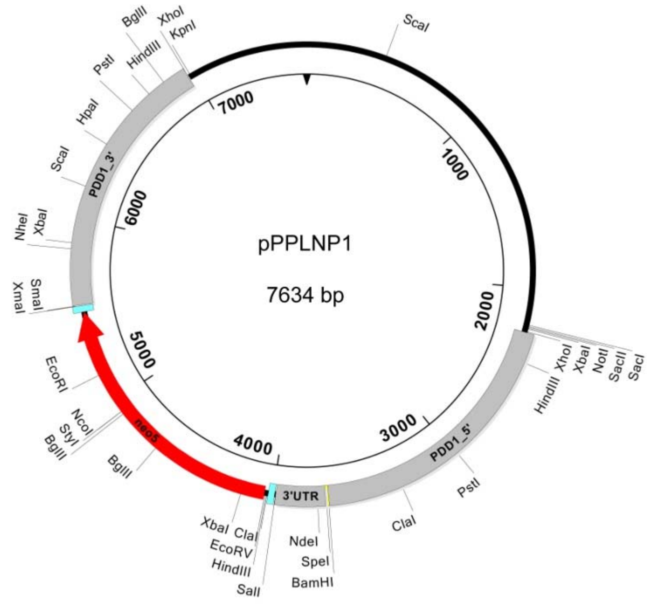


```
>pPPLNP1
GCACTTTTCGGGGAAATGTGCGCGGAACCCCTATTTGTTTATTTTTCTAAATACATTCAAATATGTATCCGCTCATGAGACAATAACCCTGATAAATGCTTCAATAATATTGAAAAAGGAAGAGTATGAGTATTCAACATT
TCCGTGTCGCCCTTATTCCCTTTTTTGCGGCATTTTGCCTTCCTGTTTTTGCTCACCCAGAAACGCTGGTGAAAGTAAAAGATGCTGAAGATCAGTTGGGTGCACGAGTGGGTTACATCGAACTGGATCTCAACAGCGGTA
AGATCCTTGAGAGTTTTCGCCCCGAAGAACGTTTTCCAATGATGAGCACTTTTAAAGTTCTGCTATGTGGCGCGGTATTATCCCGTATTGACGCCGGGCAAGAGCAACTCGGTCGCCGCATACACTATTCTCAGAATGACT
TGGTTGAGTACTCACCAGTCACAGAAAAGCATCTTACGGATGGCATGACAGTAAGAGAATTATGCAGTGCTGCCATAACCATGAGTGATAACACTGCGGCCAACTTACTTCTGACAACGATCGGAGGACCGAAGGAGCTAA
CCGCTTTTTTGCACAACATGGGGGATCATGTAACTCGCCTTGATCGTTGGGAACCGGAGCTGAATGAAGCCATACCAAACGACGAGCGTGACACCACGATGCCTGTAGCAATGGCAACAACGTTGCGCAAACTATTAACTG
GCGAACTACTTACTCTAGCTTCCCGGCAACAATTAATAGACTGGATGGAGGCGGATAAAGTTGCAGGACCACTTCTGCGCTCGGCCCTTCCGGCTGGCTGGTTTATTGCTGATAAATCTGGAGCCGGTGAGCGTGGGTCTC
GCGGTATCATTGCAGCACTGGGGCCAGATGGTAAGCCCTCCCGTATCGTAGTTATCTACACGACGGGGAGTCAGGCAACTATGGATGAACGAAATAGACAGATCGCTGAGATAGGTGCCTCACTGATTAAGCATTGGTAAC
TGTCAGACCAAGTTTACTCATATATACTTTAGATTGATTTAAAACTTCATTTTTAATTTAAAAGGATCTAGGTGAAGATCCTTTTTGATAATCTCATGACCAAAATCCCTTAACGTGAGTTTTCGTTCCACTGAGCGTCAG
ACCCCGTAGAAAAGATCAAAGGATCTTCTTGAGATCCTTTTTTTCTGCGCGTAATCTGCTGCTTGCAAACAAAAAAACCACCGCTACCAGCGGTGGTTTGTTTGCCGGATCAAGAGCTACCAACTCTTTTTCCGAAGGTAA
CTGGCTTCAGCAGAGCGCAGATACCAAATACTGTCCTTCTAGTGTAGCCGTAGTTAGGCCACCACTTCAAGAACTCTGTAGCACCGCCTACATACCTCGCTCTGCTAATCCTGTTACCAGTGGCTGCTGCCAGTGGCGATA
AGTCGTGTCTTACCGGGTTGGACTCAAGACGATAGTTACCGGATAAGGCGCAGCGGTCGGGCTGAACGGGGGGTTCGTGCACACAGCCCAGCTTGGAGCGAACGACCTACACCGAACTGAGATACCTACAGCGTGAGCTAT
GAGAAAGCGCCACGCTTCCCGAAGGGAGAAAGGCGGACAGGTATCCGGTAAGCGGCAGGGTCGGAACAGGAGAGCGCACGAGGGAGCTTCCAGGGGGAAACGCCTGGTATCTTTATAGTCCTGTCGGGTTTCGCCACCTCT
GACTTGAGCGTCGATTTTTGTGATGCTCGTCAGGGGGGCGGAGCCTATGGAAAAACGCCAGCAACGCGGCCTTTTTACGGTTCCTGGCCTTTTGCTGGCCTTTTGCTCACATGTTCTTTCCTGCGTTATCCCCTGATTCTG
TGGATAACCGTATTACCGCCTTTGAGTGAGCTGATACCGCTCGCCGCAGCCGAACGACCGAGCGCAGCGAGTCAGTGAGCGAGGAAGCGGAAGAGCGCCCAATACGCAAACCGCCTCTCCCCGCGCGTTGGCCGATTCATT
AATGCAGCTGGCACGACAGGTTTCCCGACTGGAAAGCGGGCAGTGAGCGCAACGCAATTAATGTGAGTTAGCTCACTCATTAGGCACCCCAGGCTTTACACTTTATGCTTCCGGCTCGTATGTTGTGTGGAATTGTGAGCG
GATAACAATTTCACACAGGAAACAGCTATGACCATGATTACGCCAAGCTCGAAATTAACCCTCACTAAAGGGAACAAAAGCTGGAGCTCCACCGCGGTGGCGGCCGCTCTAGAACTAGGTCACTCGAGCACATAAATTTAA
TCCAAATCATCAATCACAAAATAAATAGAAATATTTATTTTATACAAACAGCTGAAATAAAGAAAATATTTTACATTAGCTGTTTAACTCTATTCTAAAATTCAATGCAAATTTAATGCCTTTGCTGAAAATAAATTATAA
GCTTTTTGATAAATATCGCTAAAATGCTGATTGATAATACCATTTTTGATTTACTTTTTAAATCTGTTTAATAAAAATATAAAAAGTTGCTCAAAAATCTGATTTTTTTTTGATATTGAAATCAGATTAAAATCTAAAATT
AAGGCTAACAAATTTATTATTATTATTCAAAAGAACCCATAATTTATTACATTATAAGAAAGTTTAAATATTGGAAGGCCTTTGCCGTAATGAAAAACTTTATCATCAGTAGGTAATCCTTACCTATATATTTCTAATGAT
GAAGGAGATGGCCATATCCCTCGGGATTGAAAATCATGAAAAACTGGCTGCAATGCTGATCAAAGAAATATAAATTTAAGGATATACCGTACAAATTAAATAGCAAAACAAGCTACTCTATTATAAATTTTTCGAAAGAAA
ATATATAGAGTGTAGCTTAAGCTATAATTATATATATTTAATTATGCATATTTACTAAATTAATACTGCTATAAAATTCCCTATCACGAAAAGTCCAAGTGATGGAATAAAGAATTTACCTTATACAGTTAAAATATTTTA
GTTACCATTGCGCACTGCAGCAAATAAACATGATTGTTGAGGTTTTCTCAATAATTTGTTTGTTTTGGTAATCTGACATAACAACTCATATAAAAAAAACAAAACACTTCTCTTTTTTTCTTGATTAAAAAAAATTTAAAT
TTTCAATCCCTATTTCAACGATAGGTAAATAAAAAGAAATTTATTTATGCTACAAATACAAAAAATATTACAAAAAGAATTTAAATAAATTACTAAGTCGGTATTGTCTTAGTCATTCATTTATTTATTTATTTTTCAAAG
TTTAAAAAAGAATAAAATCAATAAAAAATAAAGCATTCATCTTTAAATAAAATATGATTAATTCAACATCGATTTATTTATTTAGATTTGTATTCATAAAATCTTATTGTTTTTTAGATTGATTAGAATAAATAATTAATA
TTTTAATCCTACCTACTAATAAATTTCTAAAATTTTAATTGATTTTGTTTGTCTATTTTTGATATTTGAGATTTCATCCGAATTATTTGAGTCTTTTTCACTAAGTTTAATTAGGTAAACAACTTTAAACATTTAGAATTT
AATTTAATTAAAACTATTGATTAATGCTTTGATGTTTACTGTGAAATCACTCTAATAATTTGTAAATAATTATTTTAAATCCAACCAAATATTTTGTTAATTTTATAAAAATAATAAATTTAAAAATTCTCCTAATTAGTA
AGCTAAAAAAGCAATAAATATTTCAATAAAGAAAGGATCCcgacgtACTAGTTAAATTATTTAGTGATTACGGCTTGATTAAGCTCTTAAAACATATGATGAACTCAATAAATTTTCTTATTAGAAACCAAAAAAATACTT
GGAAAAAAATTTTTAAAATAAATATATTTCTCATGATAAAAATATATTTTTTTGAAAAATATTGCTGATTATTTATTTATTAATTATTACATTTTACATATTTTGTAACGATTGAGTTTTTAGTTTAAAGAGTTTTAATTT
CACATTTTAATTTATGAAAATTTATTCAATTAAAATAGTCGACATAACTTCGTATAATGTATGCTATACGAAGTTATGGTATCGATAAGCTTGATATCTTCAAAGTATGGATTAATTATTTCAAATTATTAGAAGGTAATT
AATCTGCATAAATTCAAAACTATAAAAATAAAACATTAAAATTAATTCAACCTTATTGAAGCATCAAAATCTGAATCTCTAGAAAGACTGATTCTGATTGGATAATTTTTCGGCGCTAAGGATTTTGGATTAAAGAAAATT
AGATTTAATTATTAATCATGATTTGAATAGGATAGCAAGAATATTTGTTTGGTTTAAAAGGGAAAGCGGGTAATTATCAAAAATTTATAAATAATTTTAAAACAATAAATAGAAAAACAAATAAGATTATAAAAACTTACA
AAAATGATTGAACAAGATGGTTTACACGCTGGTTCTCCCGCCGCTTGGGTCGAAAGACTTTTCGGTTATGACTGGGCTCAACAAACCATCGGTTGCTCTGATGCCGCCGTCTTCCGTCTTTCTGCTCAAGGTCGTCCTGTT
CTTTTCGTCAAGACCGACCTTTCTGGTGCCCTTAATGAACTTCAAGATGAAGCTGCCCGTCTTTCTTGGCTTGCCACCACCGGTGTTCCTTGCGCTGCTGTCCTTGACGTTGTCACTGAAGCCGGTAGAGACTGGCTTCTT
TTAGGTGAAGTCCCCGGTCAAGATCTTCTTTCTTCTCACCTTGCTCCTGCCGAAAAAGTTTCTATCATGGCTGATGCTATGCGTCGTCTTCATACCCTTGATCCCGCTACCTGCCCTTTCGACCACCAAGCCAAACATCGT
ATCGAACGTGCTCGTACTCGTATGGAAGCCGGTCTTGTCGATCAAGATGATCTTGACGAAGAACATCAAGGTCTTGCCCCTGCCGAACTTTTCGCCAGACTTAAGGCCCGTATGCCCGACGGTGAAGATCTTGTCGTCACC
CATGGTGATGCCTGCTTACCCAATATCATGGTTGAAAATGGTCGTTTTTCTGGTTTCATCGACTGTGGTCGTCTTGGTGTCGCCGACCGTTATCAAGATATTGCCTTAGCTACCCGTGATATTGCTGAAGAACTTGGTGGT
GAATGGGCTGACCGTTTCCTTGTCCTTTACGGTATCGCCGCTCCCGATTCTCAACGTATCGCCTTCTATCGTCTTCTTGACGAATTCTTCTGAGATCCTTAAATTAAAAATTCAATATATATTTACAAACTTTCATATAAA
ATAAATATATTATATAAAATTAATTTTTAGTGTATTATATTAACATTAAAGCACCAAAAAAACGTGTTAATATACTACTATAAAATATAATTTATTCCAAATTGACTAAAATCATTATTTTACAACTCATTTGTATATATA
TTTTATGTCAATTATTTTTTTTAACTTTCTAAAAAAAAAAATTCCTCTTCACATACATGTTAGCTCTTAAAAATTTGTCTGCAAATCCAATAATAATATTTTTTTTTGCCATTAAATTTTCAAATTTTTACTGGAAAAATG
CAGCATAACTTCGTATAATGTATGCTATACGAAGTTATCCCGGGTTTTATCAAATTAAAATTTAAATAGGATTGATTGATTTAAAAAAGATTAAATTAGGAAAATGGTTTGATTTATTTAAAACATAATCGAAACATAAAA
AATTAATATAGTATTATTCCATGTATTTATAAATTAGACTCAATAGATTTAGTTTTATGGTGTAGCTTCTTCTTAAACATCTTAGATTATTTGTTAATATAGTTAAACTCCTATTTTTTAATCGCTAAATTTATTACTTTT
TTACTTTTAGGCTTAACCTATAGATAATTAAGATAAATCATGATCGTGAGAATCTAGCTAGCGAGATAGCTGAGCGATAGAAGAAACTCTAGAATTGTGTGATTAAATAAATTTTTCTCTATCTTCTTTTGTATTCAAATA
TTAGTTTTACTTATCTTCAGCTTAATCAAAATTTTATAGAGAATTTATTTGTGGTTCAAAGTATTCTTATTCTTCATGCACATGTGTAGGACTTACGTTATGGCCGTAATATTCGTCGTTAGTTGGGAGTTAGATTCTTGT
TTTTAGTTTAATTGCACTGCTTGGAAGGAATTTTTTGCTTTACCCTCCTAACTAACTTTAACTAGCTGCAAAATGAGTAAAAGTAGTTTTTTGAAAACTTTAAGTACTTTTTTATTGCTTTTATTACTCATGAGGTTATCT
ATAAACTGACTGCAAAATATTTTAATCTTAGTTAGCTTAGTTGAATGTTTTCAATTTTATATTTTAGCTGCTTTCACCTTATTACAAGACTTCCTCAGAGTATTGAGACTTGTTATCGAATGTTTTCAAATTAAATTCCTA
AGAAGATGGCTTCACTGCAAATTTAGGGTTATACATTTTTTAAATATCTGTCTAAGATGGAGGTTAACGAATCTTTGTTTAAGATTTTTCTAAATTGTTAATTGAAATTTAATTTTCACTTCCACTTTTGTTATTTCCTTA
TCCTTAATGCTAACCTGCAATTTTAGCTTAGTTGTTGATTTATTTATTCGAAAATGTAGCATAGTAACCTTATTACATATTTTAGTTAAAAGAAGTATTATTGTTTTCCTGACCTTCTTAATATTATTTTTCATTTACTGC
AGCACCATTACCATTTGCTTAATTTTAATACAAGTCTTCTTAAAATGAAACCTAGTTAAAATGCTCTCCCTTCTATCTTGGTTGATACCCTTAGCTTTTTAAGAATTTGATTTCATCTTAAAGCTTTTGATTTGCTAAACT
CTACTATTATAGACTTTCTTATAGCTATTGAACAGTTTGAAAATTAGCTGTTTAATCATTTTGCATTTTATTTTCCAGATCTGTTTATGCCTTTGCATTTTCATTTTCTTCTTATTCTTTGTTTTTTTTCTCTACTGCTTC
CTTTCTTTGCTGAAGAATCCTAGCAAAGCTATTATCAACATTCTCGATAATTGGTCTACTCGAGGTTGGTACCCAATTCGCCCTATAGTGAGTCGTATTACAATTCACTGGCCGTCGTTTTACAACGTCGTGACTGGGAAA
ACCCTGGCGTTACCCAACTTAATCGCCTTGCAGCACATCCCCCTTTCGCCAGCTGGCGTAATAGCGAAGAGGCCCGCACCGATCGCCCTTCCCAACAGTTGCGCAGCCTGAATGGCGAATGGGACGCGCCCTGTAGCGGCG
CATTAAGCGCGGCGGGTGTGGTGGTTACGCGCAGCGTGACCGCTACACTTGCCAGCGCCCTAGCGCCCGCTCCTTTCGCTTTCTTCCCTTCCTTTCTCGCCACGTTCGCCGGCTTTCCCCGTCAAGCTCTAAATCGGGGGC
TCCCTTTAGGGTTCCGATTTAGTGCTTTACGGCACCTCGACCCCAAAAAACTTGATTAGGGTGATGGTTCACGTAGTGGGCCATCGCCCTGATAGACGGTTTTTCGCCCTTTGACGTTGGAGTCCACGTTCTTTAATAGTG
GACTCTTGTTCCAAACTGGAACAACACTCAACCCTATCTCGGTCTATTCTTTTGATTTATAAGGGATTTTGCCGATTTCGGCCTATTGGTTAAAAAATGAGCTGATTTAACAAAAATTTAACGCGAATTTTAACAAAATAT
TAACGCTTACAATTTAGGTG

PDD1 5' flanking genomic sequence       2244..3700
PDD1 3’ UTR                             3719..3985
loxP                                    3992..4025
neo5                                    4047..5502
loxP                                    5504..5537
PDD1 3' flanking genomic sequence       5544..6967
pBlueScriptSK(+)                        6977..2234
```

Phosphor-mimic *PDD1* genes for in vivo analyses

```
>MIM10
ATGTCTCAGAAAAAGAGTTTAAAATAAAAGAGAAAGTAAGACTATAGCTCTGATGAAGAAGAGGAAGAAGAGGATCAATATGAAGTTGAAAAGATCTTAGACTCTAGATTTAATCCTAAGACCAAGCAAAAAGAATATCTT
GTCAAATGGGAAAGTATTATTAAATATTTATCTATTTATCATATTAATTCTTTTTATATATATATAAAGACTGGCCTATTGAAGACTCTACCTGGGAACCTTACGAGCATCTTTCGAATGTCAAAGAAATAGTTTAGGCCT
TTGAAAAGAAGCAAAAAGCTAACGTTATGCCTCAACCTACTGGACCTATCACTAGAGTAAATGCCTAGAAAGACCCTTAAAAAAAGAACAGACTATCTTTGAATTCTGAAATCAGCAAATCTTTACCTCAAGAAGAAGAAA
TTTAGACATCTAAAGAAGACAGTAAAAAATAAGCAGTCAAAAAATTCCAACCAGCTTCAAGAAGAAAATCTATTAGTTCTTAAGAAGATGAAGACCTTTAAGCTGAAGAAGTTCCACAGTAGCCTGAAAGTAAAAAAGATA
AAAATGATGGTGCCTTTGAAGAACCCAACAATGCTGATGCAGACGAAGAAGAGCTTGTATTTGAAGAAATTGTTGACAAAAGAATTCTTGATGGATAAACAGAATATTTAATACGCTTCTAAAGTAATTTATTTATTTATT
TGTTAATTTTAAATAAACAATTTTAATTAAAATATTTTTAATTTATTTATTTAATTAATTAATTAAAATAGATGTCTCTTAGCCATAATGGGTAGATGTTGGACAATTGATAGCTATTAAAGATGATGTTATAGCTTATGA
AGATAAAATTGCTGCTTAGAGTTAGCTCAATAAGACTAAGAACTTGAAAGAAAATGCTAAACATTCAGAATAACAATAGCAAAGCGAAAATAATAACTTAGAGACGGAAGAAGTTGAAGAAGATAAAGATGAGAAAAAAAG
AAGTTTACTTGCGAATTCAAAGAGACCCACTGCTAAAAGATCATAGCCATTCAATTAAGATGAAGAGAAAGAAAAAGAAGTCACTTAGCCTACCTCGAATTTAGGAGAAAAAGGTCAATCTTAGTAAGTTGAAAAAGAACA
AGCAACAAACTCACAAACTTAACAGCCTCAAACAGCACACAGAAGTGGCTCAAGACTCGAATAGATTCAATCAAATGCCAATCAAGTTACTTAATAAGCTTAATAGCTATCAAACACCAACAATTCTTCCTCTACATCTCT
TGAAGTTTCATCAAAAATGCCTAGCTAAATGTCTTAAAAACGCAGACCTATTGAACTTACTGAAATTTAACAAGGTGATTTCAAGACCGATAATGTCGATAAGATTGAAATCCAAGGAGATTTTAATGACATAATGACTTC
AAGGTTTGAAGTTTTTTGGAAAATCAGATAAGACAATGTTACCCCTGCTTCCTAAGTCTATTCAGCCTCTTACCTGAGAAGATATGAGCCTTAAGTTCTCATCGATTTCCTCTTACAACATTCAAACTAATCTCAATTAAG
ACAAGCTAATTAATAACTTACTCATTGA

Intron    155..210, 688..776


>MIM14
ATGTCTCAGAAAAAGAGTTTAAAATAAAAGAGAAAGTAAGACTATAGCGAAGATGAAGAAGAGGAAGAAGAGGATCAATATGAAGTTGAAAAGATCTTAGACTCTAGATTTAATCCTAAGACCAAGCAAAAAGAATATCTT
GTCAAATGGGAAAGTATTATTAAATATTTATCTATTTATCATATTAATTCTTTTTATATATATATAAAGACTGGCCTATTGAAGACTCTACCTGGGAACCTTACGAGCATCTTTCGAATGTCAAAGAAATAGTTTAGGCCT
TTGAAAAGAAGCAAAAAGCTAACGTTATGCCTCAACCTACTGGACCTATCACTAGAGTAAATGCCTAGAAAGACCCTTAAAAAAAGAACAGACTAGAATTGAATTCTGAAATCGAAAAATCTTTACCTCAAGAAGAAGAAA
TTTAGACATCTAAAGAAGACAGTAAAAAATAAGCAGTCAAAAAATTCCAACCAGCTTCAAGAAGAAAATCTATTAGTTCTTAAGAAGATGAAGACCTTTAAGCTGAAGAAGTTCCACAGTAGCCTGAAAGTAAAAAAGATA
AAAATGATGGTGCCTTTGAAGAACCCAACAATGCTGATGCAGACGAAGAAGAGCTTGTATTTGAAGAAATTGTTGACAAAAGAATTCTTGATGGATAAACAGAATATTTAATACGCTTCTAAAGTAATTTATTTATTTATT
TGTTAATTTTAAATAAACAATTTTAATTAAAATATTTTTAATTTATTTATTTAATTAATTAATTAAAATAGATGTCTCTTAGCCATAATGGGTAGATGTTGGACAATTGATAGCTATTAAAGATGATGTTATAGCTTATGA
AGATAAAATTGCTGCTTAGAGTTAGCTCAATAAGACTAAGAACTTGAAAGAAAATGCTAAACATTCAGAATAACAATAGCAAAGCGAAAATAATAACTTAGAGACGGAAGAAGTTGAAGAAGATAAAGATGAGAAAAAAAG
AAGTTTACTTGCGAATTCAAAGAGACCCACTGCTAAAAGATCATAGCCATTCAATTAAGATGAAGAGAAAGAAAAAGAAGTCACTTAGCCTACCGAAAATTTAGGAGAAAAAGGTCAATCTTAGTAAGTTGAAAAAGAACA
AGCAACAAACTCACAAACTTAACAGCCTCAAACAGCACACAGAAGTGGCTCAAGACTCGAATAGATTCAATCAAATGCCAATCAAGTTACTTAATAAGCTTAATAGCTATCAAACACCAACAATTCTTCCTCTACATCTCT
TGAAGTTTCATCAAAAATGCCTAGCTAAATGTCTTAAAAACGCAGACCTATTGAACTTACTGAAATTTAACAAGGTGATTTCAAGACCGATAATGTCGATAAGATTGAAATCCAAGGAGATTTTAATGACATAATGACTTC
AAGGTTTGAAGTTTTTTGGAAAATCAGATAAGACAATGTTACCCCTGCTTCCTAAGTCTATTCAGCCTCTTACCTGAGAAGATATGAGCCTTAAGTTCTCATCGATTTCCTCTTACAACATTCAAACTAATCTCAATTAAG
ACAAGCTAATTAATAACTTACTCATTGA

Intron    155..210, 688..776


>MIM18
ATGTCTCAGAAAAAGAGTTTAAAATAAAAGAGAAAGTAAGACTATGAAGAAGATGAAGAAGAGGAAGAAGAGGATCAATATGAAGTTGAAAAGATCTTAGACTCTAGATTTAATCCTAAGACCAAGCAAAAAGAATATCTT
GTCAAATGGGAAAGTATTATTAAATATTTATCTATTTATCATATTAATTCTTTTTATATATATATAAAGACTGGCCTATTGAAGACTCTACCTGGGAACCTTACGAGCATCTTTCGAATGTCAAAGAAATAGTTTAGGCCT
TTGAAAAGAAGCAAAAAGCTAACGTTATGCCTCAACCTACTGGACCTATCACTAGAGTAAATGCCTAGAAAGACCCTTAAAAAAAGAACAGACTAGAATTGAATGAGGAAATCGAAAAATCTTTACCTCAAGAAGAAGAAA
TTTAGACATCTAAAGAAGACAGTAAAAAATAAGCAGTCAAAAAATTCCAACCAGCTTCAAGAAGAAAATCTATTAGTGAATAAGAAGATGAAGACCTTTAAGCTGAAGAAGTTCCACAGTAGCCTGAAAGTAAAAAAGATA
AAAATGATGGTGCCTTTGAAGAACCCAACAATGCTGATGCAGACGAAGAAGAGCTTGTATTTGAAGAAATTGTTGACAAAAGAATTCTTGATGGATAAACAGAATATTTAATACGCTTCTAAAGTAATTTATTTATTTATT
TGTTAATTTTAAATAAACAATTTTAATTAAAATATTTTTAATTTATTTATTTAATTAATTAATTAAAATAGATGTCTCTTAGCCATAATGGGTAGATGTTGGACAATTGATAGCTATTAAAGATGATGTTATAGCTTATGA
AGATAAAATTGCTGCTTAGAGTTAGCTCAATAAGGAAAAGAACTTGAAAGAAAATGCTAAACATTCAGAATAACAATAGCAAAGCGAAAATAATAACTTAGAGACGGAAGAAGTTGAAGAAGATAAAGATGAGAAAAAAAG
AAGTTTACTTGCGAATTCAAAGAGACCCACTGCTAAAAGATCATAGCCATTCAATTAAGATGAAGAGAAAGAAAAAGAAGTCACTTAGCCTACCGAAAATTTAGGAGAAAAAGGTCAATCTTAGTAAGTTGAAAAAGAACA
AGCAACAAACTCACAAACTTAACAGCCTCAAACAGCACACAGAAGTGGCTCAAGACTCGAATAGATTCAATCAAATGCCAATCAAGTTACTTAATAAGCTTAATAGCTATCAAACACCAACAATTCTTCCTCTACATCTCT
TGAAGTTTCATCAAAAATGCCTAGCTAAATGTCTTAAAAACGCAGACCTATTGAACTTACTGAAATTTAACAAGGTGATTTCAAGACCGATAATGTCGATAAGATTGAAATCCAAGGAGATTTTAATGACATAATGACTTC
AAGGTTTGAAGTTTTTTGGAAAATCAGATAAGACAATGTTACCCCTGCTTCCTAAGTCTATTCAGCCTCTTACCTGAGAAGATATGAGCCTTAAGTTCTCATCGATTTCCTCTTACAACATTCAAACTAATCTCAATTAAG
ACAAGCTAATTAATAACTTACTCATTGA

Intron    155..210, 688..776


>MIM22
ATGTCTCAGAAAAAGAGTTTAAAATAAAAGAGAAAGTAAGACTATGAAGAAGATGAAGAAGAGGAAGAAGAGGATCAATATGAAGTTGAAAAGATCTTAGACTCTAGATTTAATCCTAAGACCAAGCAAAAAGAATATCTT
GTCAAATGGGAAAGTATTATTAAATATTTATCTATTTATCATATTAATTCTTTTTATATATATATAAAGACTGGCCTATTGAAGACTCTACCTGGGAACCTTACGAGCATCTTTCGAATGTCAAAGAAATAGTTTAGGCCT
TTGAAAAGAAGCAAAAAGCTAACGTTATGCCTCAACCTACTGGACCTATCACTAGAGTAAATGCCTAGAAAGACCCTTAAAAAAAGAACAGACTAGAATTGAATGAGGAAATCGAAAAAGAATTACCTCAAGAAGAAGAAA
TTTAGACATCTAAAGAAGACAGTAAAAAATAAGCAGTCAAAAAATTCCAACCAGCTTCAAGAAGAAAATCTATTGAAGAATAAGAAGATGAAGACCTTTAAGCTGAAGAAGTTCCACAGTAGCCTGAAAGTAAAAAAGATA
AAAATGATGGTGCCTTTGAAGAACCCAACAATGCTGATGCAGACGAAGAAGAGCTTGTATTTGAAGAAATTGTTGACAAAAGAATTCTTGATGGATAAACAGAATATTTAATACGCTTCTAAAGTAATTTATTTATTTATT
TGTTAATTTTAAATAAACAATTTTAATTAAAATATTTTTAATTTATTTATTTAATTAATTAATTAAAATAGATGTCTCTTAGCCATAATGGGTAGATGTTGGACAATTGATAGCTATTAAAGATGATGTTATAGCTTATGA
AGATAAAATTGCTGCTTAGAGTTAGCTCAATAAGGAAAAGAACTTGAAAGAAAATGCTAAACATTCAGAATAACAATAGCAAAGCGAAAATAATAACTTAGAGACGGAAGAAGTTGAAGAAGATAAAGATGAGAAAAAAAG
AAGTTTACTTGCGAATGAAAAGAGACCCACTGCTAAAAGATCATAGCCATTCAATTAAGATGAAGAGAAAGAAAAAGAAGTCACTTAGCCTACCGAAAATTTAGGAGAAAAAGGTCAATCTTAGTAAGTTGAAAAAGAACA
AGCAACAAACTCACAAACTTAACAGCCTCAAACAGCACACAGAAGTGGCGAAAGACTCGAATAGATTCAATCAAATGCCAATCAAGTTACTTAATAAGCTTAATAGCTATCAAACACCAACAATTCTTCCTCTACATCTCT
TGAAGTTTCATCAAAAATGCCTAGCTAAATGTCTTAAAAACGCAGACCTATTGAACTTACTGAAATTTAACAAGGTGATTTCAAGACCGATAATGTCGATAAGATTGAAATCCAAGGAGATTTTAATGACATAATGACTTC
AAGGTTTGAAGTTTTTTGGAAAATCAGATAAGACAATGTTACCCCTGCTTCCTAAGTCTATTCAGCCTCTTACCTGAGAAGATATGAGCCTTAAGTTCTCATCGATTTCCTCTTACAACATTCAAACTAATCTCAATTAAG
ACAAGCTAATTAATAACTTACTCATTGA

Intron    155..210, 688..776


>MIM22+Ins6K
ATGTCTCAGAAAAAGAGTTTAAAATAAAAGAGAAAGTAAGACTATGAAGAAGATGAAGAAGAGGAAGAAGAGGATCAATATGAAGTTGAAAAGATCTTAGACTCTAGATTTAATCCTAAGACCAAGCAAAAAGAATATCTT
GTCAAATGGGAAAGTATTATTAAATATTTATCTATTTATCATATTAATTCTTTTTATATATATATAAAGACTGGCCTATTGAAGACTCTACCTGGGAACCTTACGAGCATCTTTCGAATGTCAAAGAAATAGTTTAGGCCT
TTGAAAAGAAGCAAAAAGCTAACGTTATGCCTCAACCTACTGGACCTATCACTAGAGTAAATGCCTAGAAAGACCCTTAAAAAAAGAACAGACTAGAATTGAATGAGGAAATCGAAAAAAAGGAATTACCTCAAGAAGAAG
AAATTTAGACATCTAAAGAAGACAGTAAAAAATAAGCAGTCAAAAAATTCCAACCAGCTTCAAGAAGAAAATCTATTGAAAAGGAATAAGAAGATGAAGACCTTTAAGCTGAAGAAGTTCCACAGTAGCCTGAAAGTAAAA
AAGATAAAAATGATGGTGCCTTTGAAGAACCCAACAATGCTGATGCAGACGAAGAAGAGCTTGTATTTGAAGAAATTGTTGACAAAAGAATTCTTGATGGATAAACAGAATATTTAATACGCTTCTAAAGTAATTTATTTA
TTTATTTGTTAATTTTAAATAAACAATTTTAATTAAAATATTTTTAATTTATTTATTTAATTAATTAATTAAAATAGATGTCTCTTAGCCATAATGGGTAGATGTTGGACAATTGATAGCTATTAAAGATGATGTTATAGC
TTATGAAGATAAAATTGCTGCTTAGAGTTAGCTCAATAAGGAAAAGAAGAACTTGAAAGAAAATGCTAAACATTCAGAATAACAATAGCAAAGCGAAAATAATAACTTAGAGACGGAAGAAGTTGAAGAAGATAAAGATGA
GAAAAAAAGAAGTTTACTTGCGAATAAGGAAAAGAGACCCACTGCTAAAAGATCATAGCCATTCAATTAAGATGAAGAGAAAGAAAAAGAAGTCACTTAGCCTACCGAAAAGAATTTAGGAGAAAAAGGTCAATCTTAGTA
AGTTGAAAAAGAACAAGCAACAAACTCACAAACTTAACAGCCTCAAACAGCACACAGAAGTGGCGAAAAGAGACTCGAATAGATTCAATCAAATGCCAATCAAGTTACTTAATAAGCTTAATAGCTATCAAACACCAACAA
TTCTTCCTCTACATCTCTTGAAGTTTCATCAAAAATGCCTAGCTAAATGTCTTAAAAACGCAGACCTATTGAACTTACTGAAATTTAACAAGGTGATTTCAAGACCGATAATGTCGATAAGATTGAAATCCAAGGAGATTT
TAATGACATAATGACTTCAAGGTTTGAAGTTTTTTGGAAAATCAGATAAGACAATGTTACCCCTGCTTCCTAAGTCTATTCAGCCTCTTACCTGAGAAGATATGAGCCTTAAGTTCTCATCGATTTCCTCTTACAACATTC
AAACTAATCTCAATTAAGACAAGCTAATTAATAACTTACTCATTGA

Intron    155..210, 694..782


>MIM22+Sub6K
ATGTCTCAGAAAAAGAGTTTAAAATAAAAGAGAAAGTAAGACTATGAAGAAGATGAAGAAGAGGAAGAAGAGGATCAATATGAAGTTGAAAAGATCTTAGACTCTAGATTTAATCCTAAGACCAAGCAAAAAGAATATCTT
GTCAAATGGGAAAGTATTATTAAATATTTATCTATTTATCATATTAATTCTTTTTATATATATATAAAGACTGGCCTATTGAAGACTCTACCTGGGAACCTTACGAGCATCTTTCGAATGTCAAAGAAATAGTTTAGGCCT
```

TTGAAAAGAAGCAAAAAGCTAACGTTATGCCTCAACCTACTGGACCTATCACTAGAGTAAATGCCTAGAAAGACCCTTAAAAAAAGAACAGACTAGAATTGAATGAGGAAATCGAAAAAGAATTACCTAAAGAAGAAGAAA
TTTAGACATCTAAAGAAGACAGTAAAAAATAAGCAGTCAAAAAATTCCAACCAGCTTCAAGAAGAAAATCTATTGAAGAAAAAGAAGATGAAGACCTTTAAGCTGAAGAAGTTCCACAGTAGCCTGAAAGTAAAAAAGATA
AAAATGATGGTGCCTTTGAAGAACCCAACAATGCTGATGCAGACGAAGAAGAGCTTGTATTTGAAGAAATTGTTGACAAAAGAATTCTTGATGGATAAACAGAATATTTAATACGCTTCTAAAGTAATTTATTTATTTATT
TGTTAATTTTAAATAAACAATTTTAATTAAAATATTTTTAATTTATTTATTTAATTAATTAATTAAAATAGATGTCTCTTAGCCATAATGGGTAGATGTTGGACAATTGATAGCTATTAAAGATGATGTTATAGCTTATGA
AGATAAAATTGCTGCTTAGAGTTAGCTCAAGAAGGAAAAGAACTTGAAAGAAAATGCTAAACATTCAGAATAACAATAGCAAAGCGAAAATAATAACTTAGAGACGGAAGAAGTTGAAGAAGATAAAGATGAGAAAAAAAG
AAGTTTACTTGCGAAGGAAAAGAGACCCACTGCTAAAAGATCATAGCCATTCAATTAAGATGAAGAGAAAGAAAAAGAAGTCACTTAGCCTACCGAAAAGTTAGGAGAAAAAGGTCAATCTTAGTAAGTTGAAAAAGAACA
AGCAACAAACTCACAAACTTAACAGCCTCAAACAGCACACAGAAGTGGCGAAAGACTCGAAAAGATTCAATCAAATGCCAATCAAGTTACTTAATAAGCTTAATAGCTATCAAACACCAACAATTCTTCCTCTACATCTCT
TGAAGTTTCATCAAAAATGCCTAGCTAAATGTCTTAAAAACGCAGACCTATTGAACTTACTGAAATTTAACAAGGTGATTTCAAGACCGATAATGTCGATAAGATTGAAATCCAAGGAGATTTTAATGACATAATGACTTC
AAGGTTTGAAGTTTTTTGGAAAATCAGATAAGACAATGTTACCCCTGCTTCCTAAGTCTATTCAGCCTCTTACCTGAGAAGATATGAGCCTTAAGTTCTCATCGATTTCCTCTTACAACATTCAAACTAATCTCAATTAAG
ACAAGCTAATTAATAACTTACTCATTGA

Intron   155..210, 688..776

Codon optimized *PDD1* genes for *E. coli* expression

>opt.WT_FL
ATGAGCCAAAAAAAATCCCTGAAACAAAAACGTAAACAAGACTATAGCAGCGACACCGAGGAAGAGGAGGAGGACCAATATGAAGTGGAAAAAATCCTGGATAGCCGCTTTAATCCGAAAACCAAACAAAAAGAGTATCTG
GTGAAATGGGAGAACTGGCCGATCGAAGATTCAACCTGGGAACCGTATGAACATCTGAGCAACGTGAAAGAAATCGTACAGGCCTTTGAGAAAAAACAGAAAGCCAACGTGATGCCTCAACCAACAGGTCCGATTACCCGT
GTTAATGCCCAGAAAGACCCACAGAAAAAAAACCGTCTGAGCCTGAATAGCAGTATCAGCAAATCTCTGCCACAAGAAGAGGAAATCCAAACCAGCAAAGAGGATAGCAAAAAACAAGCCGTCAAAAAATTCCAGCCGGCA
AGTCGTCGTAAAAGCATCTCTAGTCAGAGCGATAGTGATCTGCAAGCAGAAGAAGTGCCTCAGCAGCCAGAGTCTAAAAAAGACAAAAATGATGGCGCCTTTGAAGAACCGAATAACGCCGATGCCGATGAAACGGAACTG
GTGTTCGAGGAGATTGTGGATAAACGTATTCTGGATGGCCAGACCGAATATCTGATCCGCTTTCAGAACGTTAGTCAGCCACAGTGGGTTGATGTTGGCCAGCTGATTGCCATCAAAGATGACGTGATCGCCTATGAGGAC
AAAATCGCCGCCCAAAGTCAACTGAACAAAACGAAAAACCTGAAAGAGAACGCCAAACATTCTAGTCAACAGCAGCAGTCCGAGAACAATAATCTGGAGACTGAAGAGGTCGAAGAGGACAAAGACAGCAAAAAACGCTCC
CTGCTGGCAAATTCTAAACGCCCGACGGCGAAACGCTCTCAACCGTTCAACCAGGACGAGGAAAAAGAGAAATCCGTCACCCAGCCTACAAGCAACCTGGGATCGAAAGGTCAGAGTCAGCAAGTGGAGAAAGAACAAGCG
ACCAATAGCCAGACACAACAGCCACAGACAGCACATCGTAGCGGTAGTCGTCTGAGTCAAATCCAATCGAATGCTAACCAAGTGACACAACAGGCCCAACAACTGAGTAATACCAACAACAGCTCCAGCACTAGTCTGGAA
GTGTCCAGTAAAATGCCGTCACAGATGAGTCAGAAACGTCGCCCGATTGAACTGACTGAAATTCAACAGGGGGACTTTAAAACCGACAACGTGGATAAAATCGAGATCCAAGGCGACTTCAACGATATCATGACCTCCCGC
TTCGAAGTGTTTTGGAAAATCCGCCAGGACAATGTTACACCGGCAAGCCAGGTGTATTCTGCCTCTTATCTGCGTCGCTATGAACCACAAGTCCTGATCGACTTTCTGCTGCAACACTCCAATCAGTCACAGCTGCGCCAG
GCAAATCAGCAACTGACCCATTGA

>opt.W50/53A_FL
ATGAGCCAAAAAAAATCCCTGAAACAAAAACGTAAACAAGACTATAGCAGCGACACCGAGGAAGAGGAGGAGGACCAATATGAAGTGGAAAAAATCCTGGATAGCCGCTTTAATCCGAAAACCAAACAAAAAGAGTATCTG
GTGAAAGCCGAGAACGCCCCGATCGAAGATTCAACCTGGGAACCGTATGAACATCTGAGCAACGTGAAAGAAATCGTACAGGCCTTTGAGAAAAAACAGAAAGCCAACGTGATGCCTCAACCAACAGGTCCGATTACCCGT
GTTAATGCCCAGAAAGACCCACAGAAAAAAAACCGTCTGAGCCTGAATAGCAGTATCAGCAAATCTCTGCCACAAGAAGAGGAAATCCAAACCAGCAAAGAGGATAGCAAAAAACAAGCCGTCAAAAAATTCCAGCCGGCA
AGTCGTCGTAAAAGCATCTCTAGTCAGAGCGATAGTGATCTGCAAGCAGAAGAAGTGCCTCAGCAGCCAGAGTCTAAAAAAGACAAAAATGATGGCGCCTTTGAAGAACCGAATAACGCCGATGCCGATGAAACGGAACTG
GTGTTCGAGGAGATTGTGGATAAACGTATTCTGGATGGCCAGACCGAATATCTGATCCGCTTTCAGAACGTTAGTCAGCCACAGTGGGTTGATGTTGGCCAGCTGATTGCCATCAAAGATGACGTGATCGCCTATGAGGAC
AAAATCGCCGCCCAAAGTCAACTGAACAAAACGAAAAACCTGAAAGAGAACGCCAAACATTCTAGTCAACAGCAGCAGTCCGAGAACAATAATCTGGAGACTGAAGAGGTCGAAGAGGACAAAGACAGCAAAAAACGCTCC
CTGCTGGCAAATTCTAAACGCCCGACGGCGAAACGCTCTCAACCGTTCAACCAGGACGAGGAAAAAGAGAAATCCGTCACCCAGCCTACAAGCAACCTGGGATCGAAAGGTCAGAGTCAGCAAGTGGAGAAAGAACAAGCG
ACCAATAGCCAGACACAACAGCCACAGACAGCACATCGTAGCGGTAGTCGTCTGAGTCAAATCCAATCGAATGCTAACCAAGTGACACAACAGGCCCAACAACTGAGTAATACCAACAACAGCTCCAGCACTAGTCTGGAA
GTGTCCAGTAAAATGCCGTCACAGATGAGTCAGAAACGTCGCCCGATTGAACTGACTGAAATTCAACAGGGGGACTTTAAAACCGACAACGTGGATAAAATCGAGATCCAAGGCGACTTCAACGATATCATGACCTCCCGC
TTCGAAGTGTTTTGGAAAATCCGCCAGGACAATGTTACACCGGCAAGCCAGGTGTATTCTGCCTCTTATCTGCGTCGCTATGAACCACAAGTCCTGATCGACTTTCTGCTGCAACACTCCAATCAGTCACAGCTGCGCCAG
GCAAATCAGCAACTGACCCATTGA

>opt.I456D_FL
ATGAGCCAAAAAAAATCCCTGAAACAAAAACGTAAACAAGACTATAGCAGCGACACCGAGGAAGAGGAGGAGGACCAATATGAAGTGGAAAAAATCCTGGATAGCCGCTTTAATCCGAAAACCAAACAAAAAGAGTATCTG
GTGAAATGGGAGAACTGGCCGATCGAAGATTCAACCTGGGAACCGTATGAACATCTGAGCAACGTGAAAGAAATCGTACAGGCCTTTGAGAAAAAACAGAAAGCCAACGTGATGCCTCAACCAACAGGTCCGATTACCCGT
GTTAATGCCCAGAAAGACCCACAGAAAAAAAACCGTCTGAGCCTGAATAGCAGTATCAGCAAATCTCTGCCACAAGAAGAGGAAATCCAAACCAGCAAAGAGGATAGCAAAAAACAAGCCGTCAAAAAATTCCAGCCGGCA
AGTCGTCGTAAAAGCATCTCTAGTCAGAGCGATAGTGATCTGCAAGCAGAAGAAGTGCCTCAGCAGCCAGAGTCTAAAAAAGACAAAAATGATGGCGCCTTTGAAGAACCGAATAACGCCGATGCCGATGAAACGGAACTG
GTGTTCGAGGAGATTGTGGATAAACGTATTCTGGATGGCCAGACCGAATATCTGATCCGCTTTCAGAACGTTAGTCAGCCACAGTGGGTTGATGTTGGCCAGCTGATTGCCATCAAAGATGACGTGATCGCCTATGAGGAC
AAAATCGCCGCCCAAAGTCAACTGAACAAAACGAAAAACCTGAAAGAGAACGCCAAACATTCTAGTCAACAGCAGCAGTCCGAGAACAATAATCTGGAGACTGAAGAGGTCGAAGAGGACAAAGACAGCAAAAAACGCTCC
CTGCTGGCAAATTCTAAACGCCCGACGGCGAAACGCTCTCAACCGTTCAACCAGGACGAGGAAAAAGAGAAATCCGTCACCCAGCCTACAAGCAACCTGGGATCGAAAGGTCAGAGTCAGCAAGTGGAGAAAGAACAAGCG
ACCAATAGCCAGACACAACAGCCACAGACAGCACATCGTAGCGGTAGTCGTCTGAGTCAAATCCAATCGAATGCTAACCAAGTGACACAACAGGCCCAACAACTGAGTAATACCAACAACAGCTCCAGCACTAGTCTGGAA
GTGTCCAGTAAAATGCCGTCACAGATGAGTCAGAAACGTCGCCCGATTGAACTGACTGAAATTCAACAGGGGGACTTTAAAACCGACAACGTGGATAAAATCGAGATCCAAGGCGACTTCAACGATATCATGACCTCCCGC
TTCGAAGTGTTTTGGAAAATCCGCCAGGACAATGTTACACCGGCAAGCCAGGTGTATTCTGCCTCTTATCTGCGTCGCTATGAACCACAAGTCCTGGACGACTTTCTGCTGCAACACTCCAATCAGTCACAGCTGCGCCAG
GCAAATCAGCAACTGACCCATTGA

>opt.MIM14_FL
ATGTCTCAAAAAAAAAGCCTGAAGCAAAAACGCAAACAGGATTATAGCGAAGATGAGGAAGAGGAAGAAGAAGACCAGTATGAAGTGGAGAAAATCCTCGATTCACGGTTTAACCCCAAAACAAAACAGAAAGAGTATCTG
GTGAAGTGGGAAAACTGGCCCATTGAGGATAGCACATGGGAGCCATACGAACATTTATCTAATGTGAAGGAGATTGTGCAGGCGTTCGAAAAAAAACAAAAAGCGAATGTTATGCCCCAGCCCACGGGTCCGATTACGCGC
GTTAATGCGCAAAAGGACCCACAGAAGAAGAATCGCCTCGAACTCAACTCGGAGATTGAAAAATCACTCCCCCAAGAAGAAGAAATTCAGACGAGTAAAGAGGATTCTAAAAAACAGGCCGTCAAAAAGTTCCAGCCGGCT
TCGCGGCGCAAGAGTATTAGCTCCCAGGAAGATGAGGACCTGCAAGCAGAAGAAGTTCCGCAGCAGCCGGAAAGTAAAAAGGATAAAAATGATGGCGCATTTGAAGAACCTAACAACGCTGATGCAGACGAAGAGGAACTG
GTGTTCGAGGAAATTGTGGATAAACGCATTTTGGACGGTCAGACTGAGTACTTGATCCGTTTTCAGAATGTCAGCCAACCTCAGTGGGTTGACGTTGGTCAGCTGATTGCTATCAAAGACGATGTGATCGCGTACGAAGAT
AAAATCGCCGCACAGTCACAGCTGAATAAAACAAAAAACTTGAAGGAAAACGCTAAACATTCGGAGCAGCAGCAGCAGTCCGAAAACAACAATTTGGAAACTGAAGAAGTGGAGGAAGACAAAGATGAAAAGAAGCGCAGT
CTGCTGGCGAATAGCAAACGTCCGACTGCGAAGCGTTCACAACCGTTCAATCAGGATGAAGAAAAAGAAAAAGAAGTGACCCAGCCAACCGAAAATCTCGGCGAAAAAGGCCAATCGCAGCAGGTGGAAAAAGAACAGGCC
ACGAATAGCCAGACTCAGCAGCCGCAGACCGCCCATCGCAGTGGCTCGCGCCTTGAACAGATCCAATCGAACGCGAATCAGGTAACGCAGCAGGCCCAGCAGTTAAGCAACACCAATAATAGCTCTTCTACCAGCCTGGAG
GTTAGCTCAAAAATGCCTAGTCAGATGTCTCAGAAACGCCGCCCGATCGAACTGACCGAGATTCAGCAGGGGGATTTCAAGACGGACAATGTGGACAAAATTGAGATTCAGGGTGATTTCAATGATATCATGACGTCTCGC
TTTGAAGTTTTTTGGAAAATTCGCCAGGATAATGTAACGCCAGCGAGCCAAGTGTACAGTGCCAGCTACCTGCGCCGTTACGAACCTCAGGTCCTGATCGATTTCCTGTTACAACACTCTAATCAGTCTCAGCTGCGCCAG
GCGAATCAGCAACTTACGCACTGA

>opt.MIM22_FL
ATGTCTCAAAAAAAAAGCCTGAAACAAAAACGCAAGCAAGATTATGAAGAAGATGAAGAGGAAGAAGAGGAAGACCAGTATGAGGTGGAAAAAATCCTGGATAGTCGTTTCAATCCGAAAACAAAGCAGAAAGAATATCTT
GTGAAATGGGAAAATTGGCCCATCGAAGATAGCACGTGGGAACCTTACGAACATCTGTCGAATGTTAAAGAAATCGTCCAGGCTTTTGAAAAAAAACAGAAAGCCAATGTGATGCCTCAGCCTACCGGACCGATCACACGT
GTGAACGCGCAGAAAGATCCTCAGAAGAAAAATCGCCTGGAGCTGAATGAAGAAATCGAGAAAGAACTGCCGCAGGAGGAGGAAATTCAGACCAGCAAAGAAGATTCGAAAAAGCAGGCTGTGAAAAAGTTTCAACCGGCA
AGCCGTCGCAAAAGTATTGAAGAACAGGAAGATGAGGATTTGCAGGCGGAGGAAGTGCCCCAGCAACCTGAATCCAAAAAAGATAAAAACGATGGAGCGTTTGAAGAGCCAAACAATGCGGACGCAGACGAAGAGGAACTC
GTGTTCGAGGAGATTGTGGACAAACGCATTTTAGATGGTCAGACCGAATACCTCATTCGTTTTCAGAACGTTAGTCAGCCGCAATGGGTAGACGTAGGGCAGTTAATTGCCATTAAAGATGACGTGATCGCGTATGAAGAT
AAAATTGCCGCCCAAAGTCAGCTGAACAAGGAAAAAAACTTAAAAGAGAATGCTAAGCACAGCGAACAACAACAACAAAGCGAAAATAACAACCTCGAAACAGAGGAAGTGGAAGAAGATAAAGATGAAAAAAAACGCTCC
TTACTGGCAAACGAAAAACGCCCGACCGCGAAACGCTCCCAGCCCTTTAATCAGGACGAAGAGAAGGAGAAGGAAGTCACACAGCCCACGGAGAATTTAGGCGAAAAAGGTCAGAGCCAGCAGGTAGAAAAAGAACAGGCT
ACCAATTCTCAGACGCAGCAGCCGCAAACCGCCCACCGTTCCGGCGAACGTCTGGAGCAGATCCAGAGCAACGCTAATCAAGTTACGCAGCAGGCGCAGCAACTGTCTAATACCAACAATAGCAGCAGTACTTCGCTCGAA
GTGTCCAGCAAGATGCCGTCCCAGATGTCTCAAAAACGTCGTCCAATTGAACTTACGGAGATCCAACAGGGTGACTTTAAGACGGACAATGTGGATAAGATTGAAATTCAGGGGGATTTTAATGATATCATGACCTCTCGT
TTCGAGGTTTTTTGGAAAATCCGCCAAGACAATGTCACCCCCGCGTCGCAGGTTTACTCAGCCTCGTATCTTCGCCGTTACGAACCGCAAGTGCTTATTGATTTTCTGCTCCAGCACTCAAATCAATCGCAGCTTCGCCAA
GCCAACCAGCAGTTGACGCACTGA

>opt.MIM22+Ins6K_FL
ATGTCCCAAAAGAAATCGTTAAAGCAGAAACGTAAGCAGGATTATGAGGAAGATGAAGAAGAAGAAGAAGAGGACCAGTACGAAGTCGAGAAGATCCTGGATTCCCGCTTCAACCCGAAAACTAAGCAAAAAGAGTACCTG
GTTAAATGGGAAAACTGGCCAATTGAGGATTCGACTTGGGAACCGTATGAACATTTGTCAAATGTGAAAGAGATTGTACAGGCGTTTGAGAAAAAACAAAAAGCGAACGTTATGCCGCAGCCGACGGGCCCCATTACTCGT
GTAAATGCGCAGAAAGATCCACAGAAGAAAAATCGCCTGGAGCTGAATGAAGAGATCGAAAAAAAAGAATTACCTCAGGAAGAAGAAATTCAAACGAGCAAGGAAGACTCAAAAAAACAGGCCGTGAAGAAATTTCAGCCT
GCGAGCCGCCGTAAAAGTATCGAAAAAGAACAAGAAGATGAAGACCTGCAAGCCGAGGAAGTTCCGCAACAGCCGGAATCCAAGAAAGATAAAAATGACGGCGCCTTTGAAGAACCAAATAATGCCGATGCGGATGAGGAG
GAACTTGTGTTCGAAGAAATCGTTGATAAACGTATCCTGGACGGGCAAACGGAATATCTGATCCGCTTTCAGAATGTCTCTCAACCGCAGTGGGTGGACGTTGGTCAATTGATTGCCATTAAAGACGATGTCATTGCGTAT
GAGGATAAAATCGCCGCGCAATCGCAGCTGAACAAAGAAAAGAAGAACCTGAAGGAAAACGCTAAACACTCTGAACAACAGCAGCAGTCAGAGAATAACAATCTCGAAACCGAGGAAGTAGAAGAAGATAAAGATGAAAAA
AAACGGTCGCTCCTGGCTAATAAAGAAAAGCGCCCGACAGCAAAACGGAGCCAGCCTTTCAACCAGGATGAAGAAAAAGAAAAAGAAGTGACGCAGCCGACAGAAAAAAACCTGGGCGAAAAAGGACAATCGCAGCAGGTG
GAGAAAGAGCAGGCGACGAACAGTCAAACTCAGCAGCCTCAGACCGCACACCGCAGTGGAGAGAAGCGTCTGGAACAAATCCAAAGTAATGCAAATCAAGTGACCCAACAGGCTCAACAACTGAGCAATACGAACAATAGC
AGCTCCACCTCGTTGGAGGTCAGCTCAAAGATGCCTAGCCAGATGAGCCAAAAGCGTCGCCCGATTGAGCTGACCGAAATTCAGCAGGGTGATTTTAAGACCGACAATGTAGACAAGATCGAAATTCAGGGAGATTTTAAC
GATATTATGACTTCACGGTTCGAGGTCTTCTGGAAAATCCGTCAAGATAATGTAACCCCGGCCTCTCAGGTTTATTCGGCCAGCTATCTTCGTCGTTATGAACCCCAGGTCCTCATCGATTTTCTGCTCCAGCACTCGAAT
CAGTCTCAACTTCGCCAGGCCAACCAGCAGCTTACCCATTGA

>opt. WT_HNG1
AAAAAACAGAAAGCCAACGTGATGCCTCAACCAACAGGTCCGATTACCCGTGTTAATGCCCAGAAAGACCCACAGAAAAAAAACCGTCTGAGCCTGAATAGCAGTATCAGCAAATCTCTGCCACAAGAAGAGGAAATCCAA
ACCAGCAAAGAGGATAGCAAAAAACAAGCCGTCAAAAAATTCCAGCCGGCAAGTCGTCGTAAAAGCATCTCTAGTCAGAGCGATAGTGATCTGCAAGCAGAAGAAGTGCCTCAGCAGCCAGAGTCTAAAAAAGACAAAAAT
GATGGCGCCTTTGAAGAACCGAATAACGCCGATGCCGATGAAACGGAACTGTGA

>opt.WT_HNG2
ATCAAAGATGACGTGATCGCCTATGAGGACAAAATCGCCGCCCAAAGTCAACTGAACAAAACGAAAAACCTGAAAGAGAACGCCAAACATTCTAGTCAACAGCAGCAGTCCGAGAACAATAATCTGGAGACTGAAGAGGTC
GAAGAGGACAAAGACAGCAAAAAACGCTCCCTGCTGGCAAATTCTAAACGCCCGACGGCGAAACGCTCTCAACCGTTCAACCAGGACGAGGAAAAAGAGAAATCCGTCACCCAGCCTACAAGCAACCTGGGATCGAAAGGT
CAGAGTCAGCAAGTGGAGAAAGAACAAGCGACCAATAGCCAGACACAACAGCCACAGACAGCACATCGTAGCGGTAGTCGTCTGAGTCAAATCCAATCGAATGCTAACCAAGTGACACAACAGGCCCAACAACTGAGTAAT
ACCAACAACAGCTCCAGCACTAGTCTGGAAGTGTCCAGTAAAATGCCGTCACAGATGAGTCAGAAACGTCGCCCGATTGAACTGACTGAAATTTGA

A map and the sequence of pCAM_MDS-IES

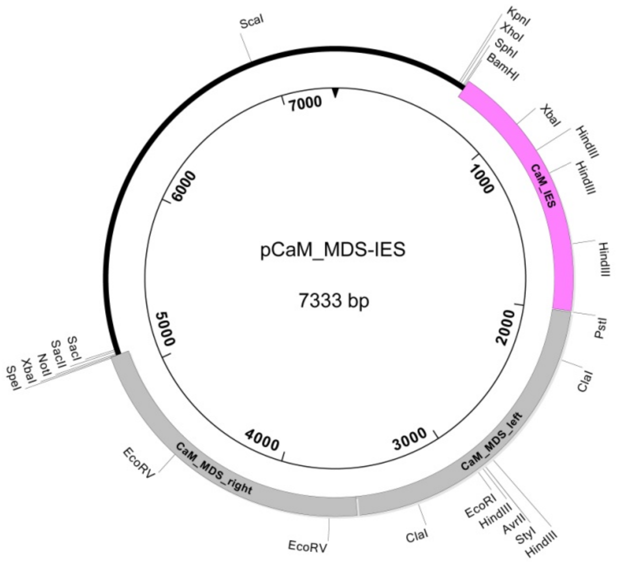


```
> pCAM_MDS-IES
CACCTAAATTGTAAGCGTTAATATTTTGTTAAAATTCGCGTTAAATTTTTGTTAAATCAGCTCATTTTTTAACCAATAGGCCGAAATCGGCAAAATCCCTTATAAATCAAAAGAATAGACCGAGATAGGGTTGAGTGTTGT
TCCAGTTTGGAACAAGAGTCCACTATTAAAGAACGTGGACTCCAACGTCAAAGGGCGAAAAACCGTCTATCAGGGCGATGGCCCACTACGTGAACCATCACCCTAATCAAGTTTTTTGGGGTCGAGGTGCCGTAAAGCACT
AAATCGGAACCCTAAAGGGAGCCCCCGATTTAGAGCTTGACGGGGAAAGCCGGCGAACGTGGCGAGAAAGGAAGGGAAGAAAGCGAAAGGAGCGGGCGCTAGGGCGCTGGCAAGTGTAGCGGTCACGCTGCGCGTAACCAC
CACACCCGCCGCGCTTAATGCGCCGCTACAGGGCGCGTCCCATTCGCCATTCAGGCTGCGCAACTGTTGGGAAGGGCGATCGGTGCGGGCCTCTTCGCTATTACGCCAGCTGGCGAAAGGGGGATGTGCTGCAAGGCGATT
AAGTTGGGTAACGCCAGGGTTTTCCCAGTCACGACGTTGTAAAACGACGGCCAGTGAATTGTAATACGACTCACTATAGGGCGAATTGGGTACCGGGCCCCCCCTCGAGACGACGCATGCCTACAGGATCCAATTTACAAA
AGTTTGAATAATGAATTTTAGCACATTTAAATATTATTATATCAGTAAATAAAATATTCTTAAATTCTATAAAATATTTCTTATATATTTTTAAATATTACCAAATCAAAAACTAATATTTTTTAACAATTCAAATTAAAA
AAAAAATTAATTGTTTTAATTTTATGATTTACTAAATTTCAATTTTTAATTTCTTAACCTTAGATGTTTTAAAATAAGATAGATTTCTTCATTAAAAAAATTTGATTAATTTACTAGGCACTAAAATTTTTTTATTTTTAAA
AAAACTATTTTTAAAATAGATTTTTCTAGATTTAAATTACAAATAAAGATAATTTTTTTAATCACAAATGTTCAATTAATAGAGTATTTTTATGAACTTAATTTAATTTTATTATAGCACTTTGTAATTTAAAAAAATAGT
CATCGCTATTTATTAGCTATAATTTATTTTCTGATATATTTTGAAGCTTTAAATAAAAAATAGTATGTCATCAAAATATTTATAAATTTTGCCGAAAAATAAAAAACATTGATCTAAATTTTCAAAATTTATGGTTTTCCA
GAATTTTCAATCTAAAAAATTCTGGAAATTAAGCTTCATTAATAATTGAATGAAAACATAAATTTTATGTTTAATTAAATTTTTCTTTTTAAATATAGGAAATGCTTCCAAATTTTATACTTTGAGTAGAGTTTTTGTAAT
TTTTATTAATTTTTTTGAATCCATGCACTATTAAAAATCTAAATAAATTATCATTATTTTTTTTTTTTTTTTCTTTTTATAACTCTATTTTAAATTAATATTAAATTTTATTCTTCTTATTGATAAAATTATTTGATTGAT
AGTTCATTATCAGCCATCAAGCATTTTTAAAATAGCCAATTGCTTTAATTTTATAATTTTAAAAACATATTATTAAAAATAGTATGTATTTCTTTTTACAGTTAAGCATACTAAGCTTTATTTTAATATTAAATTTGTAAG
CAATCCTATTTAGAGAACAGTTAGTTGATAAAAATCAGTTAGGCTTTAGTTTTAAATTATTTAAGAAACATTTGCATTATAAAAACTTCTTTAAAAATTTTATTTTTAACCAAAATTTTTTTTTATGAAATGAAATATTTA
TAAAATCTTAATTTAATTAAATTCAATTTTGCATATTTTGATTAAATGAGATATTTATATGTTGTATAGCTTTATATTATGTGTTTTAACTGTATCAATCAATAAATAAATGAATTAAATTGAAATAACTTGAATCAAATT
GAAAATAAATCTTAAAATAACTGCAGCGAAGTTAGAGCGAACCTCATAGGTGACGGATTTTAGAAGACCCCAAAAAGAAACGGATGTAATACCAATTGATTGATTGATTGATTGATTGTTTGATTTATTTATTTATTTATT
TTAAATATATTTAAAAATCATAATGAATAGATGTTAGAATGAATGTTATTGGAGAGATTTCTCAAGCTATTAACTTTGACAATGATAACCTGTATGTATAAATTAATGATAAATTAACAGGATCGATCAGCTATATTTTTAT
TTATTTATCCACTATAGTTATATTGAATGGGAGTTTTTTTTACCTCCTCACTGGTAGCCAGATTATGAGGATTTGCAAGAAAATATGCCAATTTTATCAGGACACATTAGCGAAGATATGAAAAGGGATTGGAATTTTTTG
TAAATATCTAAATTTAATTAATTAGCTATTTCATAATTGTTAAATATATTTAATTAGCTCATCCATAACCCAAATAGCTTCTCCTATTTAATTAAAAAACAAAGATGGTTAAAACGAACTCATAACTAACTTCTGCTTCCC
ATTTGATATTTAGGTTTAAACTAAACAATAAACATGTAAATCTATCTTATTAATTATTTTAGAGTATTAAAGAAATTAATTATATTTATTCAAATATTAGTTGAAGAATTATCCAAATTCCCTATATTATTCCTTACATTA
AGTTCAATAGATTCATGGGGTAGAAAAACCCTTTAAGGTAAATATATGAATTATTCTAATTGCATTTATCAATGTTATTTTTTCAATTATCATTAAGGATACACATATTGTGAAGTTCCAAGAAAACCAGGATACCATGAA
ATAACTGTTAAAGCTTGGGCTCCTGCTTAAAGTATTTACTAAAAAGTACATGAGTTTTTCCTAGGAGGACAAACTAAGCTTTATGATATAAAAAAAATAGCTTAGAATTCAATTTTAAATGATGAAGTAAATAAATATCTT
TTAGATAATTAAAAAGCTCTAAATAAATATTAAATACTAACTATTAACAGAATACATAAAGCGCTATTGATAGATTTAATCTAACTACTGAGAGTAAAGGAGCTGTTAAATTAATATTTAATGTTGCATTTTAGTCAAAGT
AATTAATTATAAATGATGTCAAAAACAAATATTTAGTTAGTTATTAAATCATGTTTAAATTAGAAAACGTAAAGAAGAATAAACTGCCATTTACCATGCAAAAAGATTAAATCTCAAAAGTAATAATAAAAGTTAATAGA
TCCATCTGAAATCGATTAGCAAGGATAACAAGATAACTCTTATGCACCTGTTATAAAGTAATATTTTTTATCAAGTTAATATATTTTAATTACAATAATTAAAAAGAAACGAGTATACAACTTCACTAAGATATACTACGT
AGAAATTAAATTGATATTTATGATTATGCTTAAACAATTAAATTGTTTTATCAACTAATTAACTAACTAACTAACTTATTCTATTAAAATTCTTTAAAATTATTGTATATTATTGTAAAATTTATTTATTTATCCAAATAA
ATATTTATTTTGTTAGTATTTTGTTTATCATTAACAAATTTTAGTTTTAAAAAATAAAATAATAGCAAAAAATAATAAATAAAATTAATTTATGTTATATAAAATTTGATTAAATAATATAAAAAAAAAAAAATTTATTTTT
TATTTTATTAATTTTTCAATTTAAATAAGATATCTTAGAAGATTCGTAGAAATATTAAAATTAATTATTATTGATATTATTTAGTAATTCGTTGAAAATCAACTATTTTATATTATTAAAAAGACCAAAGAGTAAAATGAT
ATTTAGATAATTTGGTGCATTTTAAAGTAAAATTTAATTCTGCTTTTCTTCAAGCTCTCGTGGTTTTTTGAGAAGCGGTATAGGCTATAGTATAGATAATATTCCTGAATATGTGGATAAAGCTATTCAAAATTAATAAGT
TATAGATAATTTACAAAATTTGACAGATAAATATTTAATTGCAATTAGTAAGAATGACAAATAGTCTATATAGTAATTAACTAAAAAGAGTTTATGTGAAAAAACTAATTAGTGGATTGATGAAGTTTCCAAAAAGGGAGA
GAGCTTTTAGCTATTAGGTACAGATAATCAAAGTGTTTCTCTTTAATGTTTCACAAGTTATAGCTATATTTATGGATTGGCAAATAACTTGGATAACAAATAGTTCGAATCTCACTCAATTTATAATTTAAATAAAAACTT
TTTGGTGAAAAGGTTGTTATTCATAGAGGGTTTTAAAGCTCACCAATTATTAACAAAGAAATCTATTTTAGTAGAAGCATTTGTATACAACACTCTTAGACTTTCAATCAAAAAAGATACTAAGCAATAATAAGGCTAATA
AAATTTATAATATGCAGGTTATAAGATTTGTTTTGTTTGTGATGGAAAATACGAATAATAAACTCCCTATATATTGCTTGATAATGATAAAAAGTAATTGTACAGCCAAGAAGATAAGCAAAAGTCTTTCGAATTAAACTG
TTGGAAAATAGCAGATATCAACGATTGGATATTTGGTGATCTTTAGTAATAAGATTTTTAAAAATGGGCTTTCCAAGCAGTAATGGATTAATAAAATGAATAAATAGCTGACTTAAAATCAACTTAAACAAAATGATTATC
AATATTTTTATAAATTGTTTGTATATAAAATAAATTACTATAATTTAATTTATTATCAATTTTAAATATTTGATTTAAATATTTTATTAATTATTTAATATAATATAATTAAAAAGAAATTATTTTATTTTATTAAAAGCA
AATATTTTATTTAGATAATTTGTTAAATAATAAAAATGATTTAATTTAATTTAAATAAATAAACTAAATTATAAATTTAAGAATATTTAATGATTTAGTTTAATTTGAAAAAAATGTAAAAATATTTTTGATATTTTAAAA
TATTAATTTTACTGATAATCAATCTTAATCGTGTTATAAATTATTGAAGAAATTAATTTGTTTATTAAAAATATGTTTCAAAAATATTTTTTTCTATGAATAAAAAGTAGAATTGATTTATTTATTGATTTTGATATTAAA
TTAATATTTATTTTTAAATGCAAACTAGTTCTAGAGCGGCCGCCACCGCGGTGGAGCTCCAGCTTTTGTTCCCTTTAGTGAGGGTTAATTTCGAGCTTGGCGTAATCATGGTCATAGCTGTTTCCTGTGTGAAATTGTTAT
CCGCTCACAATTCCACACAACATACGAGCCGGAAGCATAAAGTGTAAAGCCTGGGGTGCCTAATGAGTGAGCTAACTCACATTAATTGCGTTGCGCTCACTGCCCGCTTTCCAGTCGGGAAACCTGTCGTGCCAGCTGCAT
TAATGAATCGGCCAACGCGCGGGGAGAGGCGGTTTGCGTATTGGGCGCTCTTCCGCTTCCTCGCTCACTGACTCGCTGCGCTCGGTCGTTCGGCTGCGGCGAGCGGTATCAGCTCACTCAAAGGCGGTAATACGGTTATCC
ACAGAATCAGGGGATAACGCAGGAAAGAACATGTGAGCAAAAGGCCAGCAAAAGGCCAGGAACCGTAAAAAGGCCGCGTTGCTGGCGTTTTTCCATAGGCTCCGCCCCCCTGACGAGCATCACAAAAATCGACGCTCAAGT
CAGAGGTGGCGAAACCCGACAGGACTATAAAGATACCAGGCGTTTCCCCCTGGAAGCTCCCTCGTGCGCTCTCCTGTTCCGACCCTGCCGCTTACCGGATACCTGTCCGCCTTTCTCCCTTCGGGAAGCGTGGCGCTTTCT
CATAGCTCACGCTGTAGGTATCTCAGTTCGGTGTAGGTCGTTCGCTCCAAGCTGGGCTGTGTGCACGAACCCCCCGTTCAGCCCGACCGCTGCGCCTTATCCGGTAACTATCGTCTTGAGTCCAACCCGGTAAGACACGAC
TTATCGCCACTGGCAGCAGCCACTGGTAACAGGATTAGCAGAGCGAGGTATGTAGGCGGTGCTACAGAGTTCTTGAAGTGGTGGCCTAACTACGGCTACACTAGAAGGACAGTATTTGGTATCTGCGCTCTGCTGAAGCCA
GTTACCTTCGGAAAAAGAGTTGGTAGCTCTTGATCCGGCAAACAAACCACCGCTGGTAGCGGTGGTTTTTTTGTTTGCAAGCAGCAGATTACGCGCAGAAAAAAAGGATCTCAAGAAGATCCTTTGATCTTTTCTACGGGG
TCTGACGCTCAGTGGAACGAAAACTCACGTTAAGGGATTTTGGTCATGAGATTATCAAAAAGGATCTTCACCTAGATCCTTTTAAATTAAAAATGAAGTTTTAAATCAATCTAAAGTATATATGAGTAAACTTGGTCTGAC
AGTTACCAATGCTTAATCAGTGAGGCACCTATCTCAGCGATCTGTCTATTTCGTTCATCCATAGTTGCCTGACTCCCCGTCGTGTAGATAACTACGATACGGGAGGGCTTACCATCTGGCCCCAGTGCTGCAATGATACCG
CGAGACCCACGCTCACCGGCTCCAGATTTATCAGCAATAAACCAGCCAGCCGGAAGGGCCGAGCGCAGAAGTGGTCCTGCAACTTTATCCGCCTCCATCCAGTCTATTAATTGTTGCCGGGAAGCTAGAGTAAGTAGTTCG
CCAGTTAATAGTTTGCGCAACGTTGTTGCCATTGCTACAGGCATCGTGGTGTCACGCTCGTCGTTTGGTATGGCTTCATTCAGCTCCGGTTCCCAACGATCAAGGCGAGTTACATGATCCCCCATGTTGTGCAAAAAAGCG
GTTAGCTCCTTCGGTCCTCCGATCGTTGTCAGAAGTAAGTTGGCCGCAGTGTTATCACTCATGGTTATGGCAGCACTGCATAATTCTCTTACTGTCATGCCATCCGTAAGATGCTTTTCTGTGACTGGTGAGTACTCAACC
AAGTCATTCTGAGAATAGTGTATGCGGCGACCGAGTTGCTCTTGCCCGGCGTCAATACGGGATAATACCGCGCCACATAGCAGAACTTTAAAAGTGCTCATCATTGGAAAACGTTCTTCGGGGCGAAAACTCTCAAGGATC
TTACCGCTGTTGAGATCCAGTTCGATGTAACCCACTCGTGCACCCAACTGATCTTCAGCATCTTTTACTTTCACCAGCGTTTCTGGGTGAGCAAAAACAGGAAGGCAAAATGCCGCAAAAAAGGGAATAAGGGCGACACGG
AAATGTTGAATACTCATACTCTTCCTTTTTCAATATTATTGAAGCATTTATCAGGGTTATTGTCTCATGAGCGGATACATATTTGAATGTATTTAGAAAAATAAACAAATAGGGGTTCCGCGCACATTTCCCCGAAAAGTG
C
```

```
CaM IES                    696..1994
CaM MDS left               2001..3558
CaM MDS right              3559..5105
pBlueScriptSK(+)           5100..695
```

Supplementary References

Aronica, L., Bednenko, J., Noto, T., DeSouza, L.V., Siu, K.W., Loidl, J., Pearlman, R.E., Gorovsky, M.A., and Mochizuki, K. (2008). Study of an RNA helicase implicates small RNA-noncoding RNA interactions in programmed DNA elimination in Tetrahymena. Genes Dev *22*, 2228-2241.

Busch, C.J., Vogt, A., and Mochizuki, K. (2010). Establishment of a Cre/loxP recombination system for N-terminal epitope tagging of genes in Tetrahymena. BMC Microbiol *10*, 191.

Cassidy-Hanley, D., Bowen, J., Lee, J.H., Cole, E., VerPlank, L.A., Gaertig, J., Gorovsky, M.A., and Bruns, P.J. (1997). Germline and somatic transformation of mating Tetrahymena thermophila by particle bombardment. Genetics *146*, 135-147.

Gorovsky, M.A., Yao, M.C., Keevert, J.B., and Pleger, G.L. (1975). Isolation of micro- and macronuclei of Tetrahymena pyriformis. Methods Cell Biol *9*, 311-327.

Iwamoto, M., Mori, C., Kojidani, T., Bunai, F., Hori, T., Fukagawa, T., Hiraoka, Y., and Haraguchi, T. (2009). Two distinct repeat sequences of Nup98 nucleoporins characterize dual nuclei in the binucleated ciliate tetrahymena. Curr Biol *19*, 843-847.

Kataoka, K., Schoeberl, U.E., and Mochizuki, K. (2010). Modules for C-terminal epitope tagging of Tetrahymena genes. J Microbiol Methods *82*, 342-346.

Keller, C., Adaixo, R., Stunnenberg, R., Woolcock, K.J., Hiller, S., and Buhler, M. (2012). HP1(Swi6) mediates the recognition and destruction of heterochromatic RNA transcripts. Mol Cell *47*, 215-227.

Liu, Y., Song, X., Gorovsky, M.A., and Karrer, K.M. (2005). Elimination of foreign DNA during somatic differentiation in Tetrahymena thermophila shows position effect and is dosage dependent. Eukaryot Cell *4*, 421-431.

Loidl, J., and Scherthan, H. (2004). Organization and pairing of meiotic chromosomes in the ciliate Tetrahymena thermophila. J Cell Sci *117*, 5791-5801.

Mochizuki, K. (2008). High efficiency transformation of Tetrahymena using a codon-optimized neomycin resistance gene. Gene *425*, 79-83.

Motl, J.A., and Chalker, D.L. (2011). Zygotic expression of the double-stranded RNA binding motif protein Drb2p is required for DNA elimination in the ciliate Tetrahymena thermophila. Eukaryot Cell *10*, 1648-1659.

Noto, T., Kurth, H.M., Kataoka, K., Aronica, L., DeSouza, L.V., Siu, K.W., Pearlman, R.E., Gorovsky, M.A., and Mochizuki, K. (2010). The Tetrahymena argonaute-binding protein Giw1p directs a mature argonaute-siRNA complex to the nucleus. Cell *140*, 692-703.

Schoeberl, U.E., Kurth, H.M., Noto, T., and Mochizuki, K. (2012). Biased transcription and selective degradation of small RNAs shape the pattern of DNA elimination in Tetrahymena. Genes Dev *26*, 1729-1742.

Taus, T., Kocher, T., Pichler, P., Paschke, C., Schmidt, A., Henrich, C., and Mechtler, K. (2011). Universal and confident phosphorylation site localization using phosphoRS. J Proteome Res *10*, 5354-5362.

Vogt, A., and Mochizuki, K. (2013). A domesticated PiggyBac transposase interacts with heterochromatin and catalyzes reproducible DNA elimination in Tetrahymena. PLoS Genet *9*, e1004032.

Woehrer, S.L., Aronica, L., Suhren, J.H., Busch, C.J., Noto, T., and Mochizuki, K. (2015). A Tetrahymena Hsp90 co-chaperone promotes siRNA loading by ATP-dependent and ATP-independent mechanisms. EMBO J *34*, 559-577.
