# Supplementary material for: Phosphorylation of an HP1-like Protein Regulates Heterochromatin Body Assembly for DNA Elimination
Source: Dev Cell. 2015 Dec 21;35(6):775–88. doi: 10.1016/j.devcel.2015.11.017 (PMC4695338; doi:10.1016/j.devcel.2015.11.017)

Developmental Cell

Supplemental Information

Phosphorylation of an HP1-like protein regulates heterochromatin body assembly for DNA elimination

Kensuke Kataoka and Kazufumi Mochizuki

Supplementary Data S1, related to Figure 1:

1. Nuclear events during the life cycle of *Tetrahymena thermophila* (related to Figure 1)
2. Summary of the protein localization screen (related to Figure 1)
3. Localizations of EGFP-tagged proteins (related to Figure 1)

1. Nuclear events during the life cycle of *Tetrahymena thermophila* (related to Figure 1)

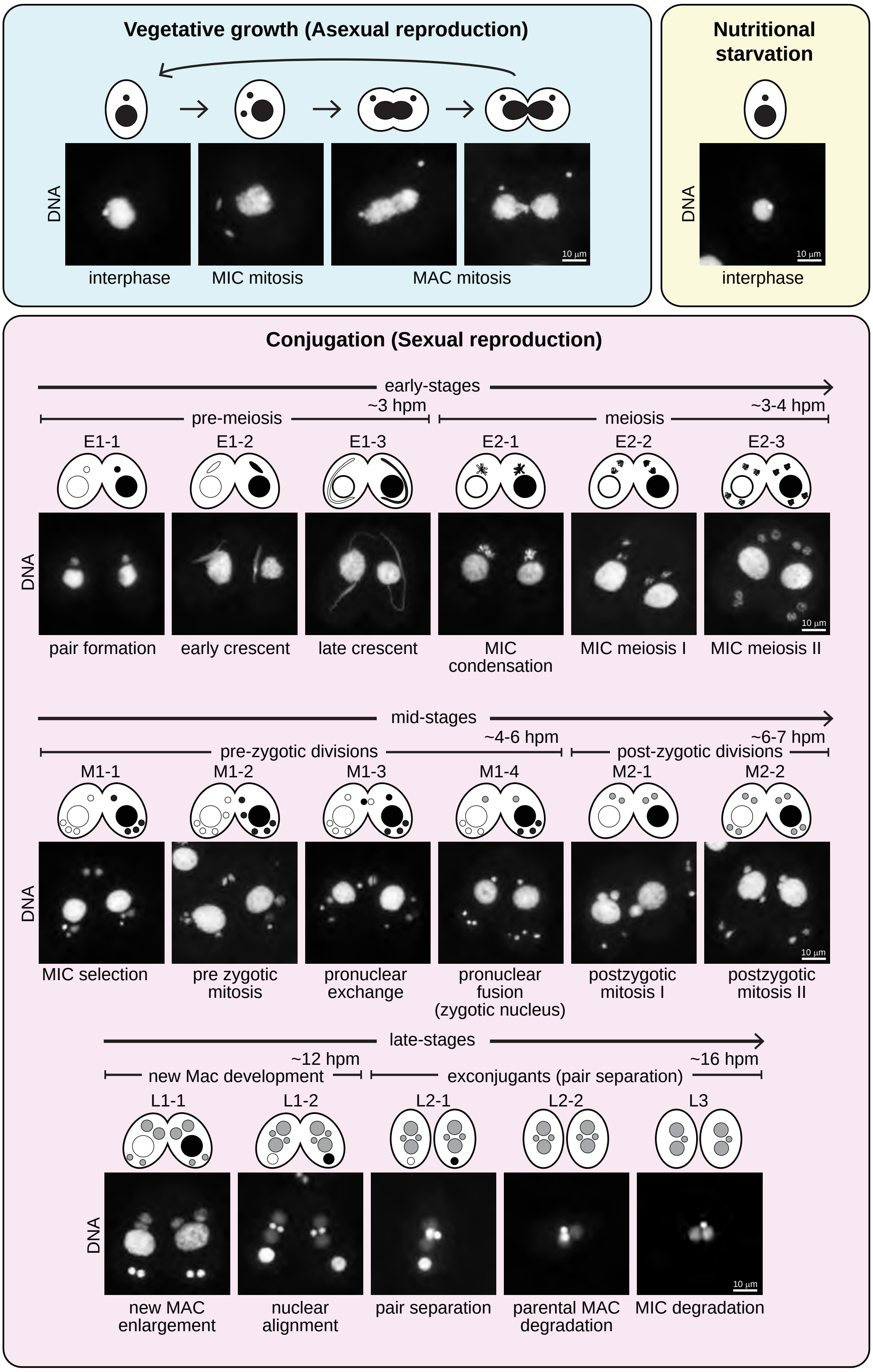

2. Summary of the protein localization screen (related to Figure 1)

					EGFP-tagged protein localization																
							conjugation stages**														
**TTHERM_#**	**GenBank**	**#EST**	**Gene name**	**Homology**	**Exponentially growing**	**Starved**	**E1-1**	**E1-2**	**E1-3**	**E2-1**	**E2-2**	**E2-3**	**M1-1**	**M1-3**	**M2-1**	**M2-2**	**L1-1**	**L1-2**	**L2-1**	**L2-2**	**L3**
TTHERM_00006160	XP_001008097.1	23	*COI15*		UD	UD	UD	UD	UD	UD	UD	UD	UD	UD	UD	UD	New MAC, Cytoplasm, Conjusome	New MAC, Cytoplasm, Conjusome	New MAC	New MAC	UD
TTHERM_00008690	XP_001008152.1	16		Peptidase C65 Otubain-like	UD	UD	UD	UD	UD	UD	UD	UD	UD	UD	UD	UD	Cytoplasm, Nuclei	Cytoplasm, Nuclei	Parental MAC	UD	UD
TTHERM_000279929	EWS73862.1	18	*JUB2*		UD	UD	UD	UD	UD	UD	UD	UD	UD	UD	UD	UD	Parental MAC, New MAC	Parental MAC, New MAC	Parental MAC, New MAC, Heterochromatin body	New MAC, Heterochromatin body	New MAC, Heterochromatin body
TTHERM_000295779	EWS75101.1	18			UD	UD	UD	MIC	MIC	MIC	MIC	MIC	MIC	MIC	MIC	UD	UD	UD	UD	UD	UD
TTHERM_00036890	XP_977090.1	14			UD	UD	UD	UD	Parental MAC	Parental MAC	Parental MAC	Parental MAC	Parental MAC	Parental MAC	Parental MAC	Parental MAC	Parental MAC, New MAC	Parental MAC, New MAC	Parental MAC, New MAC	New MAC	UD
TTHERM_00049220	XP_001014817.3	19	*COI20*		UD	UD	UD	UD	MIC, Conjugation junction	MIC, Conjugation junction	MIC, Conjugation junction	MIC, Conjugation junction	MIC, Conjugation junction	MIC, Conjugation junction	Conjugation junction	Conjugation junction	Conjugation junction	Conjugation junction	UD	UD	UD
TTHERM_000549648	EWS76725.1	18		Guanylyl cyclase, ATPase-like	UD	UD	UD	UD	UD	UD	UD	UD	UD	UD	Parental MAC	Parental MAC	New MAC	New MAC	New MAC	UD	UD
TTHERM_00079530	XP_001015793.1	29	*COI5*	Cyclin N-terminal domain	UD	UD	UD	Conjugation junction	Conjugation junction	Conjugation junction	Conjugation junction	Conjugation junction	Conjugation junction	Conjugation junction	UD	UD	UD	UD	UD	UD	UD
TTHERM_00086720	XP_001012720.1	28	*COI6*	Chromodomain, HP1-like	UD	UD	UD	UD	UD	UD	UD	UD	UD	UD	Parental MAC	Parental MAC	Parental MAC, New MAC, Conjusome	New MAC, Conjusome	New MAC	New MAC, Heterochromatin body	New MAC, Heterochromatin body
TTHERM_00092800	XP_001012833.2	21			UD	UD	Cytoplasm	Cytoplasm	Cytoplasm	Cytoplasm	Cytoplasm	Cytoplasm	Cytoplasm	Cytoplasm	Cytoplasm	Cytoplasm	Cytoplasm	Cytoplasm	UD	UD	UD
TTHERM_00112830	XP_001010678.3	18			UD	UD	UD	UD	UD	Parental MAC	UD	UD	UD	UD	UD	UD	UD	UD	UD	UD	UD
TTHERM_00133730	XP_001019652.2	13			UD	UD	MIC	MIC	MIC	UD	UD	UD	UD	UD	UD	UD	UD	UD	UD	UD	UD
TTHERM_00189440	XP_001016620.1	25	*COI11*	Leucine-rich repeats	UD	UD	UD	UD	UD	UD	UD	UD	Around MIC	Around MIC	Conjugation junction	Conjugation junction	Conjugation junction	Conjugation junction, Parental MAC	Conjugation junction	Conjugation junction	UD
TTHERM_00193969	XP_001471369.2	19	*COI21*		UD	UD	UD	UD	UD	UD	UD	UD	UD	UD	UD	UD	Parental MAC, New MAC, Cytoplasm, Conjusome	New MAC, Cytoplasm, Conjusome	New MAC	New MAC	UD
TTHERM_00219320	XP_001020604.1	38			UD	UD	Parental MAC	Parental MAC	Parental MAC	Parental MAC	Parental MAC	UD	UD	UD	UD	UD	UD	UD	UD	UD	UD
TTHERM_00237610	EAS04546.2	19	*JUB1*		UD	UD	UD	UD	UD	UD	UD	UD	UD	UD	Parental MAC, Conjusome	Conjusome	New MAC, Conjusome	New MAC, Conjusome	New MAC, Heterochromatin body	New MAC, Heterochromatin body	New MAC, Heterochromatin body
TTHERM_00289290	XP_001018646.2	17			UD	UD	MIC	MIC	MIC	MIC	UD	UD	UD	UD	UD	UD	MIC, New MAC	MIC, New MAC	MIC, New MAC	UD	UD
TTHERM_00299879	XP_001471014.1	69	*WAG1*	GW Repeat	UD	UD	UD	Parental MAC	Parental MAC	Parental MAC	Parental MAC	Parental MAC	Parental MAC	Parental MAC	Parental MAC	Parental MAC	Parental MAC, New MAC, Cytoplasm	New MAC	New MAC	UD	UD
TTHERM_00370840	XP_001009514.2	17		Adhesin lipoprotein-like	UD	UD	UD	UD	Granules (lysosome/ autophagosome) in posterior cytoplasm	Granules (lysosome/ autophagosome) in posterior cytoplasm	Granules (lysosome/ autophagosome) in posterior cytoplasm	Granules (lysosome/ autophagosome) in posterior cytoplasm	Granules (lysosome/ autophagosome) in posterior cytoplasm	Granules (lysosome/ autophagosome) in posterior cytoplasm	Granules (lysosome/ autophagosome) in posterior cytoplasm	Granules (lysosome/ autophagosome) in posterior cytoplasm	Granules (lysosome/ autophagosome) in posterior cytoplasm	Granules (lysosome/ autophagosome) in posterior cytoplasm, around Parental MAC	Granules (lysosome/ autophagosome) in posterior cytoplasm, around Parental MAC	UD	UD
TTHERM_00402050	XP_001014078.3	22	*COI12*	TPR domain	UD	UD	UD	Cytoplasm, MIC, parental MAC	Cytoplasm, MIC, parental MAC	Cytoplasm, Parental MAC	Cytoplasm, Parental MAC	Cytoplasm, Parental MAC	UD	UD	UD	UD	UD	UD	UD	UD	UD
TTHERM_00420400*	XP_001033295.2	24	*COI14*		UD	UD	UD	UD	MIC	MIC	MIC	MIC	MIC	MIC	MIC	MIC	MIC, New MAC	UD	UD	UD	UD
TTHERM_00433790	XP_001011404.1	24		TPR domain	UD	UD	Basal body/Mitochondria	Basal body/Mitochondria	Basal body/Mitochondria	Basal body/Mitochondria	Basal body/Mitochondria	Basal body/Mitochondria	Basal body/Mitochondria	Basal body/Mitochondria	Basal body/Mitochondria	Basal body/Mitochondria	Basal body/Mitochondria	Basal body/Mitochondria	Basal body/Mitochondria	Basal body/Mitochondria	Basal body/Mitochondria
TTHERM_00442420	XP_001033155.3	18	*JUB3*	WD-40 domain, ESC-like	UD	UD	UD	Parental MAC	Parental MAC	Parental MAC	Parental MAC	Parental MAC	Parental MAC	Parental MAC	Parental MAC	Parental MAC	Parental MAC, New MAC	New MAC	New MAC	New MAC, Heterochromatin body	New MAC, Heterochromatin body
TTHERM_00471000	XP_001032990.1	17			UD	UD	MIC	MIC	MIC	MIC, parental MAC	MIC, parental MAC	MIC, parental MAC	MIC, parental MAC	MIC, parental MAC	MIC, parental MAC	MIC, parental MAC	MIC, New MAC	MIC, New MAC	MIC, New MAC	UD	UD
TTHERM_00471720	XP_001033056.3	15			UD	UD	UD	UD	Parental MAC	Parental MAC	Parental MAC	Parental MAC	Parental MAC	Parental MAC	Parental MAC	Parental MAC	New MAC	New MAC	New MAC	UD	UD
TTHERM_00474920	XP_001023974.1	15			UD	UD	MIC	MIC	MIC	MIC	MIC	MIC	MIC	MIC, MIC	UD	UD	UD	UD	UD	UD	UD
TTHERM_00497670	XP_001027955.2	24	*COI17*		UD	UD	UD	UD	Parental MAC	Parental MAC	Parental MAC	Parental MAC	Parental MAC	Parental MAC	Parental MAC	Parental MAC	Parental MAC, New MAC	Parental MAC, New MAC	Parental MAC, New MAC	Parental MAC, New MAC	UD
TTHERM_00499370	XP_001022195.1	14		LIA3-like	UD	UD	UD	UD	UD	UD	UD	UD	UD	UD	UD	UD	New MAC, Conjusome	New MAC, Conjusome	New MAC, Conjusome	UD	UD
TTHERM_00522820	XP_001014460.2	32	*COI4*		UD	UD	UD	UD	Conjugation junction	Conjugation junction	Conjugation junction	Conjugation junction	Conjugation junction	Conjugation junction	Conjugation junction	Conjugation junction	Conjugation junction	Conjugation junction	Conjugation junction	ND	ND
TTHERM_00526270a*	XP_001028038.1	(39)	*COI3*		UD	UD	UD	UD	UD	UD	UD	UD	UD	UD	UD	Parental MAC, Conjusome	Parental MAC, New MAC, Conjusome	Parental MAC, New MAC, Conjusome	Parental MAC, New MAC, Heterochromatin body	New MAC, Heterochromatin body	UD
TTHERM_00526270b*	XP_001028038.1	(39)		Peptidase family C54-like	UD	UD	UD	UD	UD	UD	UD	UD	UD	UD	UD	UD	UD	Cytoplasm	Cytoplasm	UD	UD
TTHERM_00564480	XP_001022047.3	23	*COI16*	LIA6-like	UD	UD	UD	UD	UD	UD	UD	UD	UD	UD	UD	Parental MAC	Parental MAC, New MAC, Conjusome	Parental MAC, New MAC, Conjusome	Parental MAC, New MAC, Heterochromatin body	New MAC, Heterochromatin body	New MAC, Heterochromatin body
TTHERM_00572190	XP_001027725.2	40			UD	UD	MIC	MIC	MIC	MIC	MIC	MIC	MIC	MIC	MIC	MIC	MIC, New MAC, Conjusome	MIC, New MAC	MIC, New MAC	MIC, New MAC	UD
TTHERM_00616290	XP_001024699.1	14	*JUB4*		UD	UD	UD	UD	Parental MAC	Parental MAC	Parental MAC	Parental MAC	Parental MAC	Parental MAC	Parental MAC	Parental MAC	Parental MAC, New MAC	New MAC	New MAC, Heterochromatin body	New MAC, Heterochromatin body	UD
TTHERM_00622830	XP_001022508.1	14		DHHC zinc finger domain	UD	UD	Granules (lysosome/ autophagosome) in posterior cytoplasm	Granules (lysosome/ autophagosome) in posterior cytoplasm	Granules (lysosome/ autophagosome) in posterior cytoplasm	Granules (lysosome/ autophagosome) in posterior cytoplasm	Granules (lysosome/ autophagosome) in posterior cytoplasm	Granules (lysosome/ autophagosome) in posterior cytoplasm	Granules (lysosome/ autophagosome) in posterior cytoplasm	Granules (lysosome/ autophagosome) in posterior cytoplasm	Granules (lysosome/ autophagosome) in posterior cytoplasm	Granules (lysosome/ autophagosome) in posterior cytoplasm	Granules (lysosome/ autophagosome) in posterior cytoplasm	Granules (lysosome/ autophagosome) in posterior cytoplasm, around Parental MAC	Granules (lysosome/ autophagosome) in posterior cytoplasm, around Parental MAC	UD	UD
TTHERM_00637050	XP_001032366.1	15			UD	UD	MIC	MIC	MIC	UD	UD	UD	UD	MIC	MIC	MIC	MIC, New MAC	MIC, New MAC	UD	UD	UD
TTHERM_00649180	XP_001032312.3	43		Smc-like	UD	UD	Basal body/Mitochondria, Cytoplasm	Basal body/Mitochondria, Cytoplasm	Basal body/Mitochondria, Cytoplasm	Basal body/Mitochondria, Cytoplasm	Basal body/Mitochondria, Cytoplasm	Basal body/Mitochondria, Cytoplasm	Basal body/Mitochondria, Cytoplasm	Basal body/Mitochondria, Cytoplasm	Basal body/Mitochondria, Cytoplasm	Basal body/Mitochondria, Cytoplasm	Basal body/Mitochondria, Cytoplasm	Basal body/Mitochondria, Cytoplasm	UD	UD	UD
TTHERM_00693080	XP_001023573.1	18		Cyclin N-terminal domain	UD	UD	UD	UD	MIC	MIC	MIC	MIC	MIC	MIC	UD	UD	UD	UD	UD	UD	UD
TTHERM_00728900	XP_001022676.1	16			UD	UD	Conjugation junction	Conjugation junction	Conjugation junction	Conjugation junction	Conjugation junction	Conjugation junction	Conjugation junction	Conjugation junction	Conjugation junction	Conjugation junction	Conjugation junction	Conjugation junction	Conjugation junction	UD	UD
TTHERM_00825660	XP_001031434.3	17			UD	UD	UD	UD	UD	UD	UD	UD	UD	UD	UD	UD	UD	New MAC, Cytoplasm	New MAC	New MAC	UD
TTHERM_01085480	XP_001030381.1	19	*COI19*		UD	UD	UD	UD	UD	UD	UD	UD	UD	UD	Parental MAC	Parental MAC	New MAC	New MAC	UD	UD	UD
TTHERM_01132870	EAS07342.3	18		TPR domain	UD	UD	Parental MAC	Parental MAC	Parental MAC	Parental MAC	Parental MAC	Parental MAC	Parental MAC	Parental MAC	Parental MAC	Parental MAC	Parental MAC	Parental MAC	Parental MAC	UD	UD
TTHERM_01276320	XP_001029843.2	62	*COI1 (GIW1)*		UD	UD	UD	Cytoplasm, MIC, parental MAC	Cytoplasm, MIC, parental MAC	Cytoplasm, MIC, parental MAC	Cytoplasm, MIC, parental MAC	Cytoplasm, MIC, parental MAC	Cytoplasm, MIC, parental MAC	Cytoplasm, MIC, parental MAC	Cytoplasm, MIC, parental MAC	Cytoplasm, MIC, parental MAC	Cytoplasm, MIC, Parental MAC, New MAC	Cytoplasm, MIC, Parental MAC, New MAC	Cytoplasm, MIC, Parental MAC, New MAC	ND	ND
TTHERM_01285910	XP_001029830.2	16	*ZFR1*	B-box zinc finger domain	UD	UD	Conjugation junction	Conjugation junction	Conjugation junction	Conjugation junction	Conjugation junction	Conjugation junction	Conjugation junction	Conjugation junction	Conjugation junction	Conjugation junction	Conjugation junction	Conjugation junction	Conjugation junction	UD	UD
TTHERM_01337400	XP_001029724.3	53	*JUB5*	Chromodomain, HP1-like	UD	UD	Parental MAC	Parental MAC	Parental MAC	Parental MAC	Parental MAC	Parental MAC	Parental MAC	Parental MAC	Parental MAC	Parental MAC	Parental MAC, New MAC, Conjusome	New MAC, Conjusome	New MAC, Heterochromatin body	New MAC, Heterochromatin body	New MAC, Heterochromatin body
TTHERM_01367700	XP_001026268.1	25	*COI10*		UD	UD	UD	UD	Parental MAC	Parental MAC	Parental MAC	Parental MAC	Parental MAC	Parental MAC	Parental MAC	Parental MAC	New MAC, Conjusome	New MAC, Conjusome	New MAC, Conjusome	UD	UD
TTHERM_00013110	XP_001008290.2	14		Importin-beta amine-terminal domain	MIC, MAC, Cytoplasm	MIC, MAC, Cytoplasm	MIC, Cytoplasm	MIC, Cytoplasm	MIC, Parental MAC, Cytoplasm	MIC, Parental MAC, Cytoplasm	MIC, Parental MAC, Cytoplasm	MIC, Parental MAC, Cytoplasm	MIC, Parental MAC, Cytoplasm	MIC, Cytoplasm	MIC, Cytoplasm	MIC, Cytoplasm	Parental MAC, MIC, New MAC, Cytoplasm	MIC, New MAC, Cytoplasm	MIC, New MAC, Cytoplasm	MIC, New MAC, Cytoplasm	MIC, New MAC
TTHERM_000245499	EWS72076.1	18		F-box domain	MIC, MAC	MAC	MIC, Parental MAC	MIC, Parental MAC	MIC, Parental MAC	MIC, Parental MAC	MIC, Parental MAC	MIC, Parental MAC	MIC, Parental MAC	MIC, Parental MAC	MIC, Parental MAC	MIC, Parental MAC	Parental MAC, New MAC	Parental MAC, New MAC	Parental MAC, New MAC	New MAC	New MAC
TTHERM_00028580	XP_976854.1	20		KOW motif, Spt5p-like	MAC, (MIC)	MAC	MIC, Parental MAC	MIC, Parental MAC	MIC, Parental MAC	MIC, Parental MAC	MIC, Parental MAC	MIC, Parental MAC	MIC, Parental MAC	MIC, Parental MAC	MIC, Parental MAC	MIC, Parental MAC	Parental MAC, New MAC	Parental MAC, New MAC	Parental MAC, New MAC	New MAC	New MAC
TTHERM_00091510	XP_001012803.1	14			Cytoplasm	Cytoplasm	Cytoplasm	Cytoplasm	Cytoplasm	Cytoplasm	Cytoplasm	Cytoplasm	Cytoplasm	Cytoplasm	Cytoplasm	Cytoplasm	Cytoplasm	Cytoplasm	Cytoplasm	UD	UD
TTHERM_00113310	XP_001010726.3	17		CTD binding domain?	MAC	MAC	Parental MAC	Parental MAC	Parental MAC	Parental MAC	Parental MAC	Parental MAC	Parental MAC	Parental MAC	Parental MAC	Parental MAC	Parental MAC, New MAC	Parental MAC, New MAC, Cytoplasm , Conjusome	Parental MAC, New MAC	New MAC	New MAC
TTHERM_00185640	XP_001008835.2	24	*JMJ1*	JmjC domain, JMJD3-like	MAC	UD	Parental MAC	Parental MAC	Parental MAC	Parental MAC	Parental MAC	Parental MAC	Parental MAC	Parental MAC	Parental MAC	Parental MAC	Parental MAC	Parental MAC, New MAC	New MAC	New MAC	UD
TTHERM_00204150	XP_001007101.1	15		RING-finger domain	MAC	MAC	Parental MAC	Parental MAC	Parental MAC	Parental MAC	Parental MAC	Parental MAC	Parental MAC	Parental MAC	Parental MAC	Parental MAC	Parental MAC, New MAC	New MAC	New MAC	New MAC	New MAC
TTHERM_00221110	XP_001020683.3	18		Pumilio-family RNA binding domain	Cytoplasm	Cytoplasm	Cytoplasm	Cytoplasm	Cytoplasm	Cytoplasm	Cytoplasm	Cytoplasm	Cytoplasm	Cytoplasm	Cytoplasm	Cytoplasm	Cytoplasm	Cytoplasm	Cytoplasm	UD	UD
TTHERM_00267890	XP_001015924.2	19		Zinc finger domain	MAC	MAC	Parental MAC	Parental MAC	Parental MAC	Parental MAC	Parental MAC	Parental MAC	Parental MAC	Parental MAC	Parental MAC	Parental MAC	Parental MAC, New MAC	Parental MAC, New MAC	Parental MAC, New MAC	New MAC	New MAC
TTHERM_00301910	XP_001024619.1	13		DNA topoisomerase 2-like	MAC	MAC	Parental MAC	Parental MAC	Parental MAC	Parental MAC	Parental MAC	Parental MAC	Parental MAC	Parental MAC	Parental MAC	Parental MAC	Parental MAC, New MAC	New MAC, Cytoplasm	New MAC	New MAC	New MAC
TTHERM_00439300	XP_001017827.2	24			MAC	MAC	Parental MAC	Parental MAC	Parental MAC	Parental MAC	Parental MAC	Parental MAC	Parental MAC	Parental MAC	Parental MAC	Parental MAC	Parental MAC, New MAC	Parental MAC, New MAC	New MAC	New MAC	UD
TTHERM_00460720	XP_001018768.2	15			MAC	MAC	UD	UD	Parental MAC	Parental MAC	Parental MAC	Parental MAC	Parental MAC	Parental MAC	Parental MAC	Parental MAC	New MAC	New MAC	New MAC	New MAC	UD
TTHERM_00471730	XP_001033057.1	15		Nuclear cap binding protein -like	MAC	MAC	Parental MAC	Parental MAC	Parental MAC	Parental MAC	Parental MAC	Parental MAC	Parental MAC	Parental MAC	Parental MAC	Parental MAC	Parental MAC, New MAC	Parental MAC, New MAC, Cytoplasm	Parental MAC, New MAC	New MAC	New MAC
TTHERM_00474360	XP_001023920.1	31			MAC	UD	Parental MAC	Parental MAC	Parental MAC	Parental MAC	Parental MAC	Parental MAC	Parental MAC	Parental MAC	Parental MAC	Parental MAC	Parental MAC, New MAC	Parental MAC, New MAC	Parental MAC, New MAC	UD	UD
TTHERM_00487030	XP_001032915.1	16		Thymidine Kinase-like	Cytoplasm	UD	UD	UD	UD	UD	UD	UD	UD	Cytoplasm	Cytoplasm	UD	UD	UD	UD	UD	UD
TTHERM_00540090	XP_001007934.1	16		Dimerization/Docking domain of the sperm fibrous sheath protein	Cilia	Cilia	Cilia	Cilia	Cilia	Cilia	Cilia	Cilia	Cilia	Cilia	Cilia	Cilia	Cilia	Cilia	Cilia	Cilia	Cilia
TTHERM_00704020*	XP_001032072.1	14			MAC	MAC	Parental MAC	Parental MAC	Parental MAC	Parental MAC	Parental MAC	Parental MAC	Parental MAC	Parental MAC	Parental MAC	Parental MAC	Parental MAC	Parental MAC, New MAC	Parental MAC, New MAC	New MAC	New MAC
TTHERM_00706330	XP_001010963.2	13			MAC	MAC	Parental MAC	Parental MAC	Parental MAC	Parental MAC	Parental MAC	Parental MAC	Parental MAC	Parental MAC	Parental MAC	Parental MAC	Parental MAC, New MAC	Parental MAC, New MAC, Cytoplasm	Parental MAC, New MAC	New MAC	New MAC
TTHERM_00849260	XP_001019228.3	30	*LIA6*		MAC	UD	Parental MAC	Parental MAC	Parental MAC	Parental MAC	Parental MAC	Parental MAC	Parental MAC	Parental MAC	Parental MAC	Parental MAC	Parental MAC, New MAC	Parental MAC, New MAC	Parental MAC, New MAC	Parental MAC, New MAC	UD
TTHERM_00974120	XP_001009965.1	15		Smc-like	Nuclear membrane of MAC	Nuclear membrane of MAC	Nuclear membrane of Parental MAC	Nuclear membrane of Parental MAC	Nuclear membrane of Parental MAC	Nuclear membrane of Parental MAC	Nuclear membrane of Parental MAC	Nuclear membrane of Parental MAC	Nuclear membrane of Parental MAC	Nuclear membrane of Parental MAC	Nuclear membrane of Parental MAC	Nuclear membrane of Parental MAC	Nuclear membrane of P-, New MAC	Nuclear membrane of New MAC	Nuclear membrane of New MAC	Nuclear membrane of New MAC	Nuclear membrane of New MAC
TTHERM_01014770	XP_001030823.3	13			MIC, MAC	MIC, MAC	MIC, Parental MAC	MIC, Parental MAC	MIC, Parental MAC	MIC, Parental MAC	MIC, Parental MAC	MIC, Parental MAC	MIC, Parental MAC	Pronucleus, Parental MAC	Pronucleus, Parental MAC	Pronucleus, Parental MAC	Parental MAC, MIC, New MAC	MIC, New MAC	MIC, New MAC	MIC, New MAC	MIC, New MAC
TTHERM_00034970	XP_976998.2	22		Protein kinase domain	UD	UD	UD	UD	UD	UD	UD	UD	UD	UD	UD	UD	UD	UD	UD	UD	UD
TTHERM_00046930	XP_001014688.3	41	*COI2*		UD	UD	UD	UD	UD	UD	UD	UD	UD	UD	UD	UD	UD	UD	UD	UD	UD
TTHERM_00112710	XP_001010666.1	19			UD	UD	UD	UD	UD	UD	UD	UD	UD	UD	UD	UD	UD	UD	UD	UD	UD
TTHERM_00128920	XP_001016373.1	23			UD	UD	UD	UD	UD	UD	UD	UD	UD	UD	UD	UD	UD	UD	UD	UD	UD
TTHERM_00133710	XP_001019650.1	22			UD	UD	UD	UD	UD	UD	UD	UD	UD	UD	UD	UD	UD	UD	UD	UD	UD
TTHERM_00155590	XP_001009685.3	52		HMG box	UD	UD	UD	UD	UD	UD	UD	UD	UD	UD	UD	UD	UD	UD	UD	UD	UD
TTHERM_00197670	XP_977298.1	20	*COI18*		UD	UD	UD	UD	UD	UD	UD	UD	UD	UD	UD	UD	UD	UD	UD	UD	UD
TTHERM_00309960*	XP_001021072.3	13			UD	UD	UD	UD	UD	UD	UD	UD	UD	UD	UD	UD	UD	UD	UD	UD	UD
TTHERM_00341280	XP_001018372.1	13			UD	UD	UD	UD	UD	UD	UD	UD	UD	UD	UD	UD	UD	UD	UD	UD	UD
TTHERM_00412010a*	XP_001020895.1	(23)			UD	UD	UD	UD	UD	UD	UD	UD	UD	UD	UD	UD	UD	UD	UD	UD	UD
TTHERM_00412010b*	XP_001020895.1	(23)			UD	UD	UD	UD	UD	UD	UD	UD	UD	UD	UD	UD	UD	UD	UD	UD	UD
TTHERM_00501010	XP_001022260.2	21			UD	UD	UD	UD	UD	UD	UD	UD	UD	UD	UD	UD	UD	UD	UD	UD	UD
TTHERM_00529550	XP_001032666.1	14			UD	UD	UD	UD	UD	UD	UD	UD	UD	UD	UD	UD	UD	UD	UD	UD	UD
TTHERM_00569460	XP_001027650.2	18			UD	UD	UD	UD	UD	UD	UD	UD	UD	UD	UD	UD	UD	UD	UD	UD	UD
TTHERM_00664050	XP_001020171.2	19			UD	UD	UD	UD	UD	UD	UD	UD	UD	UD	UD	UD	UD	UD	UD	UD	UD
TTHERM_00775940	XP_001021228.1	18			UD	UD	UD	UD	UD	UD	UD	UD	UD	UD	UD	UD	UD	UD	UD	UD	UD
TTHERM_00775949	EDK31494.1	18			UD	UD	UD	UD	UD	UD	UD	UD	UD	UD	UD	UD	UD	UD	UD	UD	UD
TTHERM_01014530	EAR83136.2	13		Peptidyl-tRNA hydrolase domain	UD	UD	UD	UD	UD	UD	UD	UD	UD	UD	UD	UD	UD	UD	UD	UD	UD
TTHERM_01358410	XP_001013522.1	24		ABC transporter-like	UD	UD	UD	UD	UD	UD	UD	UD	UD	UD	UD	UD	UD	UD	UD	UD	UD

* See primer sequences to determine the open reading frame used in this study.
** See "2. Nuclear events during the life cycle of *Tetrahymena thermophila*" for the conjugation stages
UD-Undetected

3. Localizations of EGFP-tagged proteins (related to Figure 1)

Cells expressing EGFP-tagged proteins (green), which were growing, starved or conjugating with wild-type cells, were fixed and DNA was stained with DAPI (magenta). For conjugation stages (E1-1~L3), see "1. Nuclear events during the life cycle of *Tetrahymena thermophila*".

TTHERM_00006160
GenBank; XP_001008097.1
Gene name; *COI15*

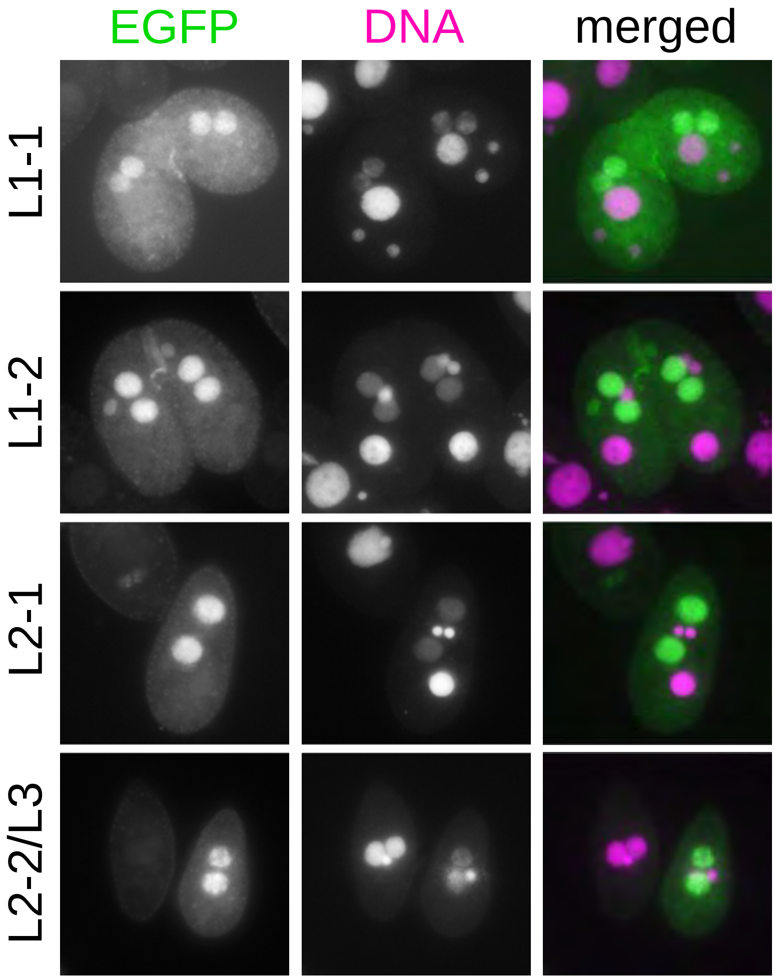

TTHERM_00008690
GenBank; XP_001008152.1

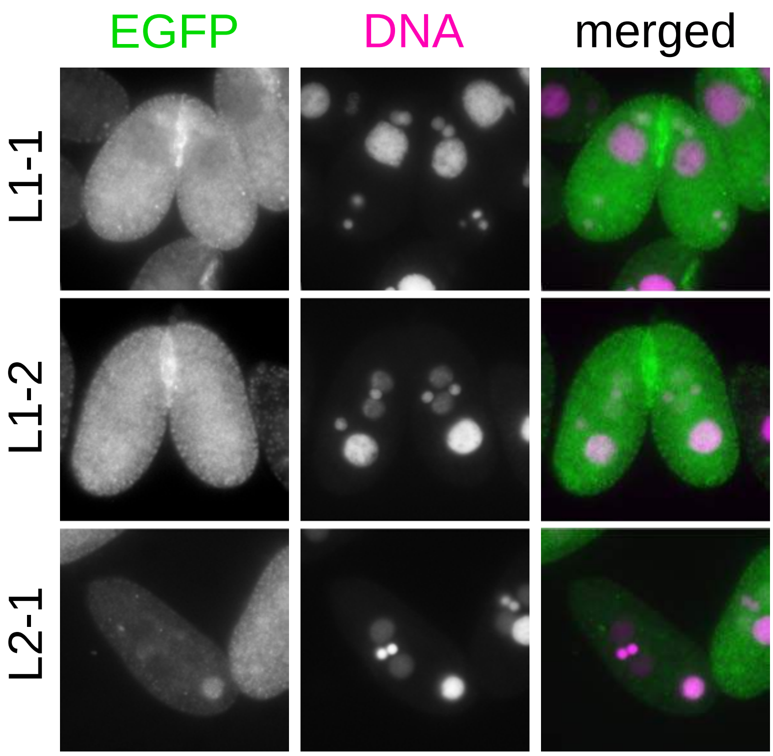

TTHERM_000279929
GenBank; EWS73862.1
Gene name; *JUB2*

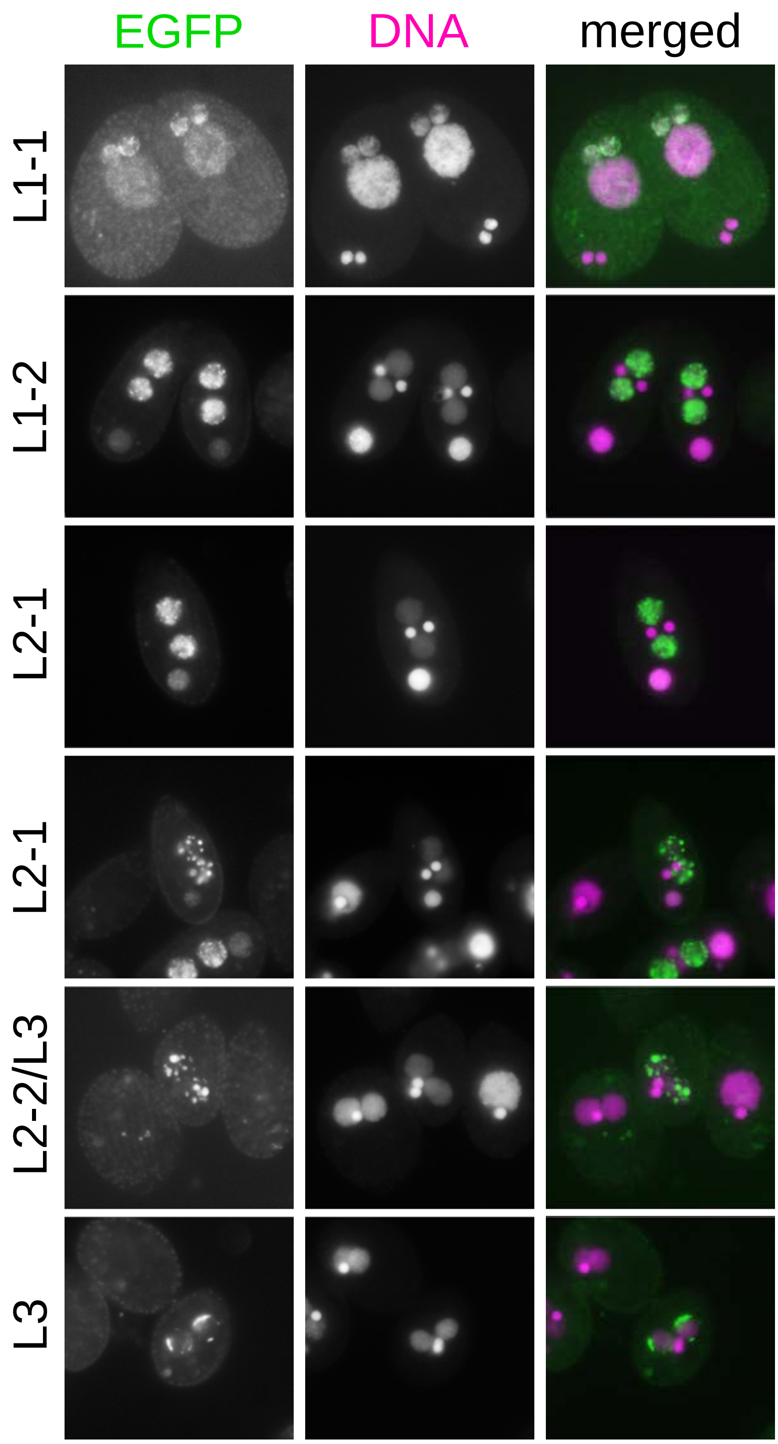

TTHERM_000295779
GenBank; EWS75101.1

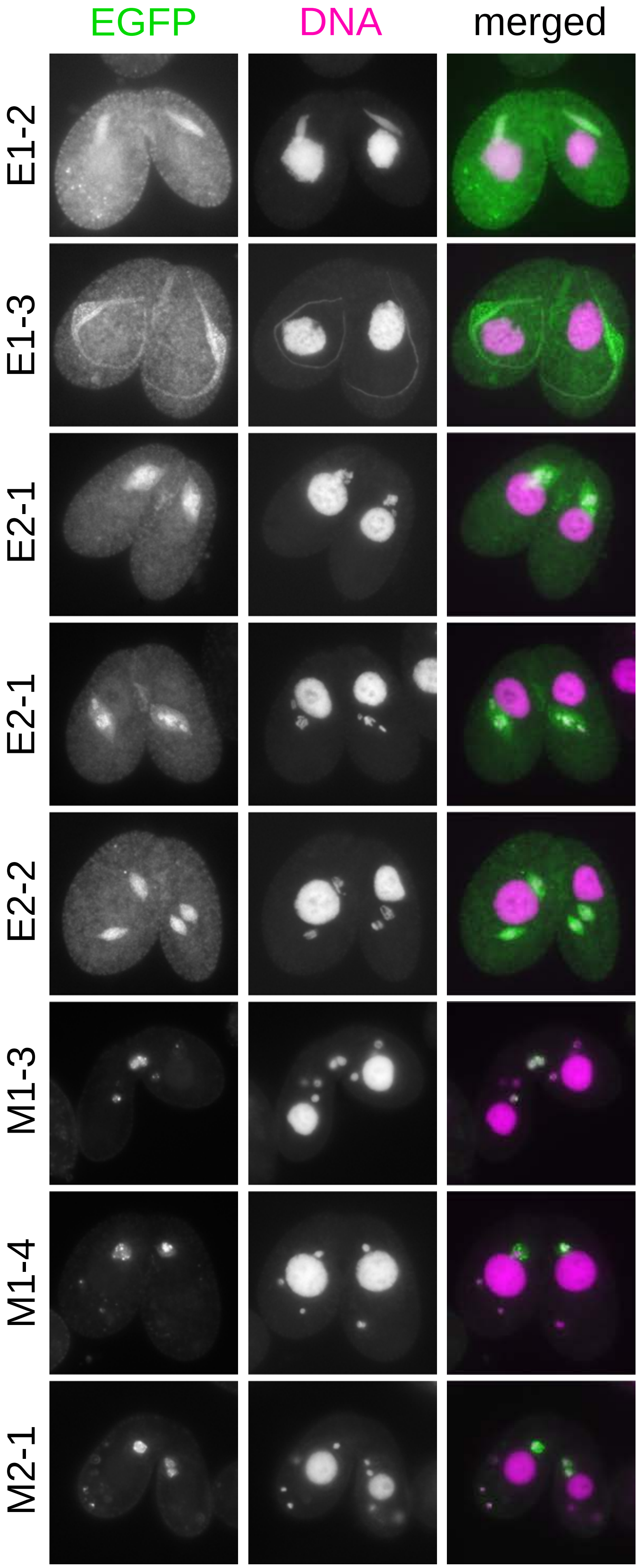

TTHERM_00036890
GenBank; XP_977090.1

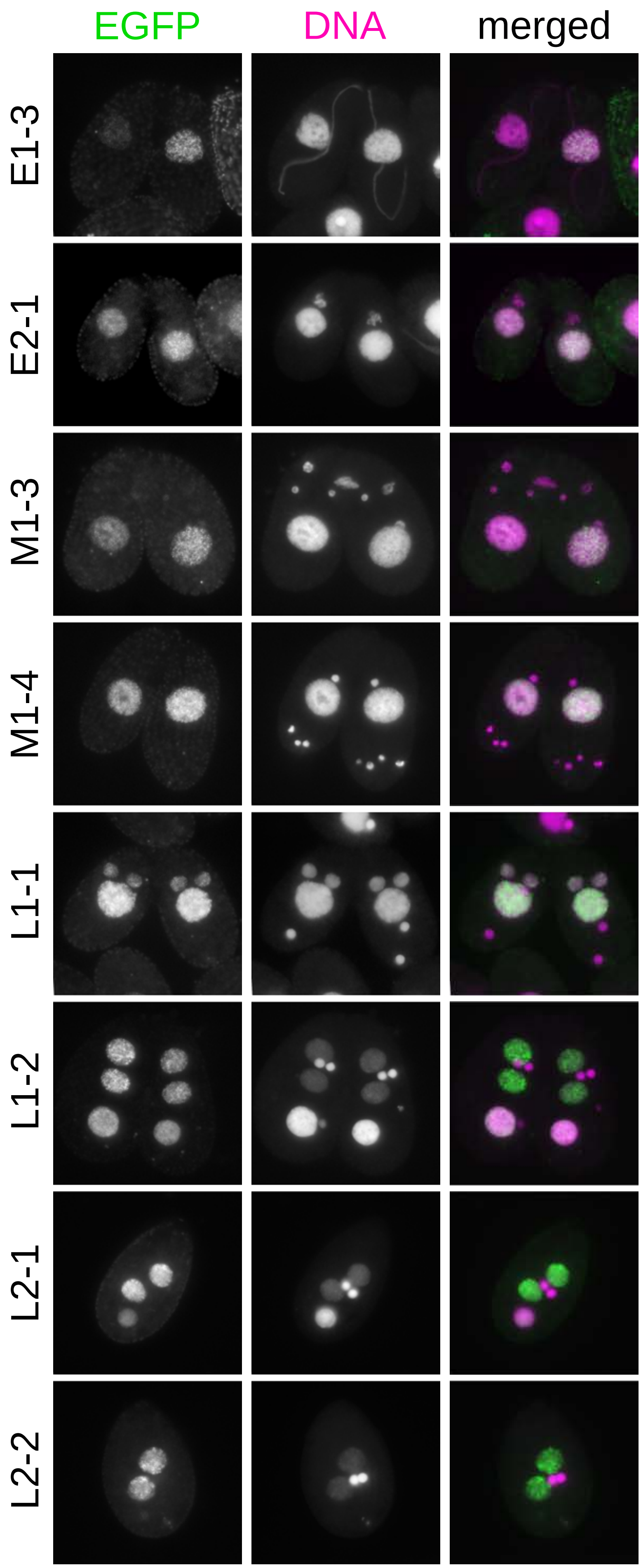

TTHERM_00049220
GenBank; XP_001014817.3
Gene name; *COI20*

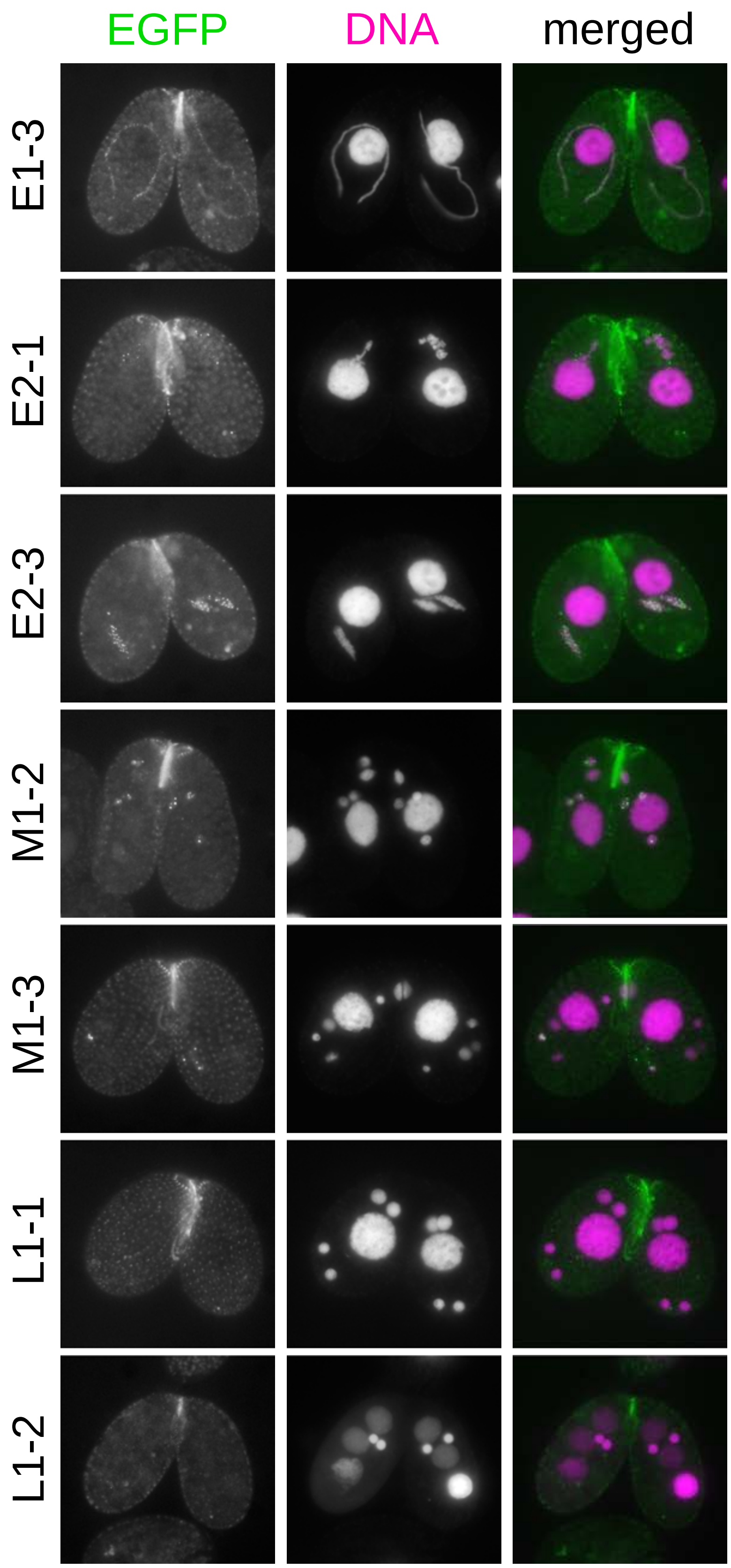

TTHERM_000549648
GenBank; EWS76725.1

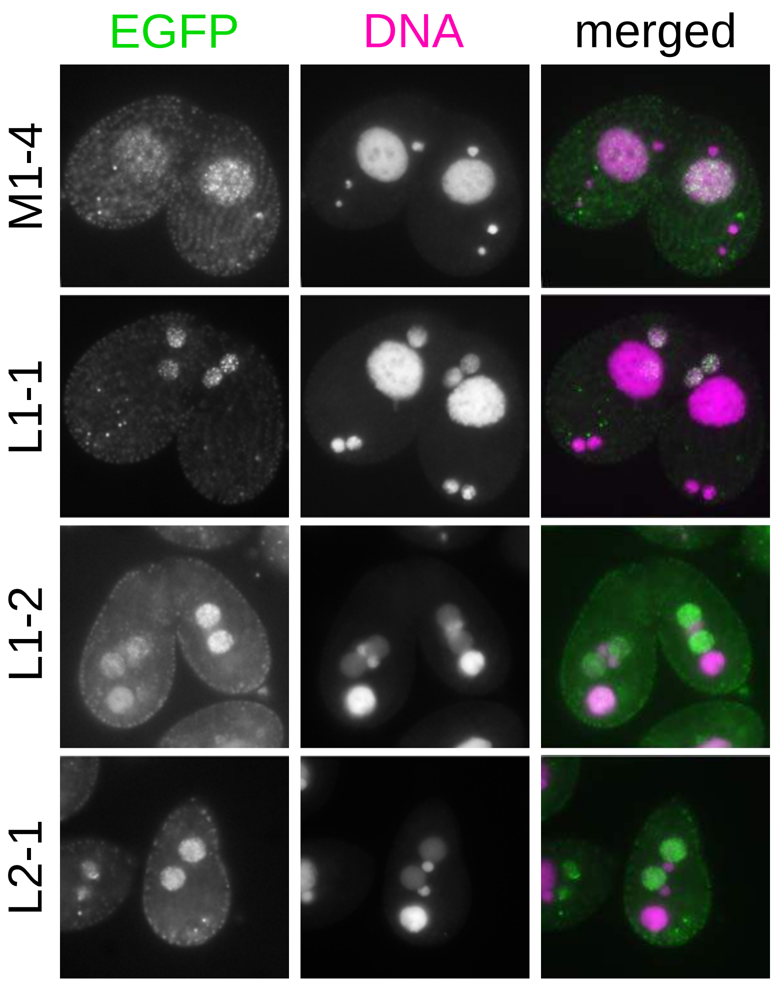

TTHERM_00079530
GenBank; XP_001015793.1
Gene name; *COI5*

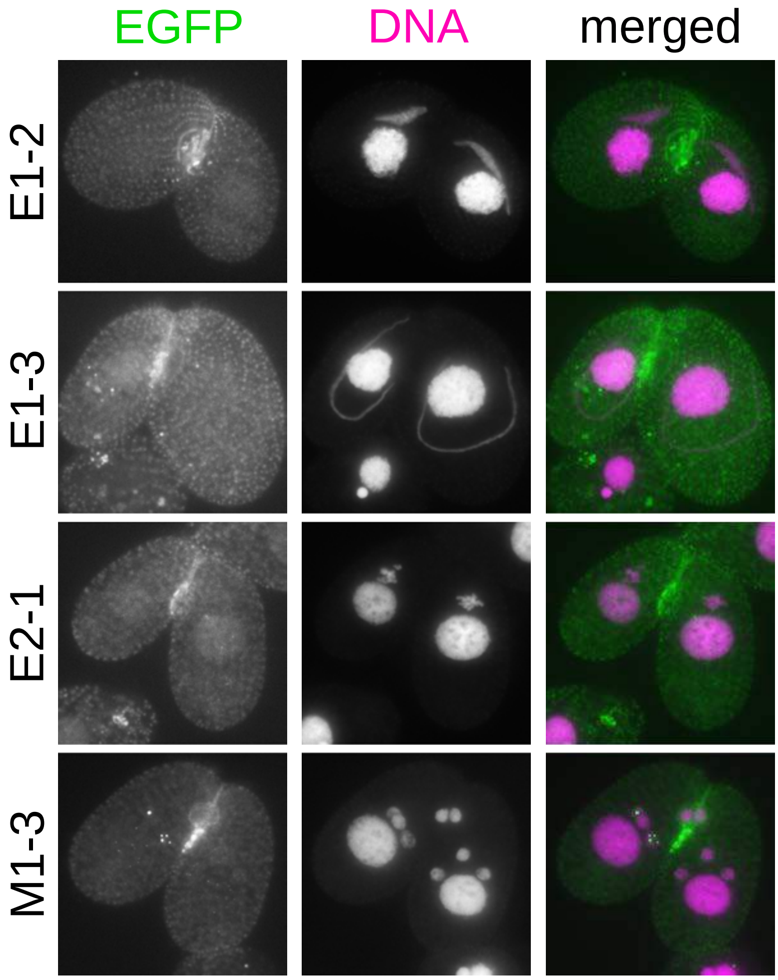

TTHERM_00086720
GenBank; XP_001012720.1
Gene name; *COI6*

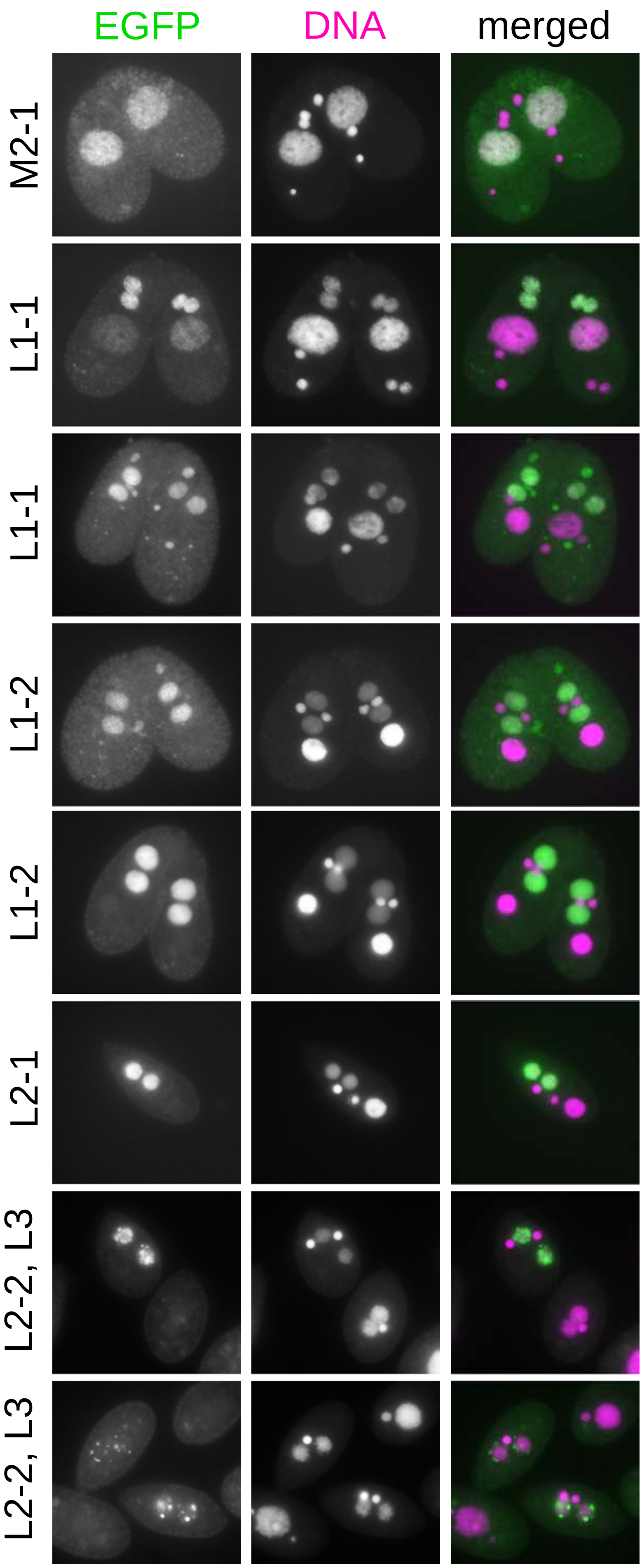

TTHERM_00092800
GenBank; XP_001012833.2

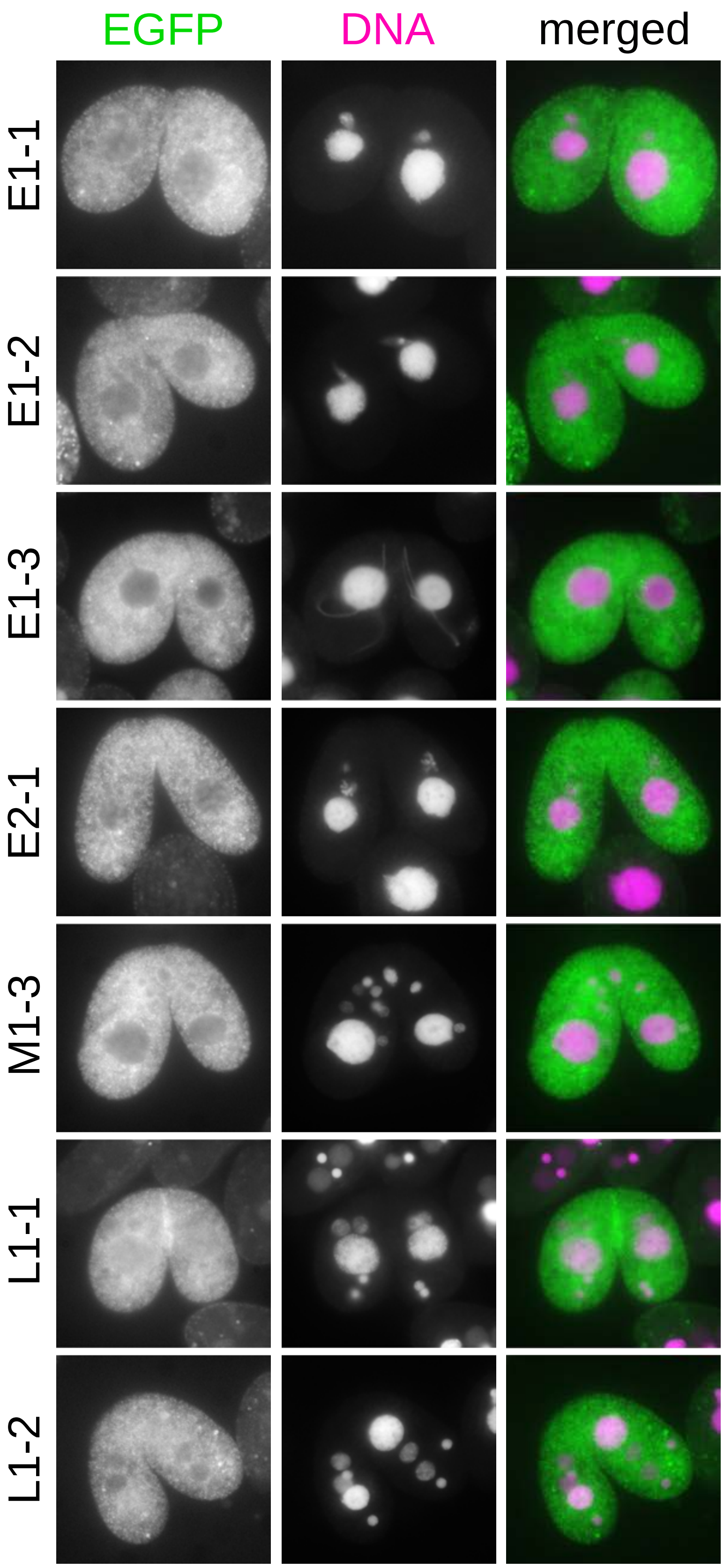

TTHERM_00112830
GenBank; XP_001010678.3

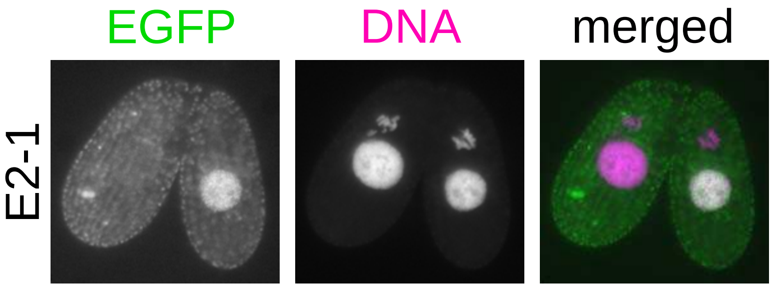

TTHERM_00133730
GenBank; XP_001019652.2

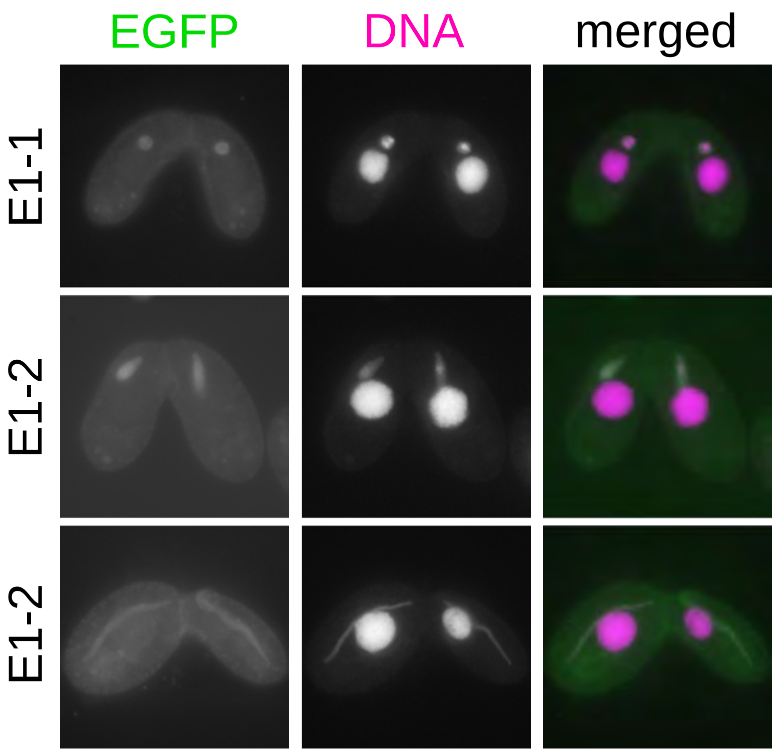

TTHERM_00189440
GenBank; XP_001016620.1
Gene name; *COI11*

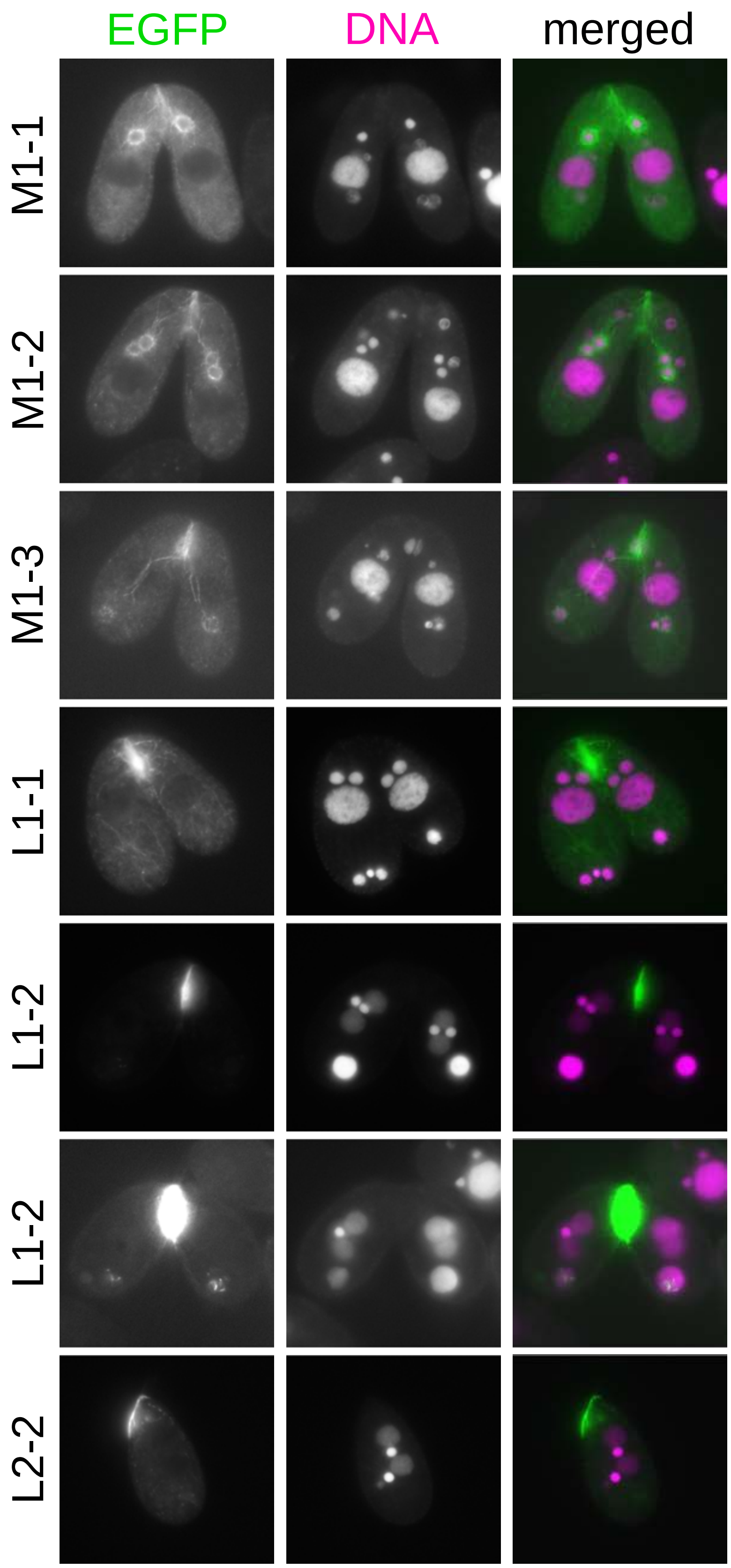

TTHERM_00193969
GenBank; XP_001471369.2
Gene name; *COI21*

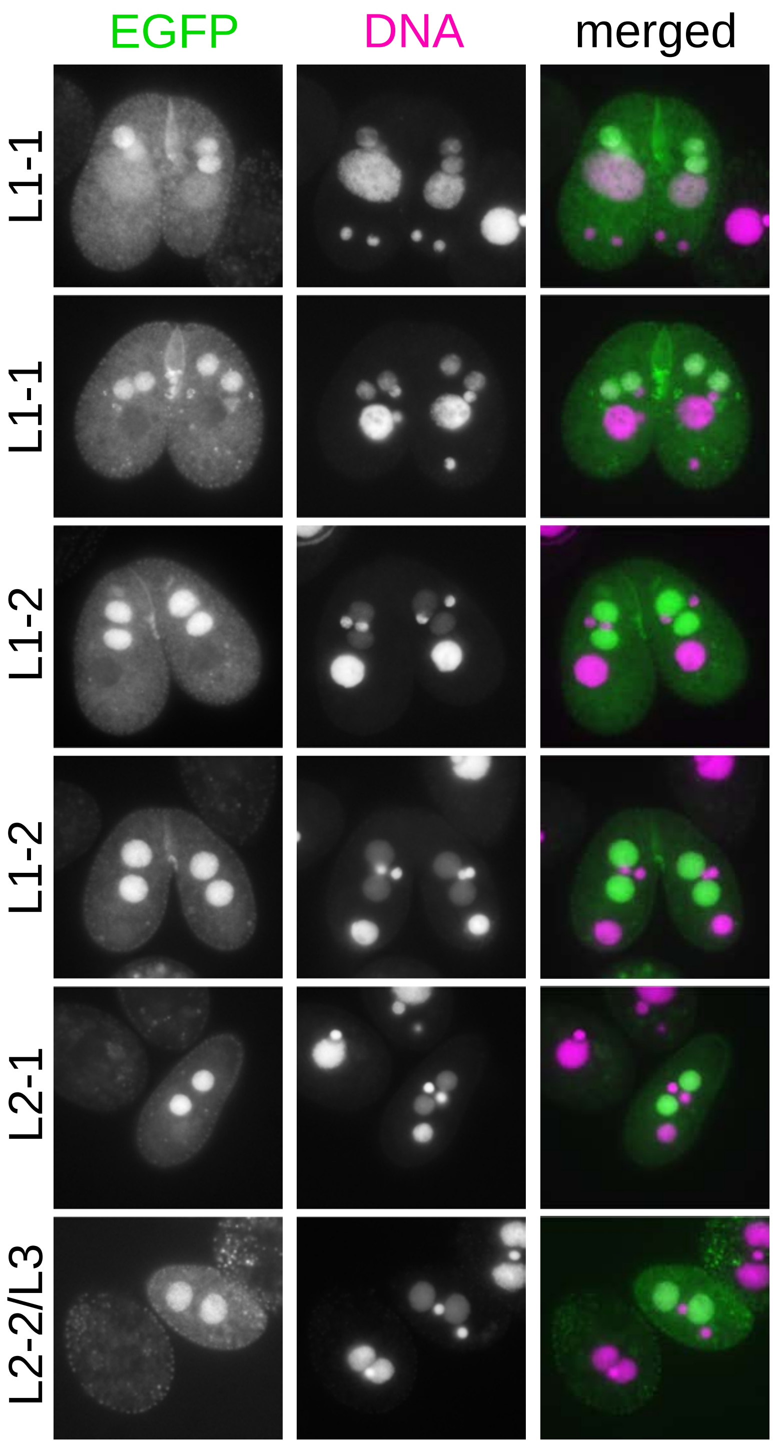

TTHERM_00219320
GenBank; XP_001020604.1

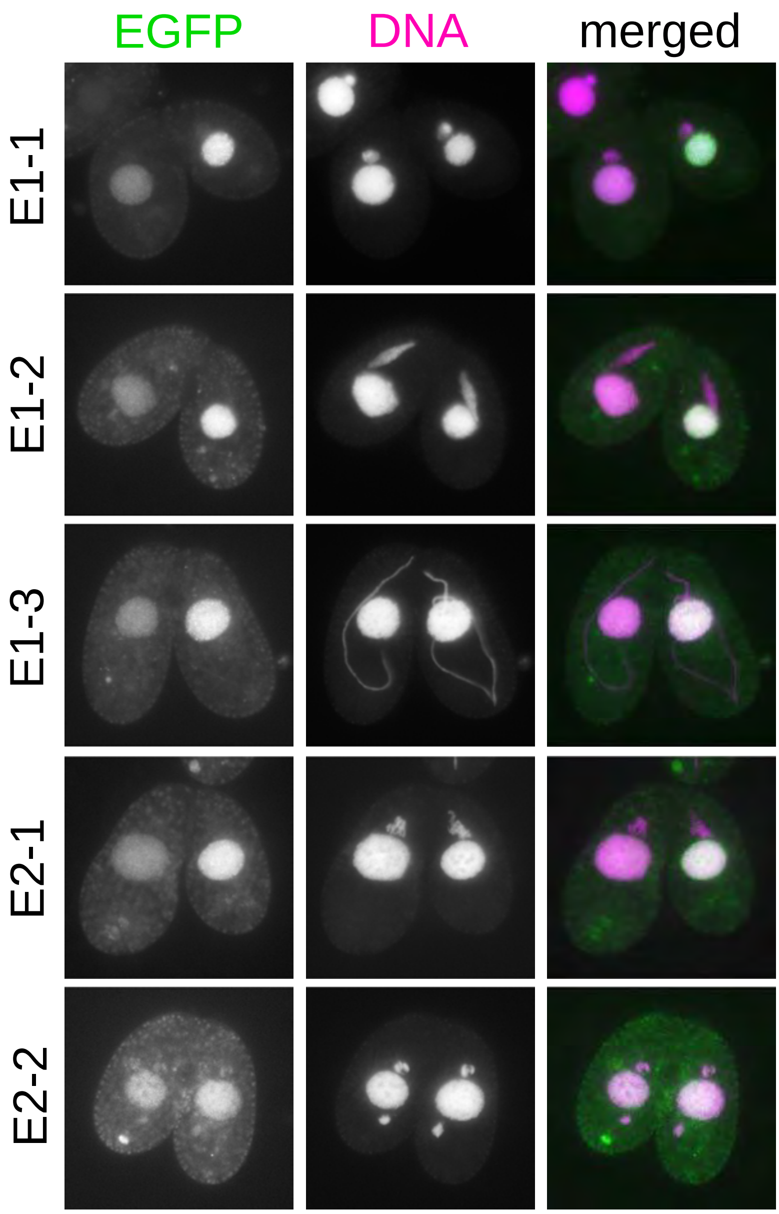

TTHERM_00237610
GenBank; EAS04546.2
Gene name; *JUB1*

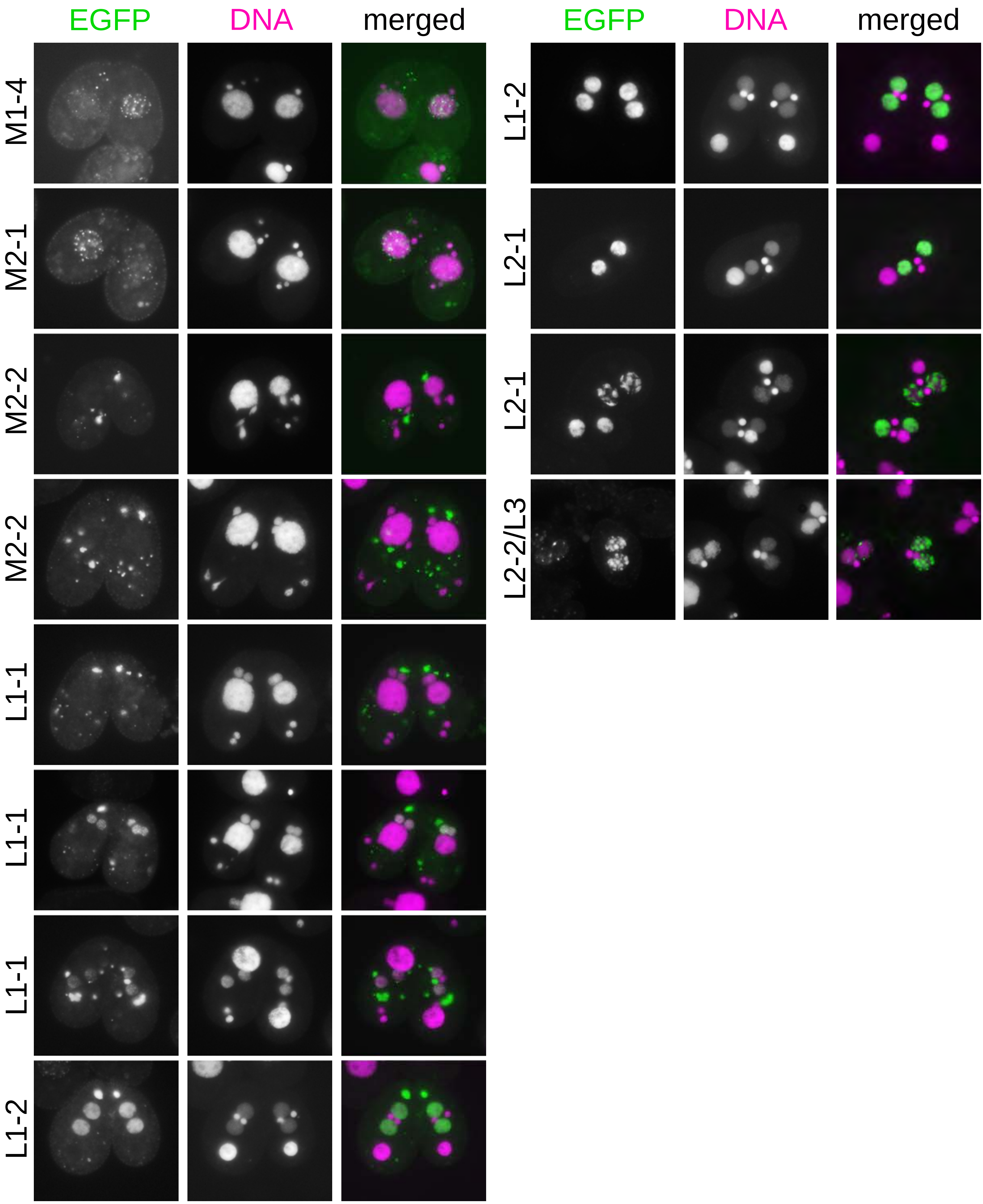

TTHERM_00289290
GenBank; XP_001018646.2

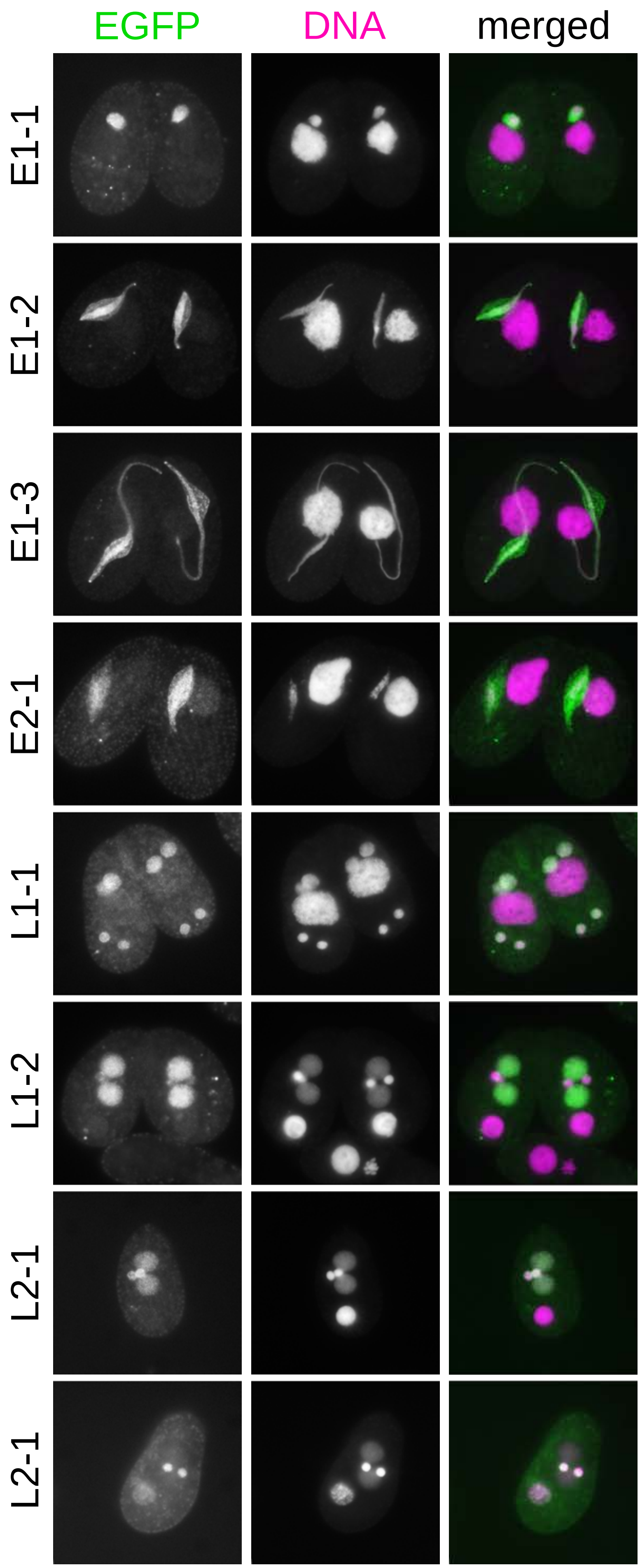

TTHERM_00299879
GenBank; XP_001471014.1
Gene name; *WAG1*

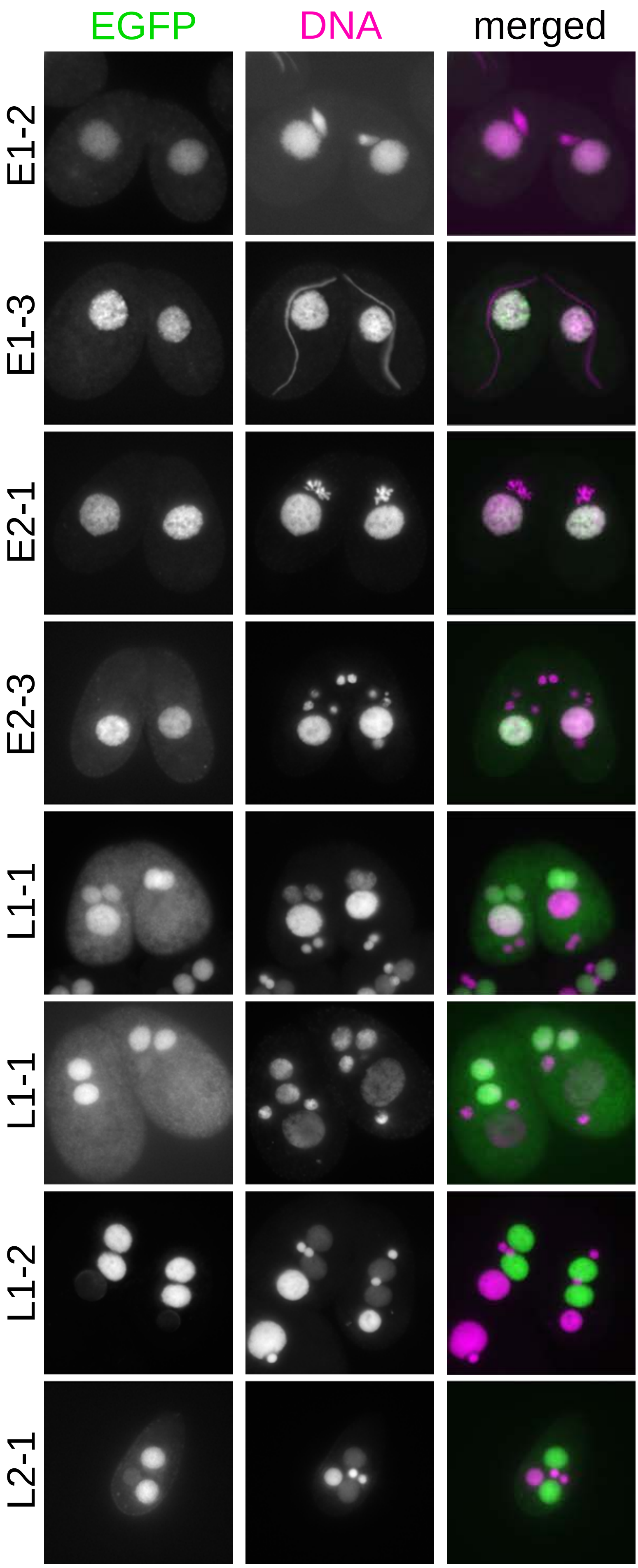

TTHERM_00370840
GenBank; XP_001009514.2

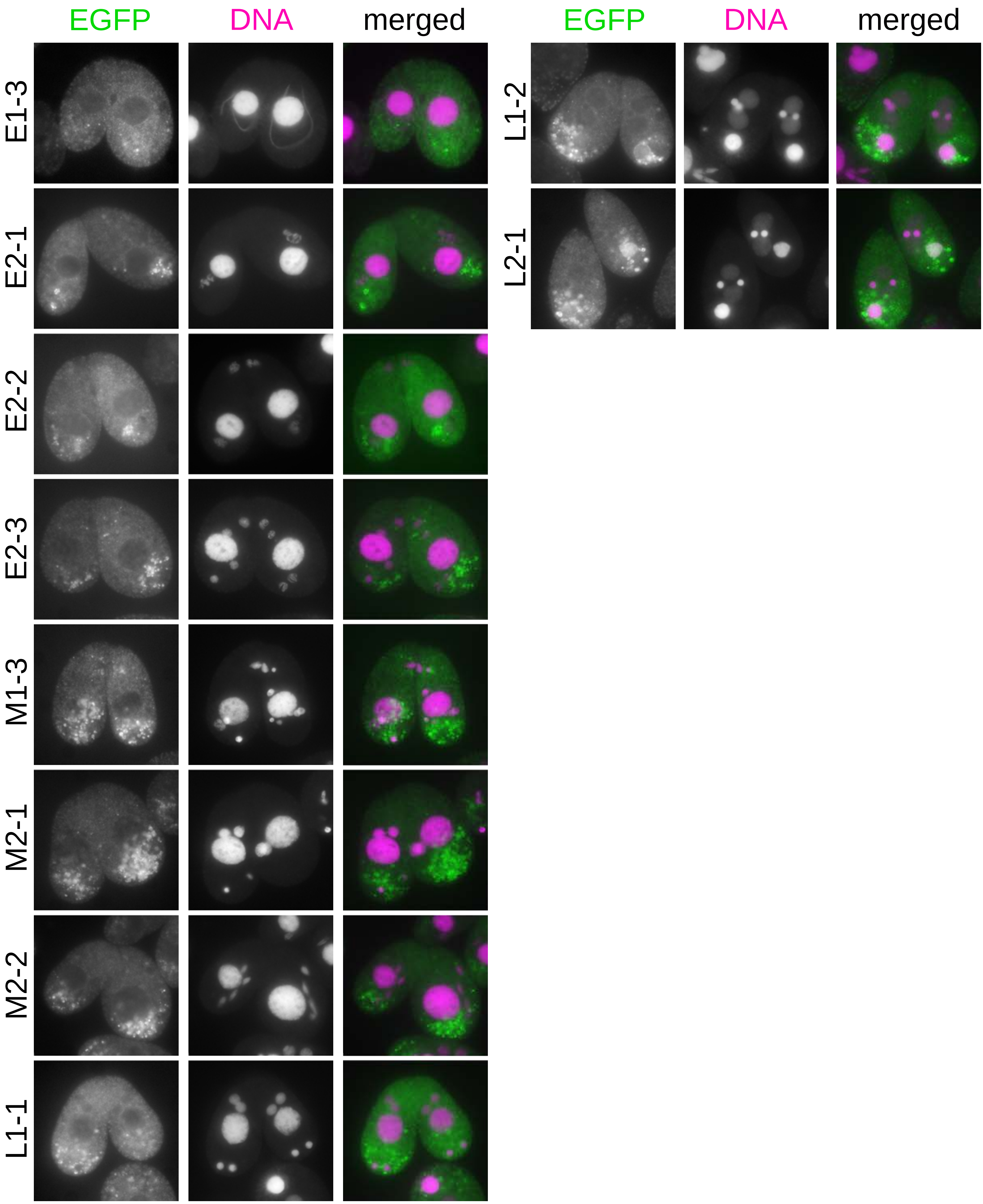

TTHERM_00402050
GenBank; XP_001014078.3
Gene name; *COI12*

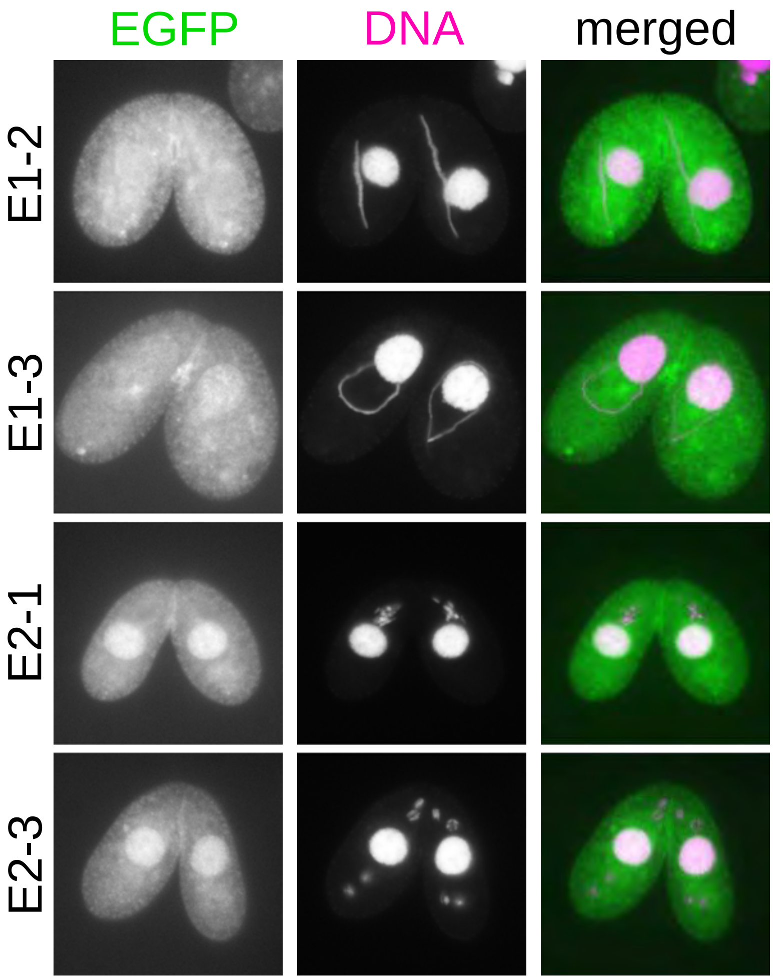

TTHERM_00420400*
GenBank; XP_001033295.2
Gene name; *COI14*

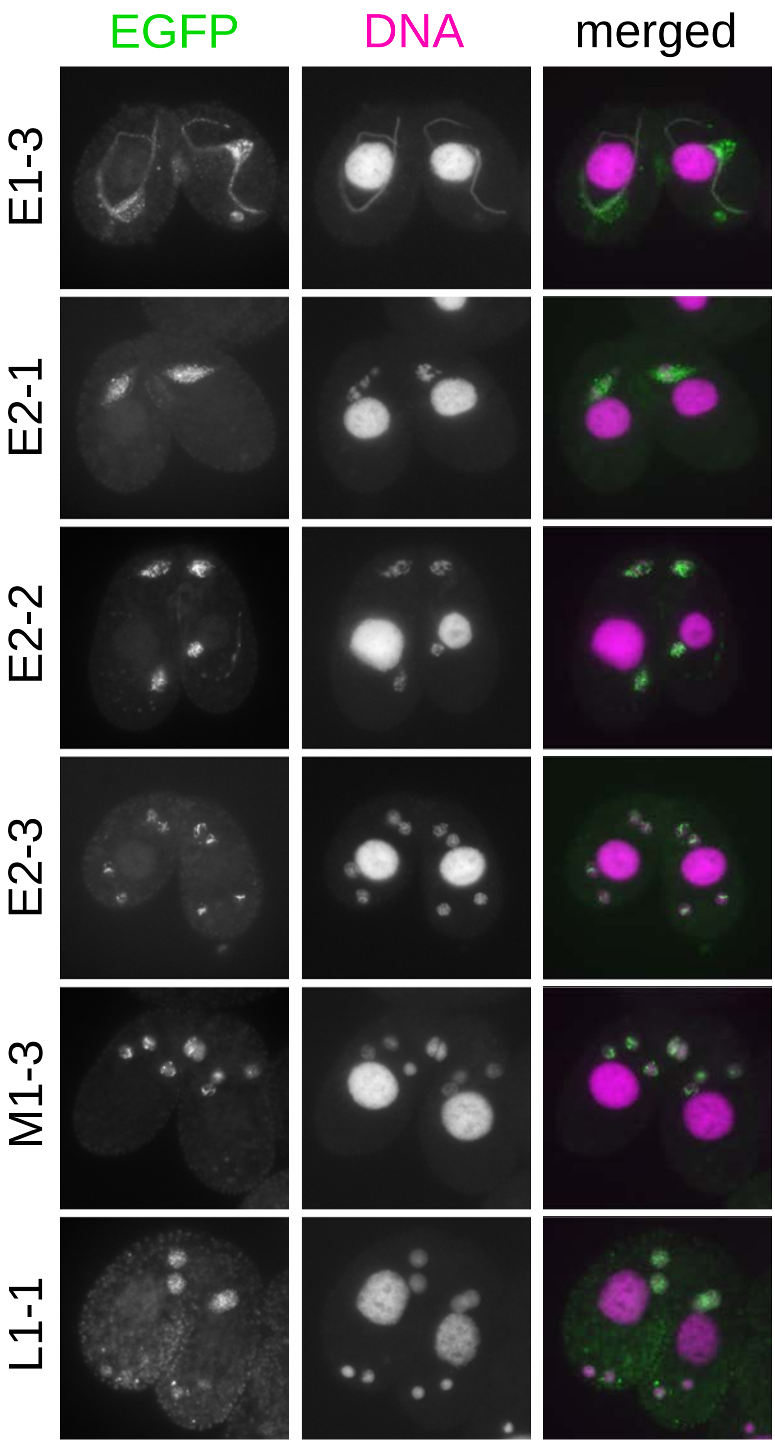

TTHERM_00433790
GenBank; XP_001011404.1

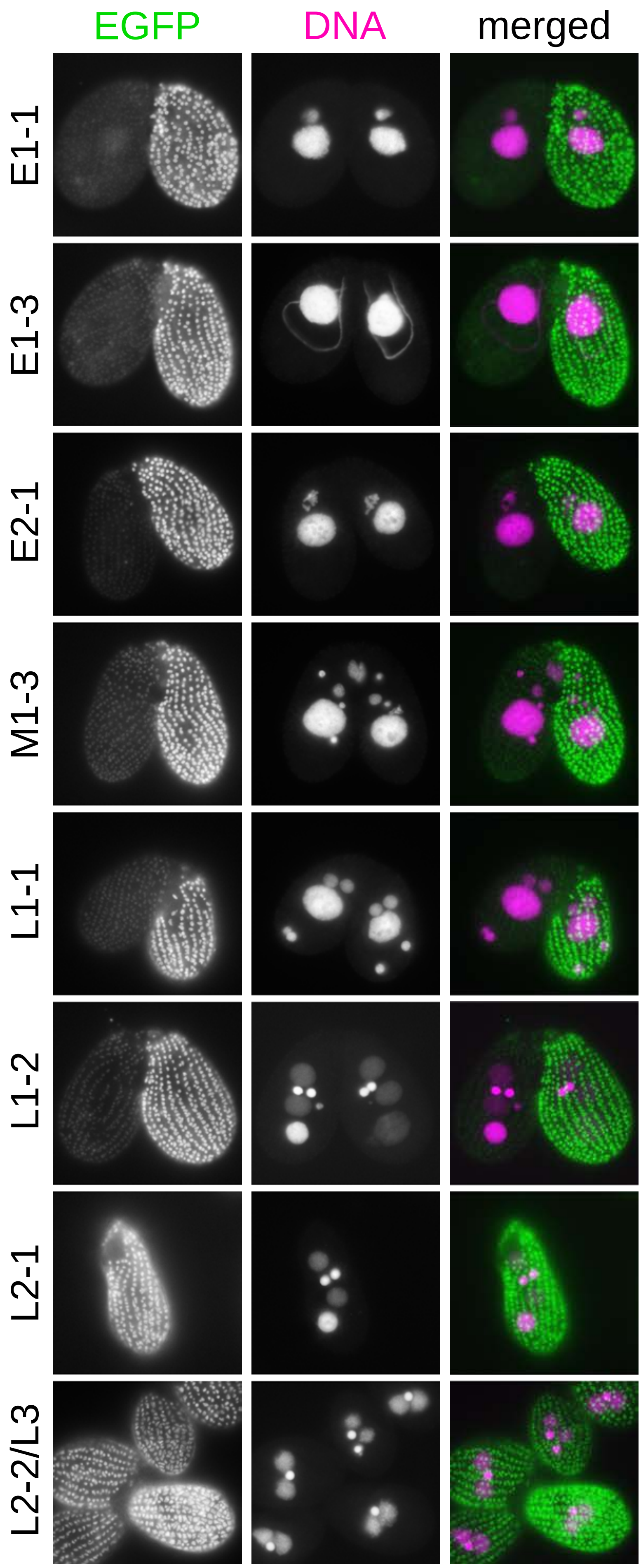

TTHERM_00442420
GenBank; XP_001033155.3
Gene name; *JUB3*

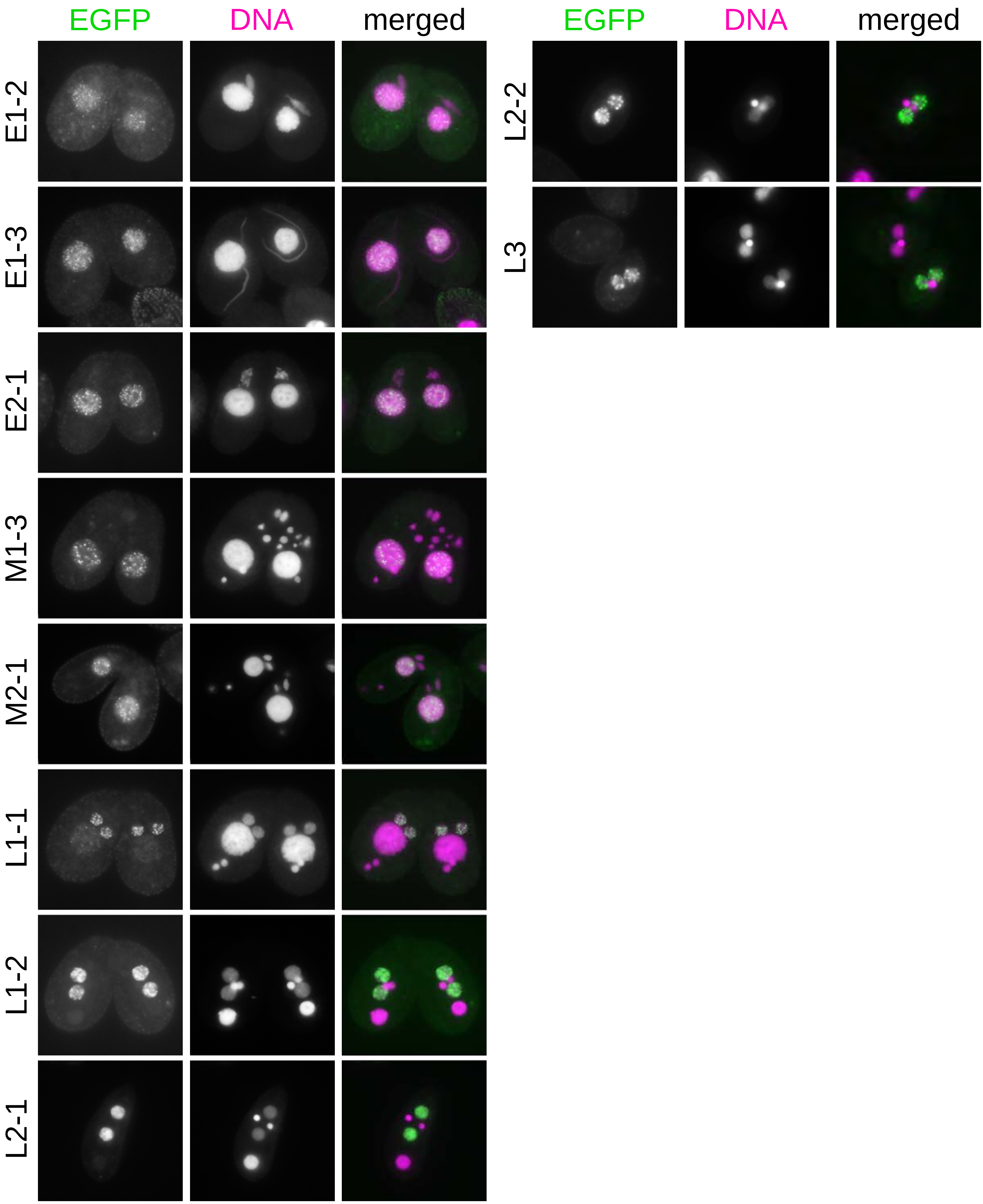

TTHERM_00471000
GenBank; XP_001032990.1

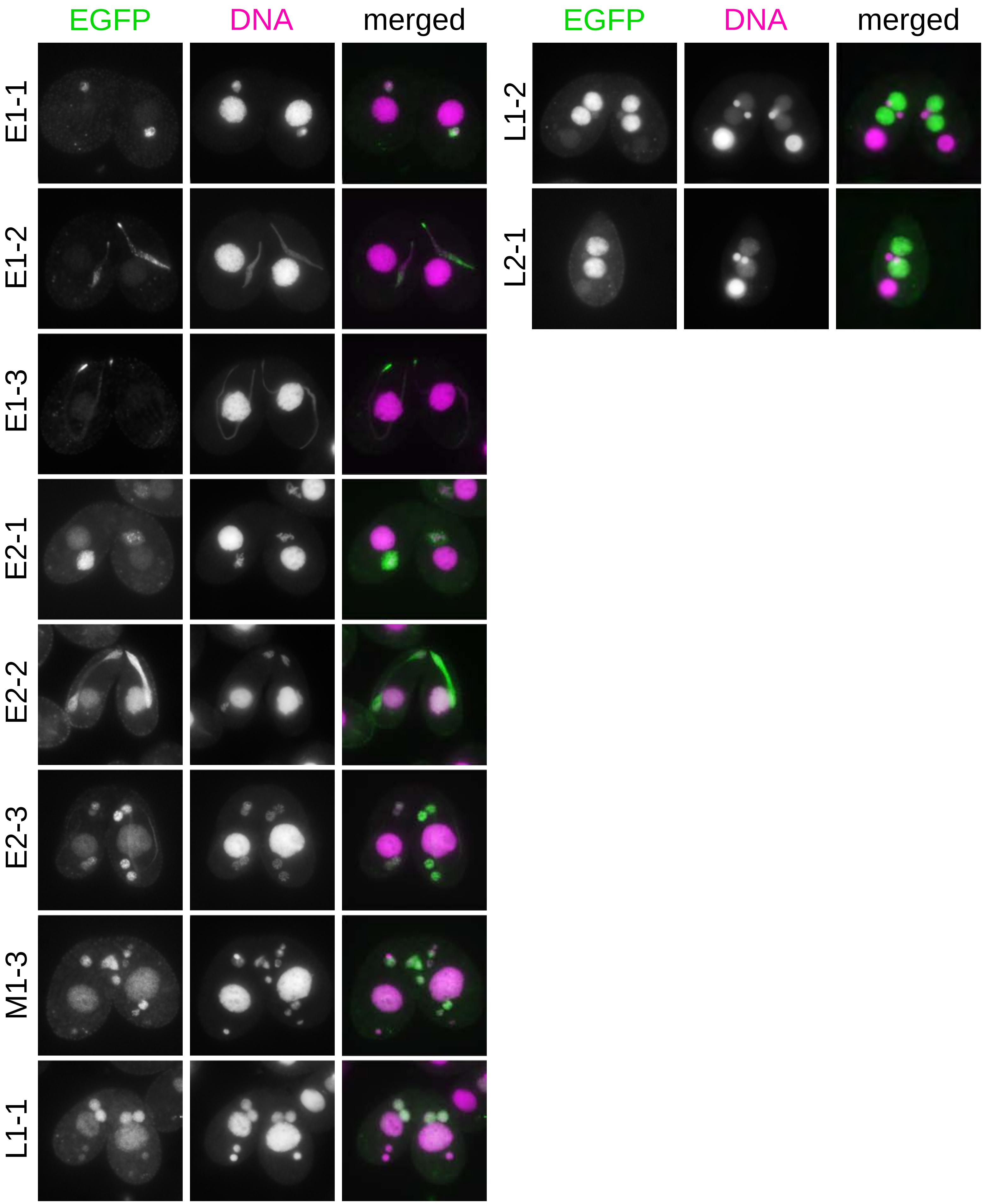

TTHERM_00471720
GenBank; XP_001033056.3

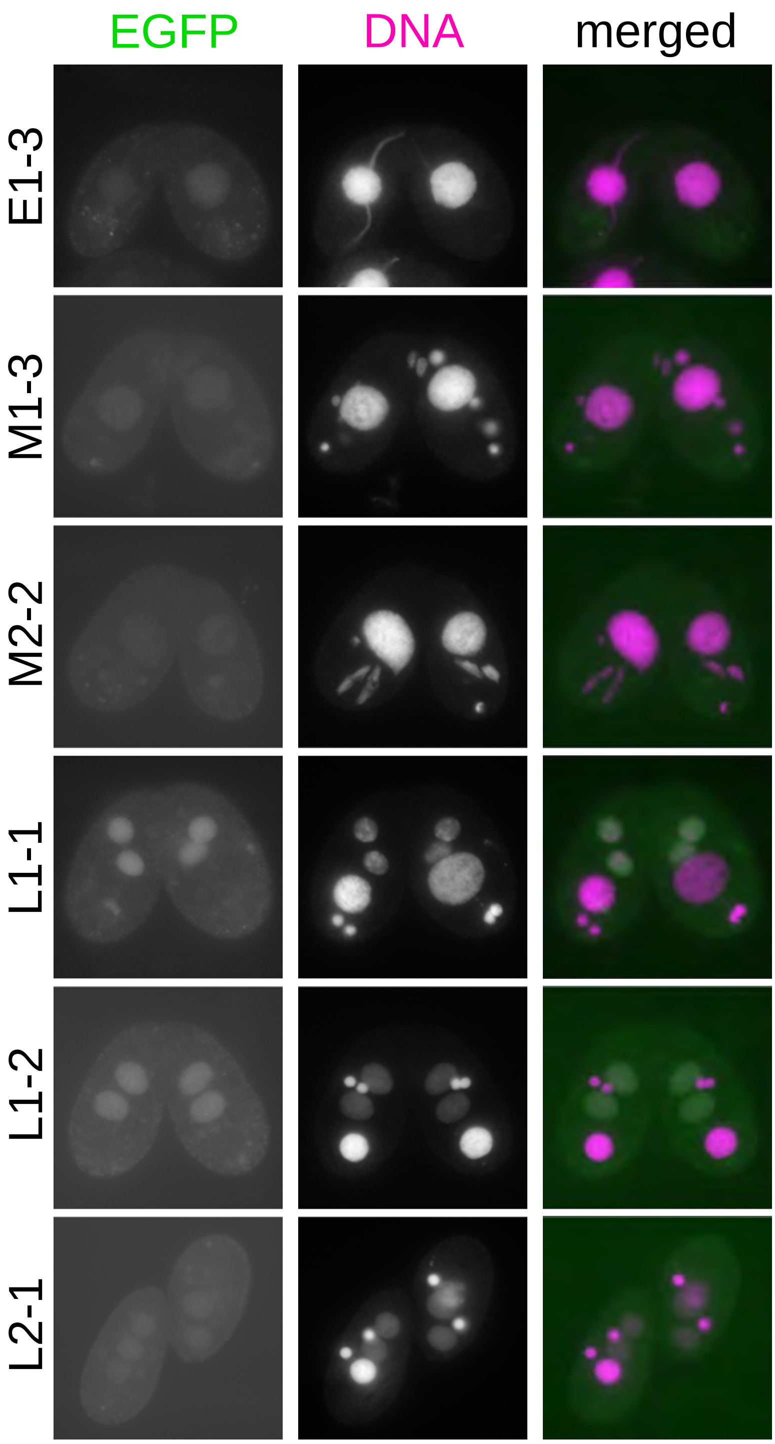

TTHERM_00474920
GenBank; XP_001023974.1

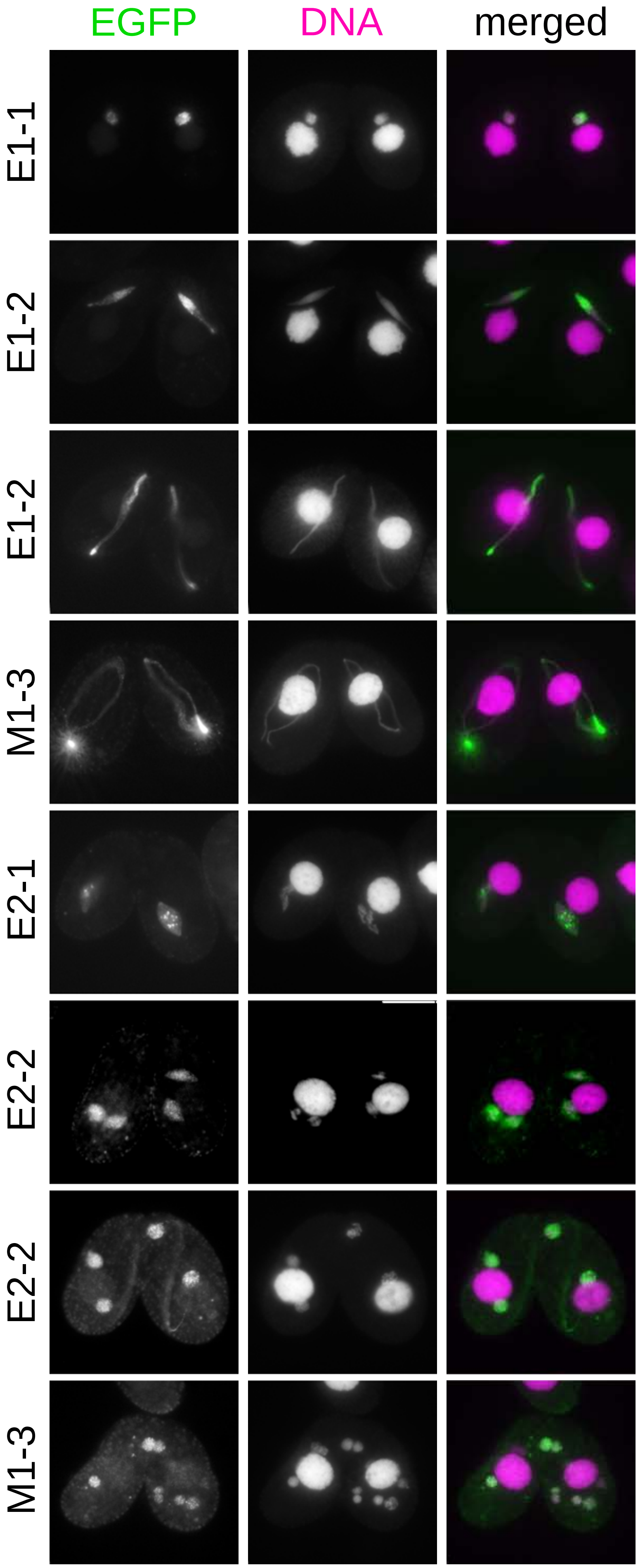

TTHERM_00497670
GenBank; XP_001027955.2
Gene name; *COI17*

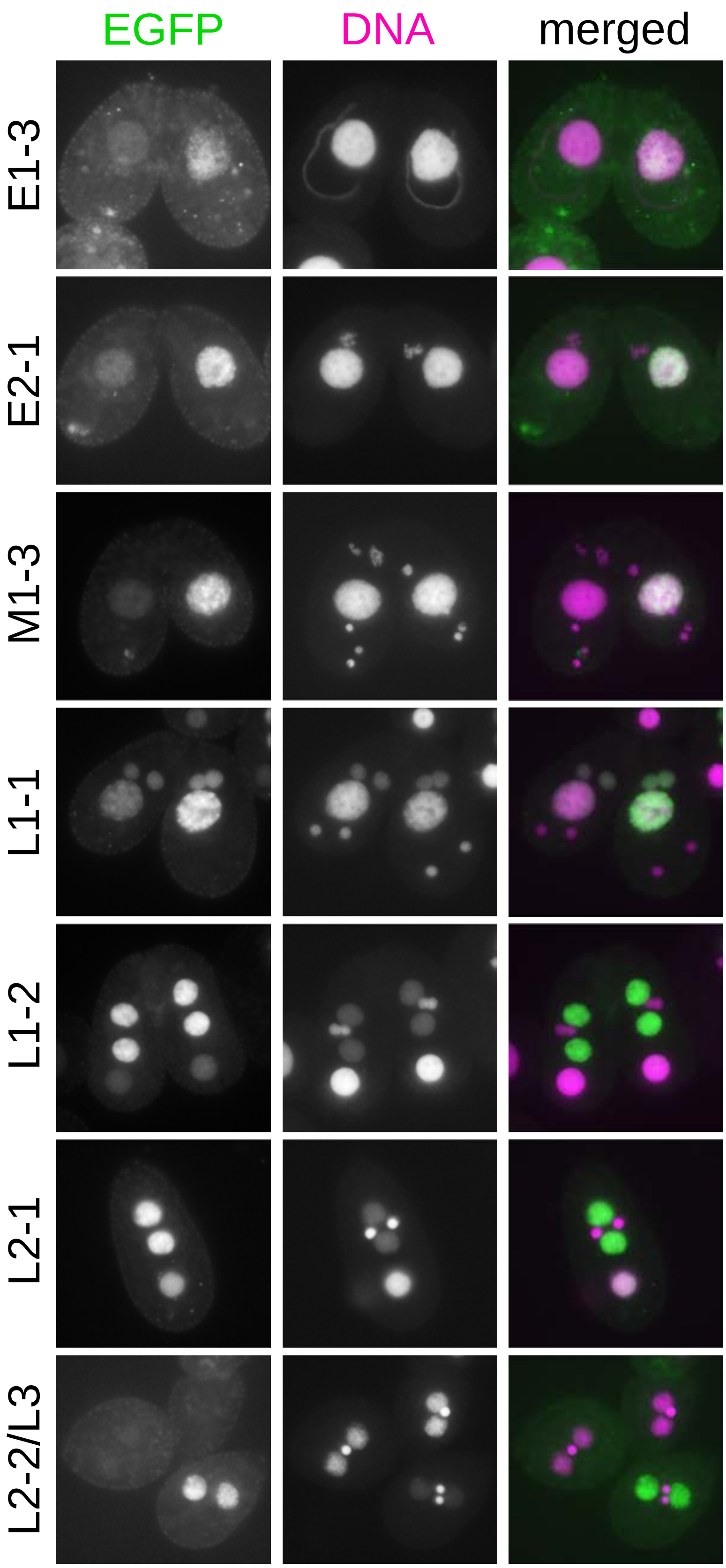

TTHERM_00499370
GenBank; XP_001022195.1

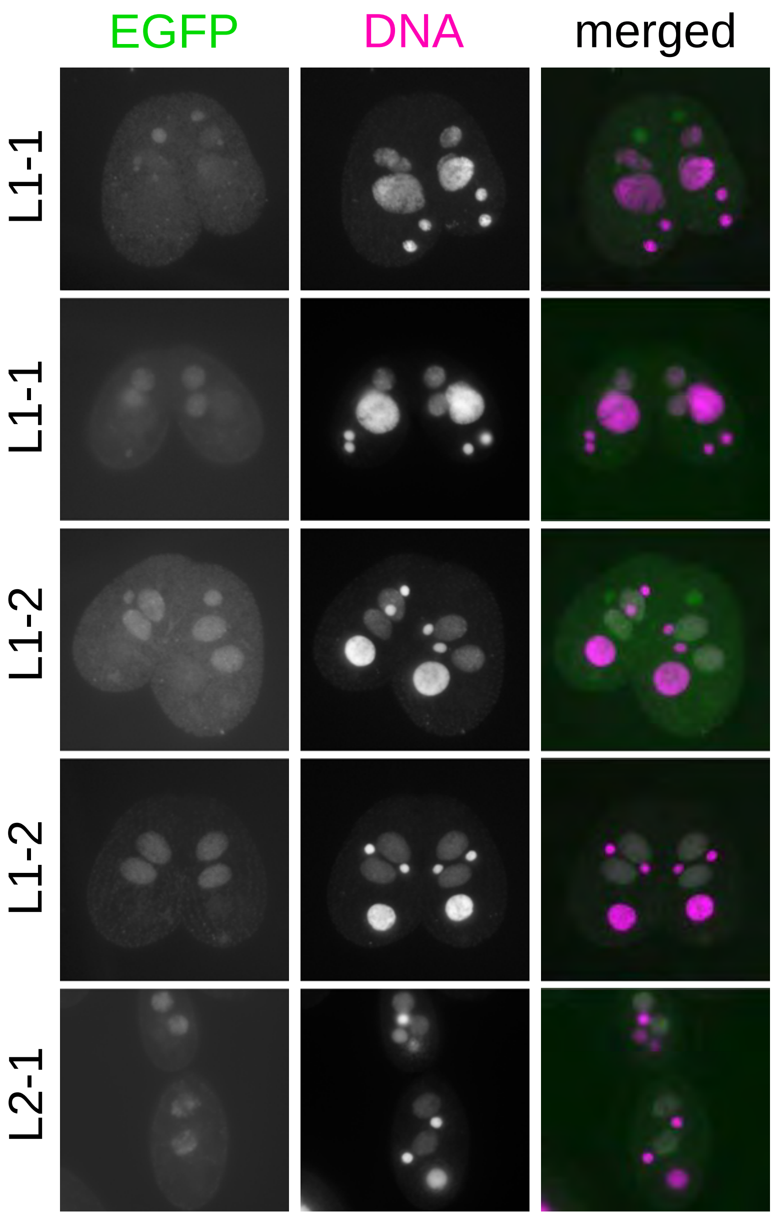

TTHERM_00522820
GenBank; XP_001014460.2
Gene name; *COI4*

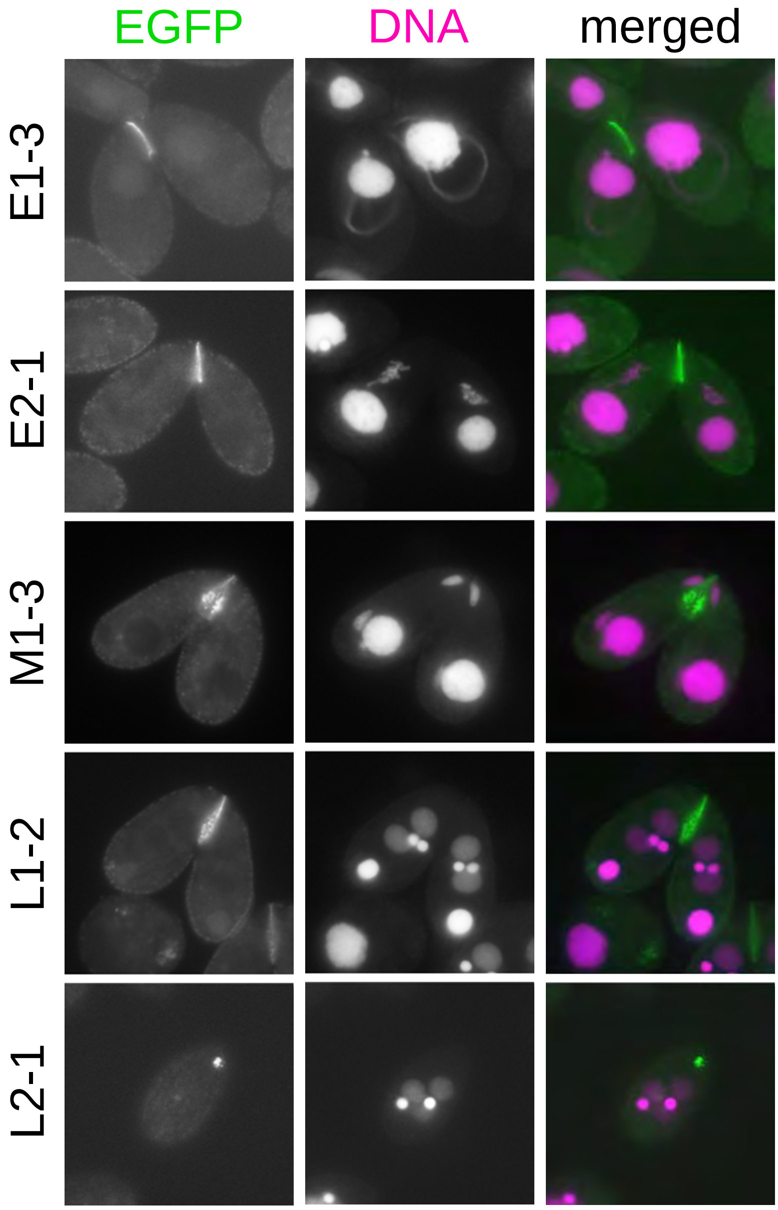

TTHERM_00526270a*
GenBank; XP_001028038.1
Gene name; *COI3*

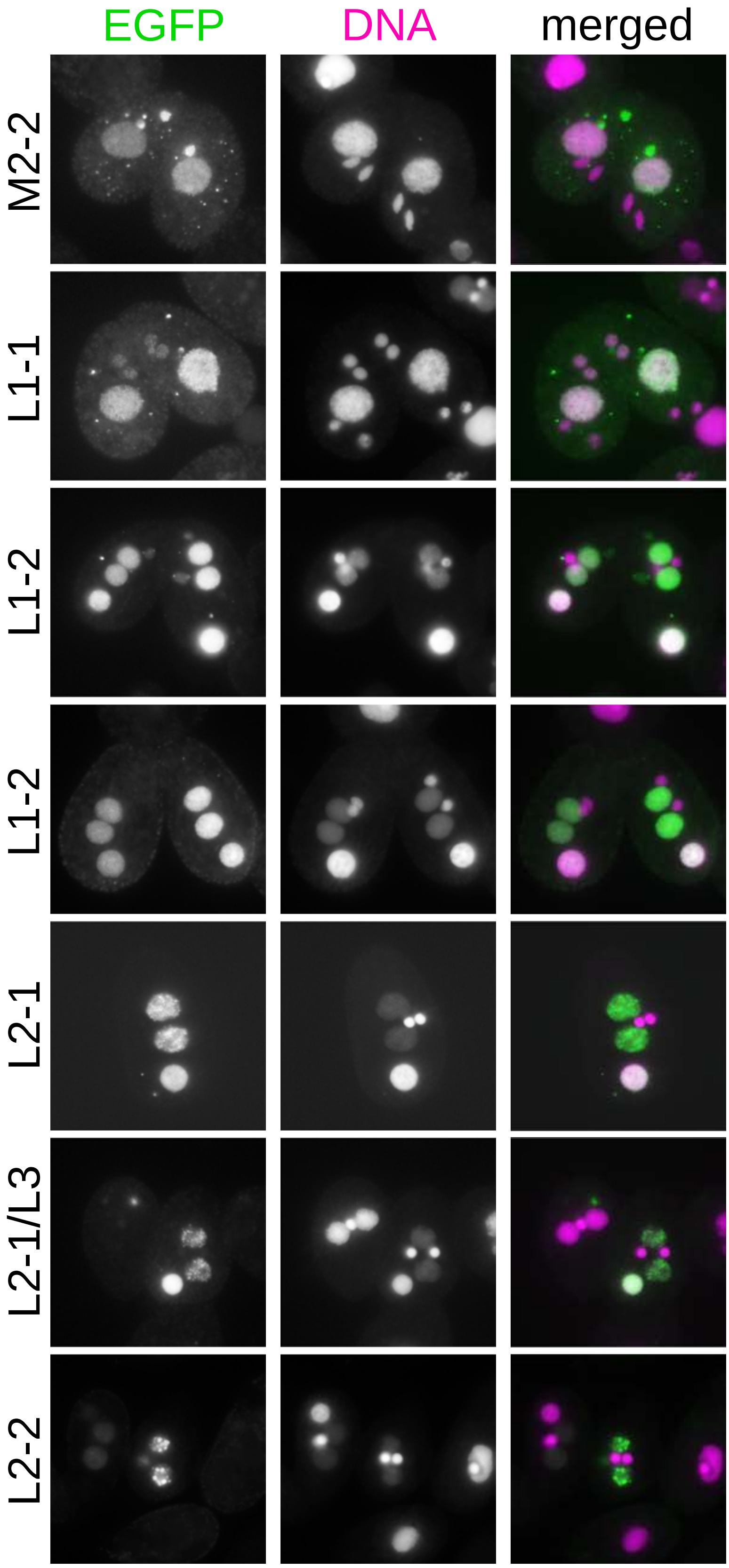

TTHERM_00526270b*
GenBank; XP_001028038.1

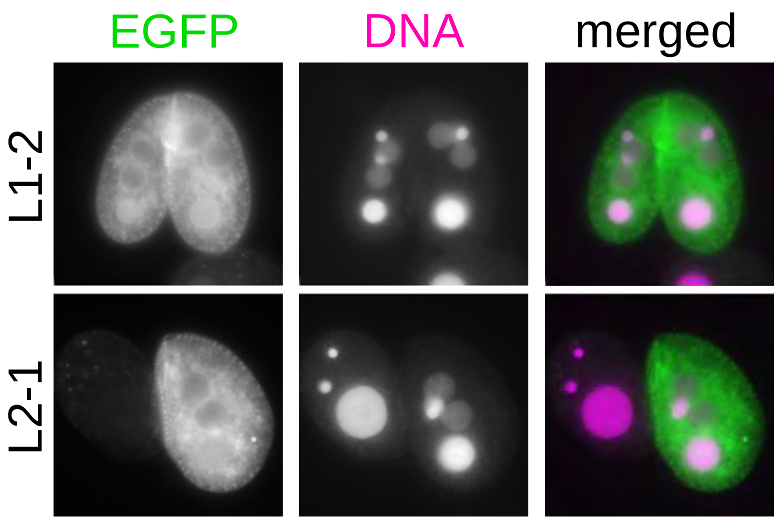

TTHERM_00564480
GenBank; XP_001022047.3
Gene name; *COI16*

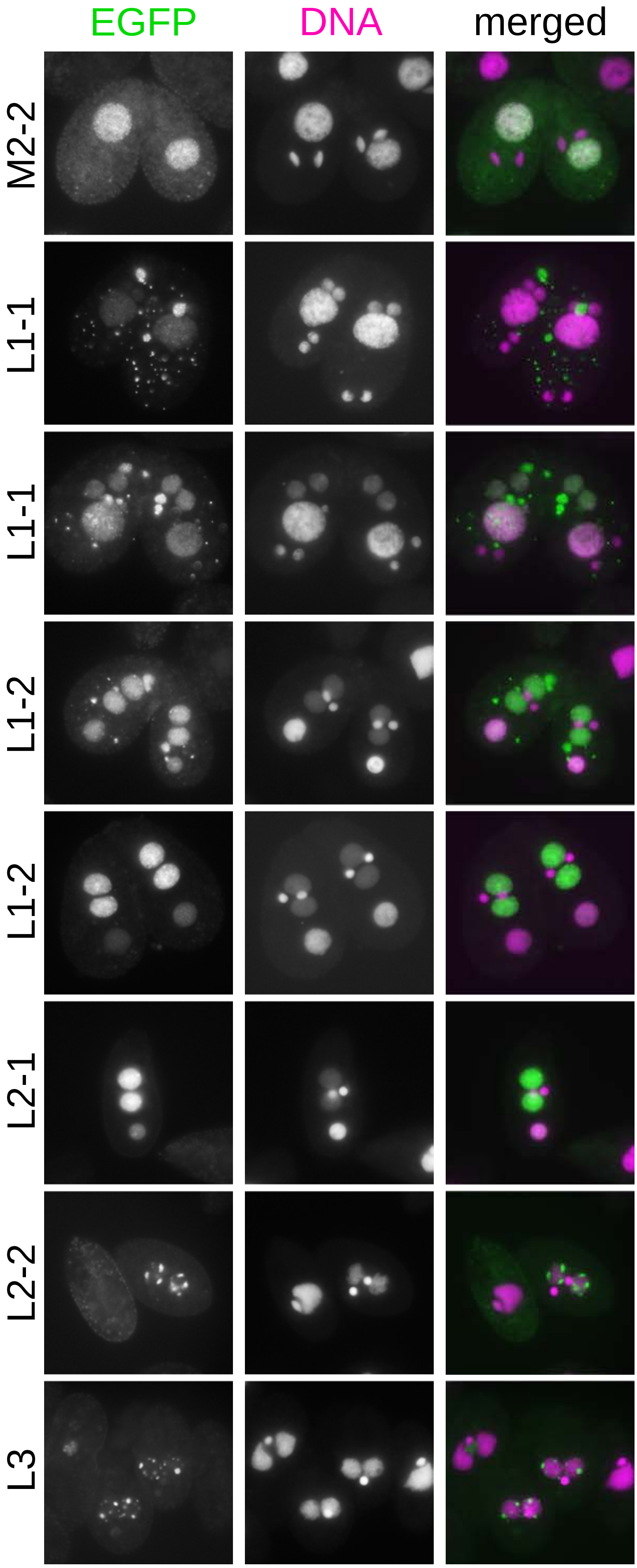

TTHERM_00572190
GenBank; XP_001027725.2

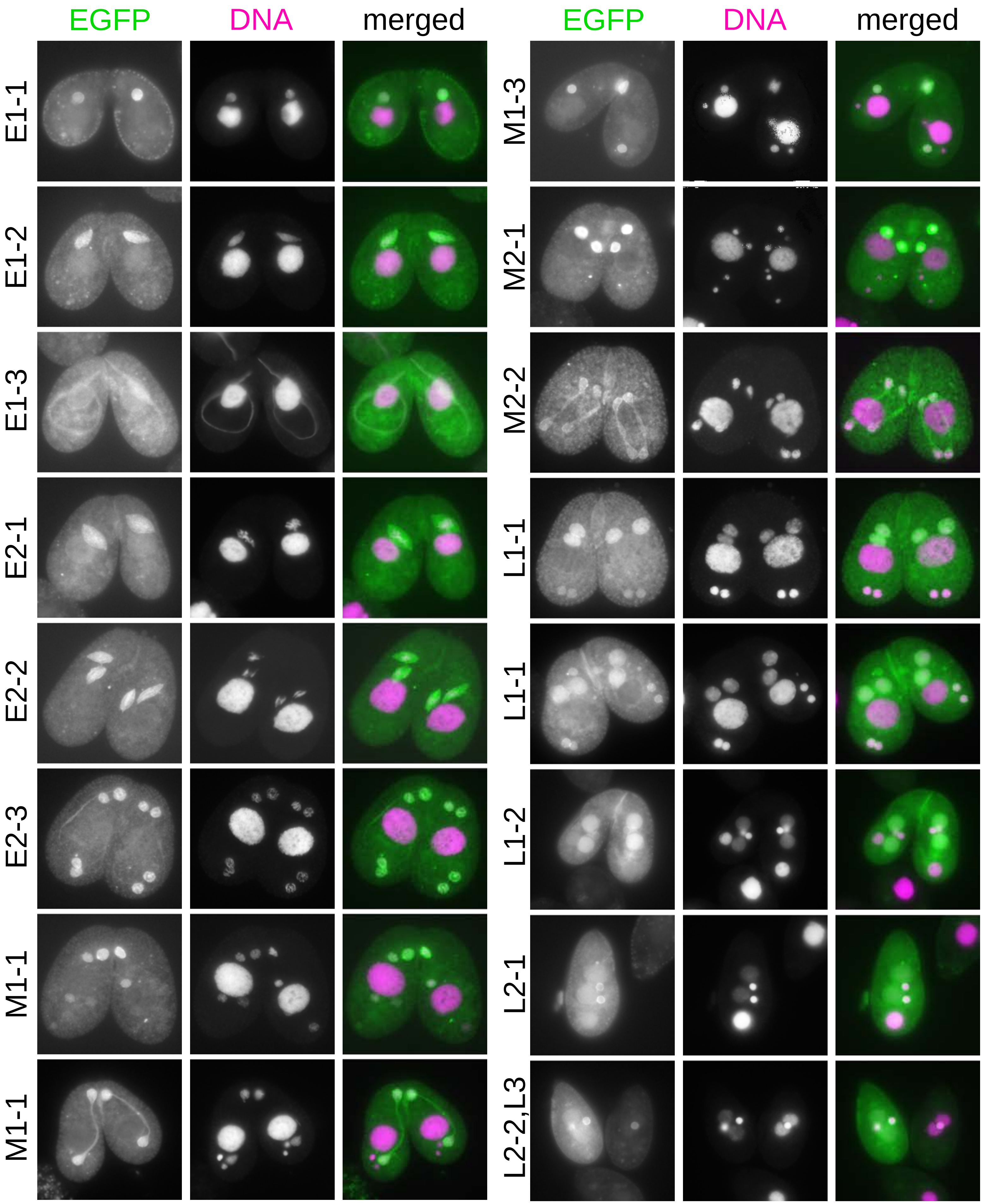

TTHERM_00616290
GenBank; XP_001024699.1
Gene name; *JUB4*

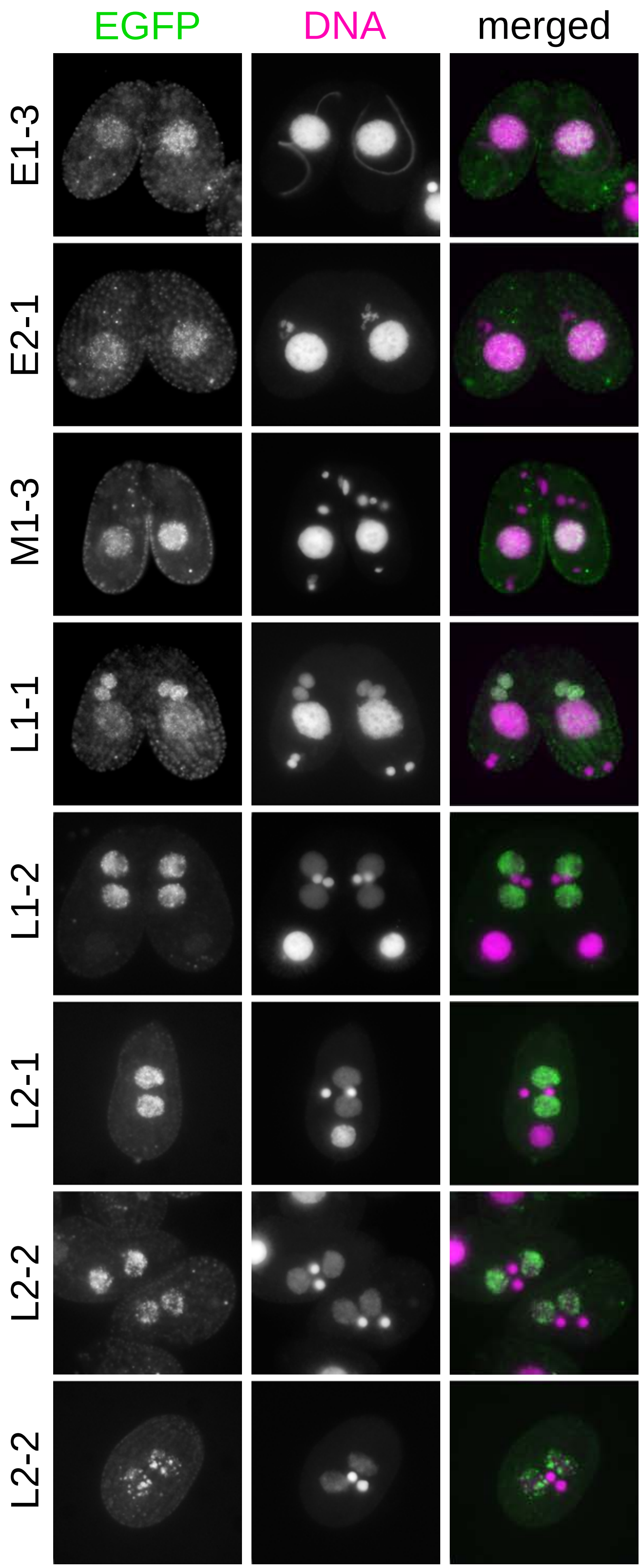

TTHERM_00622830
GenBank; XP_001022508.1

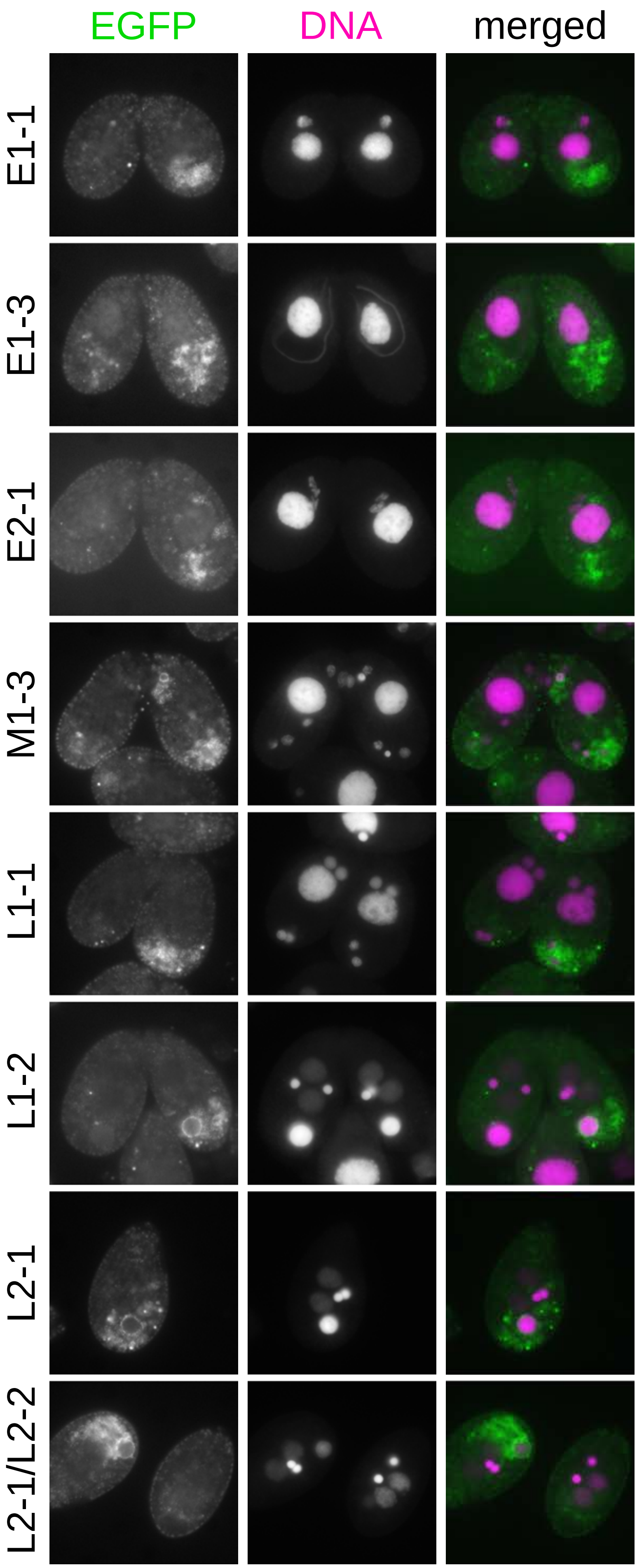

TTHERM_00637050
GenBank; XP_001032366.1

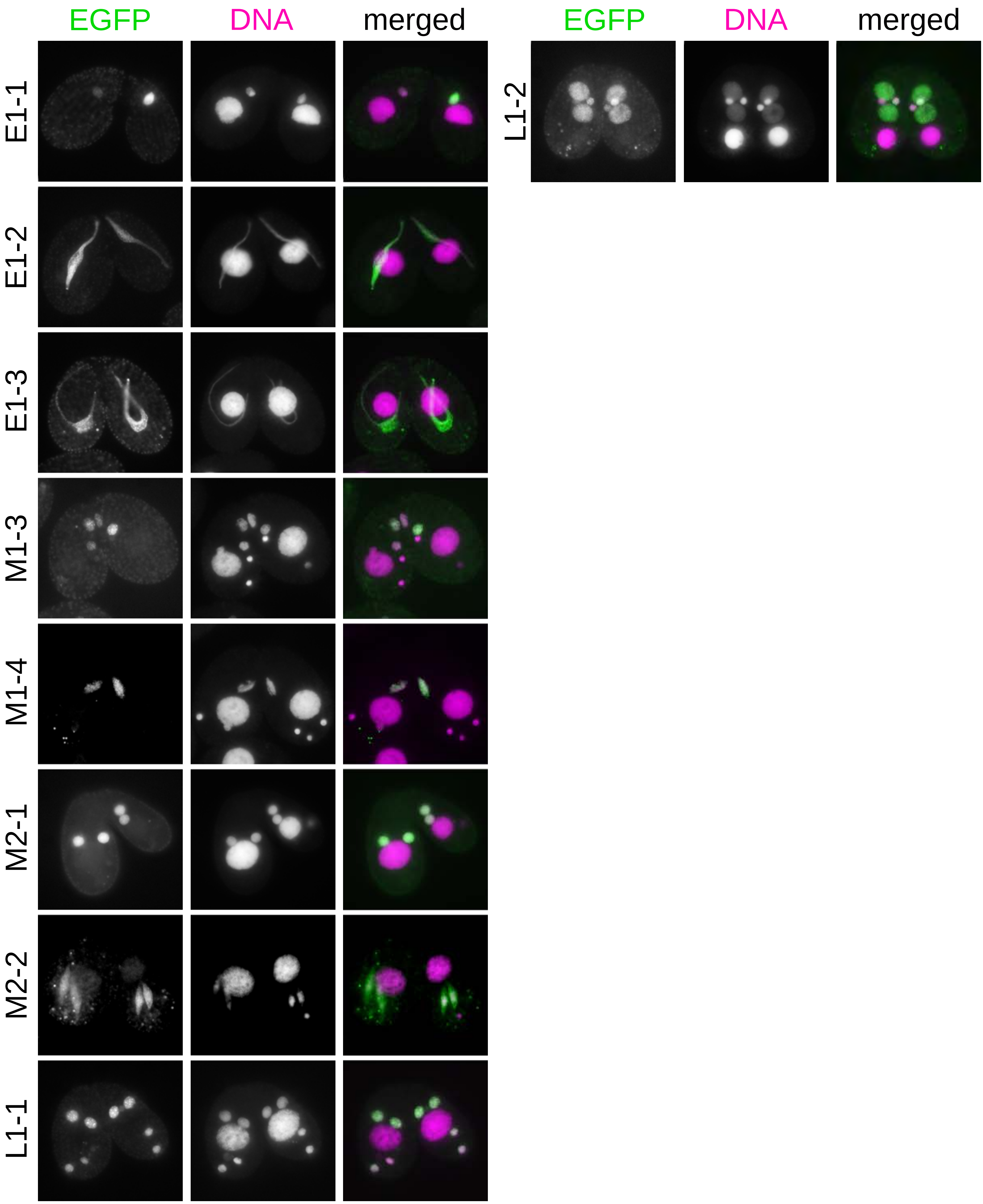

TTHERM_00649180
GenBank; XP_001032312.3

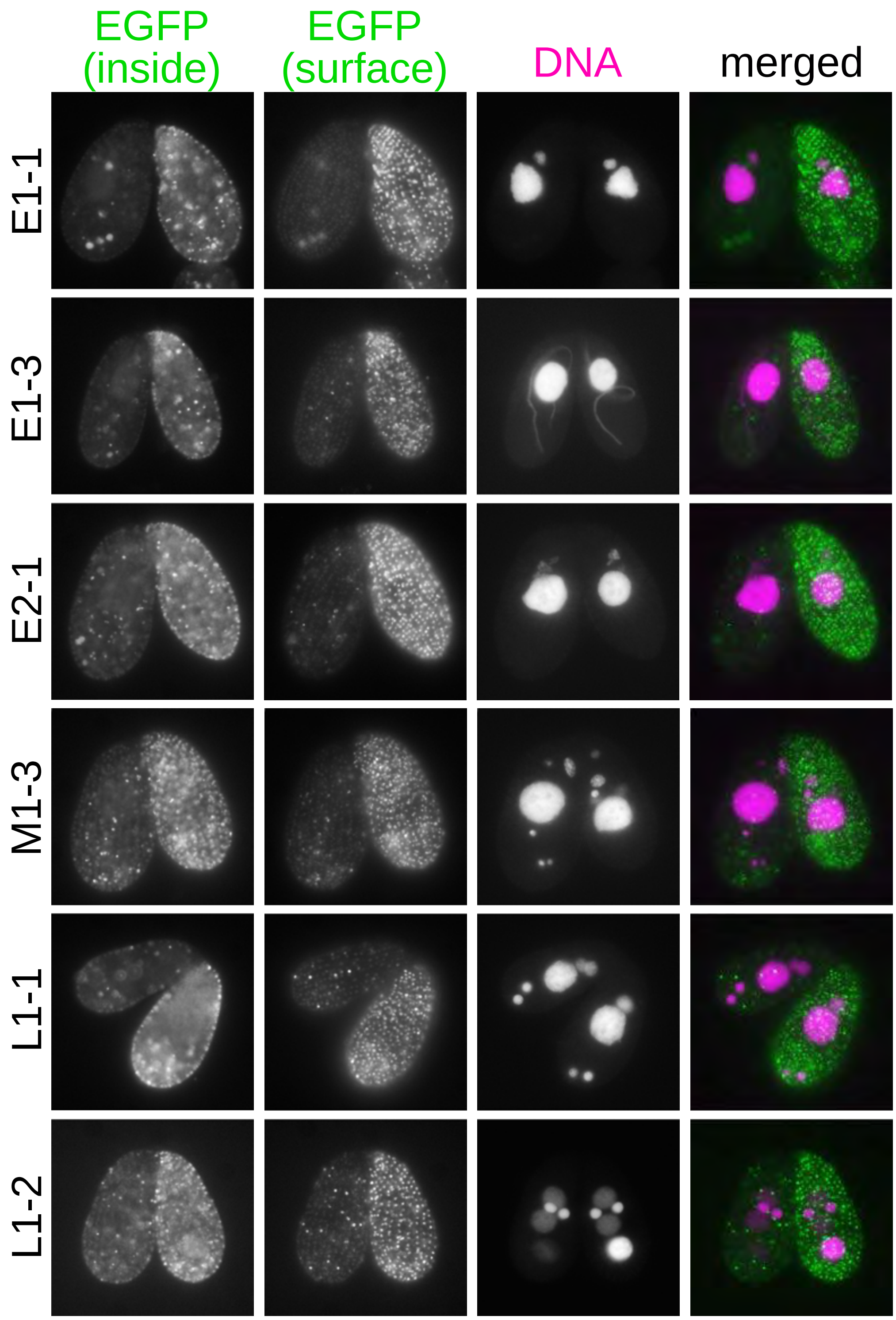

TTHERM_00693080
GenBank; XP_001023573.1

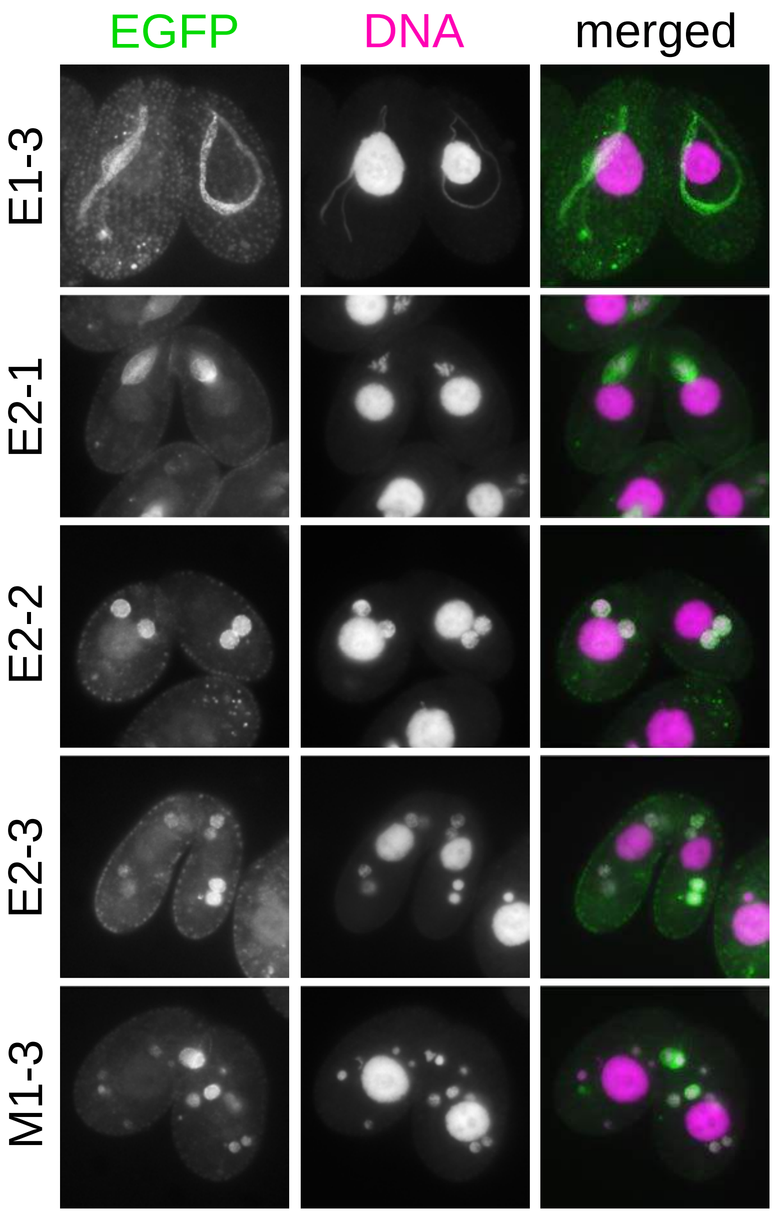

TTHERM_00728900
GenBank; XP_001022676.1

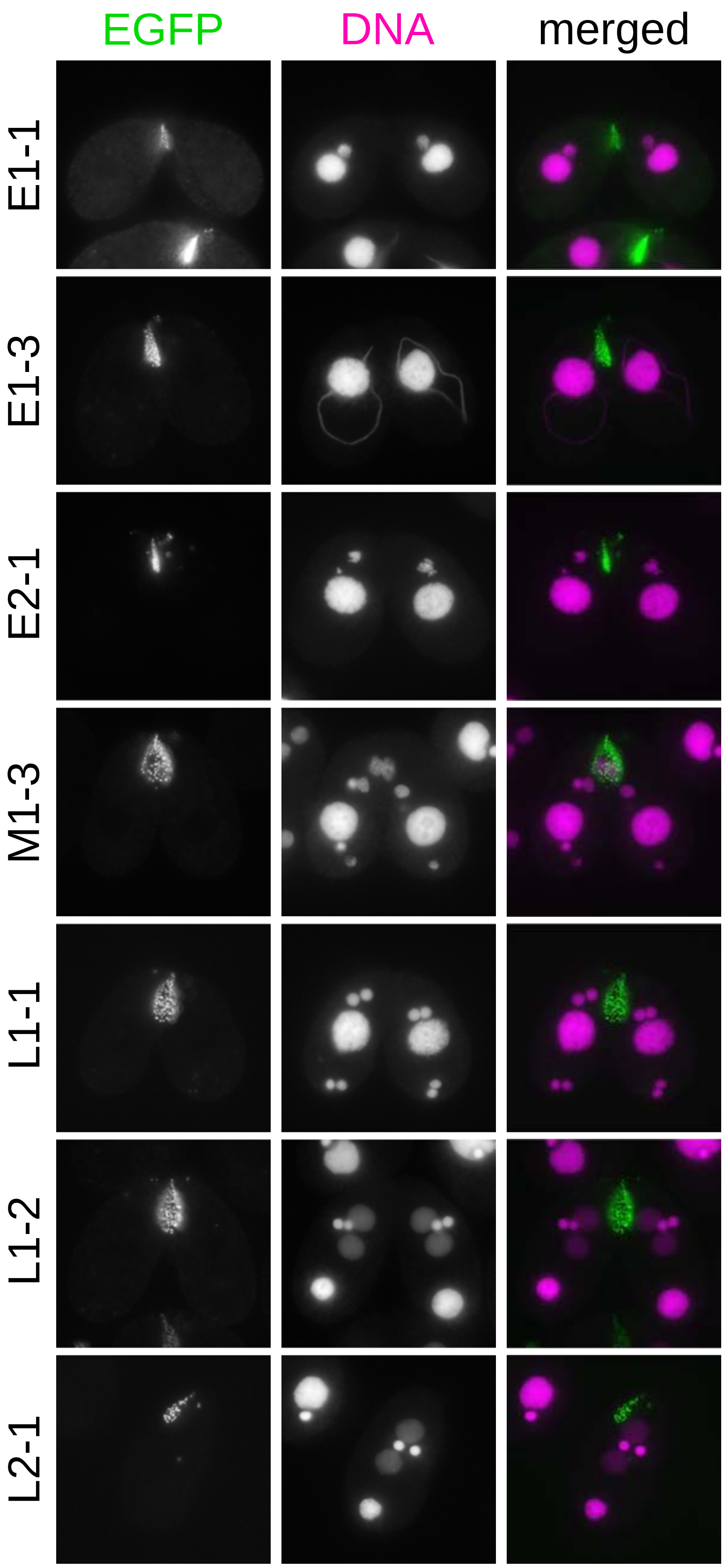

TTHERM_00825660
GenBank; XP_001031434.3

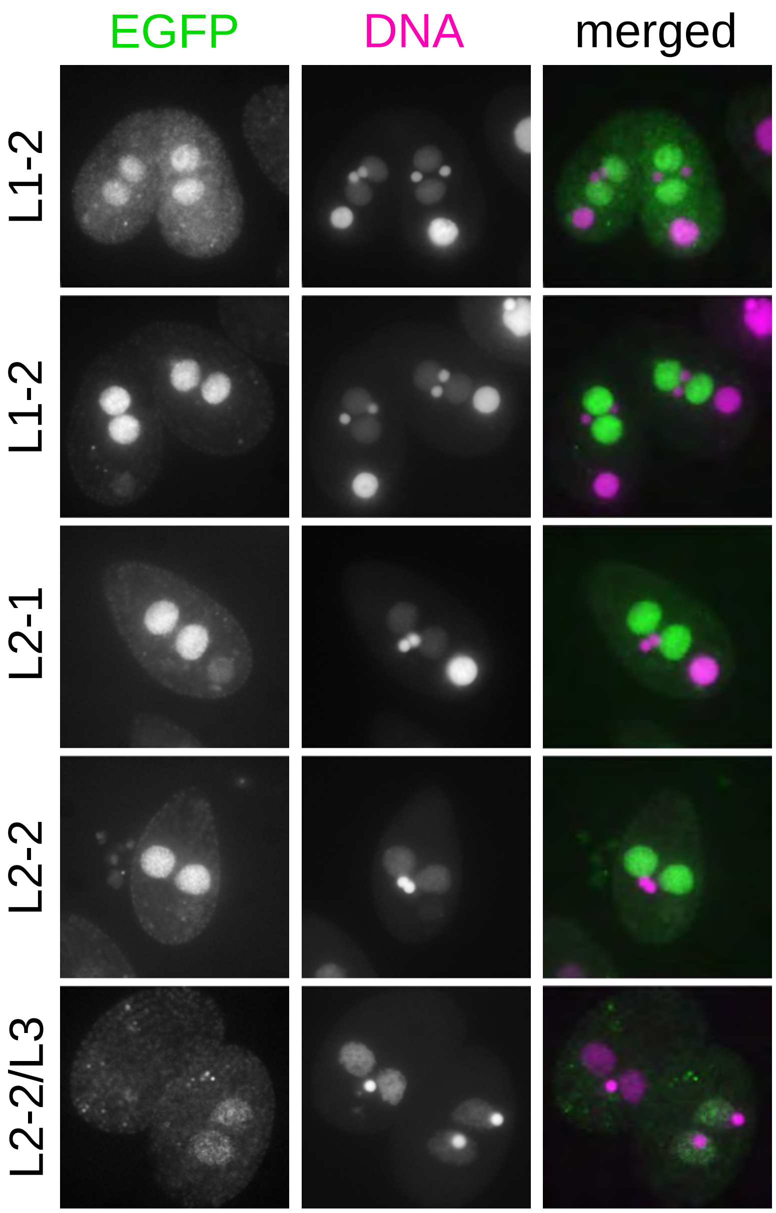

TTHERM_01085480
GenBank; XP_001030381.1
Gene name; *COI19*

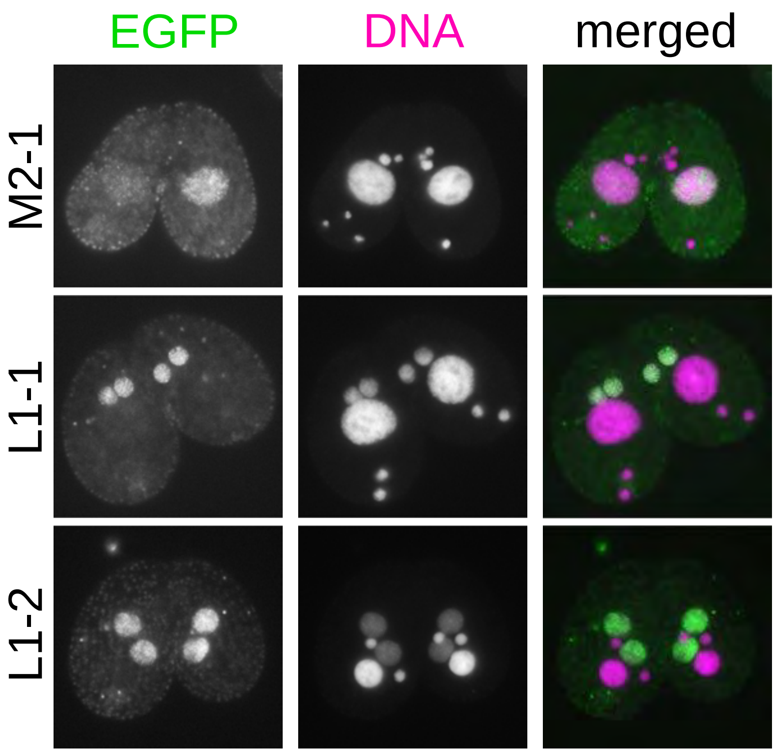

TTHERM_01132870
GenBank; EAS07342.3

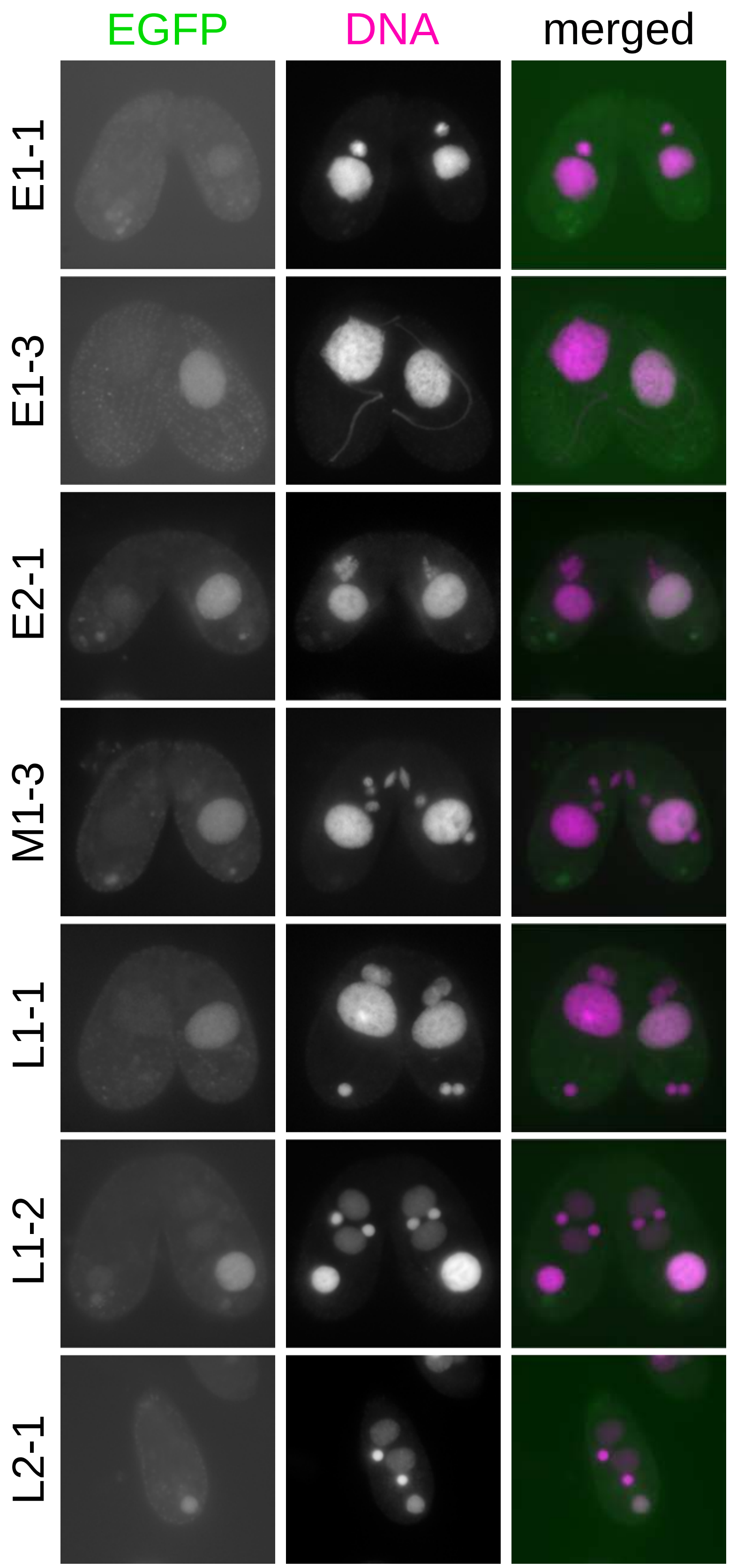

TTHERM_01276320
GenBank; XP_001029843.2
Gene name; *COI1(GWI1)*

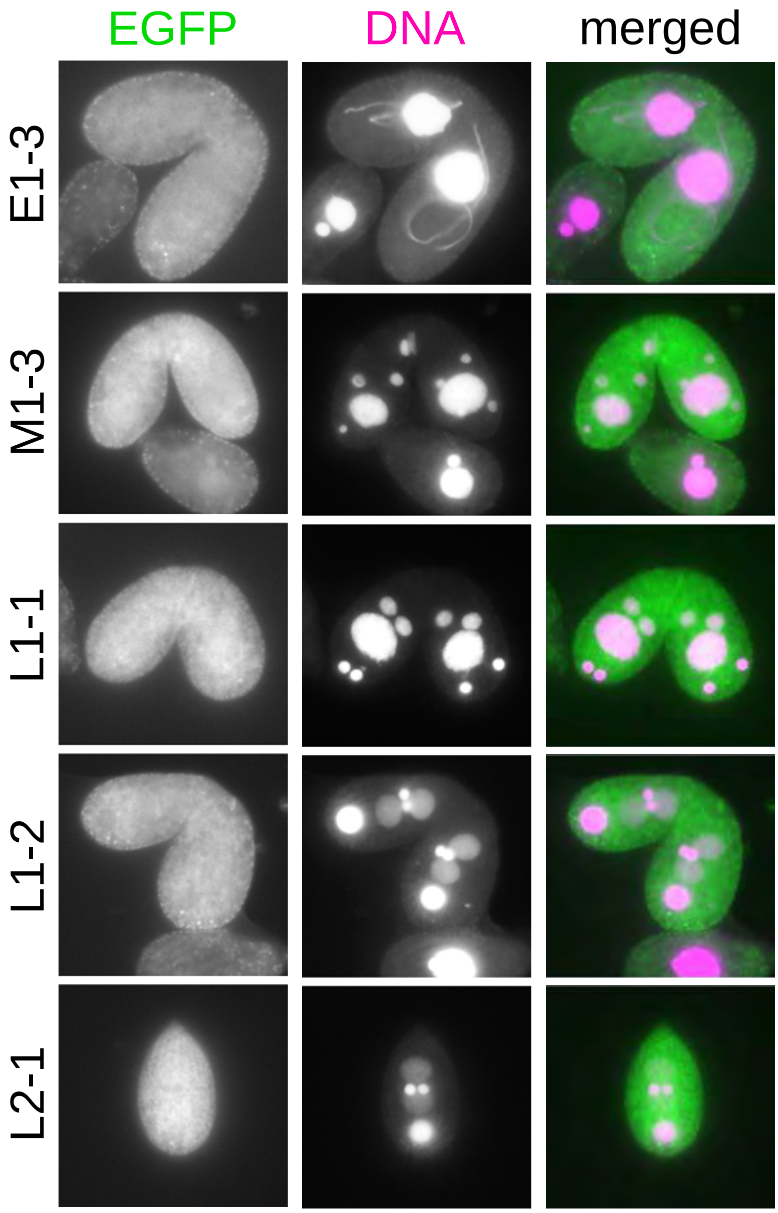

TTHERM_01285910
GenBank; XP_001029830.2
Gene name; *ZFR1*

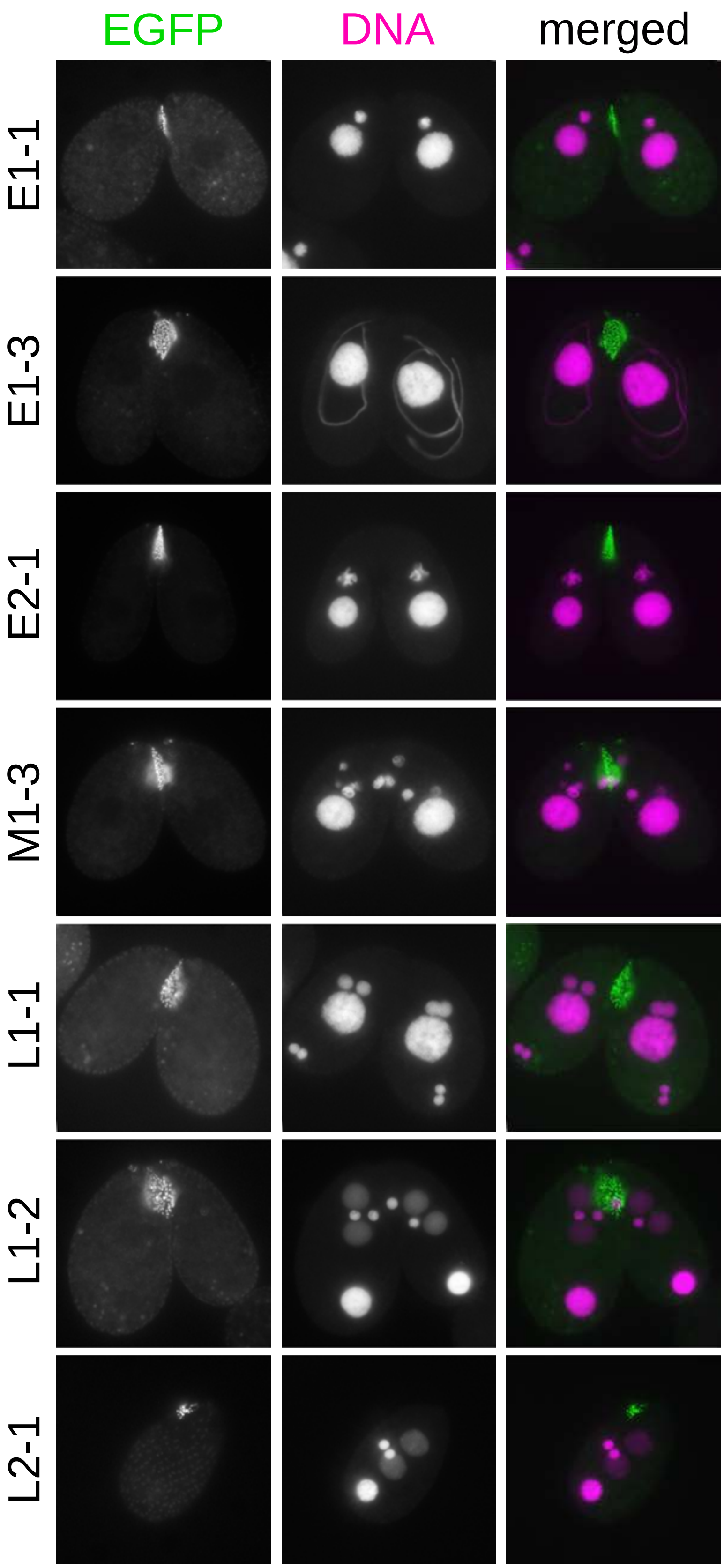

TTHERM_01337400
GenBank; XP_001029724.3
Gene name; *JUB5*

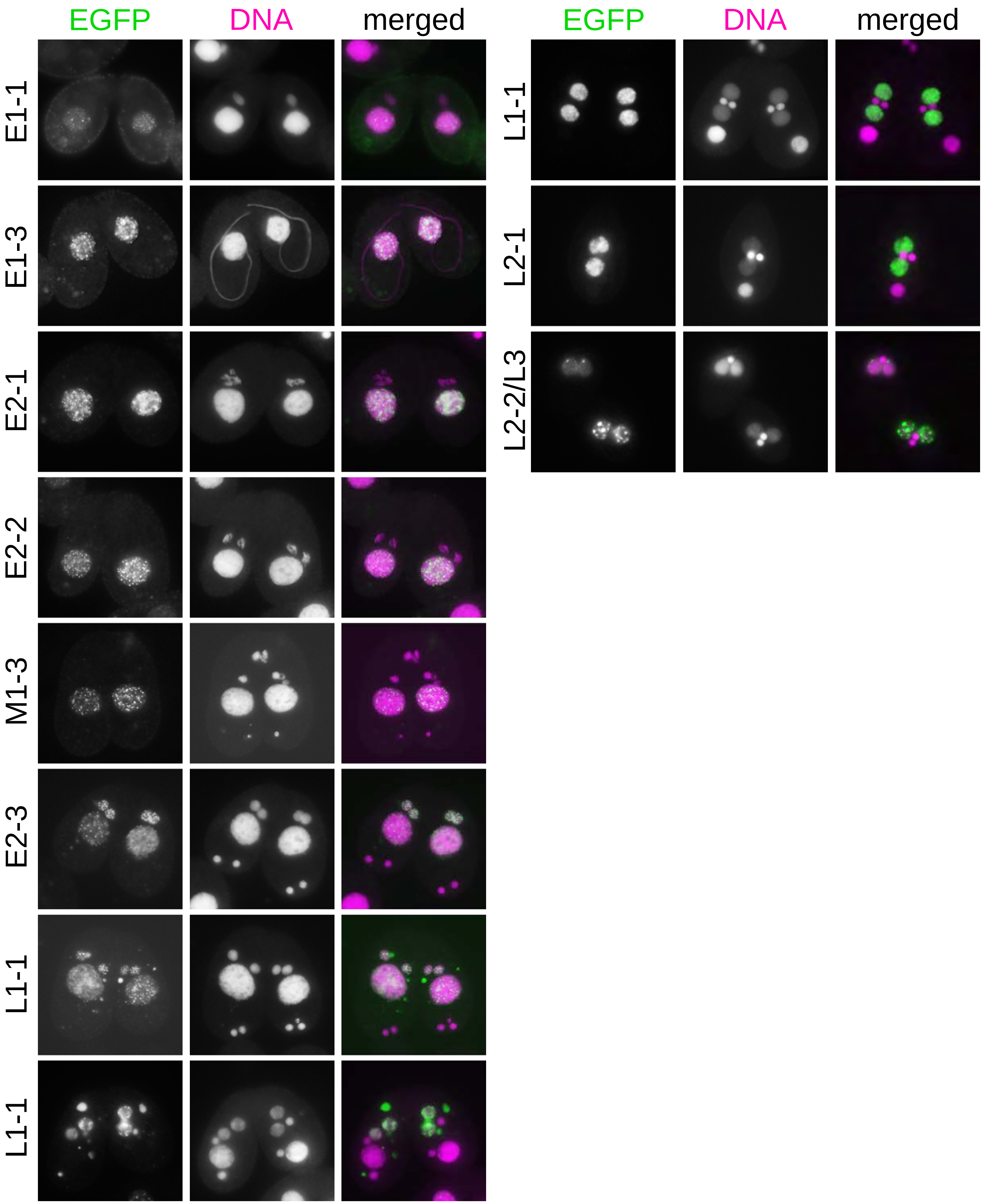

TTHERM_01367700

GenBank; XP_001026268.1

Gene name; *COI10*

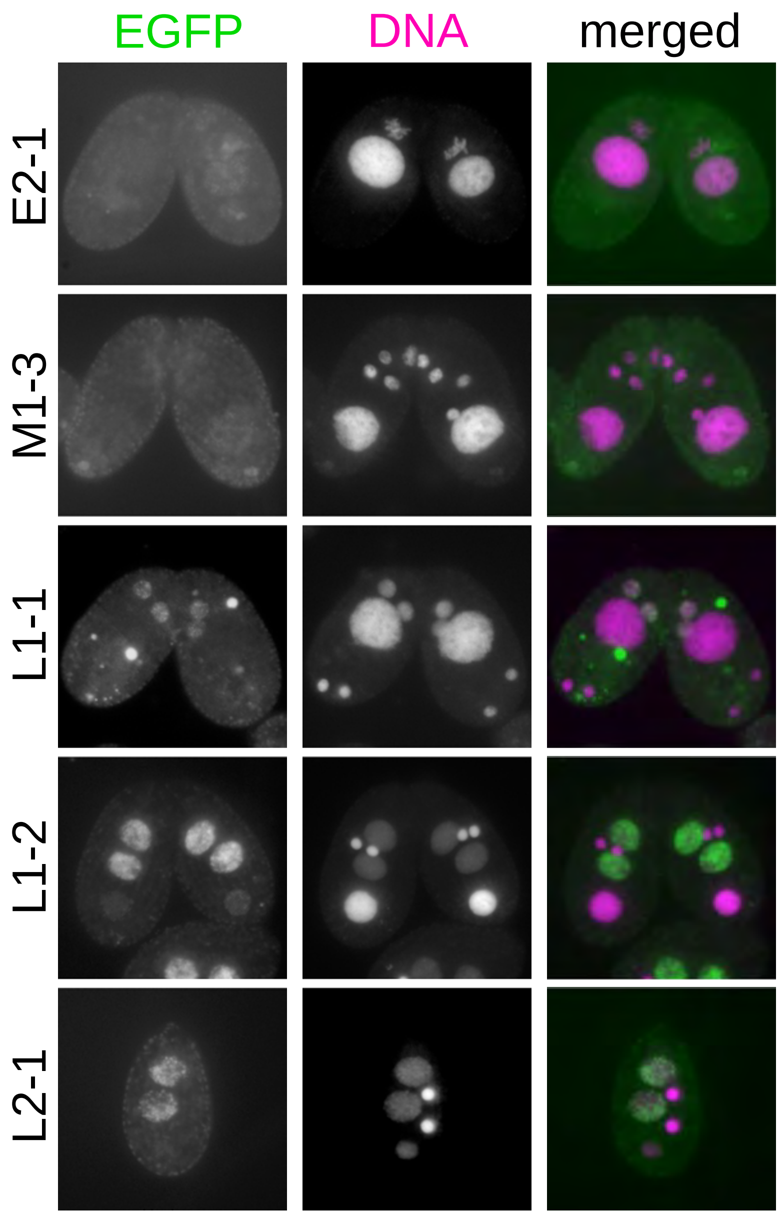

TTHERM_00013110
GenBank; XP_001008290.2

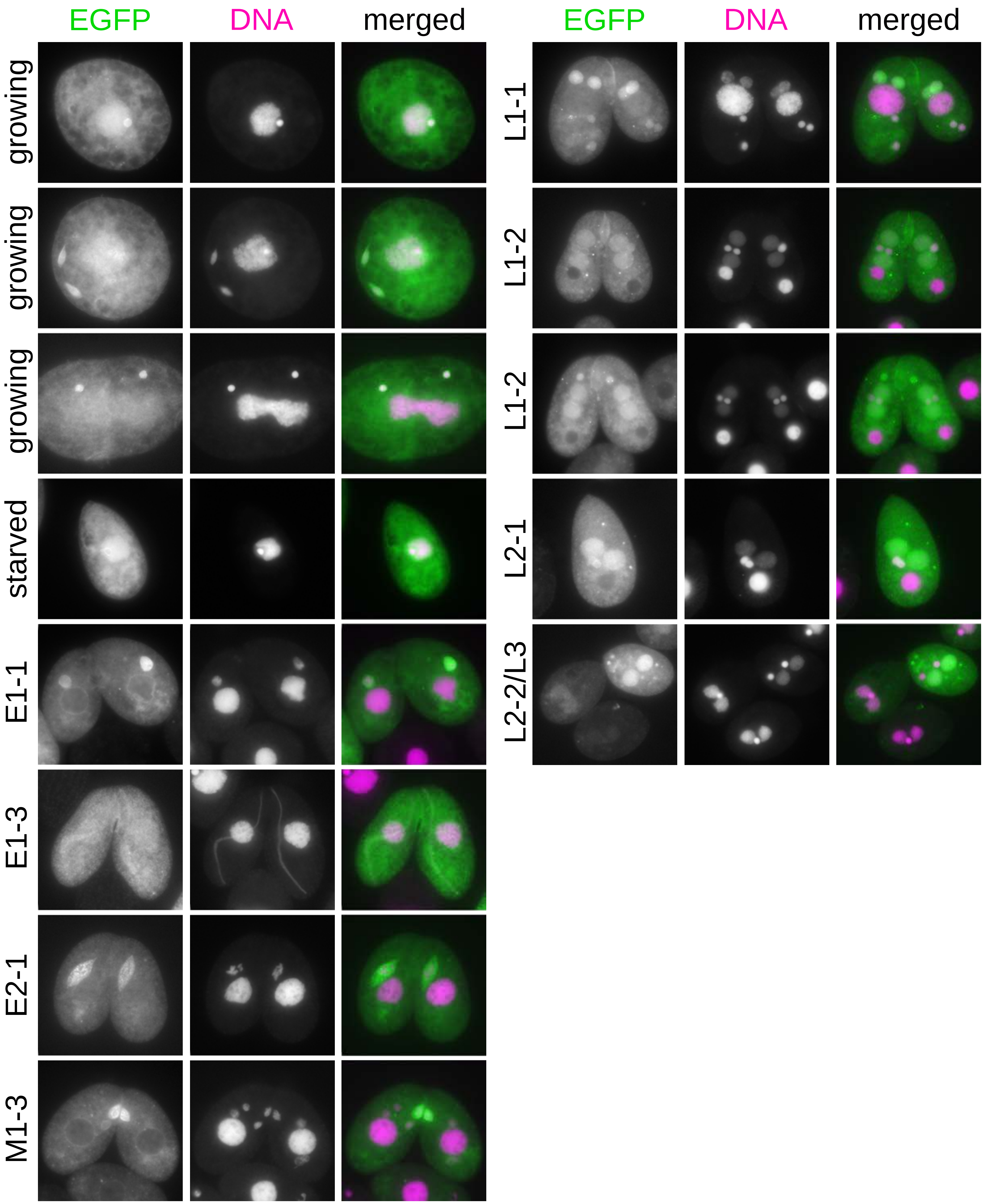

TTHERM_000245499
GenBank; EWS72076.1

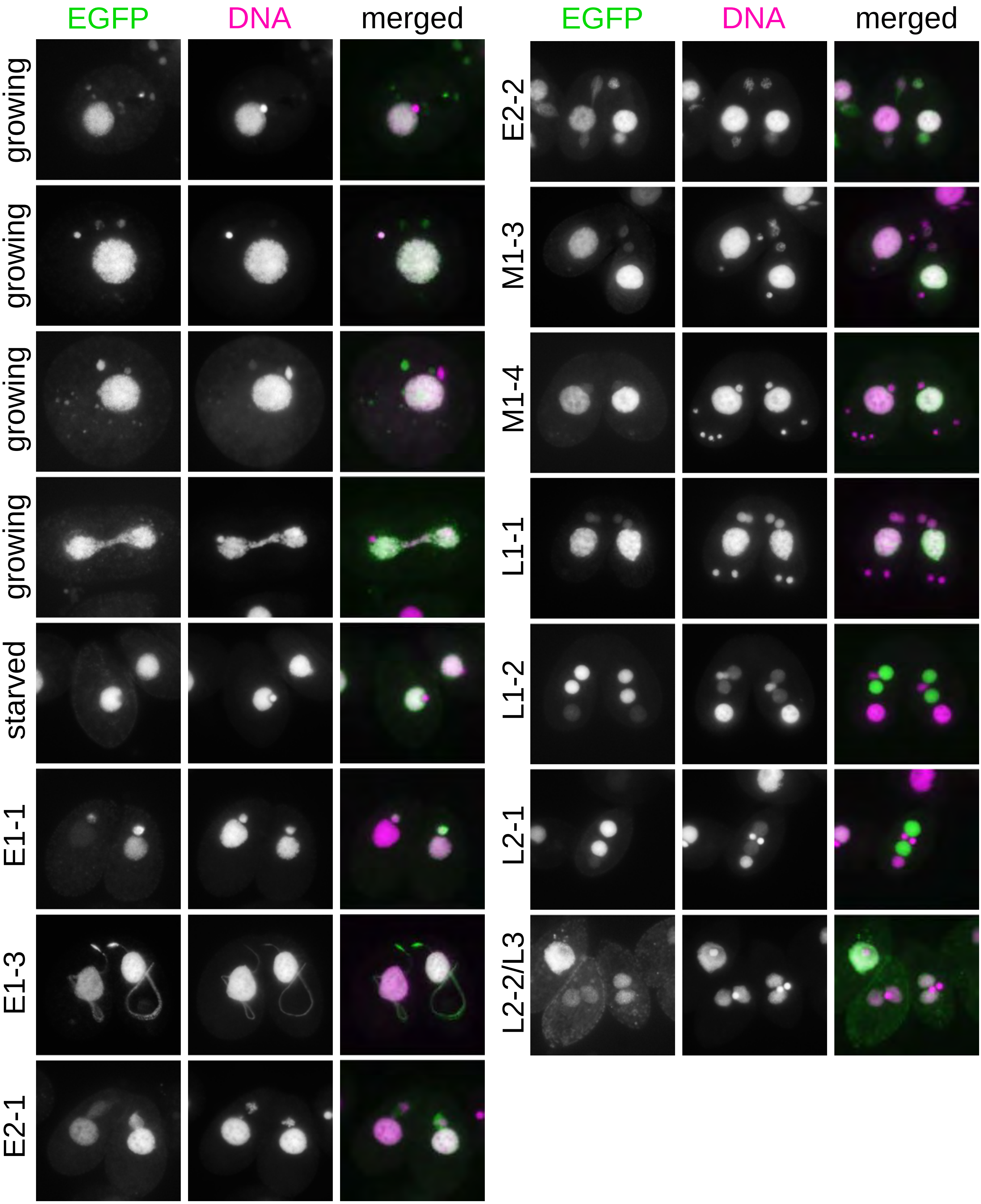

TTHERM_00028580
GenBank; XP_976854.1

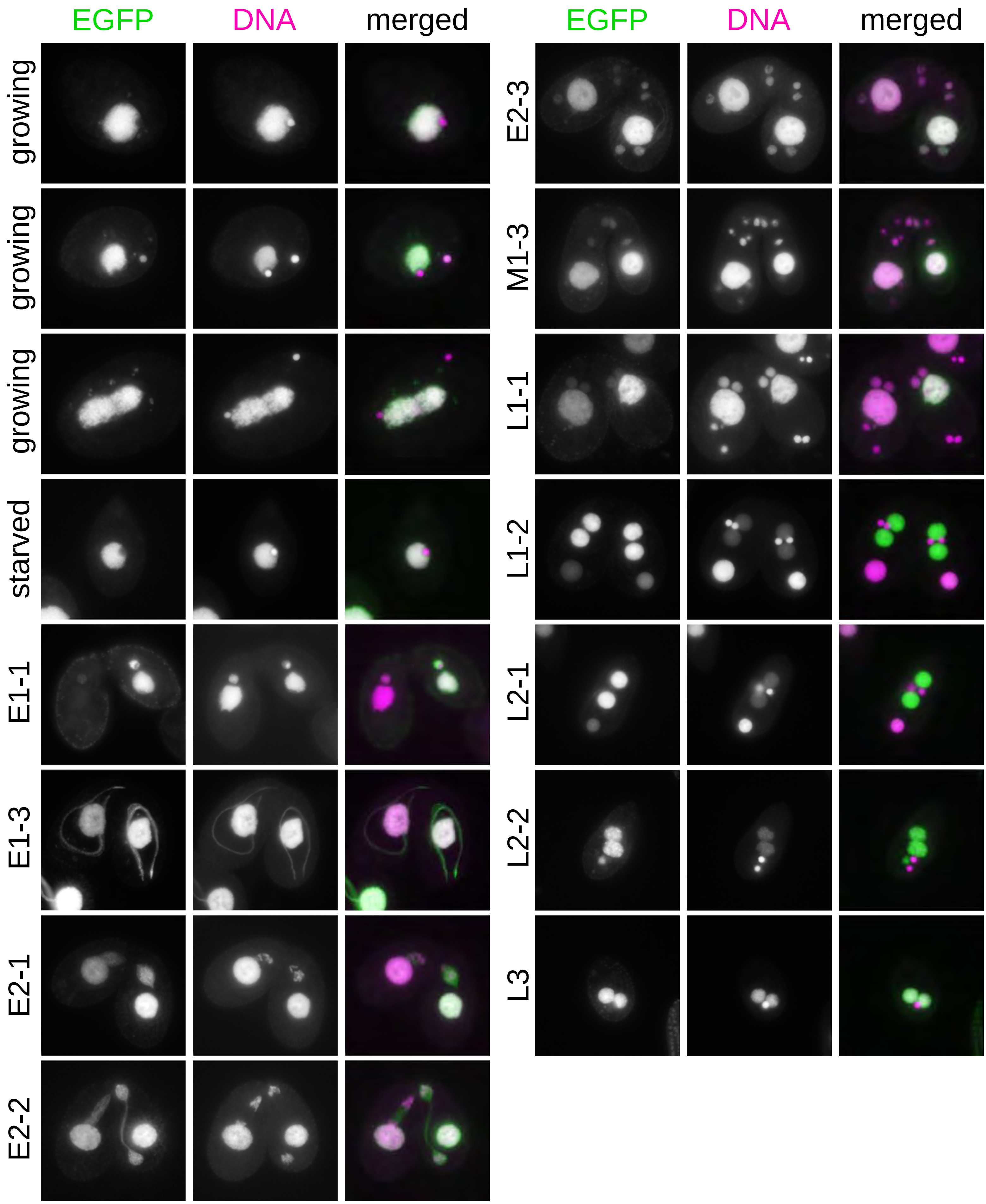

TTHERM_00091510
GenBank; XP_001012803.1

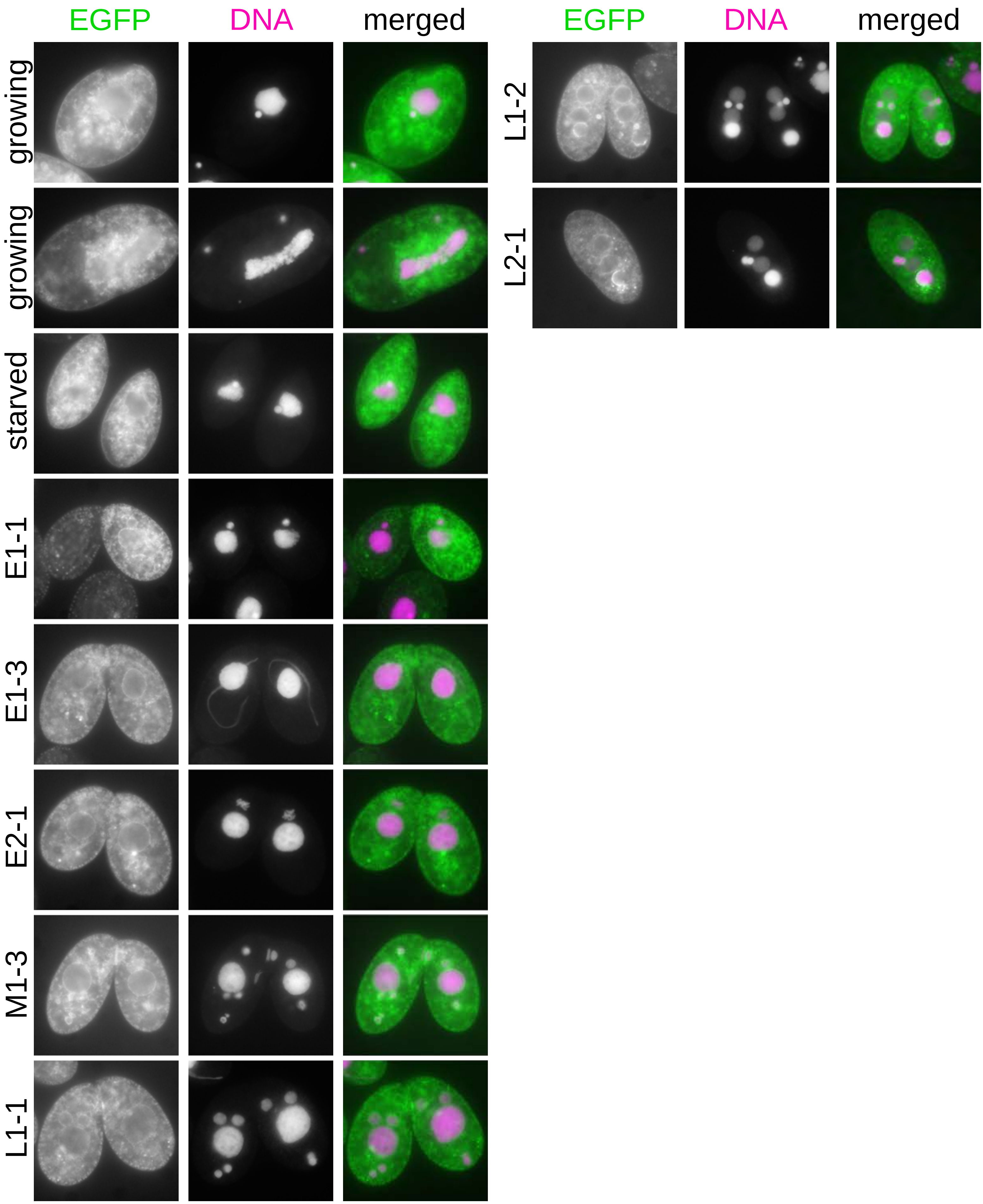

TTHERM_00113310
GenBank; XP_001010726.3

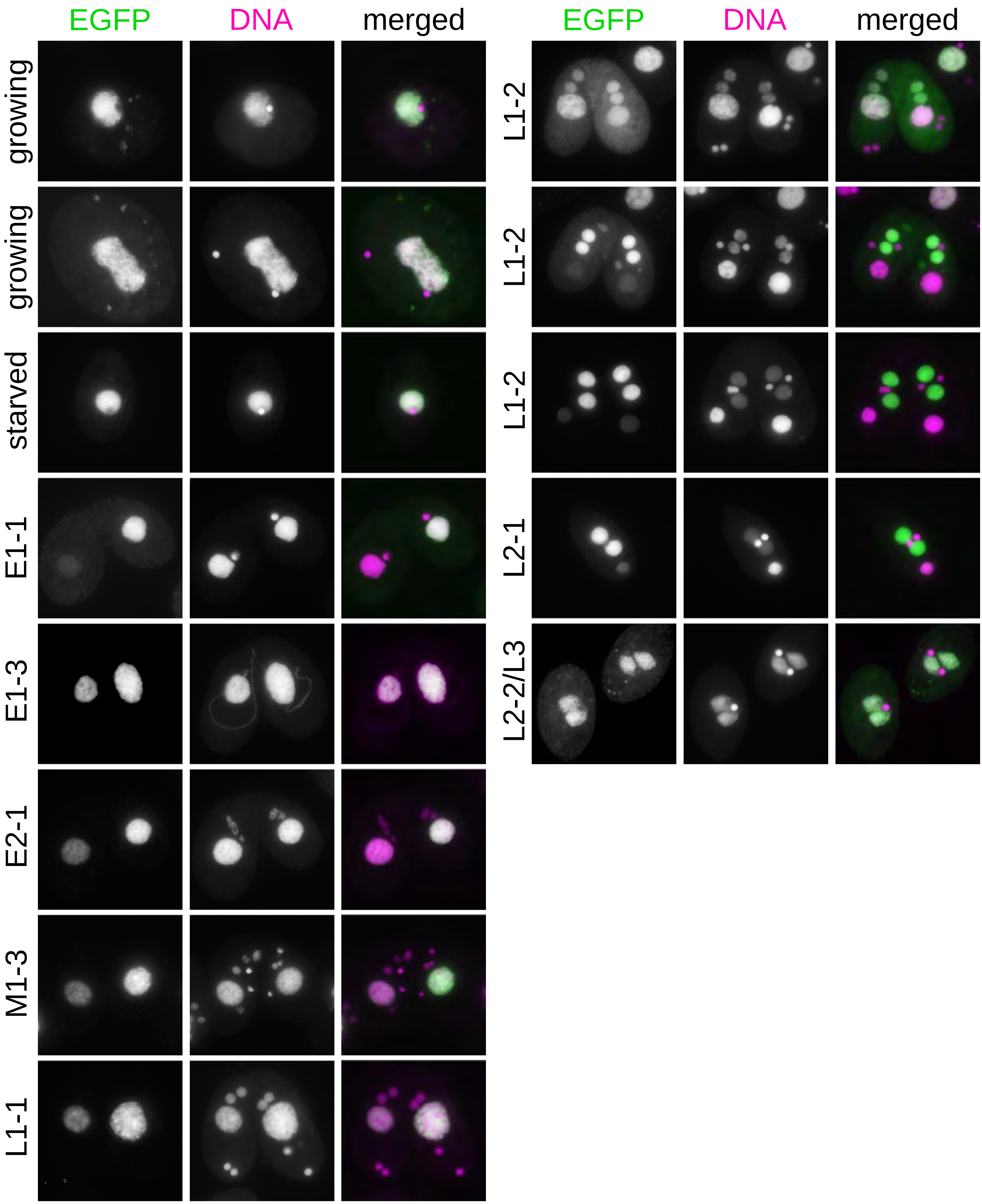

TTHERM_00185640
GenBank; XP_001008835.2
Gene name; *JMJ1*

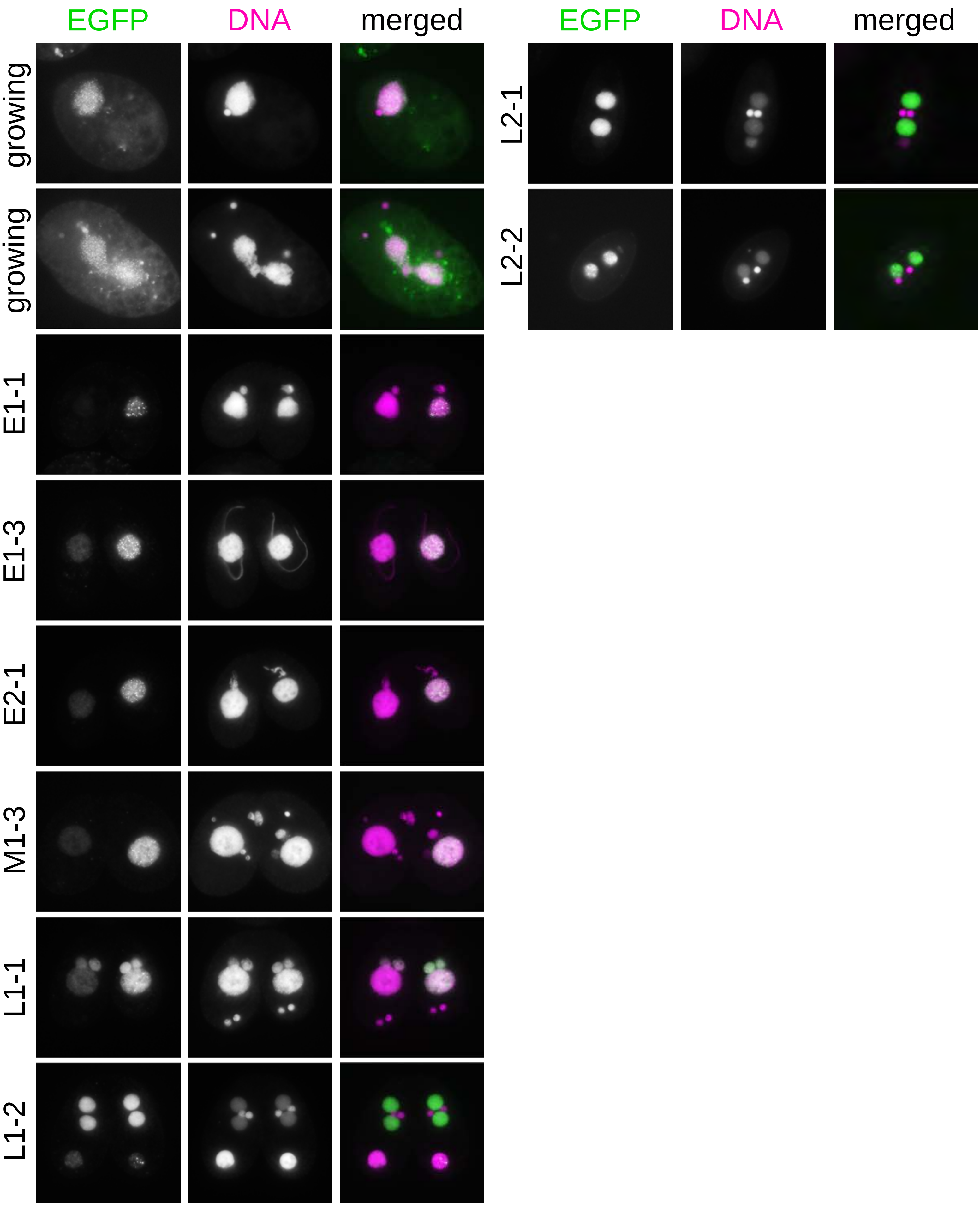

TTHERM_00204150
GenBank; XP_001007101.1

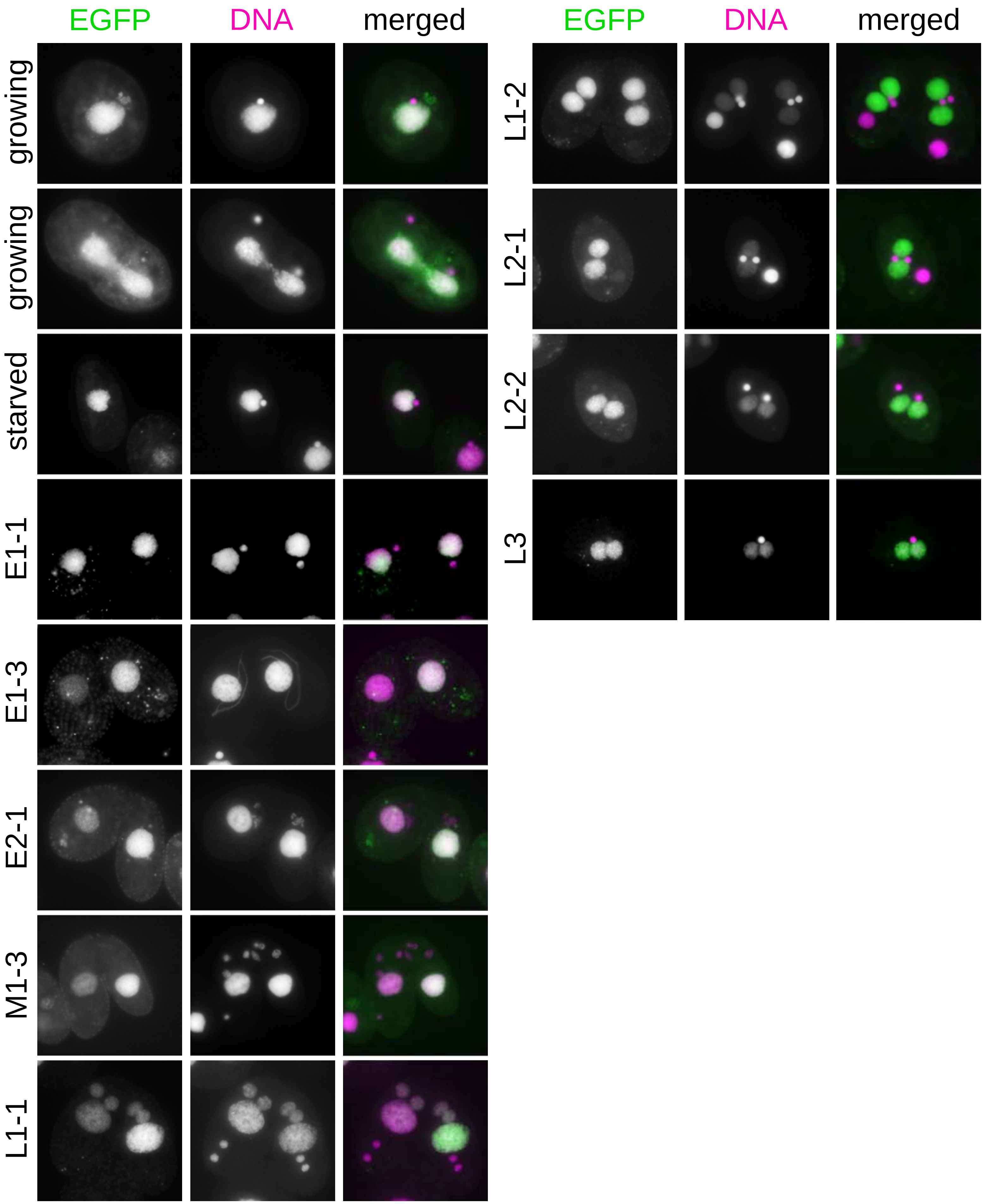

TTHERM_00221110
GenBank; XP_001020683.3

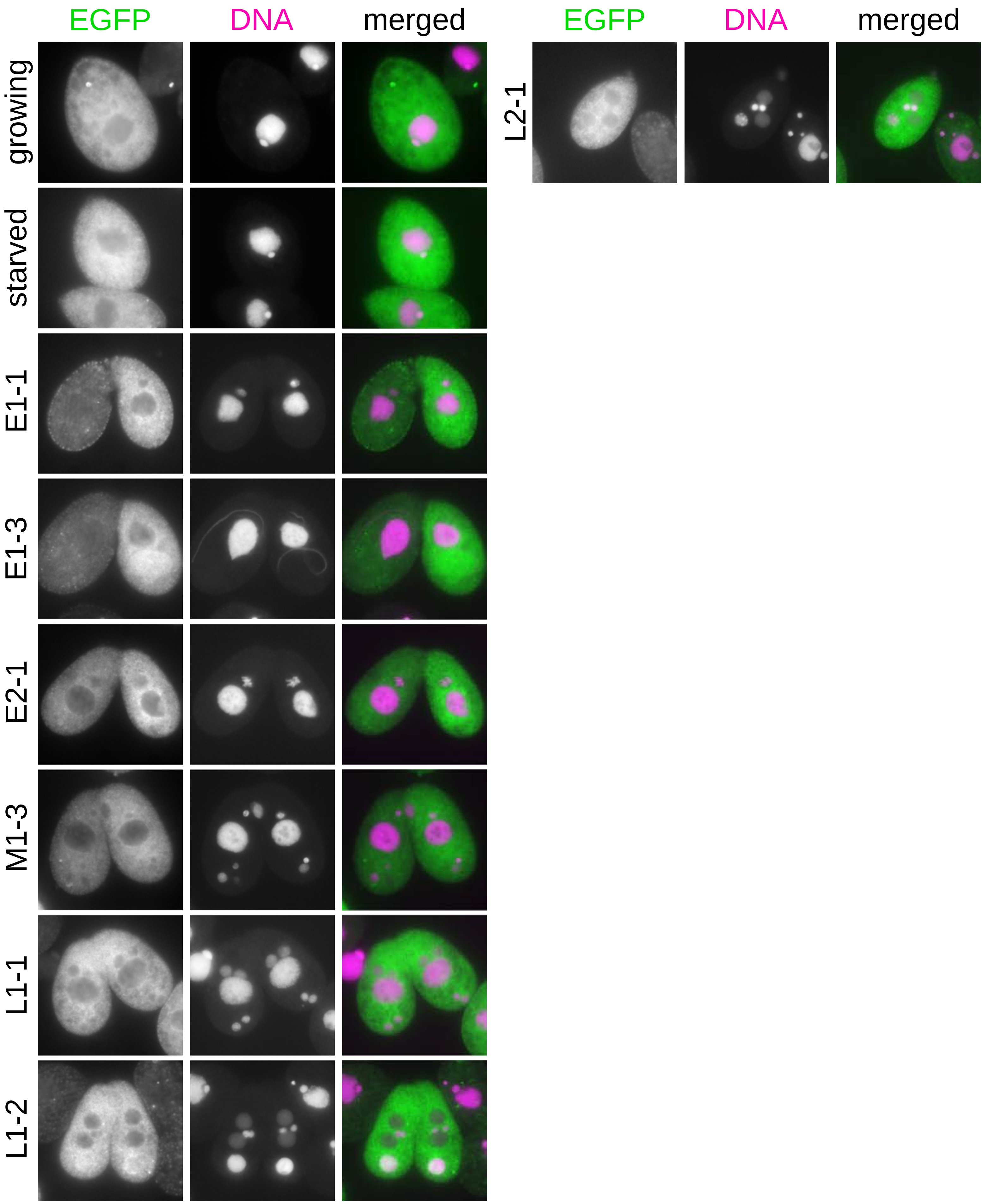

TTHERM_00267890
GenBank; XP_001015924.2

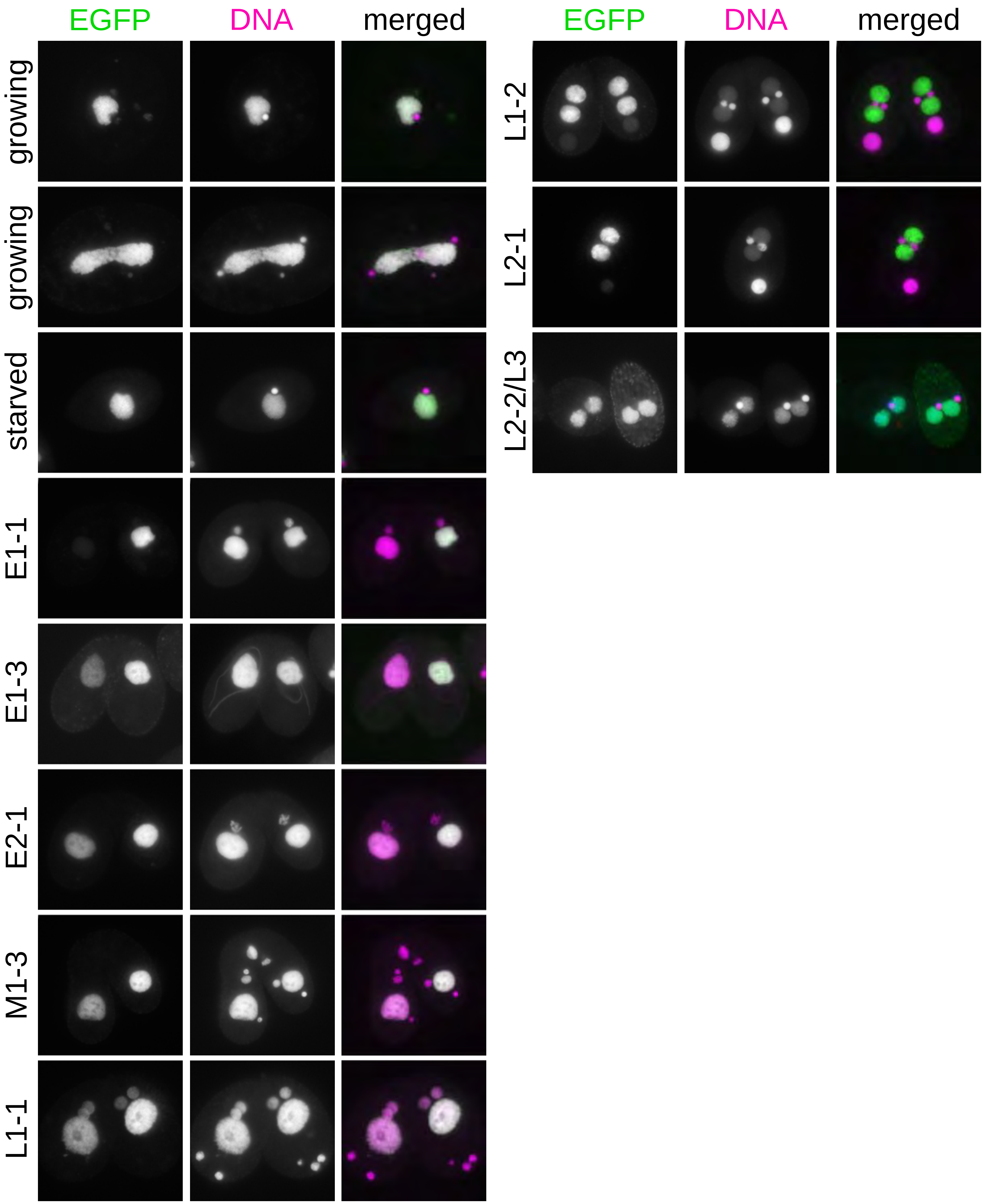

TTHERM_00301910
GenBank; XP_001024619.1

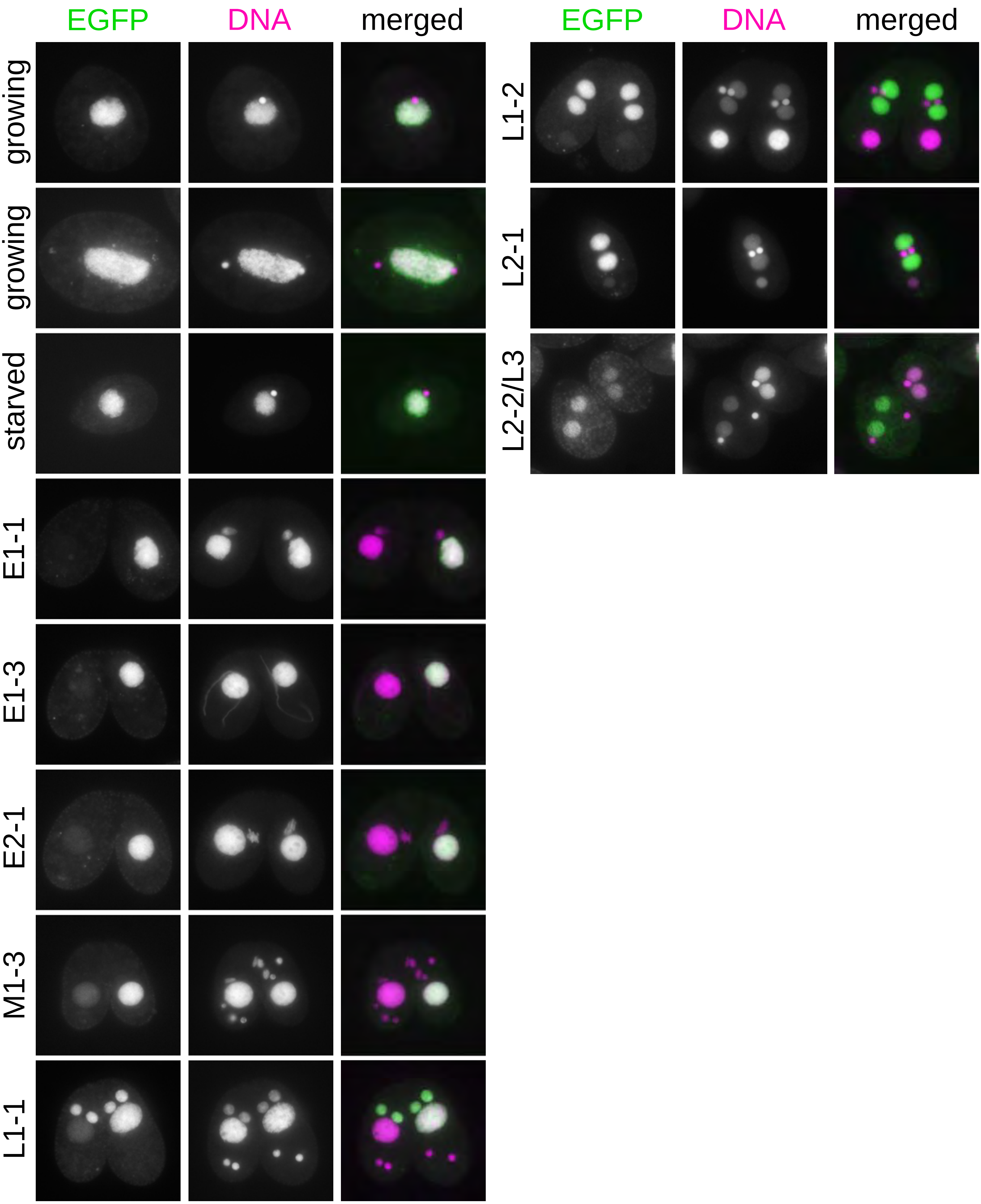

TTHERM_00439300
GenBank; XP_001017827.2

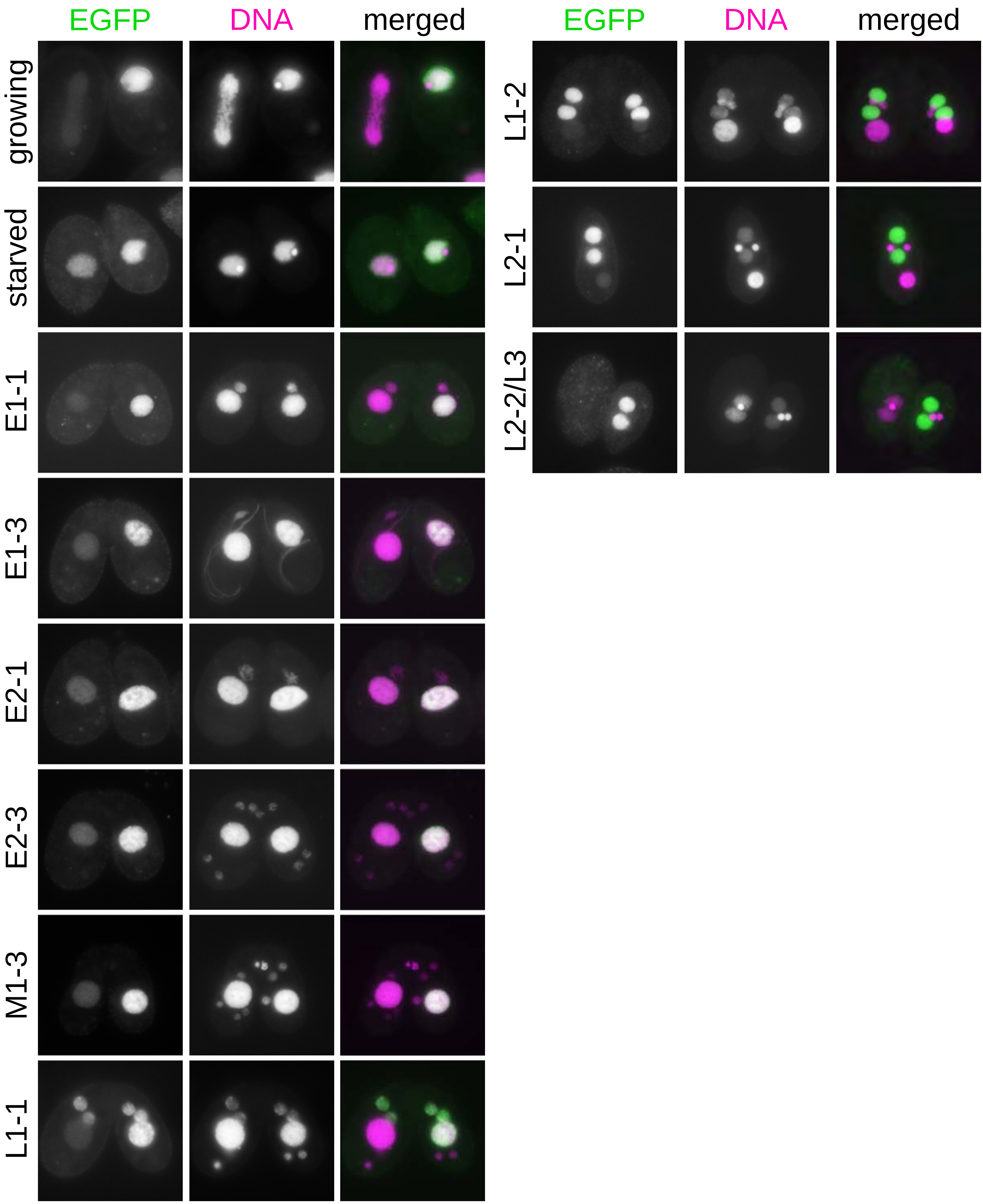

TTHERM_00460720
GenBank; XP_001018768.2

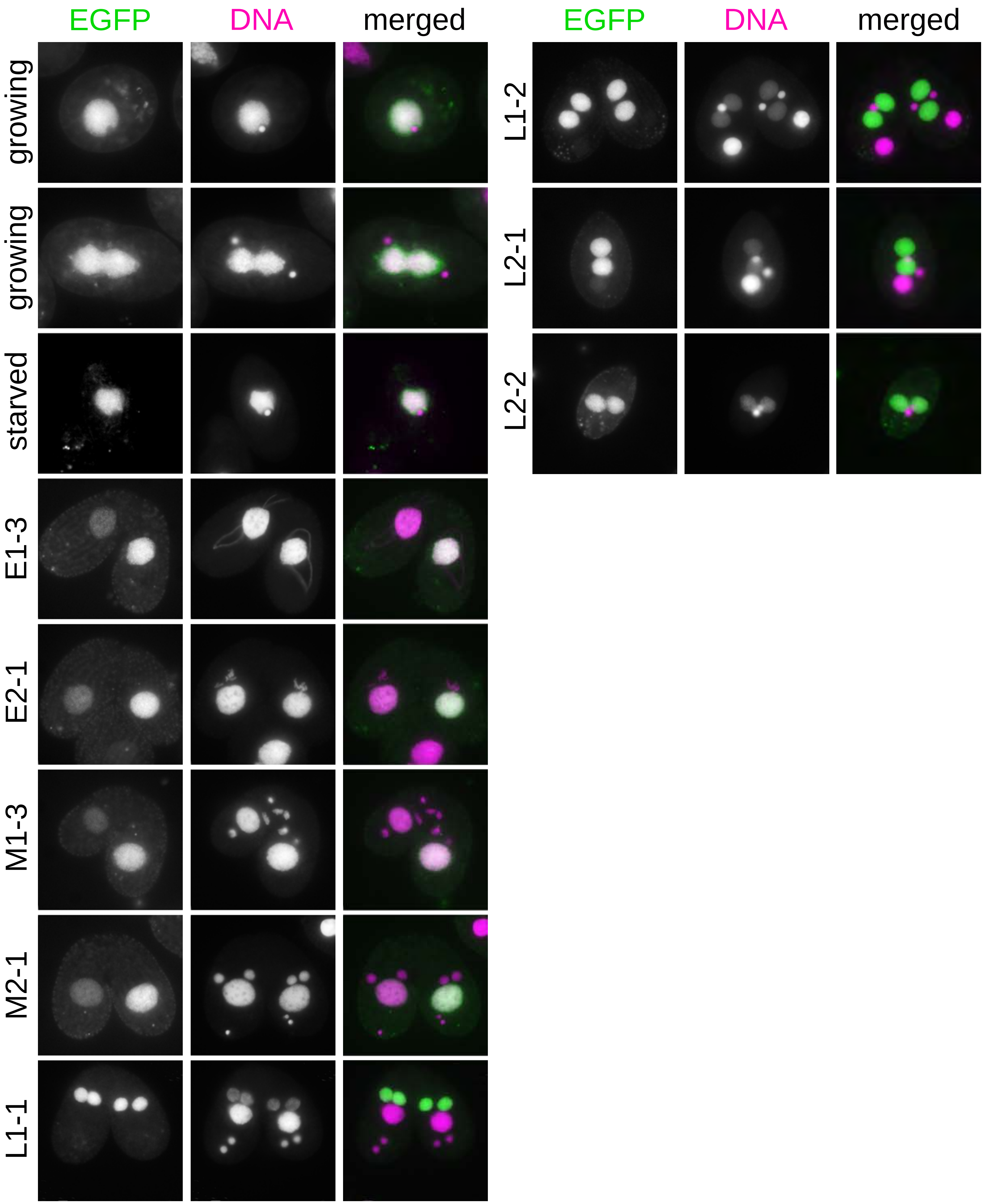

TTHERM_00471730
GenBank; XP_001033057.1

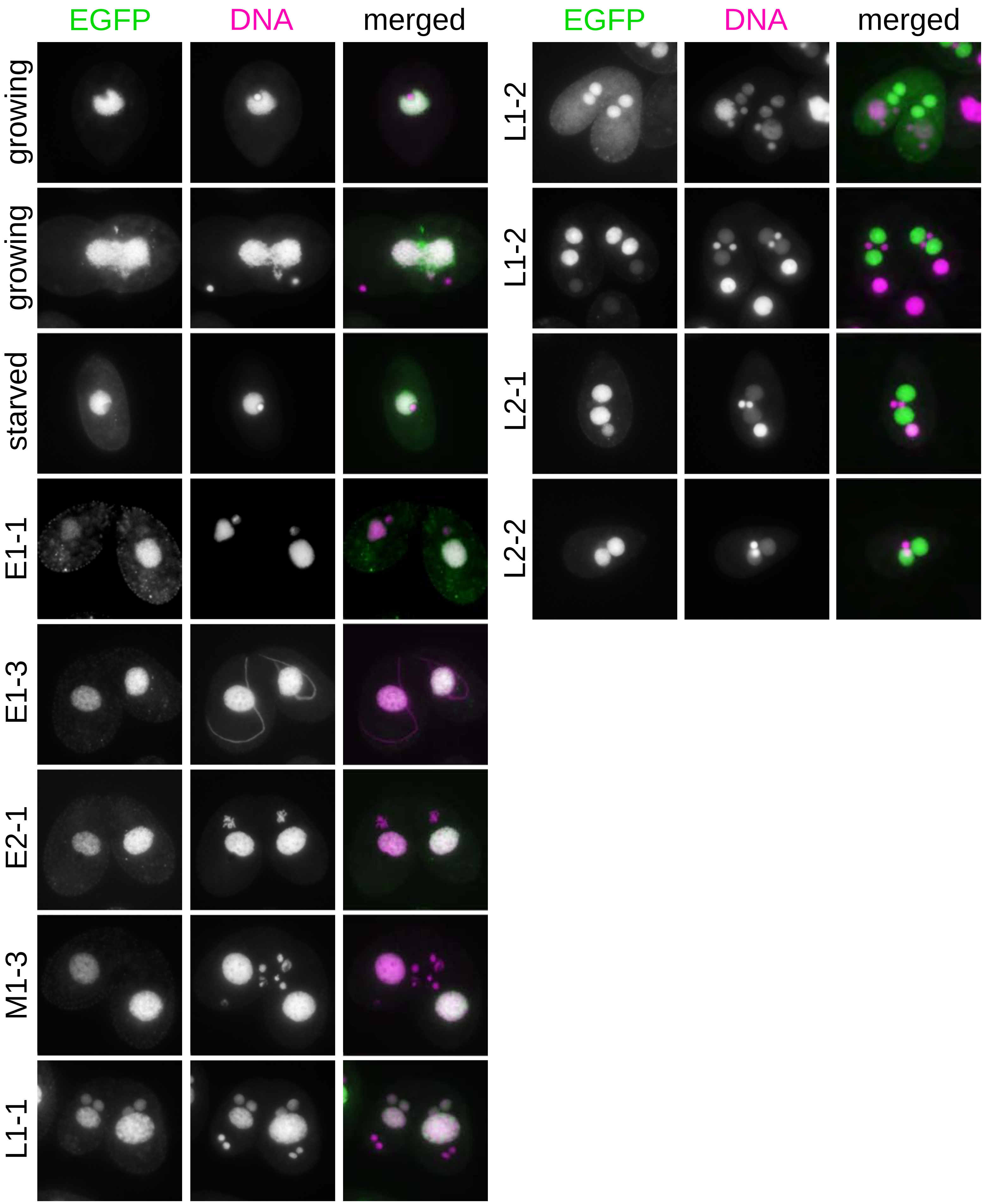

TTHERM_00474360
GenBank; XP_001023920.1

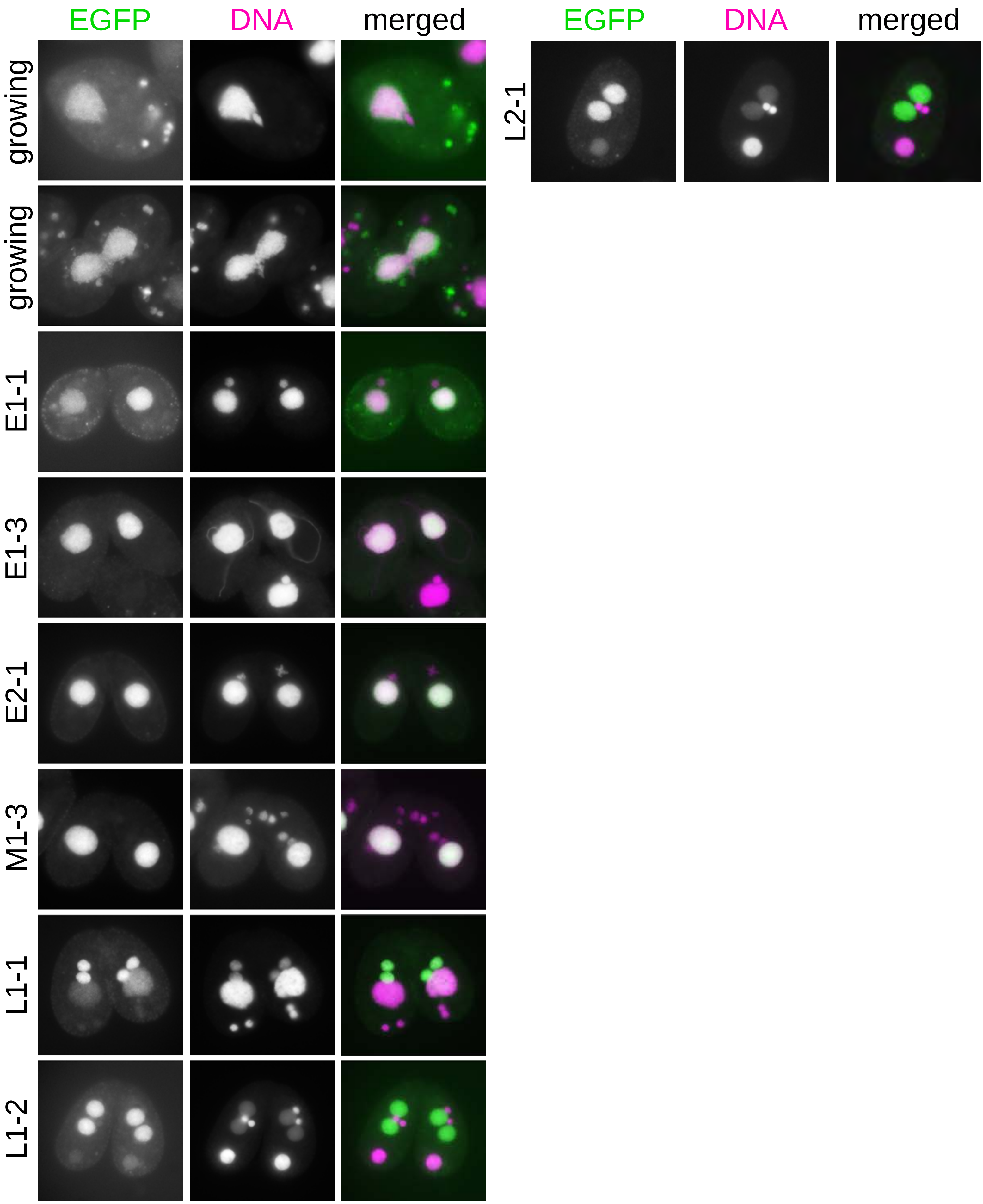

TTHERM_00487030
GenBank; XP_001032915.1

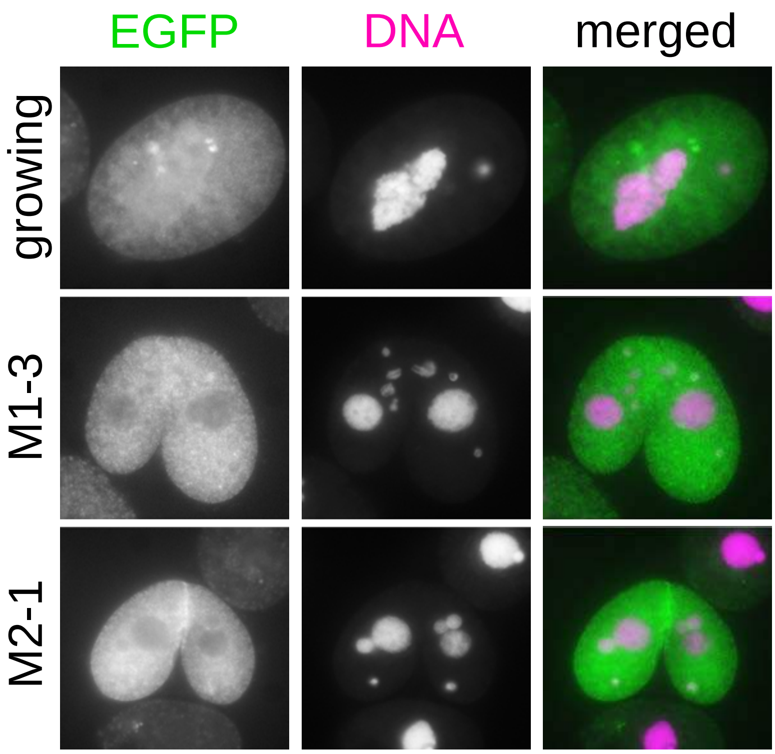

TTHERM_00540090
GenBank; XP_001015924.2

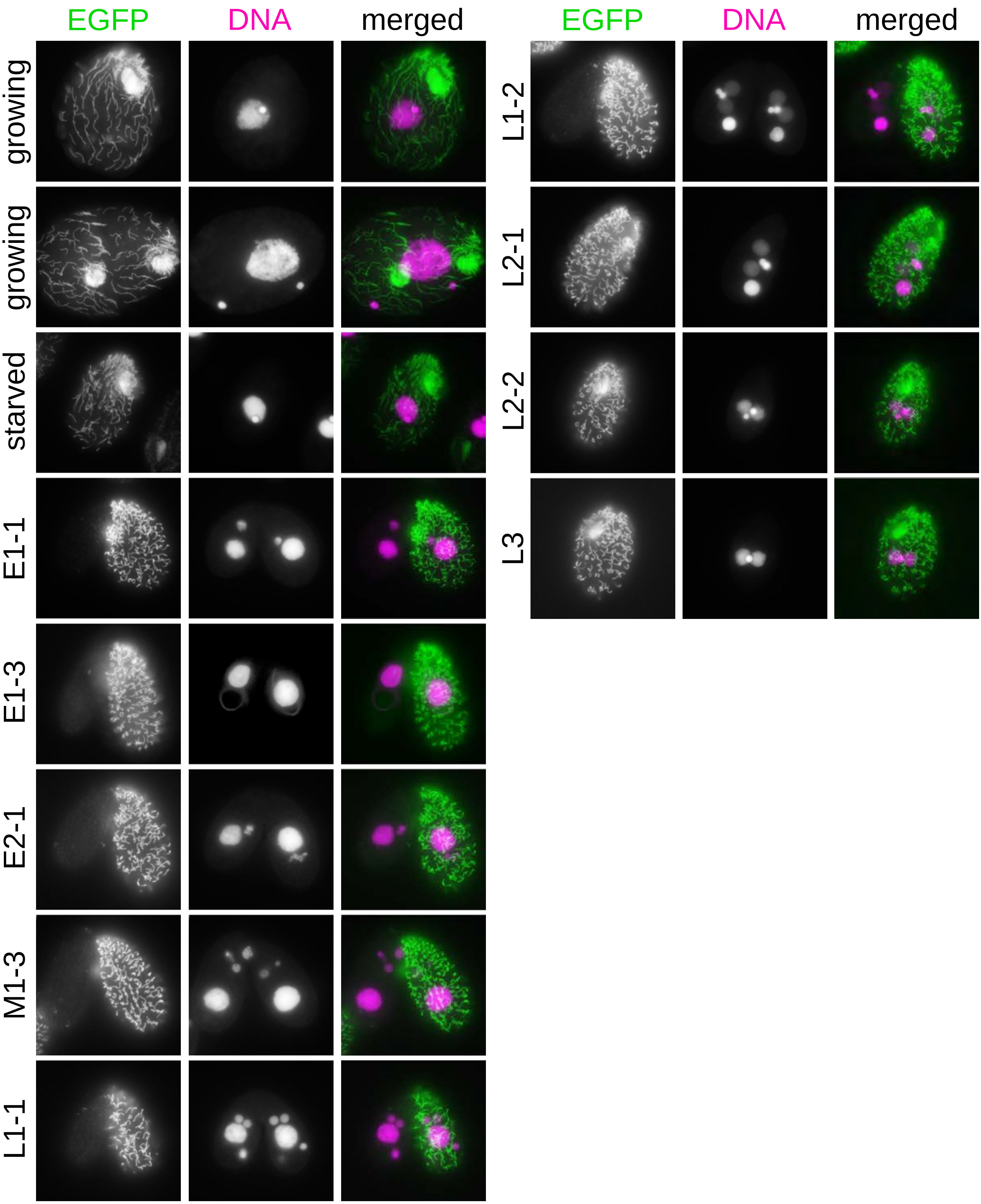

TTHERM_00704020*
GenBank; XP_001032072.1

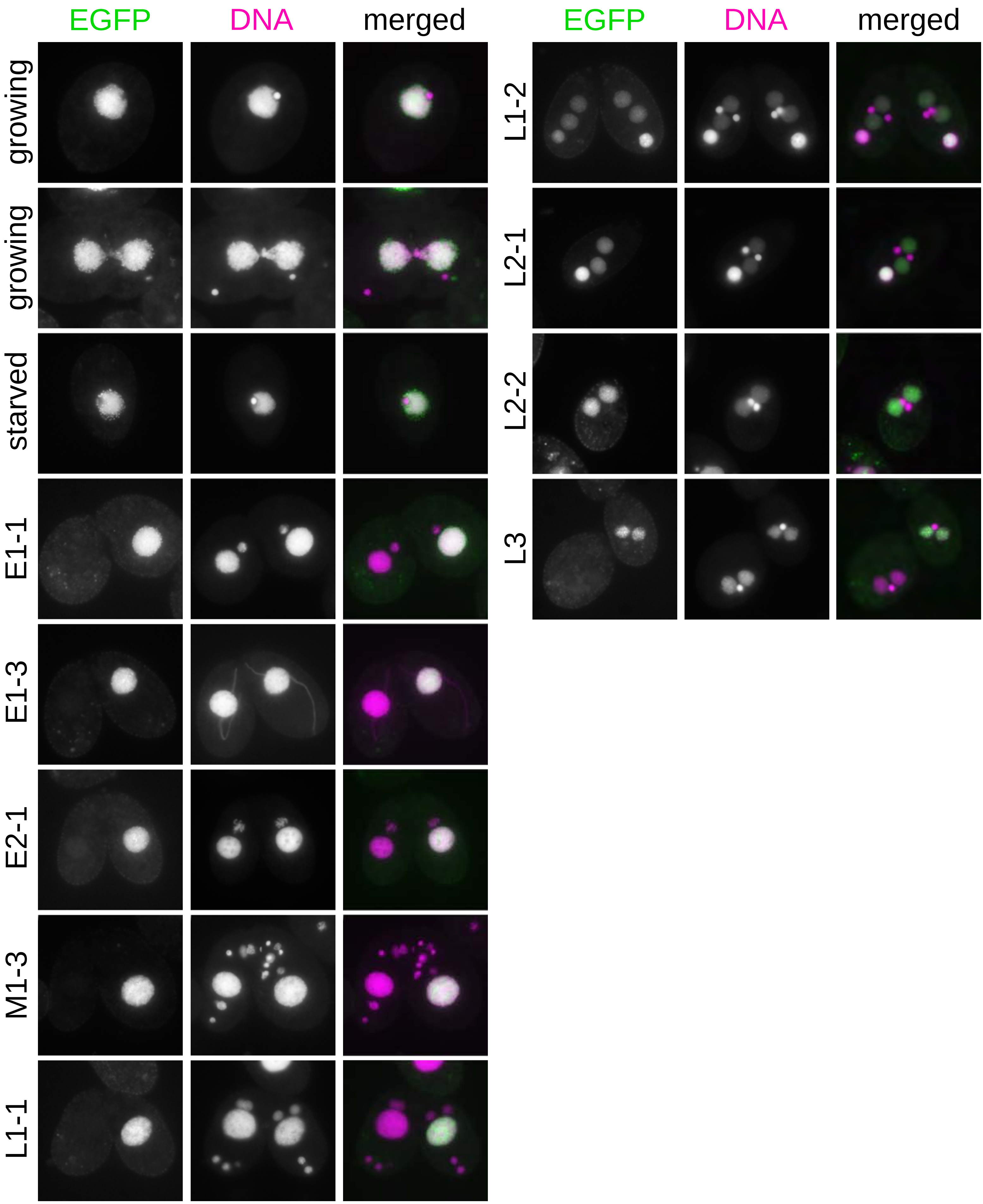

TTHERM_00706330
GenBank; XP_001010963.2

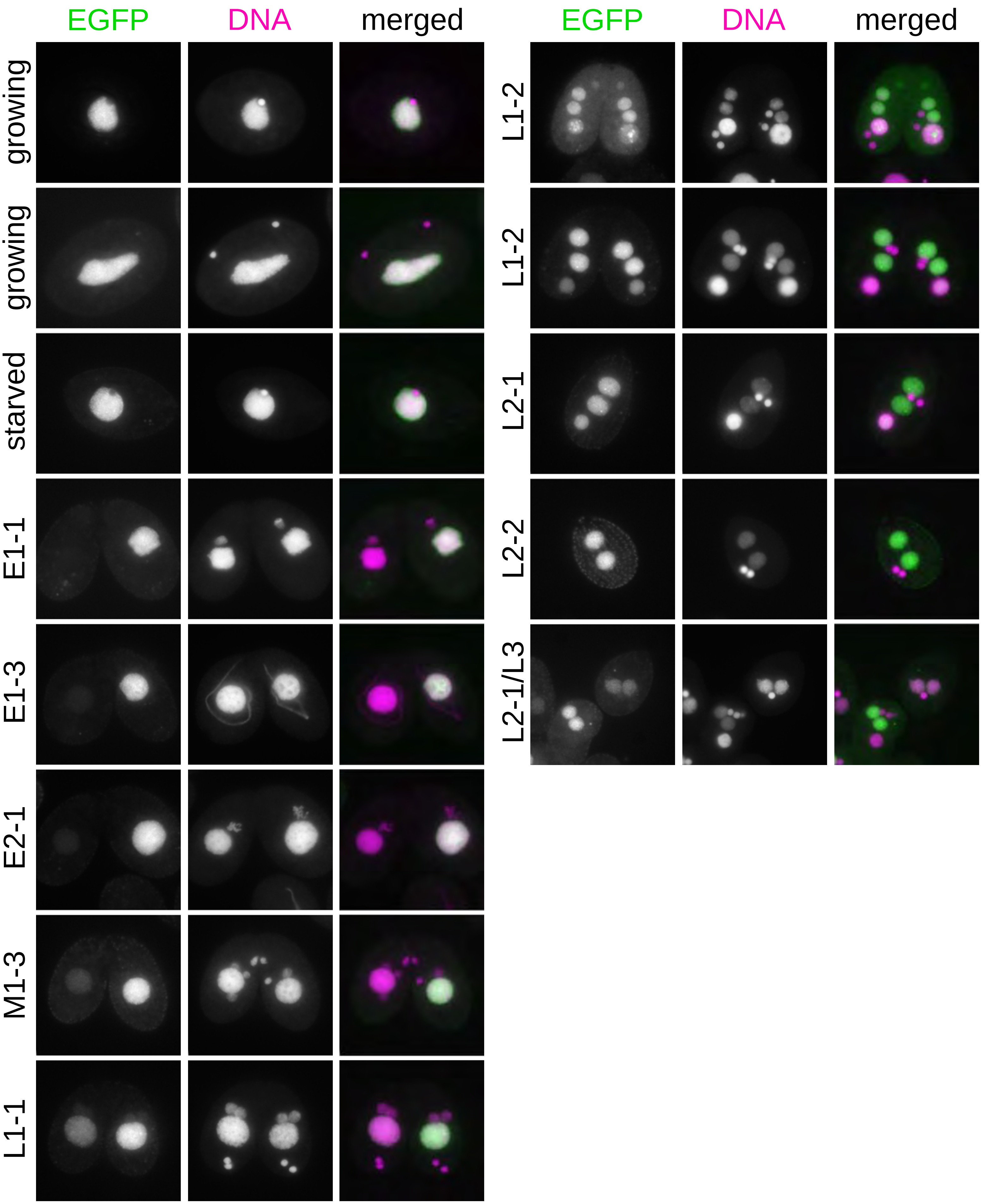

TTHERM_00849260
GenBank; XP_001019228.3
Gene name; *LIA6*

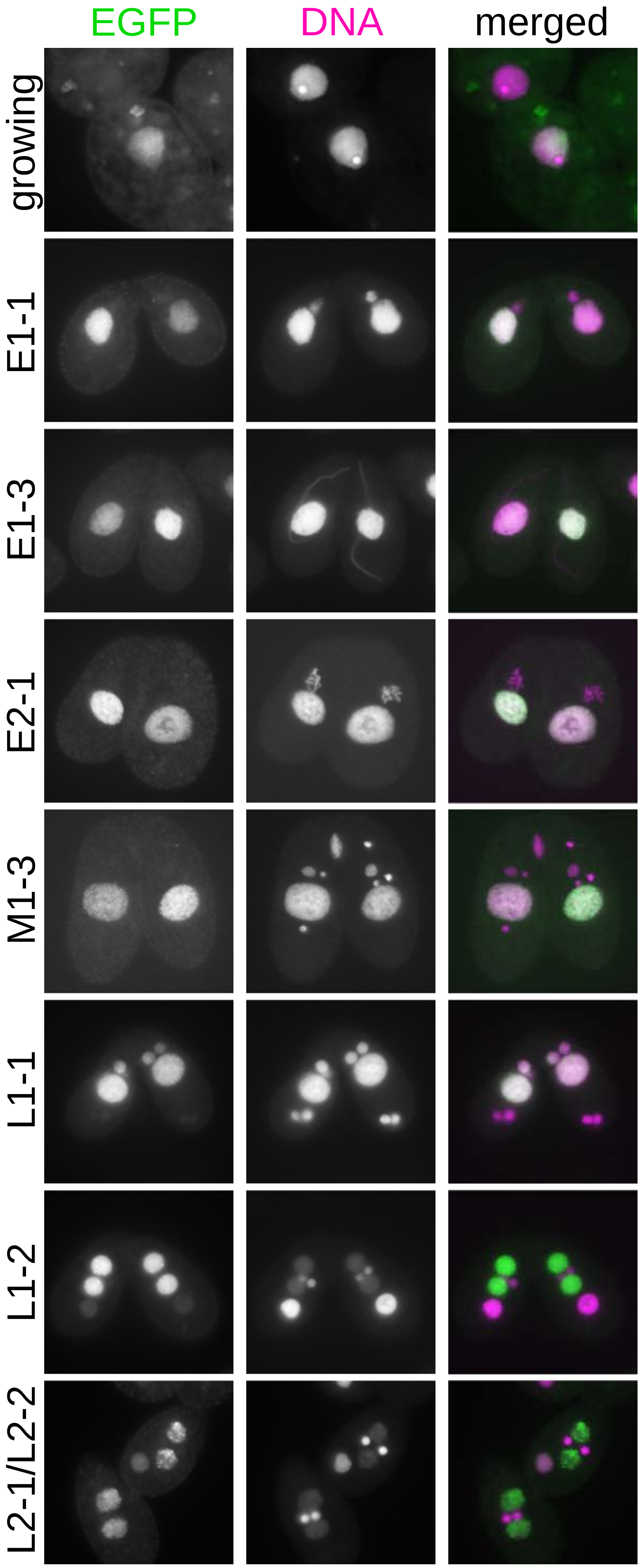

TTHERM_00974120
GenBank; XP_001009965.1

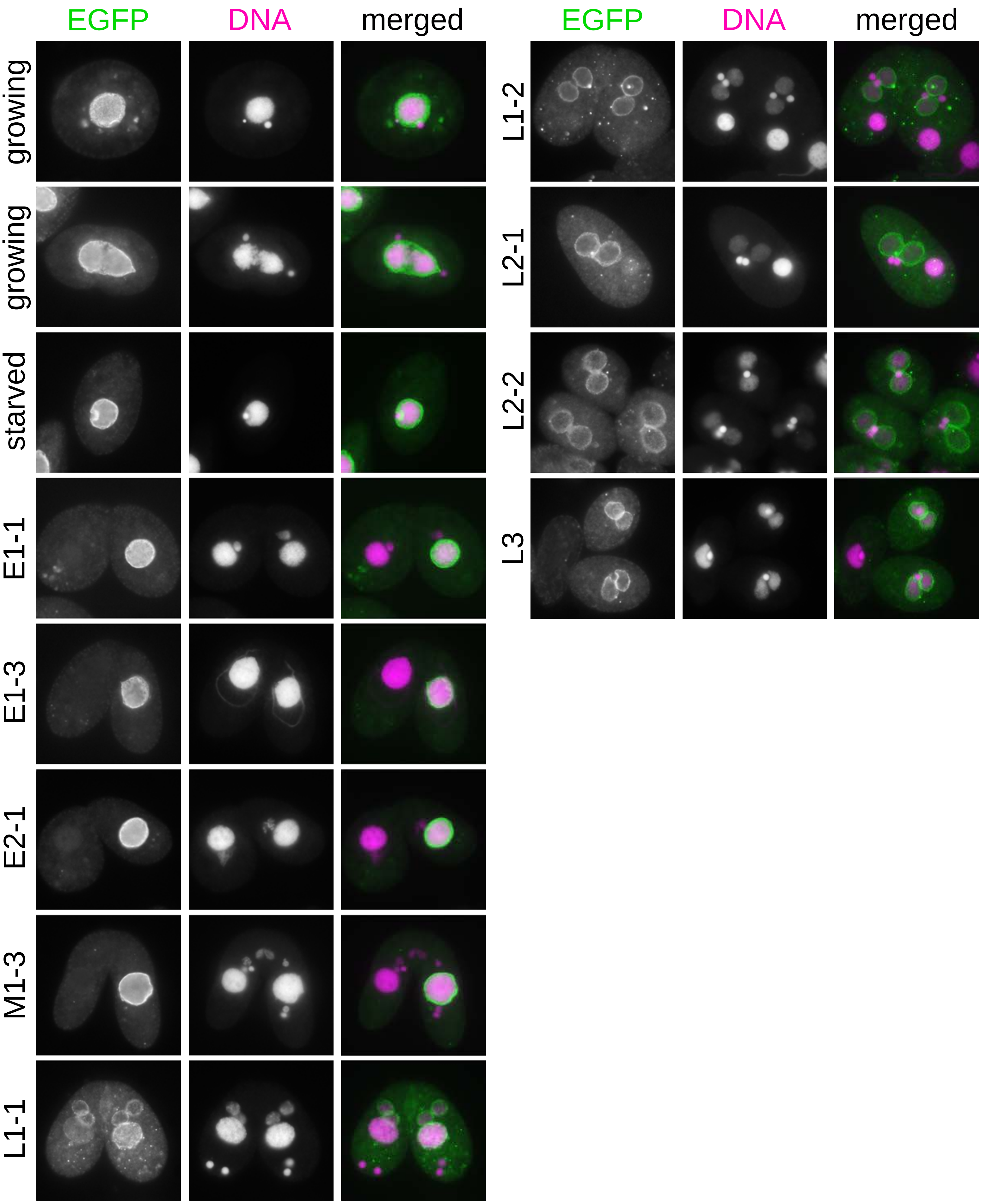

TTHERM_01014770
GenBank; XP_001030823.3

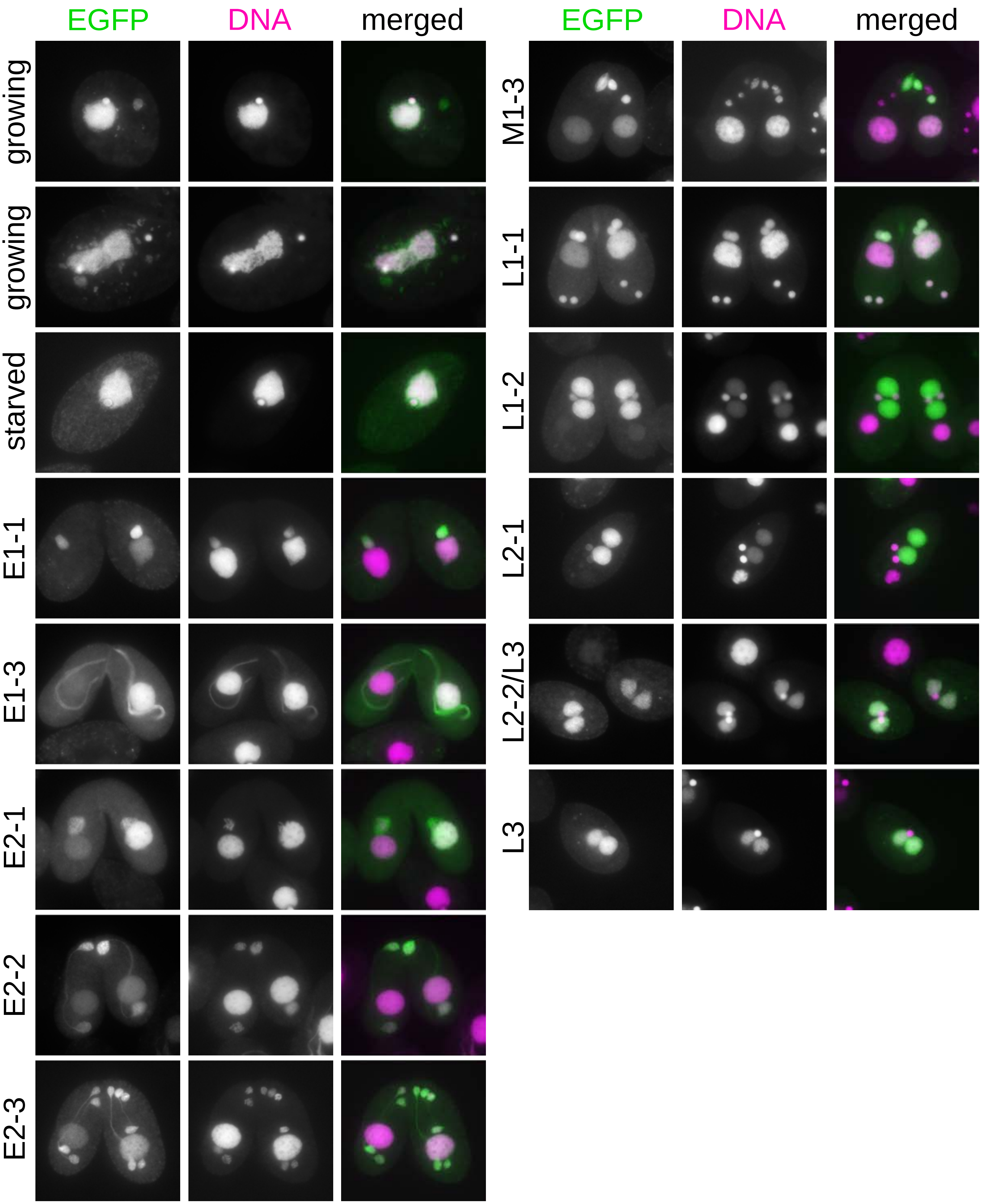

Supplement: Data S1. Summary of the Protein Localization Screen, Nuclear Events during the Life Cycle of Tetrahymena thermophila, and Localizations of EGFP-Tagged Proteins [file mmc2.pdf]
