# Supplementary material for: Phosphorylation of an HP1-like Protein Regulates Heterochromatin Body Assembly for DNA Elimination
Source: Dev Cell. 2015 Dec 21;35(6):775–88. doi: 10.1016/j.devcel.2015.11.017 (PMC4695338; doi:10.1016/j.devcel.2015.11.017)
Supplement: Document S2. Article plus Supplemental Information [file mmc3.pdf]

Article

Developmental Cell

Phosphorylation of an HP1-like Protein Regulates Heterochromatin Body Assembly for DNA Elimination

Graphical Abstract

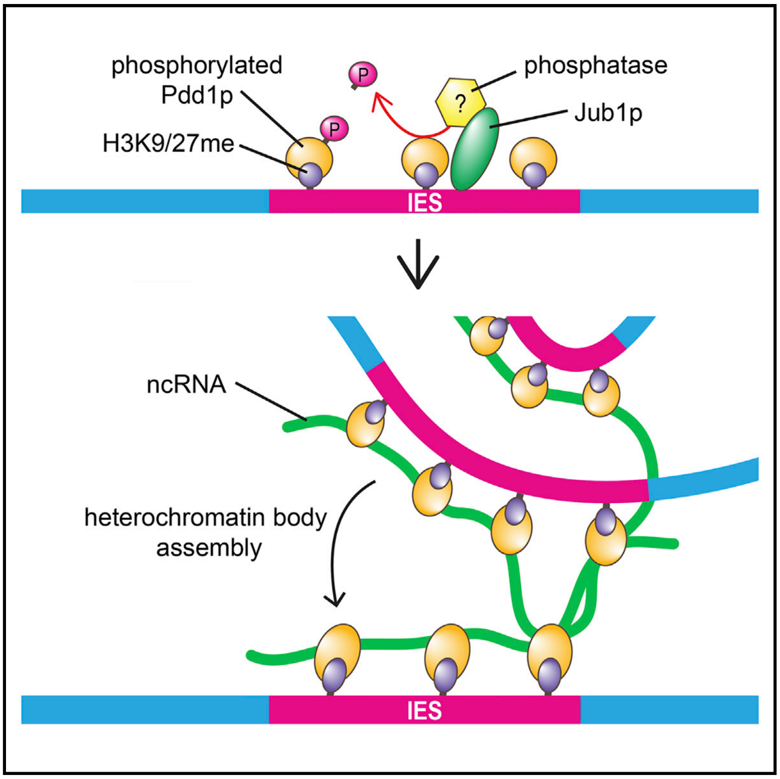

Authors

Kensuke Kataoka, Kazufumi Mochizuki

Correspondence

kazufumi.mochizuki@imba.oeaw.ac.at

In Brief

Heterochromatic loci are often assembled into higher-order heterochromatin bodies. Kataoka and Mochizuki provide evidence that developmentally programmed dephosphorylation of an HP1-like protein in *Tetrahymena* promotes its interaction with RNA, heterochromatin body assembly, and DNA elimination without altering local heterochromatic states, suggesting that heterochromatin body has an essential biological function.

Highlights

- Jub1p facilitates dephosphorylation of the HP1-like protein Pdd1p
- Pdd1p dephosphorylation promotes its RNA binding and heterochromatin body formation
- Jub1p and Pdd1p dephosphorylation is required for DNA elimination
- Heterochromatin body has function beyond local heterochromatin maintenance

Accession Numbers

GSE70083

CrossMark

Kataoka & Mochizuki, 2015, Developmental Cell *35*, 775–788
December 21, 2015 ©2015 The Authors
http://dx.doi.org/10.1016/j.devcel.2015.11.017

CellPress

Developmental Cell
Article

CellPress

Phosphorylation of an HP1-like Protein Regulates Heterochromatin Body Assembly for DNA Elimination

Kensuke Kataoka[1] and Kazufumi Mochizuki[1,*]
[1]Institute of Molecular Biotechnology of the Austrian Academy of Sciences (IMBA), Dr. Bohr-Gasse 3, 1030 Vienna, Austria
*Correspondence: kazufumi.mochizuki@imba.oeaw.ac.at
http://dx.doi.org/10.1016/j.devcel.2015.11.017
This is an open access article under the CC BY license (http://creativecommons.org/licenses/by/4.0/).

SUMMARY

Heterochromatic loci are often assembled into higher-order heterochromatin bodies in diverse eukaryotes. However, the formation and biological roles of heterochromatin bodies are poorly understood. In the ciliated protozoan *Tetrahymena*, de novo heterochromatin body formation is accompanied by programmed DNA elimination. Here, we show that the heterochromatin body component Jub1p promotes heterochromatin body formation and dephosphorylation of the Heterochromatin Protein 1-like protein Pdd1p. Through the mutagenesis of the phosphorylated residues of Pdd1p, we demonstrate that Pdd1p dephosphorylation promotes the electrostatic interaction between Pdd1p and RNA in vitro and heterochromatin body formation in vivo. We therefore propose that heterochromatin body is assembled by the Pdd1p-RNA interaction. Pdd1p dephosphorylation and Jub1p are required for heterochromatin body formation and DNA elimination but not for local heterochromatin assembly, indicating that heterochromatin body plays an essential role in DNA elimination.

INTRODUCTION

Heterochromatin is a closed and mostly transcriptionally repressed state of chromatin, which is dictated by a set of post-translational histone modifications (Jenuwein and Allis, 2001; Kouzarides, 2007). Hypoacetylation of histone tails maintains closed configuration of heterochromatin by exposing positively charged lysine (Lys) and stabilizing histone-DNA interactions (Mutskov et al., 1998). Methylated histone H3 at Lys 9 (H3K9me) recruits Heterochromatin Protein 1 (HP1), which self-oligomerizes to facilitate compaction of nucleosome arrays (Canzio et al., 2011; Cowieson et al., 2000). Similarly, methylated histone H3 at Lys 27 (H3K27me) attracts Polycomb Repressive Complex 1 (PRC1), and PRC1-DNA and PRC1-PRC1 interactions compact chromatin (Eskeland et al., 2010; Grau et al., 2011).

In certain cell types, multiple heterochromatic loci are assembled into aggregated higher order structures called heterochromatin bodies (Politz et al., 2013). In mammals to plants, constitutive heterochromatin loci at centromeric and other repetitive sequences are organized into heterochromatin bodies called chromocenters (Fransz and de Jong, 2002; Probst and Almouzni, 2011). Chromocenters are condensed their underlying sequences that are tightly silenced in differentiated mammalian cells, whereas they are more dispersed and transcribed in embryonic stem cells and in some cancer cells (Carone and Lawrence, 2013; Efroni et al., 2008; Zhu et al., 2011). In female mammalian somatic cells, a whole X chromosome forms a heterochromatin body, called the Barr body, which is suggested to be important for X inactivation (Deng et al., 2014). Moreover, in human senescent cells, heterochromatic loci are reorganized into senescence-associated heterochromatin foci (SAHF), which are proposed to be a part of the tumor suppressor pathway (Narita, 2007). Because SAHF are formed without detectable alterations of underlying histone modifications (Chandra et al., 2012), SAHF formation per se is likely involved in gene regulation. These examples indicate that, in addition to the compaction of individual heterochromatic loci, their assembly into heterochromatin bodies may play important roles in regulating chromatin activities. However, because there is no intervention system in which heterochromatin body formation is disturbed without altering local heterochromatin, whether and to what extent heterochromatin body contributes to the regulation of the underlying sequences remain unknown.

Heterochromatin and heterochromatin bodies are formed during the process of programmed DNA elimination in *Tetrahymena thermophila* (Chalker, 2008). Like most ciliated protozoans, *Tetrahymena* harbors two types of nuclei in a single cell: the transcriptionally inactive germline micronucleus (MIC) and the transcriptionally active somatic macronucleus (MAC). Nutritional starvation induces sexual reproduction, called conjugation (Figure 1A), in which the MIC undergoes meiosis and its zygotic products produce both new MIC and new MAC for progeny, whereas the parental MAC is degraded. In the new MAC, more than 8,000 internal eliminated sequences (IESs), which consist of one-third (~50 Mb) of the MIC genome, many of which are related to transposons, are removed by programmed DNA elimination (Chalker and Yao, 2011; Coyne et al., 2012; Kataoka and Mochizuki, 2011). An RNAi-related mechanism recruits the H3K9 and H3K27 dual-specific methyltransferase Ezl1p to IESs, resulting in the accumulation of H3K9/K27me and their binding HP1-like protein Pdd1p (Aronica et al., 2008; Liu et al., 2007; Taverna et al., 2002). During or prior to DNA elimination, thousands of heterochromatinized IES loci are organized into several electron-dense heterochromatin bodies (Madireddi et al., 1996) (see also Figure 1C). The heterochromatinized IESs are eventually

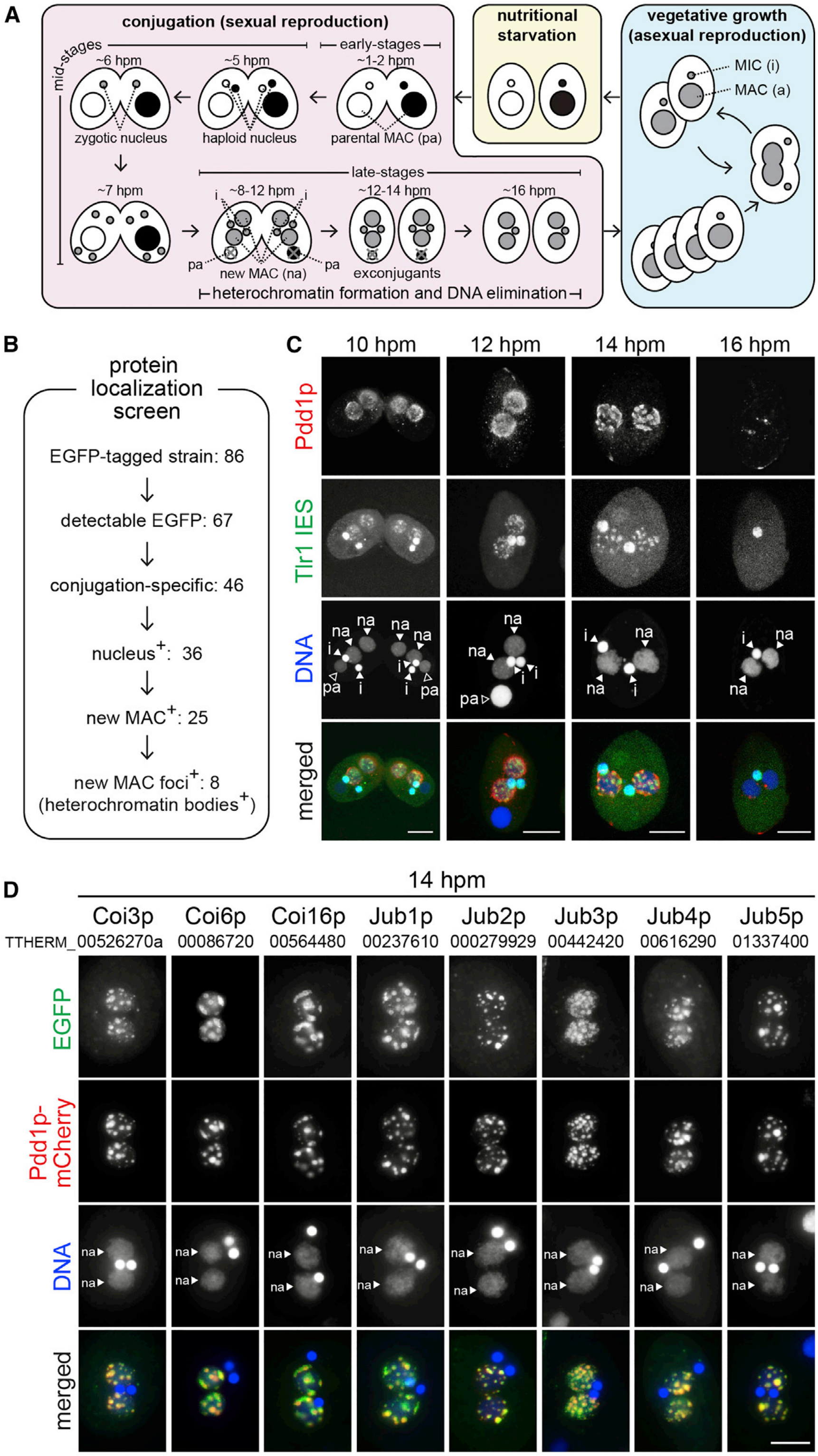

Figure 1. Identification of Heterochromatin Body Components

(A) A single *Tetrahymena thermophila* cell possesses a MAC (a) and a MIC (i). During vegetative growth, these nuclei divide and are segregated independently into daughter cells. Nutritional starvation induces the conjugation of two cells carrying different mating types. In the early conjugation stage (~1–4 hpm), the MICs undergo meiosis. In the mid-stage, one of the meiotic products is exchanged between the cells (~5 hpm) and fuses with the stationary meiotic product to form a zygotic nucleus (~6 hpm), which then divides twice to form two new MACs and two MICs (~7 hpm). At the late-stage, the new MACs (na) are enlarged (~8 hpm). The pair is dissolved and the parental MAC (pa) and one of the MICs are degraded in the exconjugants (~12–16 hpm). The exconjugants resume vegetative growth when nutrients are available.

(B) Summary of the screen for heterochromatin body components.

(C) WT cells at 10, 12, 14, and 16 hpm were hybridized with a probe complementary to the Tlr1 element (green) and stained with an anti-Pdd1p antibody (red). DNA was stained with DAPI (blue). Arrowheads indicate the MIC (i), the new MAC (na), and the parental MAC (pa). The scale bars represent 10 μm.

(D) Exconjugants expressing the indicated proteins tagged with EGFP (green) and Pdd1p-mCherry (red) were counterstained with DAPI (blue). Arrowheads indicate the new MACs (na). All pictures share the scale bar, representing 10 μm.

See also Figure S1.

excised by the domesticated transposase Tpb2p (Cheng et al., 2010; Vogt and Mochizuki, 2013).

Because DNA elimination in *Tetrahymena* occurs during the course of inducible conjugation, it serves as an ideal model to analyze the process of heterochromatin body formation. Here, we show identification and functional analyses of the heterochromatin body component Jub1p, which suggest that the heterochromatin body is assembled by phosphorylation-mediated electrostatic regulation of RNA-Pdd1p interaction and is essential for DNA elimination.

RESULTS

Identification of Proteins that Localize to Nuclear Foci in the New MAC

To identify proteins involved in the assembly of heterochromatin bodies in *Tetrahymena*, we performed a protein localization screen to identify heterochromatin body components. Because most of the reported heterochromatin body components are exclusively expressed during conjugation (Chalker, 2008), we analyzed 86 genes with expressed sequence tags only in conjugating cells. Each gene was engineered to express a fusion protein with EGFP from its endogenous MAC locus (Figure S1A, top), and its localization was observed in exponentially growing, starved, and conjugating cells.

The screen is summarized in Figure 1B, and detailed data can be found in Data S1. For 67 genes, EGFP-tagged proteins were detected at least in one of the stages analyzed, and 46 of them showed conjugation-specific expression. Among them, 36 were detected in at least one nucleus, and 25 of these localized to the new MAC. Importantly, 8 of the new MAC-localizing proteins were detected in nuclear foci at the late (14 hr post-mixing [hpm]) stage.

Nuclear Foci Proteins Identified Are Heterochromatin Body Components

To localize heterochromatin body, we first validated the HP1-like protein Pdd1p as a marker. Pdd1p, which was immunofluorescently stained by an anti-Pdd1p antibody, and the moderately repeated IES element Tlr1 (Wuitschick et al., 2002), which was detected by DNA fluorescence in situ hybridization (DNA-FISH), were first distributed homogeneously in the new MAC at the onset of new MAC differentiation (Figure 1C, 10 hpm). They then gradually accumulated into several foci at later stages (Figure 1C, 12–14 hpm) and eventually disappeared (Figure 1C, 16 hpm). Because Pdd1p and the Tlr1 IESs co-localized in the same foci (Figure 1C, 14 hpm), Pdd1p can be used as a marker for IES-containing heterochromatin bodies. Counterintuitively, the heterochromatin bodies were poorly stained with DAPI (Figure 1C, 14–16 hpm). However, because the heterochromatin bodies were stained intensely with an anti-DNA antibody (Figure S1B), we believe that DNA is concentrated in the heterochromatin bodies but DNA there has a low affinity for DAPI for an unknown reason.

We then compared the localization of mCherry-tagged Pdd1p to those of the eight foci-forming proteins tagged with EGFP (Figure S1A). All of these foci-forming proteins co-localized with Pdd1p-mCherry at 14 hpm (Figure 1D), indicating that they are heterochromatin body components. Three of them, Coi3p, Coi6p, and Coi16p, were partially characterized previously (Woehrer et al., 2015). The other five proteins were named Junk Buster 1–5 (Jub1p–5p) (Figure 1D). Jub5p was reported during the course of this study and also called Tcd1p (Xu et al., 2015). Coi6p and Jub5p are HP1-like proteins. Jub3p is a WD40 repeat protein similar to a component of PRC2 in metazoans. The other proteins show no detectable similarities with any known proteins outside of the genus *Tetrahymena*. In this study, we report the further characterization of Jub1p.

Jub1p Is a Heterochromatin Body Component

We raised an antibody against Jub1p. By western blot, the antibody detected a protein appearing only during the late conjugation stages in wild-type cells (Figure 2A). This expression pattern is consistent with that of *JUB1* mRNA (Figure S2A). Moreover, the protein was not detected in *JUB1* knockout (KO) cells (see below for construction of *JUB1* KO cells) by both western blot (Figure 2B) and immunofluorescent staining (Figure 2C, Δ*JUB1*). We therefore conclude that the antibody specifically recognizes Jub1p.

Next, the localizations of Jub1p and Pdd1p were compared by immunofluorescent staining using the anti-Jub1p and an anti-Pdd1p antibody in wild-type (WT) cells (Figure 2C, WT). Jub1p and Pdd1p localized to the new MACs (“na” in Figure 2C) right after MAC enlargement (8 hpm). They were first distributed uniformly in the new MAC (8–10 hpm) but then gradually accumulated into foci at later stages (12–14 hpm). Subsequently, the Jub1p foci became smaller (16 hpm) and disappeared when the Pdd1p-positive heterochromatin body was eliminated (18 hpm).

Jub1p Is Required for Heterochromatin Body Assembly

We established gene KO strains for *JUB1*, in which the entire *JUB1* protein-coding sequence in both the MAC and the MIC were replaced with a drug resistance gene, which was confirmed by genomic PCR (Figure 2D) and by northern blot (Figure S2B).

We analyzed heterochromatin body formation and the turnover process by categorizing exconjugants (progeny dissolved pairing) into three stages on the basis of the localization of Pdd1p (Figure 2E, top): in stage 1, Pdd1p is localized either homogeneously or in small puncta with continuous localization throughout the new MAC; in stage 2, Pdd1p is localized in discrete foci (heterochromatin bodies) with no detectable Pdd1p in the space between them; in stage 3, Pdd1p disappears completely. Approximately half of the exconjugants from WT cells were either in stage 1 or stage 2 at 12 hpm, and more than 80% of the exconjugants were in stage 3 at 15 hpm (Figure 2E, WT). By contrast, all exconjugants from the *JUB1* KO cells were in stage 1 even at 21 hpm (Figure 2E, Δ*JUB1*). Therefore, we conclude that Jub1p is required for heterochromatin body formation.

Jub1p Is Dispensable for the Establishment of Heterochromatin on IESs

Heterochromatin body formation can be disrupted by inhibiting either the establishment of heterochromatin on individual IESs or the aggregation of the multiple heterochromatinized IES loci. To determine which of these processes is disrupted in the absence of Jub1p, heterochromatin assembly on IESs was analyzed.

We first analyzed two heterochromatin-associated histone modifications, H3K9me3 and H3K27me3, by immunofluorescent staining using an anti-H3K9me3 and an anti-H3K27me3 antibody, respectively. These modifications were accumulated similarly in the new MACs of WT and *JUB1* KO cells at 8 hpm (Figures 3A and S3A). At 14 hpm, these modifications were localized in the Pdd1p-positive heterochromatin bodies in WT cells, but remained homogeneously distributed in the new MACs in *JUB1* KO cells (Figures 3B and S3B). These results indicate that in the new MAC of *JUB1* KO cells, heterochromatin is formed but not assembled into heterochromatin bodies.

We further analyzed the heterochromatin formation by chromatin immunoprecipitation (ChIP) followed by high-throughput DNA sequencing (ChIP-seq). We purified new MACs by fluorescence-activated cell sorting (FACS) from cells at 12 hpm, when most IESs remain in the new MAC chromosomes in WT cells (Austerberry et al., 1984). As previously shown for a few IESs by ChIP-PCR (Chung and Yao, 2012; Liu et al., 2007; Taverna et al., 2002), our ChIP-seq analysis using an anti-Pdd1p antibody showed that in WT cells, Pdd1p was accumulated on most of the IESs in a representative 100 kb MIC genome locus (Figure 3C, left) as well as on a modeled IES in which all predicted 1–5 kb IES loci (5,606 loci total) were compiled (Figure 3C, right). In *JUB1* KO cells, Pdd1p was enriched normally on IESs (Figure 3D). Altogether, we conclude that Jub1p is not required for

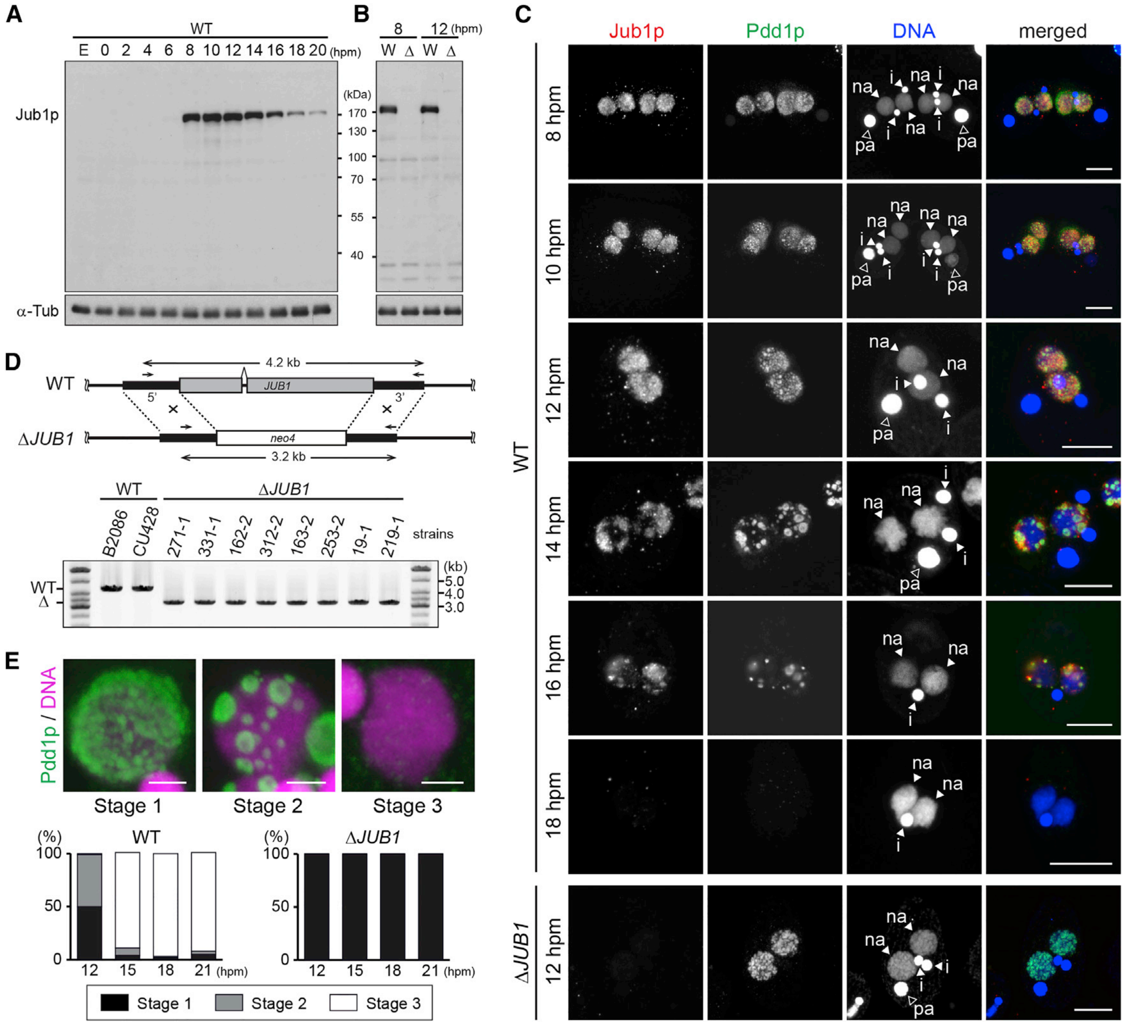

Figure 2. Jub1p Is Required for Heterochromatin Body Formation

(A and B) Exponentially growing (E), starved (0 hpm), or conjugating (2–20 hpm) WT cells (A) and WT (W) and *JUB1* KO (Δ) cells at late-conjugation stages (8–12 hpm) (B) were analyzed by western blot using an anti-Jub1p and an anti-α-Tubulin (α-Tub) antibody.

(C) WT and *JUB1* KO (Δ*JUB1*) cells at the indicated time points were stained with an anti-Jub1p (red) and an anti-Pdd1p (green) antibody. DNA was stained with DAPI (blue). Arrowheads indicate the MIC (i), new MAC (na), and parental MAC (pa). The scale bars represent 10 μm.

(D) The *JUB1* locus in WT and *JUB1* KO (Δ*JUB1*) cells are schematically shown at the top. Replacement of the *JUB1* coding with *neo4* was confirmed by genomic PCR using the primers (arrows).

(E) Three stages of heterochromatin body formation (stage 1, pre-heterochromatin body; stage 2, heterochromatin body; stage 3, post-heterochromatin body) according to Pdd1p localization (green). DNA was stained with DAPI (magenta). The scale bars represent 2 μm. Exconjugants (n = 200) at 12, 15, 18, and 21 hpm from WT and *JUB1* KO cells were analyzed, and the averaged fractions from two independent experiments are shown.

See also Figure S2.

the proper formation of heterochromatin on IESs but is involved directly in heterochromatin body formation.

Heterochromatin Is Important for the IES Localization of Jub1p

We performed ChIP-seq using the anti-Jub1p antibody and found that Jub1p was enriched on IESs in WT cells (Figure 3E). Because Jub1p has no obvious chromatin binding domains, it probably localizes on IESs through interaction with other heterochromatin components. Heterochromatin is mostly disrupted in the absence of the core heterochromatin component Pdd1p, which interacts with H3K9me3 and H3K27me3 and is required for the stable accumulation of these histone modifications (Liu et al., 2007; Taverna et al., 2002). We found that Jub1p was less enriched on IESs in *PDD1* KO cells than in WT cells (Figure 3F). This was not due to an overall reduction or aberrant cellular localization of Jub1p, because Jub1p accumulated normally (Figure 3G) and localized to the new MAC (Figure 3H) in *PDD1* KO cells. Therefore, we conclude that Pdd1p or a Pdd1p-dependent heterochromatin structure is important for the efficient recruitment of Jub1p to IESs.

CellPress

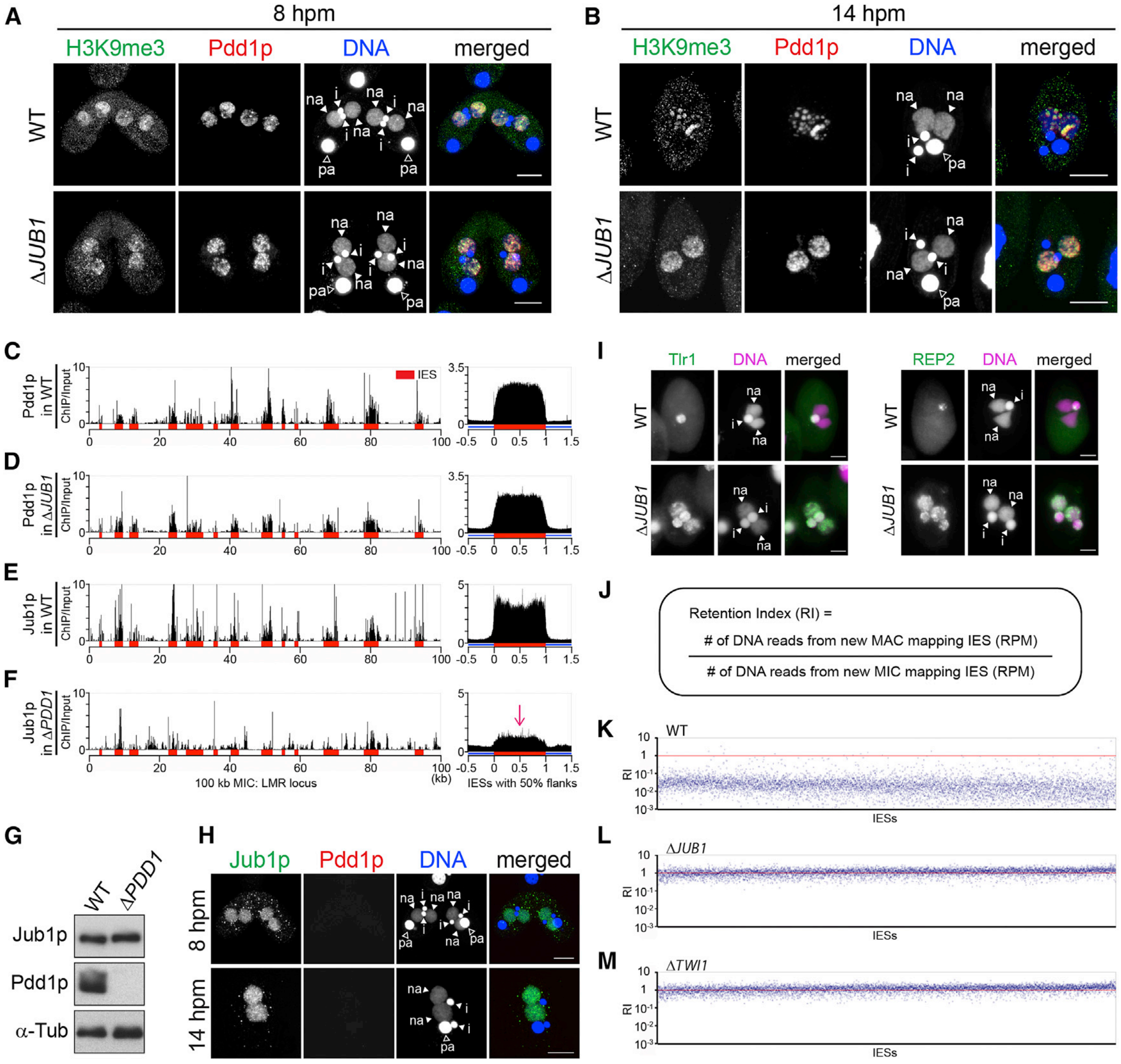

Figure 3. Jub1p Is Required for DNA Elimination but Not for Local Heterochromatin Establishment

(A and B) WT and *JUB1* KO (Δ*JUB1*) cells at 8 hpm (A) and 14 hpm (B) were stained with an anti-H3K9me3 (green) and an anti-Pdd1p (red) antibody. DNA was stained with DAPI (blue). Arrowheads indicate the MIC (i), new MAC (na), and parental MAC (pa). The scale bars represent 10 μm.

(C–F) Fragmented chromatin from the new MACs from WT (C and E), *JUB1* KO (Δ*JUB1*) (D) or *PDD1* KO (*ΔPDD1*) (F) cells at 12 hpm was immunoprecipitated with an anti-Pdd1p (C and D) or an anti-Jub1p (E and F) antibody. ChIP-seq reads were mapped to a 100 kb representative MIC locus (left; LMR locus) or to a modeled IES locus (right), which consisted of all predicted 1 to 5 kb IESs (red) and their flanks (blue). Fold enrichment relative to input is shown.

(G) Proteins from WT and *PDD1* KO (Δ*PDD1*) cells at 12 hpm were analyzed by western blot with an anti-Jub1p, anti-Pdd1p, and anti-α-Tubulin antibody.

(H) *PDD1* KO cells at 8 and 14 hpm were stained with an anti-Jub1p (green) and an anti-Pdd1p (red) antibody, and DNA was stained with DAPI (blue). Arrowheads indicate the MIC (i), new MAC (na), and parental MAC (pa). The scale bar represents 10 μm.

(I) WT and *JUB1* KO (Δ*JUB1*) cells at 36 hpm were hybridized with probes complementary to Tlr1 or REP2 (green). DNA was stained with DAPI (magenta). Arrowheads indicate the MIC (i) and new MAC (na). The scale bar represents 5 μm.

(J–M) RIs were calculated (J) for individual IESs in the new MACs from WT (K), *JUB1* KO (L), and *TWI1* KO (M) at 36 hpm. Red horizontal line indicates RI = 1 (no elimination).

See also Figure S3.

Jub1p Is Required for DNA Elimination and the Production of Viable Sexual Progeny

We next analyzed DNA elimination by DNA-FISH using probes complementary to the two moderately repeated IESs Tlr1 and REP2 (Fillingham et al., 2004; Wuitschick et al., 2002). At 36 hpm, both of the IESs were detected in the new MICs but not in the new MACs in the exconjugants from WT cells (Figure 3I, WT). By contrast, all exconjugants from *JUB1* KO cells showed staining for these IESs in the new MACs (Figure 3I, Δ*JUB1*), indicating that Jub1p is indispensable for DNA elimination of at least the Tlr1 and REP2 IESs.

To assess DNA elimination genome wide, we purified the new MACs from exconjugants at 36 hpm by FACS and analyzed their genomic DNA by high-throughput sequencing. As a reference, we also analyzed purified MICs from vegetative WT cells. As a measure for DNA elimination, a retention index (RI) (Figure 3J)

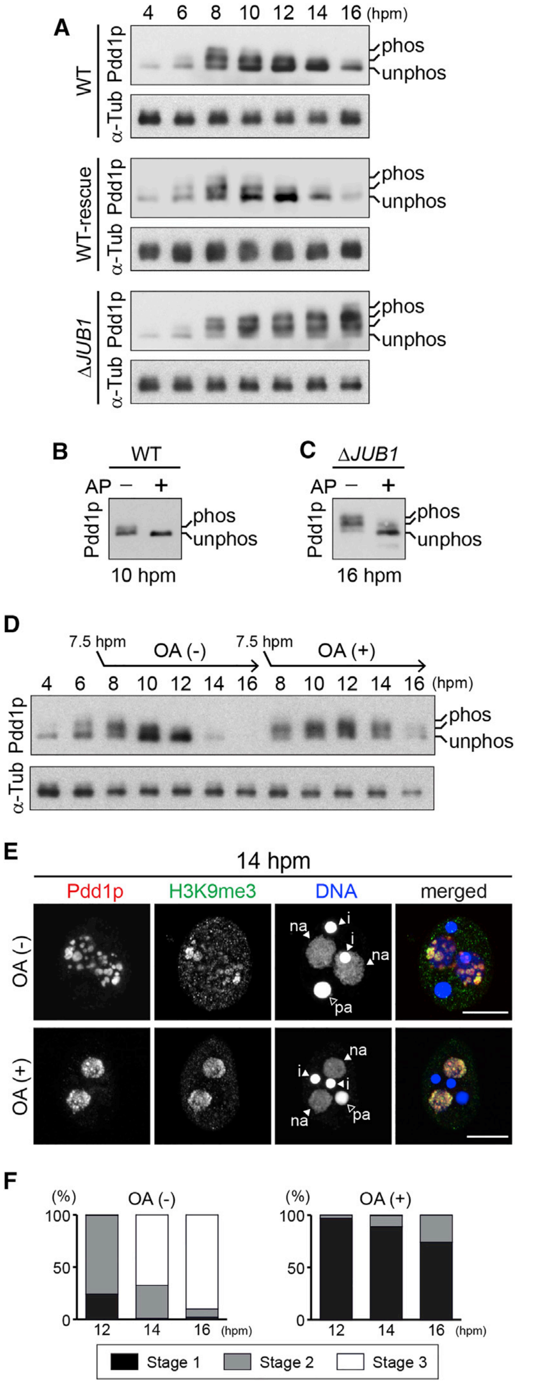

Figure 4. Pdd1p Dephosphorylation Is Inhibited in *JUB1* KO Cells

(A) Proteins from WT, WT-rescue, and *JUB1* KO (Δ*JUB1*) cells in conjugation (4–16 hpm) were analyzed by western blot with an anti-Pdd1p antibody. Phosphorylated (phos) and unphosphorylated (unphos) Pdd1p are indicated. α-Tubulin (α-Tub) was analyzed as a control.

(B and C) Lysates from WT cells at 10 hpm (B) and *JUB1* KO (Δ*JUB1*) cells at 16 hpm (C) were treated with (+) or without (−) alkaline phosphatase and analyzed as in (A).

(D) Conjugating WT cells were treated from 7.5 hpm with (+) or without (−) OA, harvested at indicated time points, and analyzed as in (A).

(E) Cells were treated as in (D) and stained at 14 hpm with anti-Pdd1p (red) and anti-H3K9me3 (green) antibody. DNA was stained with DAPI (blue). Arrowheads indicate the MIC (i), new MAC (na), and parental MAC (pa). The scale bars represent 10 μm.

(F) Cells were treated as in (D), and heterochromatin body in exconjugants (n = 200) were analyzed as in Figure 2E.

See also Figure S4.

was calculated for each IES by dividing the normalized number of reads mapping to an IES from the new MAC sample by those from the reference MIC samples. In the WT new MACs, RIs of most of the IESs were 0.001—0.1 (Figure 3K). Although, in theory, RIs of all IESs should be 0 in WT cells, MIC contamination (~2%–10%) in our new MAC preparations probably made the RI higher. By contrast, in the new MACs from *JUB1* KO cells, most of the IESs had RIs of approximately 1 (Figure 3L), indicating that most, if not all, IESs are retained in the new MAC in the absence of Jub1p. A similar IES elimination defect was detected in *TWI1* KO cells (Figure 3M), in which the RNAi-mediated pathway required for heterochromatin formation is disrupted (Liu et al., 2004; Mochizuki et al., 2002). Altogether, we conclude that Jub1p is essential for the elimination of IESs genome wide.

Consistent with previous reports that DNA elimination is required for the production of viable sexual progeny (Cheng et al., 2010; Horrell and Chalker, 2014; Mochizuki et al., 2002; Nikiforov et al., 1999), *JUB1* KO cells produced no viable sexual progeny, whereas approximately 60% of WT mating pairs produced viable progeny (Figure S3C).

Jub1p Facilitates Dephosphorylation of Pdd1p

In WT cells, at least three differently migrating Pdd1p species were detected by western blot using an anti-Pdd1p antibody at 8 hpm (Figure 4A, WT). As previously reported (Madireddi et al., 1996), all slower migrating Pdd1p disappeared after alkaline phosphatase treatment (Figure 4B), indicating that they were phosphorylated Pdd1p. From 10–14 hpm, these phosphorylated Pdd1p gradually diminished and unphosphorylated Pdd1p increased (Figure 4A, WT). We determined whether this phosphorylated-to-unphosphorylated (phos-unphos) transition is attributable to dephosphorylation of Pdd1p or instead to degradation of phosphorylated Pdd1p accompanied by de novo synthesis of unphosphorylated Pdd1p. To this aim, we analyzed Pdd1p from the WT *PDD1* gene that was introduced into the parental MAC of *PDD1* KO cells (WT-rescue). Although *PDD1* mRNA was expressed until 12 hpm in WT cells (Figure S4A), it was barely detected after 10 hpm in the WT-rescue cells (Figure S4B, WT-rescue). Even in these WT-rescue cells, in which little de novo Pdd1p synthesis was expected after 10 hpm, the phos-unphos transition of Pdd1p occurred between 10 and 12 hpm without a significant reduction in total Pdd1p (Figure 4A, WT-rescue). We therefore conclude that the phos-unphos transition of Pdd1p is mainly caused by dephosphorylation of Pdd1p.

Because the Pdd1p dephosphorylation coincides with heterochromatin body formation (Figures 1C and 2C) and because Jub1p is required for the formation of heterochromatin bodies (Figures 2E and 3B), we hypothesized that Jub1p directs heterochromatin body formation by promoting Pdd1p dephosphorylation. We therefore determined if Jub1p is required for the dephosphorylation of Pdd1p. At 8 hpm, similar amounts of slower migrating Pdd1p species were detected in WT and *JUB1* KO cells (Figure 4A). However, in later stages, these slower

migrating Pdd1p species did not decline in *JUB1* KO cells, and additional slower migrating species appeared (Figure 4A, Δ*JUB1*; 10–16 hpm). All the slower migrating species in *JUB1* KO cells disappeared after alkaline phosphatase treatment (Figure 4C), confirming that they were phosphorylated Pdd1p. Jub1p has no identifiable phosphatase-related domain. Therefore Jub1p facilitates the dephosphorylation of Pdd1p most likely by recruiting some phosphatase(s).

Inhibition of Pdd1p Dephosphorylation Disturbs Heterochromatin Body Formation

To test the importance of the dephosphorylation of Pdd1p, we treated cells with Okadaic acid (OA), an inhibitor for serine (Ser)/threonine (Thr) protein phosphatase 1 and 2A. From 7.5 hpm, just before the highest level of Pdd1p phosphorylation was observed (Figure 4A), conjugating WT cells were incubated with OA. The phosphorylated Pdd1p species remained at least till 16 hpm with OA (Figure 4D, OA+), while they were mostly disappeared by 12 hpm without OA (Figure 4D, OA−), indicating that OA inhibits the dephosphorylation of Pdd1p. In the OA-treated cells, Pdd1p and H3K9me3 were accumulated in the new MAC (Figure 4E), but cells showing heterochromatin body (stage 2) were greatly reduced (Figure 4F). Moreover DNA-FISH analyses for Tlr1 and REP2 IESs showed that most (63% for Tlr1, 87% for REP2) of the OA-treated cells did not finish DNA elimination (Figure S4C). These results are consistent with the idea that Pdd1p dephosphorylation promotes heterochromatin body formation.

Pdd1p Is Mostly Phosphorylated in Unconserved Regions

Although the data above suggest that the dephosphorylation of Pdd1p facilitates heterochromatin body formation, it is also possible that the loss of Jub1p and the OA treatment inhibit heterochromatin body formation independently of Pdd1p dephosphorylation. Therefore, we aimed to analyze the role of Pdd1p dephosphorylation by directly mutating the phosphorylated residues of Pdd1p. For this purpose, we identified the phosphorylated residues of Pdd1p. Pdd1p was immunoprecipitated from WT cells at 8 hpm, when the highest phosphorylation level of Pdd1p was observed (Figure 4A), and mass spectrometry analyses detected 31 phosphorylated Ser/Thr residues in Pdd1p. Another study has identified 10 phosphorylated Ser/Thr residues in Pdd1p (Tian et al., 2014), 2 of which were not identified in our analysis. Therefore, in total, 33 phosphorylated residues of Pdd1p have been identified (Figure 5A, open circles). Pdd1p has 2 chromodomains (CD1 and CD2) and a chromoshadow domain (CSD) (Callebaut et al., 1997), and most (31 of 33) of the identified residues are located in the N-terminal and hinge regions outside of these domains.

Experimental System to Analyze In Vivo Function of Pdd1p

To analyze the function of different Pdd1p mutants in vivo, we established a system in which the KO loci in the MAC of *PDD1* KO cells were replaced by constructs expressing *PDD1* genes from the endogenous *PDD1* promoter (Figure 5B). We validated this system by expressing WT Pdd1p (WT-rescue; Figures 5B and S5A). These WT-rescue strains formed heterochromatin bodies containing Pdd1p and H3K9me3 in the new MACs at 14 hpm (Figure 5C, WT-rescue). A time course study (Figure 5D, WT-rescue) showed that approximately half of the exconjugants from the WT-rescue strains at 12 hpm had heterochromatin bodies (stage 2), and the heterochromatin bodies disappeared (stage 3) from most of the exconjugants by 21 hpm. Whole-genome sequencing of the new MACs at 36 hpm indicated that the DNA elimination defect of the *PDD1* KO cells (Figure 5E) was rescued in the WT-rescue strains (Figure 5F). Consistently, DNA-FISH analysis at 36 hpm indicated that although Tlr1 IESs remained in the new MACs of *PDD1* KO cells, they mostly disappeared in the new MACs of the WT-rescue strains (Figure S5B). Moreover, the WT-rescue cells produced viable sexual progeny (Figure S5C). Altogether, the expression of WT Pdd1p from the parental MAC in the *PDD1* KO background was sufficient to restore all essential processes for DNA elimination, including the formation of heterochromatin bodies. Therefore, the rescue system can be used to analyze the functionalities of Pdd1p mutants in vivo.

Pdd1p Dephosphorylation Promotes Heterochromatin Body Formation by Reducing Net Negative Charge

We generated a series of constructs to express Pdd1p carrying phosphor-mimic mutations, in which 10, 14, 18, or 22 of the identified phosphorylated Ser/Thr residues in the N-terminal and hinge regions (NT, HNG1 and 2, respectively; Figure 5A) were substituted with glutamic acid (Glu) (MIM10, MIM14, MIM18, and MIM22, respectively; Figure 5B), and introduced them into *PDD1* KO cells. Cells expressing MIM10, MIM14, and MIM18 formed exconjugants with heterochromatin bodies at 14 hpm (Figure 5C). Time course analyses (Figure 5D) revealed that although the formation of heterochromatin bodies was delayed in the MIM14 strain, approximately half of the exconjugants from this strain formed heterochromatin bodies (stage 2) at 15 hpm, and the heterochromatin bodies disappeared (stage 3) in some of the exconjugants at later stages. By contrast, in the cells expressing MIM22, no heterochromatin bodies were detected at any of the time points examined (Figures 5C and 5D, MIM22). Nonetheless, Pdd1p and Jub1p accumulated similarly in the WT-rescue and MIM22 strains (Figure S5D) and localized properly on the IESs (Figure 5H). Moreover, in vitro peptide pull-down assay showed that bacterially expressed full-length WT (WT_FL) and the MIM22 mutant (MIM22_FL) Pdd1p similarly bound to the peptides corresponding to H3K9me3 and H3K27me3 (Figure S5E). Therefore, phosphor-mimic mutations in the unconserved regions do not affect the interaction of Pdd1p with the methylated histones. We conclude that the failure of the MIM22 mutant to restore heterochromatin body formation is due neither to instabilities in Pdd1p or Jub1p nor to their absence from chromatin but to a functional disturbance in Pdd1p's heterochromatin body-forming activity. Although the correlative occurrence of the dephosphorylation of Pdd1p and heterochromatin body has been suggested (Madireddi et al., 1996; Shieh and Chalker, 2013), this is the first direct demonstration showing the functional link between the two events.

The results above indicated that the more phosphor-mimic mutations in Pdd1p, the stronger the defects in heterochromatin body formation. We therefore hypothesized that the lack of

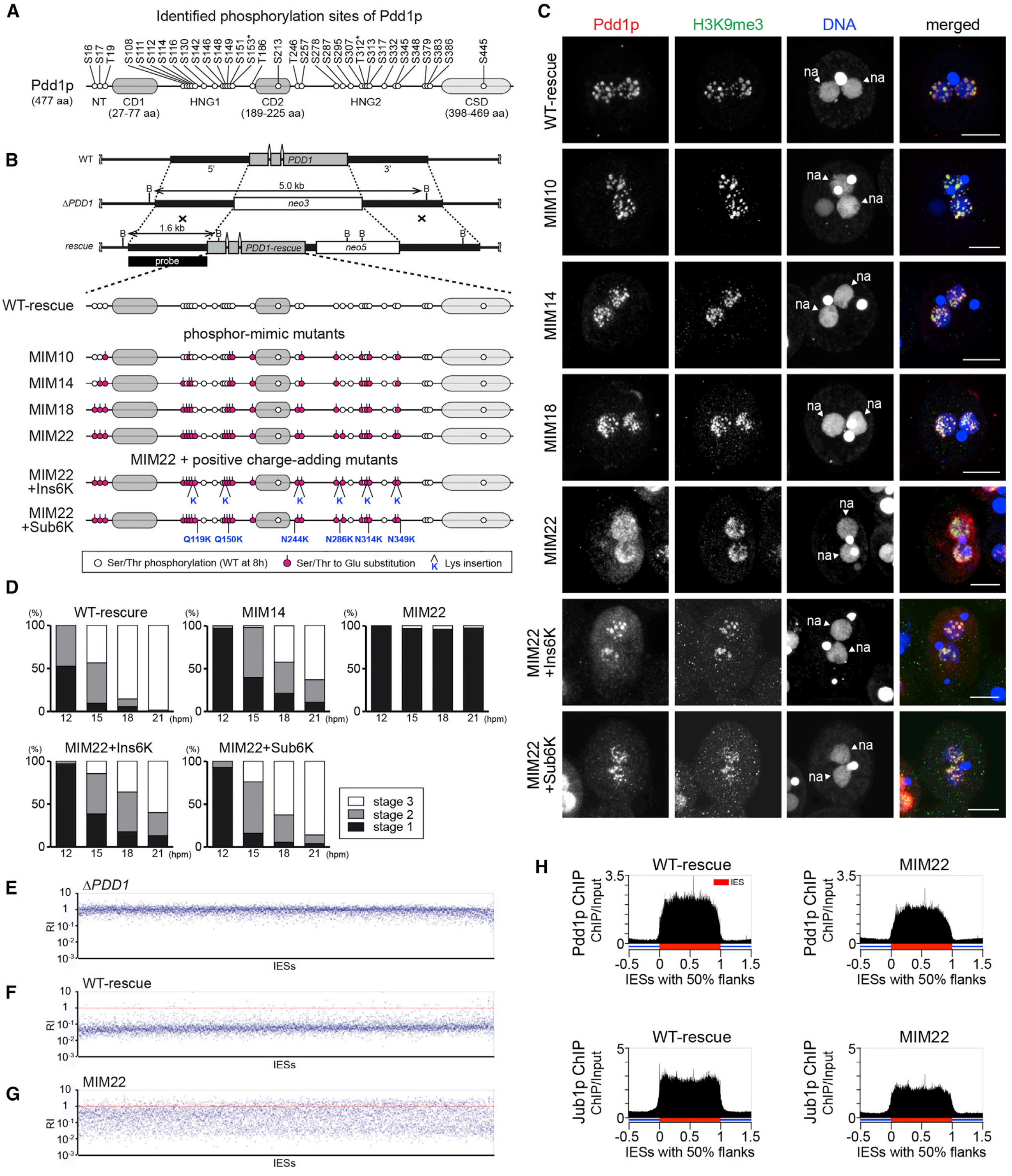

Figure 5. Phosphor-Mimic Pdd1p Mutants Inhibit Heterochromatin Body Formation

(A) Thirty-one phosphorylated Ser (S)/Thr (T) residues of Pdd1p in WT cells at 8 hpm identified in this study and additional 2 residues (marked by asterisks) identified by Tian et al. (2014) are shown as open circles. HNG1 and HNG2 indicate the non-conserved hinge regions between CD1 and CD2 and between CD2 and CSD, respectively.

(B) The WT, KO (Δ*PDD1*), and rescued loci (top) and the proteins expressed from the rescue constructs (bottom). Magenta circles indicate the introduced phosphor-mimic mutations (Ser/Thr to Glu). Lys insertions are indicated with “K,” and substitutions from Gln (Q) or Asn (N) to Lys (K) are indicated with the positions.

(legend continued on next page)

heterochromatin body-forming ability in the phosphor-mimic Pdd1p mutants was due to the increase in their net negative charge. If this was the case, additional mutations supplying positive charges to the phosphor-mimic Pdd1p mutant should restore heterochromatin body formation. To test this idea, we expressed MIM22 with six Lys insertions (MIM22+Ins6K; Figure 5B) or MIM22 with six substitutions of glutamine and asparagine for Lys (MIM22+Sub6K; Figure 5B) in the *PDD1* KO background. In these strains, the formation of heterochromatin bodies was restored to a level similar to the strains expressing MIM14 (Figures 5C and 5D), indicating that the lack of heterochromatin body-forming ability of MIM22 was due not to structural disturbances, if any, caused by the 22 amino acid substitutions but to the constitutive increase in the net negative charge. Altogether, we conclude that the dephosphorylation of Pdd1p facilitates heterochromatin body formation by reducing the net negative charge of Pdd1p.

Pdd1p Dephosphorylation Is Important for DNA Elimination and Progeny Viability

DNA-FISH analyses showed that Tlr1 IESs were completely eliminated in the exconjugants from WT-rescue and MIM10 cells. In contrast, only 40%, 19%, and 1% of the exconjugants from MIM14, MIM18, and MIM22 cells, respectively, completed DNA elimination of the Tlr1 IESs (Figure S5B). The DNA elimination defects of these phosphor-mimic mutants correlated with their abilities to produce viable sexual progeny: 71%, 10%, and 0.3% of mating pairs from WT-rescue, MIM10, and MIM14 cells, respectively, produced viable progeny, and no viable progeny were obtained from MIM18 and MIM22 cells (Figure S5C). Moreover, in the cells expressing the positive charge-added MIM22 mutants (MIM22+Ins6K and MIM22+Sub6K), both IES elimination and progeny production were restored to the level of MIM14 cells (Figures S5B and S5C).

We next assessed genome-wide DNA elimination of the new MACs of MIM22 cells at 36 hpm, as described above. RI scores of different IESs were variable, ranging from 0.01—1 (Figure 5G), indicating that different IESs were affected differently by the MIM22 mutation. This is in contrast with *JUB1* KO cells, in which eliminations of most of the IESs were inhibited (Figure 3L). Therefore, although the MIM22 mutation phenocopied *JUB1* KO in terms of heterochromatin body formation (Figures 2E, 3B, 5C, and 5D) and progeny viability (Figures S3C and S5C), the DNA elimination defect in MIM22 was less severe than that in *JUB1* KO cells. This difference might be because Jub1p can still regulate phosphorylation of the non-mutated Ser/Thr residues of MIM22.

Phosphorylation Does Not Affect the Self-Interaction of Pdd1p

We hypothesized that Pdd1p dephosphorylation might facilitate heterochromatin body formation by promoting direct interaction between the hinge regions of two Pdd1p molecules, which is otherwise inhibited by the negatively charged phosphate groups. To test this idea, we performed pull-down assays using bacterially expressed, WT Pdd1p (WT_FL) and the MIM22 phosphor-mimic Pdd1p mutant (MIM22_FL) (Figure 6A). Maltose binding protein-tagged WT_FL (MBP-WT_FL) and MIM22_FL (MBP-MIM22_FL) were similarly co-precipitated with glutathione S-transferase-tagged WT-FL (GST-WT_FL) (Figure 6B, lanes 9 and 10, asterisks). MBP-WT_FL and MBP-MIM22_FL were also co-precipitated with GST-MIM22_FL (Figure 6B, lanes 11 and 12, asterisks). These results indicate that the phosphor-mimic mutations do not prevent association of two Pdd1p molecules.

Many HP1 family proteins form homodimers through their CSDs (Cowieson et al., 2000). In addition, Swi6, the fission yeast HP1, also multimerizes through its CD (Canzio et al., 2013). We therefore speculated that Pdd1p might also multimerize through a CD-CD or CSD-CSD interaction, which might compensate for the effect of the phosphor-mimic mutations on a hinge-hinge interaction in vitro. To test this possibility, we performed pull-down assays using MBP-Pdd1p carrying either two amino acid substitutions (W50A/W53A) in CD1 or a substitution (I456D) in CSD (Figure 6A) that inhibit the CD-CD and the CSD-CSD interaction in Swi6, respectively (Canzio et al., 2013; Cowieson et al., 2000). The I456D mutation inhibited the co-precipitation of MBP-Pdd1p with GST-WT_FL (Figure 6C, lane 17), but the W50A/W53A mutations did not (Figure 6C, lane 16). Therefore, the in vitro self-interaction of Pdd1p is mediated solely by CSD, and the other domains, including the hinge regions, do not support self-interaction. We conclude that the dephosphorylation of Pdd1p induces heterochromatin body formation not by regulating the multimerization of Pdd1p but through some other mechanism.

The dimerization of HP1 proteins through their CSDs facilitates local heterochromatin compaction (Canzio et al., 2011; Cowieson et al., 2000). Because Pdd1p also homo-multimerizes through its CSD and the phosphor-mimic mutations of Pdd1p do not affect this homo-multimerization, dephosphorylation of Pdd1p is not likely involved in the local compaction of heterochromatin but rather specifically involved in heterochromatin body formation. It has been reported that the Pdd1p CSD mutation I456D causes a defect in heterochromatin body formation in vivo (Schwope and Chalker, 2014). This defect may arise because Pdd1p homo-multimerization is important for the local compaction of heterochromatin, which is a prerequisite for heterochromatin body formation.

Phosphor-Mimic Mutations Inhibit RNA Binding of Pdd1p

The hinge regions of mammalian HP1α and yeast Swi6 bind to non-coding RNAs (ncRNAs) (Keller et al., 2012; Maison et al., 2011), and RNase treatment of permeabilized mouse cells

(C) Cells at 14 hpm were stained with anti-Pdd1p (red) and anti-H3K9me3 (green) antibody, and DNA was stained with DAPI (blue). The new MACs are marked with an arrowhead (na). The scale bars represent 10 μm.
(D) Heterochromatin body in the exconjugants (n = 200) from the indicated *PDD1* mutants was analyzed as in Figure 2E.
(E–G) The RI of each IES in *PDD1* KO (E), WT-rescue (F), and MIM22 (G) strains was calculated as in Figure 3J. Red horizontal line indicates RI = 1 (no elimination).
(H) Localization of Pdd1p and Jub1p in the new MACs of WT-rescue and MIM22 cells at 12 hpm were analyzed by ChIP-seq.
See also Figure S5.

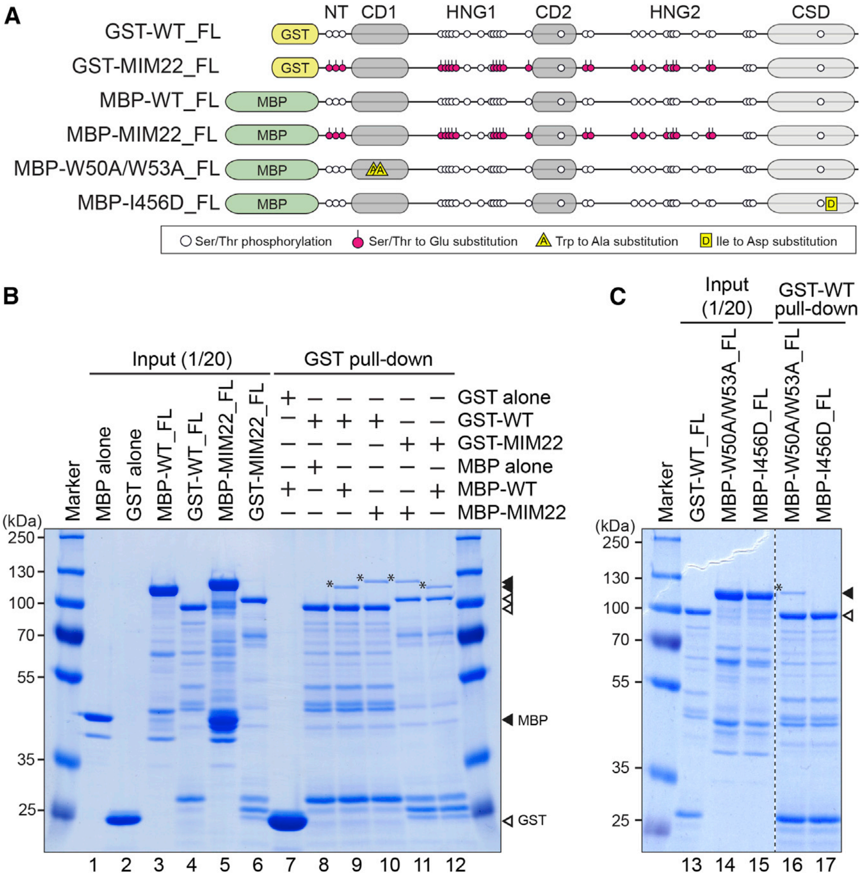

Figure 6. Phosphor-Mimetic Mutations Do Not Inhibit Self-Interaction of Pdd1p

(A) The recombinant Pdd1p proteins used for GST pull-down assays. Magenta circles indicate the introduced phosphor-mimic mutations (Ser/Thr to Glu). Trp to Ala and Ile to Asp substitutions are indicated as yellow triangles and squares, respectively.

(B and C) Input proteins (lanes 1–6 and 13–15) and proteins co-precipitated with the indicated GST-tagged proteins (lanes 7–12 and 16–17) were analyzed by SDS-PAGE followed by Coomassie blue staining. Filled and open arrowheads indicate MBP- and GST-tagged proteins, respectively. Asterisks indicate co-precipitated MBP-tagged proteins.

disassembles the chromocenter (Maison et al., 2002). Because ncRNAs are transcribed from IESs in the new MAC in *Tetrahymena* (Aronica et al., 2008). We thought that ncRNAs might mediate the Pdd1p-Pdd1p interaction and thus the formation of heterochromatin bodies.

We performed an electrophoretic mobility shift assay (EMSA) to test this possibility. The recombinant WT Pdd1p (WT_FL; Figures 7A and 7B) bound to a 723-nt single-stranded RNA (ssRNA) complementary to *EGFP* (*EGFP* ssRNA) (K_d = 65 ± 15 nM) (Figure 7C). Multiple shifts were detected in this assay, indicating that more than one Pdd1p molecule interacts with a single *EGFP* ssRNA. WT_FL also bound to a 1,305-nt ssRNA complementary to Cal IES (Cal IES ssRNA) (Figure S6A). Furthermore, the isolated two hinge regions of Pdd1p (WT_HNG1 and WT_HNG2; Figures 7A and 7B) also bound to the *EGFP* ssRNA (K_d = 130 ± 20 nM, K_d > 630 nM, respectively) (Figure 7D). We conclude that Pdd1p interacts with RNA through its hinge regions in a sequence-independent manner.

Similar EMSA experiments were performed with Pdd1p harboring 14 or 22 phosphor-mimic mutations (MIM14_FL, MIM22_FL; Figures 7A and 7B). Note that the MIM14 and MIM22 show mild and severe heterochromatin body formation defects in vivo, respectively (Figure 5D). MIM14_FL exhibited weaker interaction with the *EGFP* ssRNA (K_d = 340 ± 14 nM) compared with WT-FL, and MIM22_FL showed no detectable interaction with the RNA (Figure 7E). Therefore, the phosphor-mimic mutations disrupt the RNA-Pdd1p interaction. Importantly, MIM22+Ins6K_FL, which had insertions of 6 Lys residues into MIM22_FL (Figures 7A and 7B), interacted with the *EGFP* ssRNA with the affinity (K_d = 347 ± 31 nM) similar to that of MIM14_FL (Figure 7E), indicating that Pdd1p interacts with RNA not via its specific residues but electrostatically through the global positive charge of its hinge regions.

DISCUSSION

In this study, we showed that the heterochromatin body component Jub1p facilitates the dephosphorylation of Pdd1p. Both *JUB1* KO and phosphor-mimic mutations of Pdd1p severely compromised heterochromatin body formation and DNA elimination without affecting local heterochromatin assembly. As far as we know, these mutants are the first experimental system in any eukaryote by which we can genetically separate roles of heterochromatin body from roles of the underlying local heterochromatin. This study therefore provides the first evidence that heterochromatin body per se has an essential biological function.

RNA-Glue Model for Heterochromatin Body Formation in *Tetrahymena*

We propose a model for the molecular mechanism of heterochromatin body assembly during new MAC differentiation in *Tetrahymena* (Figure 7F). First, Pdd1p is deposited onto IESs through its interaction with H3K9/27me to form heterochromatin (step I). We believe Pdd1p is phosphorylated prior to its chromatin deposition because Pdd1p was phosphorylated in *EZL1* and *TWI1* KO cells (Figure S6B), which are defective in H3K9/27me accumulation (Liu et al., 2004, 2007). At this stage (stage 1), heterochromatinized IESs are distributed homogeneously in the new MAC. Then, Jub1p localizes to heterochromatin and recruits a phosphatase (or phosphatases) to trigger Pdd1p dephosphorylation (step II). This dephosphorylation reduces the net negative charge of the hinge regions of Pdd1p and restores its RNA binding activity (step III). Because one RNA molecule can interact with multiple

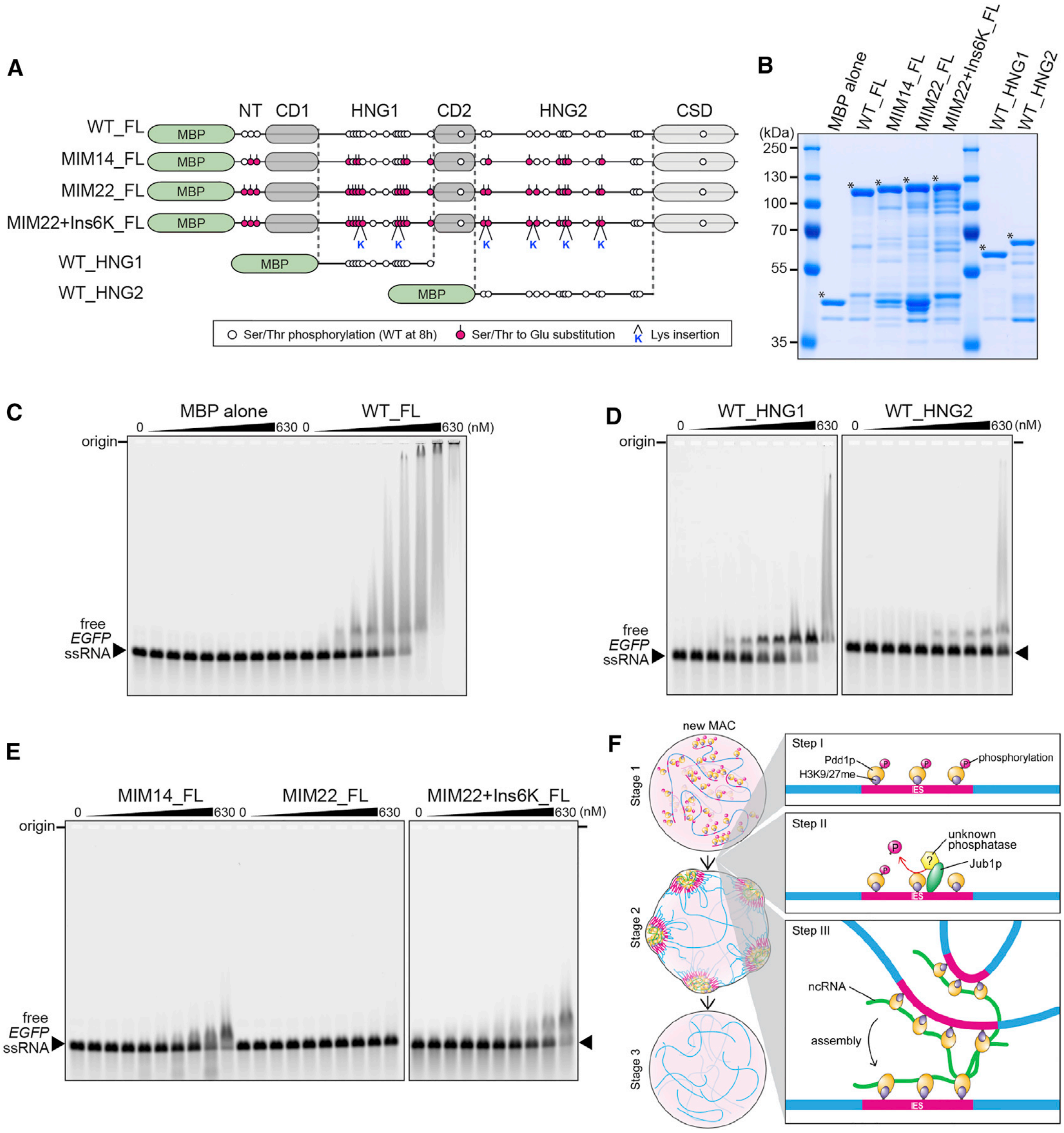

Figure 7. Hinge Regions of Pdd1p Bind to RNA through Their Net-Positive Charge

(A) The recombinant Pdd1p proteins used for EMSA. Magenta circles indicate the introduced phosphor-mimic mutations (Ser/Thr to Glu). The Lys insertions are indicated with "K."

(B) Proteins used were analyzed by SDS-PAGE followed by Coomassie blue staining. Asterisks indicate MBP-tagged Pdd1p proteins or MBP alone.

(C–E) A 723-nt *EGFP* ssRNA (10.4 nM) was titrated with the indicated proteins (0, 10.4, 26.3, 52.5, 78.3, 104, 158, 210, 420, and 630 nM) and separated by agarose gel electrophoresis.

(F) A model for heterochromatin body formation.

See also Figure S6.

Pdd1p molecules (Figure 7C), we propose that the Pdd1p-RNA interaction "glues" multiple IESs into a heterochromatin body (step III, stage 2). Finally, IESs are excised within the heterochromatin body compartments (stage 3).

Although this study clearly demonstrates that the dephosphorylation of Pdd1p plays a pivotal role in heterochromatin body formation, why Pdd1p must be phosphorylated in the first place remains unclear. ChIP-seq analysis indicated that a phosphorylation-defective Pdd1p mutant, in which 26 phosphorylated Ser/Thr residues were substituted with alanine, localized normally on IESs (unpublished data). Therefore, Pdd1p phosphorylation is unlikely to be required for its chromatin deposition but might only be required to regulate the timing of heterochromatin body formation. Alternatively, it may establish a chromatin environment for some downstream event.

Our efforts to identify the phosphatase(s) of Pdd1p have been unsuccessful. It has been proposed that Pdd1p dephosphorylation and heterochromatin body formation are triggered by DNA elimination, based on the observation that UV irradiation induced both of these events in the DNA elimination-defective *LIA5* KO

cells (Shieh and Chalker, 2013). Although this notion is seemingly contradictory to the fact that elimination of most IESs occurs after heterochromatin body formation (Austerberry et al., 1984), some IESs might be eliminated prior to heterochromatin body formation, and DNA damage signaling caused by such DNA elimination might upregulate phosphatase(s) for Pdd1p.

Currently, it is unclear which RNA species interact with Pdd1p in vivo. We previously showed that at least some IESs in the new MAC are transcribed to produce ncRNAs (Aronica et al., 2008). Although these ncRNAs were suggested to be nascent transcripts required for the interaction between the RNAi-machinery and chromatin (Aronica et al., 2008), they might also interact with Pdd1p. Future work should comprehensively identify Pdd1p-associated RNAs in vivo.

Does RNA Glue Heterochromatin Bodies in Other Eukaryotes?

The involvement of ncRNAs in heterochromatin body dynamics is not a new concept but has been reported in other eukaryotes: the mouse HP1α binds to ncRNA from major satellites, which serves as a structural platform for recruiting heterochromatin modulators for the assembly of chromocenters (Maison et al., 2002, 2011); similarly, Xist coats the inactive X chromosome and plays an important role in recruiting factors required for Barr body formation (Hall and Lawrence, 2010). However, it is unclear whether these ncRNAs are directly involved in heterochromatin body formation or indirectly through regulation of local heterochromatin. In *Tetrahymena*, an RNA binding-defective Pdd1p mutant (MIM22) inhibits heterochromatin body formation without affecting local heterochromatin formation in vivo (Figures 5C, 5D, and 5H) and Pdd1p homo-multimerization in vitro (Figure 6B). Therefore, we believe this study provides the first clear demonstration that RNA interaction with a heterochromatin component plays a direct role in heterochromatin body formation.

As for *Tetrahymena* Pdd1p, positively charged residues in the hinge region of HP1 proteins in yeast (Swi6) and mammals (HP1α) are critical for their electrostatic interactions with RNA (Keller et al., 2012; Muchardt et al., 2002), and accumulating evidences from phosphoproteomics suggest that most of the phosphorylated residues of HP1 proteins are in the unconserved region (Dephoure et al., 2008; Shimada et al., 2009; Wilson-Grady et al., 2008; Zhai et al., 2008). Therefore, phosphorylation of HP1 in many eukaryotes might also downregulate their RNA-binding activities as it does in Pdd1p. In this context, it will be interesting to study whether phosphorylations of the hinge regions of HP1 proteins affect their RNA binding and whether such regulation also plays a role in heterochromatin body formation.

EXPERIMENTAL PROCEDURES

For detailed experimental procedures, see Supplemental Experimental Procedures.

Protein and IES Localization Analyses

Immunofluorescent staining and DNA-FISH were performed as described (Loidl and Scherthan, 2004; Noto et al., 2010). For immuno-DNA-FISH, fixed cells were first hybridized with a Cy3-labeled Tlr1 probe and then used for immunofluorescent staining.

ChIP-Seq and DNA Elimination Analysis

For ChIP-seq, the new MACs at 12 hpm were fixed with Di(N-succinimidyl) glutarate and paraformaldehyde and purified by FACS. DNA library was produced from immunoprecipitated chromatin. For genome-wide DNA elimination analysis, DNA libraries were generated from the new MACs from 36 hpm exconjugants and the MICs from starved WT cells. For the both analyses, 50-nt single sequence reads were generated by a HiSeq2000 platform. The MIC genome sequence (version 2) was obtained from the *Tetrahymena* Comparative Sequencing Project (Broad Institute of MIT and Harvard).

Progeny Viability Test

For *JUB1* KO strains, a blasticidin S (bs)-resistance marker was introduced into the MAC. Conjugating pairs were isolated at 8 hpm and bs-sensitive cells were determined as sexual progeny. For the *PDD1* mutants, isolated cells were examined for their resistance to paromomycin without $CdCl_2$.

Identification of Pdd1p Phosphorylation Sites

Pdd1p in WT cells at 8 hpm was immunoprecipitated with an anti-Pdd1p antibody, digested with trypsin, chymotrypsin or subtilisin, and analyzed by mass spectrometry.

Pull-Down Assays

For peptide pull-down assay, MBP-tagged proteins were precipitated with beads coupled with peptides corresponding to the histone H3 N-terminal tail and analyzed by western blot with an anti-MBP antibody. Mean enrichment was calculated from three independent experiments. For GST pull-down assay, GST- and MBP-tagged proteins were mixed, purified with glutathione beads, and analyzed by SDS-PAGE followed by Coomassie blue staining.

EMSA

Fluorescein-12 labeled ssRNA complementary to *EGFP* (723 nt) or Cal IES (1,305 nt) was incubated with MBP-tagged proteins and separated by agarose gel electrophoresis, and the RNA was quantified. Means of dissociation constant were calculated from more than two independent experiments.

ACCESSION NUMBERS

The accession number for the sequence data reported in this paper is GEO: GSE70083.

SUPPLEMENTAL INFORMATION

Supplemental Information includes Supplemental Experimental Procedures, six figures, and one data file and can be found with this article online at http://dx.doi.org/10.1016/j.devcel.2015.11.017.

ACKNOWLEDGMENTS

We thank Hiromi Tagoh (IMP, Vienna, Austria) and the Brennecke group (IMBA, Vienna, Austria) for technical advice and the Next Generation Sequencing unit of Campus Support Facility, Vienna BioCenter, for high-throughput sequencing. This work was supported by a Naito Memorial Grant from the Naito Foundation, a European Research Council Starting Grant (204986) under the European Community's 7th Framework Program, an Austrian Science Fund stand-alone grant (P26032-B22), and core funding from the Austrian Academy of Sciences.

Received: March 19, 2015
Revised: August 29, 2015
Accepted: November 17, 2015
Published: December 10, 2015

REFERENCES

Aronica, L., Bednenko, J., Noto, T., DeSouza, L.V., Siu, K.W., Loidl, J., Pearlman, R.E., Gorovsky, M.A., and Mochizuki, K. (2008). Study of an RNA helicase implicates small RNA-noncoding RNA interactions in programmed DNA elimination in Tetrahymena. Genes Dev. 22, 2228–2241.

Austerberry, C.F., Allis, C.D., and Yao, M.C. (1984). Specific DNA rearrangements in synchronously developing nuclei of Tetrahymena. Proc. Natl. Acad. Sci. U S A *81*, 7383–7387.

Callebaut, I., Courvalin, J.C., Worman, H.J., and Mornon, J.P. (1997). Hydrophobic cluster analysis reveals a third chromodomain in the Tetrahymena Pdd1p protein of the chromo superfamily. Biochem. Biophys. Res. Commun. *235*, 103–107.

Canzio, D., Chang, E.Y., Shankar, S., Kuchenbecker, K.M., Simon, M.D., Madhani, H.D., Narlikar, G.J., and Al-Sady, B. (2011). Chromodomain-mediated oligomerization of HP1 suggests a nucleosome-bridging mechanism for heterochromatin assembly. Mol. Cell *41*, 67–81.

Canzio, D., Liao, M., Naber, N., Pate, E., Larson, A., Wu, S., Marina, D.B., Garcia, J.F., Madhani, H.D., Cooke, R., et al. (2013). A conformational switch in HP1 releases auto-inhibition to drive heterochromatin assembly. Nature *496*, 377–381.

Carone, D.M., and Lawrence, J.B. (2013). Heterochromatin instability in cancer: from the Barr body to satellites and the nuclear periphery. Semin. Cancer Biol. *23*, 99–108.

Chalker, D.L. (2008). Dynamic nuclear reorganization during genome remodeling of Tetrahymena. Biochim. Biophys. Acta *1783*, 2130–2136.

Chalker, D.L., and Yao, M.C. (2011). DNA elimination in ciliates: transposon domestication and genome surveillance. Annu. Rev. Genet. *45*, 227–246.

Chandra, T., Kirschner, K., Thuret, J.Y., Pope, B.D., Ryba, T., Newman, S., Ahmed, K., Samarajiwa, S.A., Salama, R., Carroll, T., et al. (2012). Independence of repressive histone marks and chromatin compaction during senescent heterochromatic layer formation. Mol. Cell *47*, 203–214.

Cheng, C.Y., Vogt, A., Mochizuki, K., and Yao, M.C. (2010). A domesticated piggyBac transposase plays key roles in heterochromatin dynamics and DNA cleavage during programmed DNA deletion in Tetrahymena thermophila. Mol. Biol. Cell *21*, 1753–1762.

Chung, P.H., and Yao, M.C. (2012). Tetrahymena thermophila JMJD3 homolog regulates H3K27 methylation and nuclear differentiation. Eukaryot. Cell *11*, 601–614.

Cowieson, N.P., Partridge, J.F., Allshire, R.C., and McLaughlin, P.J. (2000). Dimerisation of a chromo shadow domain and distinctions from the chromodomain as revealed by structural analysis. Curr. Biol. *10*, 517–525.

Coyne, R.S., Stover, N.A., and Miao, W. (2012). Whole genome studies of Tetrahymena. Methods Cell Biol. *109*, 53–81.

Deng, X., Berletch, J.B., Nguyen, D.K., and Disteche, C.M. (2014). X chromosome regulation: diverse patterns in development, tissues and disease. Nat. Rev. Genet. *15*, 367–378.

Dephoure, N., Zhou, C., Villén, J., Beausoleil, S.A., Bakalarski, C.E., Elledge, S.J., and Gygi, S.P. (2008). A quantitative atlas of mitotic phosphorylation. Proc. Natl. Acad. Sci. U S A *105*, 10762–10767.

Efroni, S., Duttagupta, R., Cheng, J., Dehghani, H., Hoeppner, D.J., Dash, C., Bazett-Jones, D.P., Le Grice, S., McKay, R.D., Buetow, K.H., et al. (2008). Global transcription in pluripotent embryonic stem cells. Cell Stem Cell *2*, 437–447.

Eskeland, R., Leeb, M., Grimes, G.R., Kress, C., Boyle, S., Sproul, D., Gilbert, N., Fan, Y., Skoultchi, A.I., Wutz, A., and Bickmore, W.A. (2010). Ring1B compacts chromatin structure and represses gene expression independent of histone ubiquitination. Mol. Cell *38*, 452–464.

Fillingham, J.S., Thing, T.A., Vythilingum, N., Keuroghlian, A., Bruno, D., Golding, G.B., and Pearlman, R.E. (2004). A non-long terminal repeat retrotransposon family is restricted to the germ line micronucleus of the ciliated protozoan Tetrahymena thermophila. Eukaryot. Cell *3*, 157–169.

Fransz, P.F., and de Jong, J.H. (2002). Chromatin dynamics in plants. Curr. Opin. Plant Biol. *5*, 560–567.

Grau, D.J., Chapman, B.A., Garlick, J.D., Borowsky, M., Francis, N.J., and Kingston, R.E. (2011). Compaction of chromatin by diverse Polycomb group proteins requires localized regions of high charge. Genes Dev. *25*, 2210–2221.

Hall, L.L., and Lawrence, J.B. (2010). XIST RNA and architecture of the inactive X chromosome: implications for the repeat genome. Cold Spring Harb. Symp. Quant. Biol. *75*, 345–356.

Horrell, S.A., and Chalker, D.L. (2014). LIA4 encodes a chromoshadow domain protein required for genomewide DNA rearrangements in Tetrahymena thermophila. Eukaryot. Cell *13*, 1300–1311.

Jenuwein, T., and Allis, C.D. (2001). Translating the histone code. Science *293*, 1074–1080.

Kataoka, K., and Mochizuki, K. (2011). Programmed DNA elimination in Tetrahymena: a small RNA-mediated genome surveillance mechanism. Adv. Exp. Med. Biol. *722*, 156–173.

Keller, C., Adaixo, R., Stunnenberg, R., Woolcock, K.J., Hiller, S., and Bühler, M. (2012). HP1(Swi6) mediates the recognition and destruction of heterochromatic RNA transcripts. Mol. Cell *47*, 215–227.

Kouzarides, T. (2007). Chromatin modifications and their function. Cell *128*, 693–705.

Liu, Y., Mochizuki, K., and Gorovsky, M.A. (2004). Histone H3 lysine 9 methylation is required for DNA elimination in developing macronuclei in Tetrahymena. Proc. Natl. Acad. Sci. U S A *101*, 1679–1684.

Liu, Y., Taverna, S.D., Muratore, T.L., Shabanowitz, J., Hunt, D.F., and Allis, C.D. (2007). RNAi-dependent H3K27 methylation is required for heterochromatin formation and DNA elimination in Tetrahymena. Genes Dev. *21*, 1530–1545.

Loidl, J., and Scherthan, H. (2004). Organization and pairing of meiotic chromosomes in the ciliate Tetrahymena thermophila. J. Cell Sci. *117*, 5791–5801.

Madireddi, M.T., Coyne, R.S., Smothers, J.F., Mickey, K.M., Yao, M.C., and Allis, C.D. (1996). Pdd1p, a novel chromodomain-containing protein, links heterochromatin assembly and DNA elimination in Tetrahymena. Cell *87*, 75–84.

Maison, C., Bailly, D., Peters, A.H., Quivy, J.P., Roche, D., Taddei, A., Lachner, M., Jenuwein, T., and Almouzni, G. (2002). Higher-order structure in pericentric heterochromatin involves a distinct pattern of histone modification and an RNA component. Nat. Genet. *30*, 329–334.

Maison, C., Bailly, D., Roche, D., Montes de Oca, R., Probst, A.V., Vassias, I., Dingli, F., Lombard, B., Loew, D., Quivy, J.P., and Almouzni, G. (2011). SUMOylation promotes de novo targeting of HP1α to pericentric heterochromatin. Nat. Genet. *43*, 220–227.

Mochizuki, K., Fine, N.A., Fujisawa, T., and Gorovsky, M.A. (2002). Analysis of a piwi-related gene implicates small RNAs in genome rearrangement in tetrahymena. Cell *110*, 689–699.

Muchardt, C., Guilleme, M., Seeler, J.S., Trouche, D., Dejean, A., and Yaniv, M. (2002). Coordinated methyl and RNA binding is required for heterochromatin localization of mammalian HP1alpha. EMBO Rep. *3*, 975–981.

Mutskov, V., Gerber, D., Angelov, D., Ausio, J., Workman, J., and Dimitrov, S. (1998). Persistent interactions of core histone tails with nucleosomal DNA following acetylation and transcription factor binding. Mol. Cell. Biol. *18*, 6293–6304.

Narita, M. (2007). Cellular senescence and chromatin organisation. Br. J. Cancer *96*, 686–691.

Nikiforov, M.A., Smothers, J.F., Gorovsky, M.A., and Allis, C.D. (1999). Excision of micronuclear-specific DNA requires parental expression of pdd2p and occurs independently from DNA replication in Tetrahymena thermophila. Genes Dev. *13*, 2852–2862.

Noto, T., Kurth, H.M., Kataoka, K., Aronica, L., DeSouza, L.V., Siu, K.W., Pearlman, R.E., Gorovsky, M.A., and Mochizuki, K. (2010). The Tetrahymena argonaute-binding protein Giw1p directs a mature argonaute-siRNA complex to the nucleus. Cell *140*, 692–703.

Politz, J.C., Scalzo, D., and Groudine, M. (2013). Something silent this way forms: the functional organization of the repressive nuclear compartment. Annu. Rev. Cell Dev. Biol. *29*, 241–270.

Probst, A.V., and Almouzni, G. (2011). Heterochromatin establishment in the context of genome-wide epigenetic reprogramming. Trends Genet. *27*, 177–185.

Schwope, R.M., and Chalker, D.L. (2014). Mutations in Pdd1 reveal distinct requirements for its chromodomain and chromoshadow domain in directing histone methylation and heterochromatin elimination. Eukaryot. Cell *13*, 190–201.

Shieh, A.W., and Chalker, D.L. (2013). LIA5 is required for nuclear reorganization and programmed DNA rearrangements occurring during tetrahymena macronuclear differentiation. PLoS ONE *8*, e75337.

Shimada, A., Dohke, K., Sadaie, M., Shinmyozu, K., Nakayama, J., Urano, T., and Murakami, Y. (2009). Phosphorylation of Swi6/HP1 regulates transcriptional gene silencing at heterochromatin. Genes Dev. *23*, 18–23.

Taverna, S.D., Coyne, R.S., and Allis, C.D. (2002). Methylation of histone h3 at lysine 9 targets programmed DNA elimination in tetrahymena. Cell *110*, 701–711.

Tian, M., Chen, X., Xiong, Q., Xiong, J., Xiao, C., Ge, F., Yang, F., and Miao, W. (2014). Phosphoproteomic analysis of protein phosphorylation networks in Tetrahymena thermophila, a model single-celled organism. Mol. Cell. Proteomics *13*, 503–519.

Vogt, A., and Mochizuki, K. (2013). A domesticated PiggyBac transposase interacts with heterochromatin and catalyzes reproducible DNA elimination in Tetrahymena. PLoS Genet. *9*, e1004032.

Wilson-Grady, J.T., Villén, J., and Gygi, S.P. (2008). Phosphoproteome analysis of fission yeast. J. Proteome Res. *7*, 1088–1097.

Woehrer, S.L., Aronica, L., Suhren, J.H., Busch, C.J., Noto, T., and Mochizuki, K. (2015). A Tetrahymena Hsp90 co-chaperone promotes siRNA loading by ATP-dependent and ATP-independent mechanisms. EMBO J. *34*, 559–577.

Wuitschick, J.D., Gershan, J.A., Lochowicz, A.J., Li, S., and Karrer, K.M. (2002). A novel family of mobile genetic elements is limited to the germline genome in Tetrahymena thermophila. Nucleic Acids Res. *30*, 2524–2537.

Xu, J., Yuan, Y., Liang, A., and Wang, W. (2015). Chromodomain protein Tcd1 is required for macronuclear genome rearrangement and repair in Tetrahymena. Sci. Rep. *5*, 10243.

Zhai, B., Villén, J., Beausoleil, S.A., Mintseris, J., and Gygi, S.P. (2008). Phosphoproteome analysis of Drosophila melanogaster embryos. J. Proteome Res. *7*, 1675–1682.

Zhu, Q., Pao, G.M., Huynh, A.M., Suh, H., Tonnu, N., Nederlof, P.M., Gage, F.H., and Verma, I.M. (2011). BRCA1 tumour suppression occurs via heterochromatin-mediated silencing. Nature *477*, 179–184.

Developmental Cell

Supplemental Information

Phosphorylation of an HP1-like Protein Regulates Heterochromatin Body Assembly for DNA Elimination

Kensuke Kataoka and Kazufumi Mochizuki

Figure S1

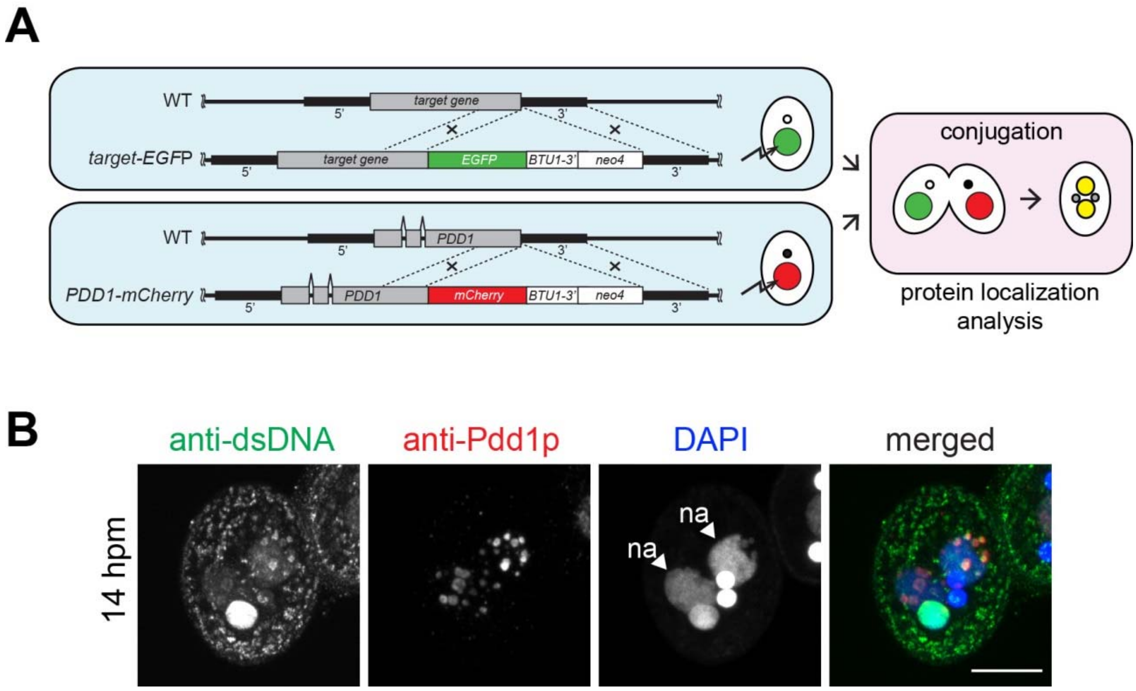

Figure S1, related to Figure 1. Experimental design for the protein localization screen
(A) A schematic representation of the protein localization screen. (Left top) The *EGFP* gene followed by the *BTU1* 3'UTR and the *neo4* drug-resistance cassette were inserted before the stop codon of an endogenous target locus in the MAC by homologous recombination. To visualize heterochromatin bodies, cells expressing an EGFP-tagged protein were crossed with cells expressing Pdd1p-mCherry (Left bottom). The localizations of the EGFP-tagged protein and Pdd1p-mCherry were observable within the same cell because the cytoplasm of conjugating cells is connected. **(B)** A wild-type cell at 14 hpm was immunostained with an anti-double-stranded DNA (dsDNA) antibody (green) and an anti-Pdd1p antibody (red). DNA was counter-stained with DAPI (blue). The new MACs are marked by arrowheads with "na". The scale bar represents 10 μm.

Figure S2

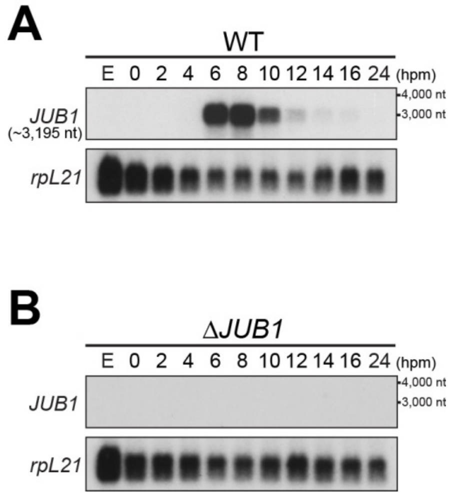

Figure S2, related to Figure 2. Northern blot analyses of *JUB1* mRNA
(A, B) *JUB1* mRNA (~3,195 nt) from exponentially growing (E), starved (0 hpm) or conjugating (2-24 hpm) wild-type (WT) (A) and *JUB1* KO (Δ*JUB1*) (B) cells were analyzed by northern blot. Constitutively expressed *rpL21* was analyzed as a control.

Figure S3

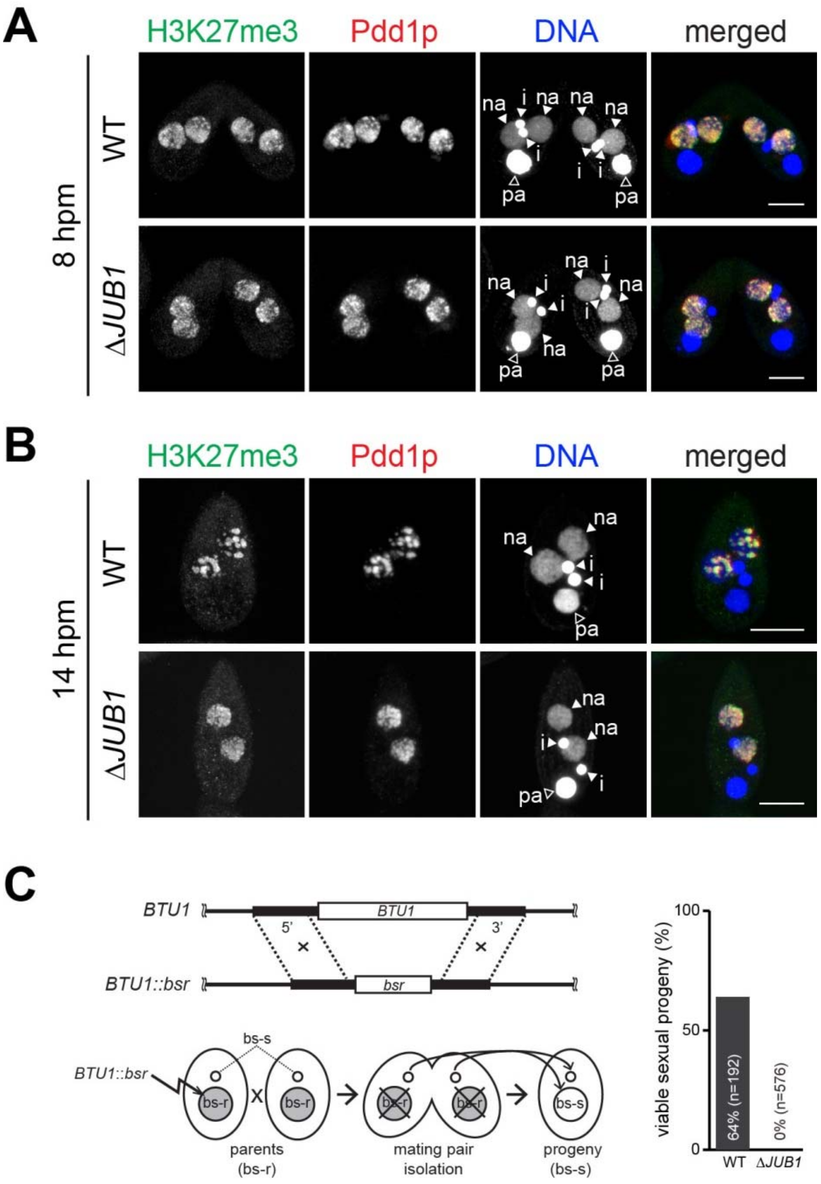

Figure S3, related to Figure 3. Analyses of methylated histones and progeny viability

(A, B) Wild-type (WT) and *JUB1* KO (Δ*JUB1*) cells at 8 hpm (A) and 14 hpm (B) were immunostained with anti-H3K27me3 (green) and anti-Pdd1p (red) antibodies. DNA was counter-stained with DAPI (blue). Arrowheads indicate the MIC (i), new MAC (na) and parental MAC (pa). Scale bars represent 10 µm. **(C)** Viability tests for the sexual progeny of *JUB1* KO cells. (Left top) A schematic representation of the *BTU1* locus. MAC loci encoding the non-essential *BTU1* gene in *JUB1* KO (Δ*JUB1*) and wild-type (WT) cells were replaced with the Blasticidin S (bs) resistance marker cassette *bsr* by homologous recombination. (Left bottom) A schematic representation of the experimental design of the assay. The bs-resistant (bs-r) cells (parents) were induced to conjugate, and the bs sensitivity of the cells grown from the isolated mating pair was assessed. The progeny are bs sensitive (bs-s) because the parental MAC carrying the *bsr* cassette is destroyed during conjugation (See Supplementary Experimental Procedures for details). (Right) The average percentages of isolated mating pairs that produced bs-s progeny from a wild-type cross and 3 independent *JUB1* KO crosses are shown.

Figure S4

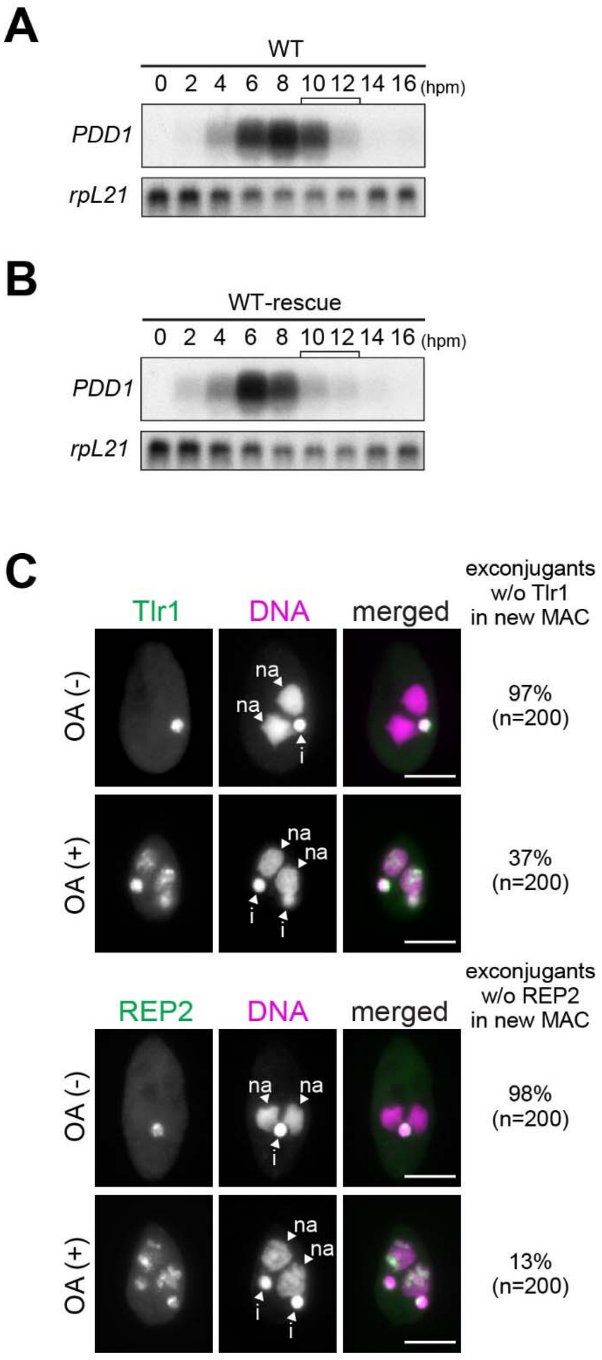

Figure S4, related to Figure 4. Northern blot analyses of *PDD1* mRNA in wild-type and WT-rescue cells, and DNA FISH analyses in wild-type cells treated with a phosphatase inhibitor

(A, B) *PDD1* mRNA from starved (0 hpm) or conjugating (2-16 hpm) wild-type (WT) (A) and WT-rescue (B) cells was analyzed by northern blot. Constitutively expressed rpL21 was analyzed as a control. **(C)** (Left) Exconjugants from wild-type treated with or without Okadaic acid [OA (+) or OA (-)] at 36 hpm were hybridized with probes complementary to Tlr1 or REP2 (green). DNA was counter-stained with DAPI (magenta). Arrowheads indicate the MIC (i), new MAC (na). Scale bar represents 10 µm. (Right) the average percentages of exconjugants that do not have Tlr1 or REP2 signal in the new MAC from 2 independent crosses (n=200) are shown.

Figure S5

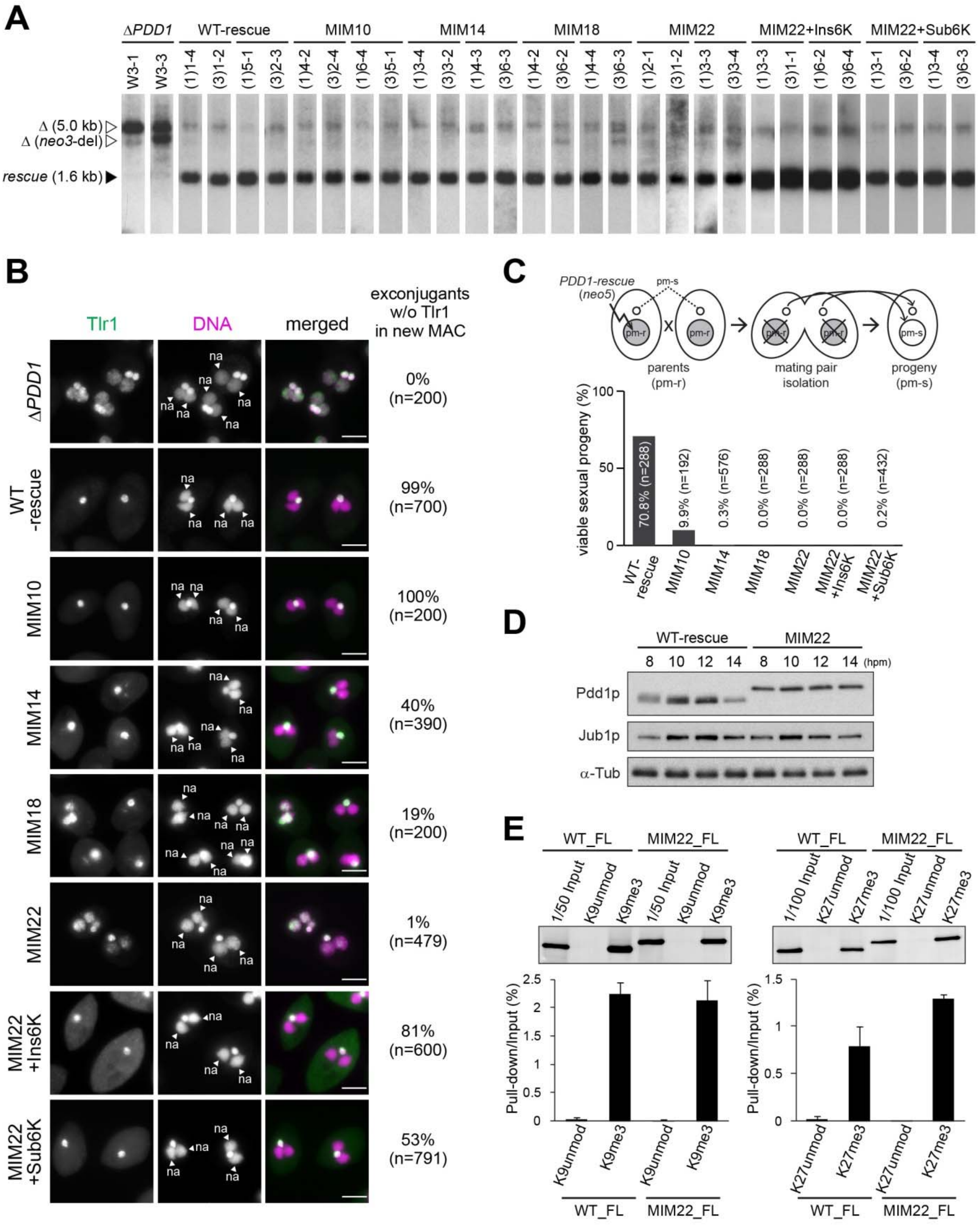

A
ΔPDD1
WT-rescue
MIM10
MIM14
MIM18
MIM22
MIM22+Ins6K
MIM22+Sub6K
Δ (5.0 kb)
Δ (neo3-del)
rescue (1.6 kb)
B
Tlr1
DNA
merged
exconjugants w/o Tlr1 in new MAC
0% (n=200)
99% (n=700)
100% (n=200)
40% (n=390)
19% (n=200)
1% (n=479)
81% (n=600)
53% (n=791)
C
PDD1-rescue (neo5)
pm-s
parents (pm-r)
mating pair isolation
progeny (pm-s)
viable sexual progeny (%)
70.8% (n=288)
9.9% (n=192)
0.3% (n=576)
0.0% (n=288)
0.0% (n=288)
0.0% (n=288)
0.2% (n=432)
D
WT-rescue
MIM22
8 10 12 14 8 10 12 14 (hpm)
Pdd1p
Jub1p
α-Tub
E
WT_FL
MIM22_FL
1/50 Input
K9unmod
K9me3
1/100 Input
K27unmod
K27me3
Pull-down/Input (%)

Figure S5, related to Figure 5. In vivo and in vitro analyses of phosphor-mimic mutants of Pdd1p

(A) Genomic DNA extracted from the rescued strains (two pairs in each rescue strain) was digested with BglII (indicated as "B" in Figure 5B), and the replacements were analyzed by southern blot using the probe depicted in Figure 5B. Background *PDD1* KO strains were also analyzed as a control. The loci from KO and *neo3*-deletion in the background strains are marked with open arrowheads with "Δ (5.0 kb)" and "Δ (*neo3*-del)" (Liu et al., 2005), respectively. The replaced loci (1.6 kb) are marked by filled arrowheads with "rescue". **(B)** Exconjugants at 36 hpm from *PDD1* KO and rescued cells as indicated were hybridized with Tlr1 probe (green). DNA was counter-stained with DAPI (magenta). Arrowheads indicate new MACs (na). Scale bar represents 10 μm. The average percentages of exconjugants that did not show Tlr1 signals in the new MAC from more than 2 independent mating pairs are given. **(C)** Viability tests for the sexual progeny of the rescued cells. (Top) A schematic representation of the experimental design of the assay. The rescued cells (parents), which are paromomycin resistant (pm-r), were induced to conjugate. The mating pairs were isolated, and the pm sensitivity of the cells grown from the isolated mating pair was assessed. The progeny are pm sensitive (pm-s) in the absence of cadmium ions because the parental MAC carrying the *neo5* cassette is destroyed during conjugation (See Supplementary Experimental procedures for details). (Bottom) The average percentages of isolated mating pairs that produced pm-s progeny from more than 2 independent crosses are shown. **(D)** Proteins from WT-rescue and MIM22 cells during late conjugation (8-14 hpm) stages were analyzed by western blot with an anti-Pdd1p antibody and an anti-Jub1p antibody. The western blot using an anti-α-Tubulin (α-Tub) antibody is also shown as a control. **(E)** Histone peptide pull-down assay. MBP-tagged full-length recombinant Pdd1p (WT_FL and MIM22_FL) was pull-downed with beads coupled with peptide corresponding to N-terminal tail of histone H3, that contains unmodified or methylated Lys at 9 (K9unmod or K9me3), or at 27 (K27unmod or K27me3), and was analyzed by western blot using anti-MBP antibody (top). The mean value (±standard deviation) of enrichment relative to Input (Pull-down/Input) form 3 independent experiments is shown (bottom).

Figure S6

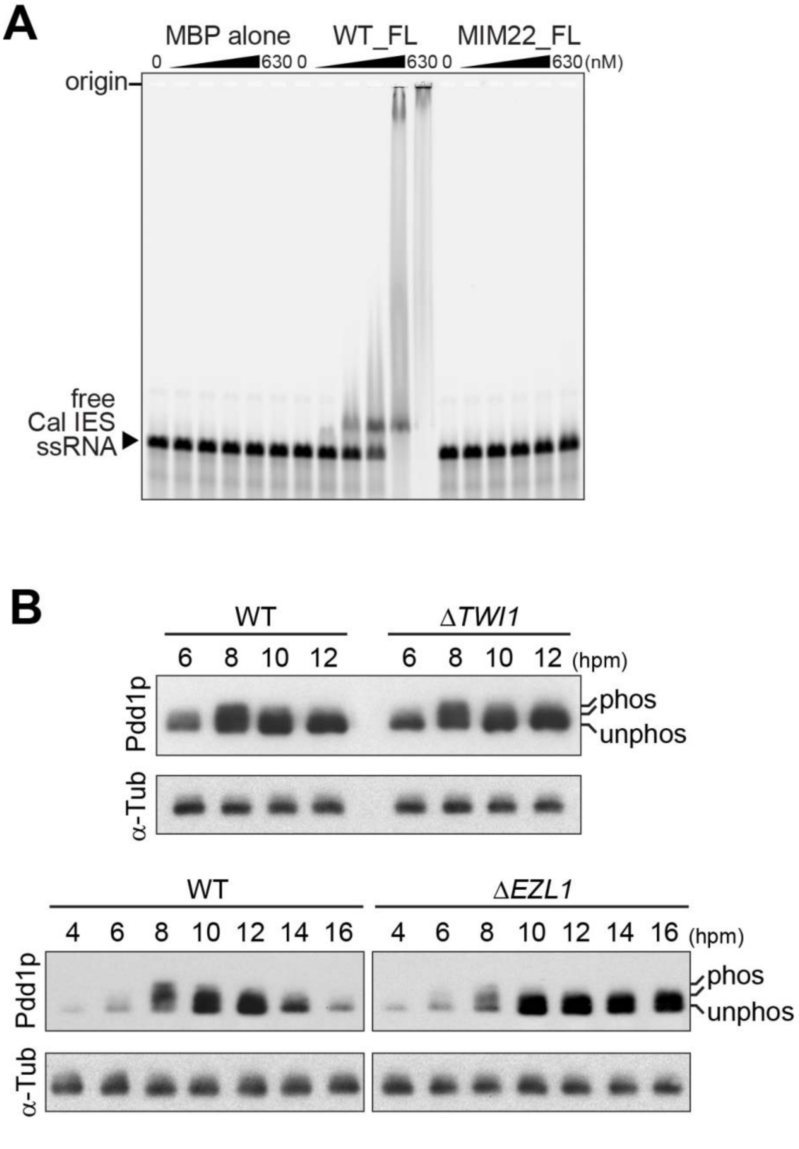

Figure 6S, related to Figure 7. EMSA with Cal IES ssRNA and western blot analyses of Pdd1p phosphorylation in *TWI1* and *EZL1* KO cells

(A) The 1,305-nt Cal IES ssRNA (10.4 nM) was titrated with a recombinant protein as indicated (0, 10.5, 52.5, 104, 420 and 630 nM) and analyzed by agarose gel electrophoresis. **(B)** Proteins from conjugating wild-type (WT), *TWI1* KO (Δ*TWI1*; 6-12 hpm) and *EZL1* KO (Δ*EZL1*; 4-16 hpm) cells were analyzed by western blot with an anti-Pdd1p antibody. Phosphorylated (phos) and unphosphorylated (unphos) Pdd1p are indicated. α-Tubulin (α-Tub) was analyzed as a control.

Supplementary Data S1, related to Figure 1:
1. Nuclear events during the life cycle of *Tetrahymena thermophila* (related to Figure 1)
2. Summary of the protein localization screen (related to Figure 1)
3. Localizations of EGFP-tagged proteins (related to Figure 1): Cells expressing EGFP-tagged proteins (green), which were growing, starved or conjugating with wild-type cells, were fixed and DNA was stained with DAPI (magenta). For conjugation stages (E1-1~L3), see "1. Nuclear events during the life cycle of *Tetrahymena thermophila*"

Supplemental Experimental Procedures

Strains and culture conditions
The wild-type *Tetrahymena thermophila* strains B2086, CU427, CU428 and SB1969 were provided by Dr. P. J. Bruns (Cornell University, USA). The *EZL1* KO strains, *PDD1-mCherry* strain and *TWI1* KO strains were described previously (Aronica et al., 2008; Kataoka et al., 2010; Noto et al., 2010). The germline *PDD1* KO strains (Motl and Chalker, 2011) were provided by Dr. Yifan Liu (University of Michigan, USA). Cells were grown in SPP medium (Gorovsky et al., 1975) containing 2% proteose peptone at 30°C overnight. To induce conjugation, exponentially growing cells (~3-5x 10^5/ml) of two different mating types were starved in 10 mM Tris-HCl (pH 7.5) at 30°C for 8-14 h and mixed for conjugation (7x 10^5/ml) at 30°C.

Protein localization screen
The coding and 3' flanking sequences (~0.5-1 kb) of the target genes (listed in Supplementary Data S1) were amplified from the genomic DNA of the wild-type strain B2086 or CU428 by PCR using PrimeSTAR HS DNA polymerase (Takara) with the primers listed below. Targeting constructs were generated by connecting these genomic DNA fragments and an *EGFP-neo4* module, which was amplified from the plasmid pEGFP-neo4, by overlapping PCR using the primers 5'RACE Outer and 3'RACE Outer as previously described (Kataoka et al., 2010). The construct was introduced into the endogenous MAC locus of a wild-type strain (B2086 or CU428) by biolistic transformation as previously described (Cassidy-Hanley et al., 1997), and a partial replacement was performed by stepwise selection in increasing concentrations up to 25 mg/ml of paromomycin sulfate (pm, Sigma) in the presence of 1 μg/ml $CdCl_2$. These transgenic cells, which were in exponentially growing, starved and conjugating with a wild-type strain (B2086 or CU428) at 2, 4, 6, 8, 10, 12 and 14 hpm, were fixed with 10% formalin and 25% methanol in PBS at 4°C overnight. Localization of the EGFP-tagged proteins was observed in cells counter-stained with 10 ng/ml *4',6-Diamidino-2-phenylindole* (DAPI), and the conjugating cells were categorized by their nuclear morphology according to the developmental stages (see Supplementary Data S1). To compare the localizations of the EGFP-tagged proteins and Pdd1p, cells expressing an EGFP-tagged protein were mated with *PDD1*-mCherry cells.

Primary antibodies
To generate an anti-Jub1p antibody, rabbits were immunized with a peptide (CRSFDKQAQGKKNSS) corresponding to amino acids 838-851 (underlined) of Jub1p (DDBJ/EMBL/GenBank EAS04546.2). The antibody was affinity purified with the peptide, dialyzed against PBS and used for all the analyses of Jub1p performed in this study. To generate an anti-Pdd1p antibody, a guinea pig was immunized with a peptide (CLGSKGQSQQVEKEQATNS) corresponding to amino acids 315-332 (underlined) of Pdd1p (DDBJ/EMBL/GenBank XP_001016207.1). The antibody was purified with protein A, dialyzed against PBS and used for all immunofluorescent staining analyses of Pdd1p and the western blot analysis shown in Figure S5D. For the other western blot analyses, immuno-DNA-FISH and ChIP-Seq, a rabbit anti-Pdd1p antibody (ab5338, Abcam) was used. The mouse anti-dsDNA antibody HYB331-01 (ab27156, Abcam) and rabbit antibodies anti-H3K9me3 (07-442, Merck Millipore) and anti-H3K27me3 (07-449, Merck Millipore) were used for immunofluorescent staining. The rabbit antibody anti-H3K4me3 (ab8580, Abcam) was used for immuno-DNA-FISH. The mouse anti-alpha-tubulin antibody 12G10 was obtained from the Developmental Studies Hybridoma Bank (University of Iowa, USA) and used for western blot analyses.

Immunofluorescent staining
Cells were fixed and processed as previously described (Loidl and Scherthan, 2004) with modifications. Briefly, cells were fixed with 10% formalin in 10 mM Tris-HCl (pH 7.5) at room temperature for 10 min. Subsequently, Triton X-100 was added (final concentration of 0.25%), and the cells were incubated at room temperature for 20 min. The fixed cells were resuspended in 10% formalin containing 3.4% sucrose and air-dried on Superfrost Ultra Plus slides (Thermo Scientific). For immunofluorescent staining, the fixed cells were incubated with the mouse 1:500 anti-dsDNA antibody, rabbit antibodies (1:1000 anti-Jub1p, 1:500 anti-H3K9me3 or 1:1000 anti-H3K27me3) or 1:1000 guinea pig anti-Pdd1p antibody at 4°C overnight and then incubated with secondary antibodies (1:1000 anti-mouse, anti-rabbit or anti-guinea pig IgG conjugated with Alexa-488, -568 or -647

[Invitrogen]) at room temperature for 2 h. They were then counter-stained with 40 ng/ml DAPI and observed using an epifluorescence microscope and a confocal laser scanning microscope.

DNA-FISH and immuno-DNA-FISH

Cells were fixed and processed as described above, and DNA-FISH was performed as previously described (Loidl and Scherthan, 2004). Cy3-labeled probes for Tlr1 and REP2 IESs were produced as previously described (Noto et al., 2010). For immuno-DNA-FISH, the fixed cells were first hybridized with a Cy3-labeled Tlr1 probe. Subsequently, immunofluorescent staining was performed with a rabbit primary antibody and with an anti-rabbit IgG secondary antibody conjugated with Alexa-488 (Invitrogen) as described above.

Establishment of germ line *JUB1* KO strains

To create the knockout construct for *JUB1*, 5' and 3' flanking regions of the *JUB1* gene were amplified by PCR using the primer sets 00237610_KO5Fw/00237610_KO5Rv and 00237610_KO3Fw/3Am-00237610_Rv, respectively (primer sequences are listed below). The pm-resistant cassette *neo4* was isolated from the pNeo4 plasmid (Mochizuki, 2008) by SmaI digestion. The PCR products and the *neo4* cassette were connected by overlapping PCR using the PCR Extender System (5 PRIME) with the primers 00237610_KO5Fw and 3Am-00237610_Rv. The resulting targeting construct was introduced into the MIC of mating cells (B2086 and CU428) by biolistic transformation as described previously (Cassidy-Hanley et al., 1997), and a heterozygous progeny was selected with pm and 6-methylpurine in the presence of 1 μg/ml $CdCl_2$. To obtain heterozygous strains expressing different mating types, the heterozygous strain was mated with the wild-type strain CU427 or SB1969, and the heterozygous sexual progeny were selected with 0.1 mg/ml pm and 15 μg/ml cycloheximide in the presence of 1 μg/ml $CdCl_2$. The resulting heterozygous strains were cultured for 10 passages without pm, and pm-sensitive (pm-s) heterozygous strains were isolated. The pm-s heterozygous cells expressing different mating types were mated, and the sexual progeny were selected with 0.1 mg/ml pm in the presence of 1 μg/ml $CdCl_2$. The *JUB1* locus of the sexual progeny was amplified by PCR with the primers 00237610_Fw9 and 3Am-00237610_Rv, and complete homozygous KO strains were selected. The losses of the *JUB1* mRNA and Jub1p in the KO strains were confirmed by northern blot using a radiolabeled probe, which was generated from DNA amplified from cDNA by PCR with the primers 00237610_Fw1 and 00237610_Rv4 and by western blot analysis using the anti-Jub1p antibody.

ChIP-Seq

Nuclei from cells at 12 hpm were dissociated and stained with DAPI in TMSN buffer (0.25 M sucrose, 10 mM Tris-HCl pH 7.5, 10 mM $MgCl_2$, 3 mM $CaCl_2$, 0.016% NP-40, 1 mM PMSF, 1x complete proteinase inhibitor cocktail (Roche), 0.1 μg/ml DAPI) using a dounce homogenizer with 15 strokes. The nuclear fraction was collected by centrifugation at 4,500 g at 4°C for 5 min and then washed with TMSN buffer once and with TMSN(-) buffer (TMSN buffer without complete proteinase inhibitor cocktail) twice. The nuclei were fixed with 1.25 mg/ml Di(N-succinimidyl) glutarate (DSG) (Sigma) in PBSN buffer (PBS with 0.016% NP-40) at room temperature for 30 min, collected by centrifugation at 9,000 g at 4°C for 5 min, and washed with PBSN buffer twice. The DSG-fixed nuclei were re-fixed with 1% paraformaldehyde (Sigma) in PBSN buffer at room temperature for 10 min. The paraformaldehyde was quenched with 0.25 M glycine at room temperature for 5 min. The nuclei were washed with PBSN buffer containing 0.1 μg/ml DAPI twice and then with PBSN(+) buffer (PBSN with 1x complete proteinase inhibitor cocktail and 0.1 μg/ml DAPI) once, and the nuclear pellet was stored at -80°C. The nuclei were resuspended in PBSN(+), and the new MACs were collected according to their size and DAPI intensity using a FACSAria III (BD Biosciences). The purity of the collected new MACs was assessed by immuno-DNA-FISH using a Cy3-labeled probe against Tlr1 and an anti-Pdd1p antibody (Abcam) for wild-type, *JUB1* KO, WT-rescue and MIM22 cells or an anti-H3K4me3 (Abcam) antibody for *PDD1* KO cells: Tlr1-negative nuclei were counted as parental (old) MACs; Tlr1-positive, and Pdd1p- or H3K4me3-positive nuclei were counted as new MACs; Tlr1-positive but Pdd1p- or H3K4me3-negative nuclei were counted as MICs. Contamination of the parental MACs was rare (<2%), and most of the contaminants in our new MAC preparations were MICs. Chromatin immunoprecipitation was performed with the new MACs only when their purity was above 80%. Chromatin from the new MACs was sheared into 150-500 bp fragments (peak ~300 bp) in Sonication buffer (50 mM Tris-HCl pH 8.0, 10 mM EDTA, 0.1% SDS, 1x complete protease inhibitor cocktail, 1 mM PMSF) using a probe sonicator. For ChIP, 20 mg of Protein A Sepharose CL-4B beads (GE Healthcare) was washed with TE twice and with ChIP buffer (25 mM Tris-HCl pH 8.0, 167 mM NaCl, 5 mM EDTA, 0.05% SDS, 1% Triton X-100, 1x complete protease inhibitor cocktail, 1 mM PMSF) 3 times and was then pre-incubated with 90 μl of ChIP buffer containing 10 μl of 10 mg/ml BSA (NEB) (total ~200 μl suspension) at 4°C for 1 h. The fragmented chromatin from 2 million new MACs in Sonication buffer was diluted with an equal volume of 2x ChIP dilution buffer (334 mM NaCl, 2% Triton X-100, 1x complete protease inhibitor cocktail), adjusted to 1 ml with ChIP buffer and incubated with a 25 μl suspension of the beads at 4°C for 2 h for the pre-absorption. To couple an antibody to the beads, the 10 μl suspension of the beads pre-incubated with BSA was incubated with an antibody (50 μl anti-Jub1p or 5 μg anti-Pdd1p) in 0.5 ml of ChIP buffer at 4°C for 2 h and was then washed with ChIP buffer 3 times. The pre-absorbed chromatin was incubated with the antibody-coupled beads at 4°C overnight. The beads were washed once with Low-salt wash buffer (20 mM Tris-HCl pH 8.0, 150 mM NaCl, 2 mM EDTA, 0.1% SDS, 1% Triton X-100) and

twice each with High-salt buffer (20 mM Tris-HCl pH 8.0, 500 mM NaCl, 2 mM EDTA, 0.1% SDS, 1% Triton X-100), LiCl wash buffer (10 mM Tris-HCl pH 8.0, 0.25 M LiCl, 1 mM EDTA, 1% sodium deoxycholate, 1% NP-40) and TE. The immunoprecipitated chromatin was eluted with Elution buffer (0.1 M NaHCO3, 1% SDS). For cross-linking reversal, the NaCl concentration of the sample was adjusted to 200 mM, and the sample was incubated at 65°C for ~12 h. The DNA was extracted, and DNA libraries were generated from the immunoprecipitated DNA and 20 ng of the fragmented chromatin DNA without immunoprecipitation (Input) using the NEBNext DNA Library Prep Reagent Set for Illumina (NEB) and the KAPA Real-Time Library Amplification Kit (KAPA Biosystems). The libraries were sequenced using the HiSeq2000 platform (Illumina) with 50-nt single-end reads. The first 36 nucleotides of sequence from the reads were used in the downstream analyses. We obtained more than 18 million reads from the ChIP samples and more than 35 million reads from the Input samples that uniquely mapped to the draft MIC genome. The raw and processed sequence data sets have been deposited at the NCBI Gene Expression Omnibus (www.ncbi.nih.gov/geo/) as GSE70083. The numbers of normalized sequence reads (RPMs) from the ChIP sample that mapped to each 100-bp bin were divided by the numbers of reads from the Input sample, and these normalized scores were mapped to a representative 100-kb MIC locus, the LMR, as previously described, (Schoeberl et al., 2012) and to a modeled IES consisting of the 5,606 predicted IESs that range from 1-5 kb in length along with their franking sequences.

Genome-wide DNA elimination analysis

Nuclei from exconjugants at 36 hpm and from wild-type vegetative cells (B2086 and CU428) that were starved overnight at 30°C were stained with DAPI and dissociated as described in the ChIP-Seq procedures above. After the nuclear dissociation, the nuclear fraction was collected by centrifugation at 4,500 g at 4°C for 5 min, washed with TMSN(-) buffer without PMSF 3 times, and stored at -80˚C. The nuclear fraction was resuspended in TMSN buffer containing 2 mM EDTA without PMSF, and the MICs from vegetative cells and the new MACs from exconjugants were collected according to their size and DAPI intensity using a FACSAria III (BD Biosciences). The purities of the collected new MAC and MIC samples were assessed by immunofluorescent staining using an anti-H3K4me3 antibody and DAPI staining: H3K4me3-negative nuclei were counted as MICs; H3K4me3-positive, DAPI-poor small nuclei were counted as new MACs; and H3K4me3-positive, DAPI-rich large nuclei were counted as parental MACs. The purity of the MIC sample was >98%, and the rare contamination was from fragmented MACs. Contamination of the parental MACs in the isolated new MAC sample was rare (<1%), and most of the contaminants in our new MAC preparations were MICs. Genomic DNA libraries were constructed from the MIC samples and the new MAC samples only when the purity was above 90%. Genomic DNAs were extracted from the MICs and the new MACs and sonicated into ~250 bp fragments. Genomic DNA libraries were generated from 20 ng of the fragmented DNA using the NEBNext DNA Library Prep Reagent Set for Illumina (NEB) and the KAPA Real-Time Library Amplification Kit (KAPA Biosystems). The libraries were sequenced using the HiSeq2000 platform with 50-nt single-end reads. The first 36 nucleotides of the sequence of reads were used in the downstream analyses. We obtained more than 48 million reads that uniquely mapped to the draft MIC genome. The raw and processed sequence data sets have been deposited at the NCBI Gene Expression Omnibus (www.ncbi.nih.gov/geo/) as GSE70083. Of the 8,752 previously predicted IESs (Schoeberl et al., 2012), 8,074 are longer than 300 nt and contain more than 100-nt A/C/G/T bases that were used to map the sequence reads. The numbers of normalized sequence reads (RPMs) from the purified new MACs that mapped to each IES were calculated and divided by the average numbers of reads from the purified, wild-type-derived MICs (from B2086 and CU428) that mapped to the corresponding IES to give the Retention Indexes (RIs) of the IESs.

Viability test for sexual progeny

To introduce a drug resistance marker into the MAC of the *JUB1* KO strains to distinguish sexual progeny from parental cells, the 5' and 3' flanking regions of the non-essential *BTU1* gene were amplified from wild-type genomic DNA using the primer sets BTU1_5Fw/BTU1_5bsrRv and BTU1_3bsrFw/BTU1_3Rv, respectively (primer sequences are listed below). The Blasticidin S (bs)-resistance marker cassette *bsr*, which consists of the *HHF1* promoter followed by the bs resistance gene and the *BTU2* 3' UTR (Iwamoto et al., 2009), was amplified from the pBla1 plasmid (a gift from Dr. Masaaki Iwamoto and Dr. Tokuko Haraguchi, KARC, NICT, Japan) with T3 and T7 primers. These PCR products, the 5' and 3' flanking regions of *BTU1* and *bsr*, were connected by overlapping PCR with the primers BTU1_5Fw and BTU1_3Rv. The resulting targeting construct was introduced into the MAC of wild-type (B2086 and CU428) and *JUB1* KO strains by biolistic transformation as previously described (Cassidy-Hanley et al., 1997). The targeted loci were replaced into the transgene by stepwise selection with increasing concentrations of bs (0.3-10 mg/ml, Invivogen) in the absence of $CdCl_2$. The partial assortment was confirmed by genomic PCR using the primer set BTU1_5Fw/BTU1_3Rv. Conjugation of the transformants was induced, and 192 conjugating pairs were isolated in SPP medium at 8 hpm. The cells grown from the isolated pairs were examined for their resistance to 0.3 mg/ml bs in SPP medium, and the bs-sensitive cells were determined as sexual progeny. For the phosphor-mimic mutants, conjugating pairs were isolated from each mating cross at 8 hpm in SPP medium, and the cells that grew were examined for their resistance to 0.5 mg/ml pm in SPP medium without $CdCl_2$. The parental cells were pm-resistant under this condition (without cadmium ions), and the pm-sensitive cells were determined as sexual progeny.

Dephosphorylation of Pdd1p for western blots

Wild-type cells (B2086 and CU428) at 10 hpm and *JUB1* KO cells at 16 hpm were lysed by sonication in 20 mM Tris-HCl pH 7.5, 100 mM NaCl, 2 mM $MgCl_2$, 2 mM $CaCl_2$, 0.1% Tween-20, 0.05 mM PMSF and 1x complete protease inhibitor cocktail (Roche). Pre-cleared lysate was incubated with or without 0.15 U/µl FastAP (Thermo Scientific) at 37°C for 20 min. An equal volume of 2x SDS sample buffer was added to the lysate, which was then incubated at 95°C for 10 min and analyzed by western blotting with a rabbit anti-Pdd1p antibody (Abcam).

Phosphatase inhibitor treatment

Okadaic acid (495604, Merck Millipore) was dissolved in DMSO (1 mM). Conjugating wild-type cells (B2086 and CU428) were incubated from 7.5 hpm with 10 µM Okadaic acid in 10 mM Tris-HCl (pH 7.5) at 30°C. As a control, the cells were treated with the same amount of DMSO without Okadaic acid.

Identification of Pdd1p phosphorylation sites

Conjugating wild-type cells (B2086 and CU428) (5.6x 10^7) at 8 hpm were lysed by sonication in 20 mM Tris-HCl pH 7.5, 100 mM NaCl, 2 mM $MgCl_2$, 2 mM $CaCl_2$, 0.1% Tween-20, 0.05 mM PMSF, 1.25x complete protease inhibitor cocktail (Roche) and 2x PhosSTOP phosphatase inhibitor cocktail (Roche), and the lysate was cleared by centrifugation at 20,000 g at 4°C for 30 min. To immunoprecipitate Pdd1p, a 100 µl suspension of Affi-Prep Protein A Support beads (Bio-Rad) were incubated with 28.5 µg of anti-Pdd1p antibody (Abcam) at 4°C overnight in PBST (PBS containing 0.04% Triton X-100) and washed 3 times each with PBST and with 0.2 M sodium borate pH 9.2. The antibody was cross-linked to the beads by incubating with 20 mM DMP (dimethyl pimelimidate dihydrochloride) in 0.2 M sodium borate pH 9.2 at room temperature for 30 min, and the beads were washed twice each with 0.2 M Tris-HCl pH 8.0 and with PBST. To remove uncross-linked antibody, the beads were washed twice with 0.1 M glycine pH 2.0 followed by washing with PBST three times. The beads were incubated with the pre-cleaned lysate at 4°C for 4.5 h and washed eight times with Washing buffer (20 mM Tris-HCl pH 7.5, 500 mM NaCl, 2 mM $MgCl_2$, 2 mM $CaCl_2$, 1% Triton X-100), three times with Washing buffer without Triton X-100 and once with 150 mM NaCl. The immunopurified Pdd1p was eluted with 0.1 M glycine pH 2. After adjusting the pH to 8, the immunoprecipitated Pdd1p was digested with trypsin, chymotrypsin or subtilisin. The digested peptides were analyzed by an LTQ Orbitrap XL mass spectrometer (Thermo Scientific) coupled with the UltiMate 3000 HPLC system (Dionex). The resulting molecular masses were searched against the Pdd1p sequence using Mascot (Matrix Science) with the additional mass of phosphorylation. The tandem mass spectra showing Mascot scores >10 and those showing Mascot scores from 5-9 and probability >90% as analyzed by phosphoRS (Taus et al., 2011) were selected for phosphorylation site assignment.

Phosphor-mimic *PDD1* mutant strains

To create a plasmid backbone for the rescue construct, the 5' and 3' franking regions of the *PDD1* gene were amplified from CU428 genomic DNA by PCR with the primer sets BX-5FNK_PDD1_FwL/SSB-5FNK_PDD1_Rv and SSS-3FNK_PDD1_Fw/KX-3FNK_PDD1_RvL, respectively (primer sequences are listed below). These two PCR products were connected by overlapping PCR using the primers BX-5FNK_PDD1_FwL and KX-3FNK_PDD1_RvL, digested with BlnI and KpnI and inserted into the SpeI and KpnI sites of pBluescript SK(+). The pm resistance cassette *neo5* franked by loxP sequences (Busch et al., 2010) was inserted into the plasmid at the SalI and SmaI sites followed by the insertion of the *PDD1* 3' UTR, which was amplified from CU428 genomic DNA by PCR using the primer set SpeI-PDD1_3UTR_Fw/SalI-PDD1_3UTR_Rv, at the SpeI and SalI sites. The resulting plasmid backbone was named pPPLNP1 (see below for the sequence). *PDD1* coding sequences were amplified from CU428 genomic DNA for the *WT-rescue* construct or amplified from synthesized DNA for the phosphor-mimic mutants *MIM10*, *14*, *18* and *22* (Genscript) and for the positive charge-added mutants *MIM22+Ins6K* and *MIM22+Sub6K* (Genscript) by PCR with the primer set BamHI-PDD1ORF_Fw/SpeI-PDD1ORF_Rv2. The sequences of *MIM10*, *MIM14*, *MIM18*, *MIM22*, *MIM22+Ins6K* and *MIM22+Sub6K* are listed below. The PCR products were inserted into the pPPLNP plasmid at the BamHI and SpeI sites. The resulting plasmids containing the targeting constructs were linearized by XhoI and transformed into the MACs of *PDD1* KO strains by biolistic transformation as previously described (Cassidy-Hanley et al., 1997). *PDD1* KO loci in the MAC were replaced almost completely by the transgene through stepwise selection with increasing concentrations of pm (0.5-60 mg/ml) in the absence of $CdCl_2$. The replacement was analyzed by southern blot in which genomic DNA from isolated transformants was digested with BglII and hybridized to a radiolabeled probe that was produced from wild-type genomic DNA amplified by PCR with the primers BX-5FNK_PDD1_FwL and SSB-5FNK_PDD1_Rv. The expression of *PDD1* mRNA in wild-type and WT-rescue cells was analyzed by northern blot using a radio-labeled probe that was generated from wild-type genomic DNA amplified by PCR with the primers 5Am-Pdd1_Fw and SpeI-PDD1ORF_Rv2.

Recombinant proteins

Full-length wild-type *PDD1*, *MIM14*, *MIM22* and *MIM22+Ins6K* genes that were codon-optimized for *E. coli* expression and named *opt.WT_FL*, *opt.MIM14_FL*, *opt.MIM22_FL* and *opt.MIM22+Ins6K_FL,* respectively,

were synthesized (Genscript and IDT). The sequences are listed below. Amplified DNA corresponding to the *PDD1* mutants *opt.W50A/W53A_FL* and *opt.I456D_FL* were produced from *opt.WT_FL* by PCR. For *opt.I456D_FL*, the primer set EcoRI-opt.PDD1_Fw/PstI-TGA-opt.PDD1_I478D_RvMAL was used (primer sequences are listed below). For *opt.W50A/W53A_FL*, two DNA fragments were first amplified from *opt.WT-FL* with the primer sets EcoRI-opt.PDD1_Fw/opt.PDD1_W72,75A_Rv1 and opt.PDD1_W72,75A_Fw1/PstI-TGA-opt.PDD1_Rv2. These fragments were connected by overlapping PCR with the primer set EcoRI-opt.PDD1_Fw/PstI-TGA-opt.PDD1_Rv2. Amplified DNA corresponding to the hinge regions of wild-type Pdd1p, *opt.WT_HNG1*: 78-188 aa and *opt.WT_HNG2*: 226-397 aa, were produced from *opt.WT_FL* by PCR with the primer sets ERI-opt.PDD1_HNG1_Fw/PstI-TGA-opt.PDD1_HING1_Rv and ERI-opt.PDD1_HNG2_Fw/PstI-TGA-opt.PDD1_HING2_Rv. For the MBP-fusion proteins, *opt.WT_FL*, *opt.WT_HNG1*, *opt.WT_HNG2*, *opt.MIM14_FL*, *opt.MIM22_FL*, *opt.MIM22+Ins6K_FL*, *opt.W50A/W53A_FL* and *opt.I456D_FL* were cloned into the EcoRI and PstI sites of pMAL-c2X (NEB). To express MBP alone, a stop codon-containing sequence (5'-TGAGCATAACGTGCCTTGC-3') was inserted into the EcoRI and PstI sites of pMAL-c2X. For the GST-fusion proteins, *opt.WT_FL* and *opt.MIM22_FL* were amplified by PCR with the primer sets EcoRI-opt.PDD1_FwpGEX/XhoI-opt.PDD1_RvpGEX and ERI-opt.MIM22_FwGEX/XhoI-opt.MIM22_RvGEX, respectively, and cloned into the EcoRI and XhoI sites of a variant of pGEX-4T-1 containing a TEV cleavable site between an N-terminal GST tag and the protein coding sequence (pGEX-4T-1-TEV, a gift from Dr. Tim Clausen, IMP, Austria). For GST alone, empty pGEX-4T-1-TEV was used. All recombinant proteins were expressed in the *E. coli* strain BL21 (DE3). Bacterial cultures were grown at 37°C in LB with ampicillin to an OD600 of ~0.8. The cultures were cooled on ice for 20 min, and protein expression was induced with 0.5 mM IPTG at 18°C overnight. The recombinant proteins were purified as described previously (Woehrer et al., 2015), dialyzed against Interaction buffer (20 mM HEPES-NaOH pH 7.5, 100 mM KCl, 1 mM EDTA, 0.1 mM DTT, 10% glycerol) at 4°C overnight, and stored at -80°C. The protein concentrations were estimated by SDS-PAGE followed by PageBlue staining (Thermo Scientific) using BSA (NEB) as a reference.

Histone peptide pull-down assay

Biotinylated Peptides corresponding to the N-terminal tail of *Tetrahymena* histone H3 (K9unmod: ARTKQTARKSTGAKAPRKQ, K9me3: ARTKQTAR[Kme3]STGAKAPRKQ, K27unmod: PRKQLASKAARKSAPATGG and K27me3: PRKQLASKAAR[Kme3]SAPATGG) were synthesized as described (Vogt and Mochizuki, 2013). Peptide (4 nmol) was incubated with 20 µl (bed volume) of Dynabeads M-280 Streptavidin (Invitrogen) in PBS at room temperature for 3 h and excess peptide was washed-out with PBS containing 0.1% Tween-20. The peptide-coupled beads were pre-incubated with Interaction buffer (20 mM HEPES-NaOH pH 7.5, 1 mM EDTA, 0.1 mM DTT, 10% glycerol, and KCl at 400 mM for K27 peptides or 500 mM for K9 peptides) containing 5% BSA at 4°C for 1 h. Recombinantly expressed MBP-WT_FL or MBP-MIM22_FL (30 pmol) was incubated with the beads in 300 µl Interaction buffer (with corresponding KCl concentration) containing 2.5% BSA at 4°C overnight. The beads were washed with Washing buffer (20 mM HEPES-NaOH pH 7.5, 1 mM EDTA, 0.1% Tween-20, and KCl at 400 mM for K27 peptides or 500 mM for K9 peptides) at room temperature 6 times for 10 min each. The bound protein was eluted with 1x SDS-PAGE sample buffer by incubation at 95°C for 10 min, resolved in 8% SDS-PAGE gel and analyzed by western blot using anti-MBP primary antibody (NEB) and anti-rabbit IgG secondary antibody conjugated with IRDye 800CW (LI-COR). The images were quantified by Odyssey CLx (LI-COR). The mean value of enrichment relative to corresponding input and the standard deviation of the mean were calculated from 3 independent experiments.

GST pull-down assays

GST and MBP fusion proteins (350 pmol each) were mixed, and the volume was adjusted to 400 µl with Interaction buffer (20 mM HEPES-NaOH pH 7.5, 100 mM KCl, 1 mM EDTA, 0.1 mM DTT, 10% glycerol). GST pull-down buffer (933.3 µl) (20 mM Tris pH 7.5, 100 mM NaCl, 0.1 mM EDTA, 0.1% Triton X-100) was added, and the proteins were incubated at 4°C for 1 h. The protein complex containing the GST-tag was affinity purified by incubation with a 20 µl suspension of Glutathione Sepharose 4B beads (GE-Healthcare) at 4°C for 1 h followed by four washes with GST pull-down buffer at 4°C. The protein was eluted from the beads by incubation with 25 µl of 1x SDS PAGE buffer at 95°C for 10 min ("GST pull-down" sample). Then, 17.5 pmol (1/20) of the input samples and 10 µl of the GST pull-down samples were analyzed by SDS-PAGE followed by PageBlue staining (Thermo Scientific).

EMSA

To produce EGFP ssRNA (723 nt) and Cal IES ssRNA (1305 nt) as substrates for EMSA, DNA templates containing the T7 promoter were generated from the plasmids pEGFP-neo4 (Kataoka et al., 2010) and pCaM_MDS-IES, which contains the Cal IES sequence from the B2086 wild-type strain (see below for the sequence), by PCR using PrimeSTAR HS DNA polymerase and the primer sets T7-opt.EGFP_Fw1/opt.EGFP_Rv1 and T7-CaM-IES_Fw1/CaM-IES_Rv1, respectively (primer sequences are listed below). The PCR products were purified with the QIAquick PCR Purification Kit (Qiagen) and Illustra MicroSpin S-400 HR Columns (GE Healthcare). In vitro transcription was performed on the PCR products using the

MEGAscript T7 transcription kit (Ambion) according to the manufacturer's instructions. To label the RNA, 1.9 mM fluorescein-12 UTP (Roche) and 5.6 mM non-labeled UTP were used in the reaction. The reaction (20 µl) was carried out at 37°C for 3 h followed by incubation with 1 µl of TURBO DNase (Ambion) at 37°C for 15 min. The labeled RNAs were purified by phenol-chloroform-isoamyl alcohol extraction followed by isopropanol precipitation. The RNAs were dissolved in nuclease-free water, and the unincorporated nucleotides were removed using mini Quick Spin RNA columns (Roche). EMSAs were performed as described previously (Keller et al., 2012) with modifications. Two microliters containing the desired amount (0.125-7.5 pmol) of protein dialyzed against Interaction buffer was incubated with 8 µl of EMSA buffer (20 mM HEPES-NaOH pH7.5, 100 mM KCl, 0.05% NP-40) at room temperature for 10 min. Two microliters of fluorescein-labeled *EGFP* ssRNA or Cal IES ssRNA (125 fmol) was added as a substrate and the mixture was incubated at room temperature for 30 min, and the samples were analyzed by 1x TBE agarose (0.5% for the FL and 1% for the HNG1 and HNG2 proteins) gel electrophoresis. Fluorescently labeled RNA was detected using a Typhoon Trio imaging system (GE Healthcare) and quantified with ImageQuant TL (GE Healthcare). The mean dissociation constant (*K*d) and the standard deviation of the mean were calculated from more than 2 independent experiments.

Primers used for C-terminal EGFP tagging constructs

a) Primers to amplify coding and 3’ flanking regions

TTHERM_#	amplicon	name	sequence (5' -> 3')
TTHERM_00006160	coding	5Am-00006160_Fw	GCTGATGGCGATGAATGAACACTGGCTATCAAATATCGATAATATTGATGTTTC
		00006160-EGFP_Rv	AAGTTCTTCACCCTTAGAAACCATGGATCCGTTAAATATATCGTTGACAGTAAGGTTAGC
	3' flanking	Neo4-00006160_Fw	CCCGGGGGATCTGAATTCGATATCAAGCTTGAAGAAGTTACTCAAGTATGTATGAACTTG
		3Am-00006160_Rv	GCGAGCACAGAATTAATACGACTAAATAAAACTTTAATATTGTACTAAGTTAC
TTHERM_00008690	coding	5Am-00008690_Fw	GCTGATGGCGATGAATGAACACTGATGAATAACATAGAAGAAAGTGCTTTCTAC
		00008690-EGFP_Rv	AAGTTCTTCACCCTTAGAAACCATGGATCCGTTTTTTTAATTCTTTTTACTTGATTATTAAATG
	3' flanking	Neo4-00008690_Fw	CCCGGGGGATCTGAATTCGATATCAAGCTTATATAATATACCACATTTTATACTTAGTCC
		3Am-00008690_Rv	GCGAGCACAGAATTAATACGACTTCTAAGATCATCATTTGCTGCCTTTCCT
TTHERM_00013110	coding	5Am-00013110_Fw	GCTGATGGCGATGAATGAACACTGATGTTGATAGTGATGGTATGTATATTGTCA
		00013110-EGFP_Rv	AAGTTCTTCACCCTTAGAAACCATGGATCCAAGTTCTTGAAGTACCTGTTAATTAATTAAT
	3' flanking	Neo4-00013110_Fw	CCCGGGGGATCTGAATTCGATATCAAGCTTCATAACCACAAACAAACACATAAAAAGCCA
		3Am-00013110_Rv	GCGAGCACAGAATTAATACGACTACAATTAAAGTTTGGACATCAATCAAAGCT
TTHERM_000245499	coding	5Am-00245500_Fw	GCTGATGGCGATGAATGAACACTGCAAGCTATGTATTCCTTAGCGTGCCAGCAC
		00245500-EGFP_Rv	AAGTTCTTCACCCTTAGAAACCATGGATCCATCTTAAATTCCTCTTTAAAATTATTTAAA
	3' flanking	Neo4-00245500_Fw	CCCGGGGGATCTGAATTCGATATCAAGCTTCTAAAATAGCTTTAAAGTACACAGTAGAATC
		3Am-00245500_Rv	GCGAGCACAGAATTAATACGACTTTCTAGAATTATTAATTCAAAATGTATGTG
TTHERM_000279929	coding	5Am-00279930_Fw	GCTGATGGCGATGAATGAACACTGGTAATGAGATGAGTTTAGATGAAGCATCAG
		00279930-EGFP_Rv	AAGTTCTTCACCCTTAGAAACCATGGATCCTTTTTTTATTTTTTTGGTATACTTTTATTTATATTC
	3' flanking	Neo4-00279930_Fw	CCCGGGGGATCTGAATTCGATATCAAGCTTTAAATCAAATGTTTGTTTCAACTACAATTC
		3Am-00279930_Rv	GCGAGCACAGAATTAATACGACTTATGTCTTATATTCTCACTAAATTATCATC
TTHERM_00028580	coding	5Am-00028580_Fw	GCTGATGGCGATGAATGAACACTGAATAATGTAGGACTTATCCTTGACATCTCT
		00028580-EGFP_Rv	AAGTTCTTCACCCTTAGAAACCATGGATCCATTATTAGCATAATAAGAAGGAGAGTTTATG
	3' flanking	Neo4-00028580_Fw	CCCGGGGGATCTGAATTCGATATCAAGCTTGATAAAATCTAAATATTCAGCCACCATAATG
		3Am-00028580_Rv	GCGAGCACAGAATTAATACGACTATCCTCTCTATCTTATTAGATAAAGAAGAG
TTHERM_000295779	coding	5Am-00295780_Fw	GCTGATGGCGATGAATGAACACTGGTAATAGGGAAATCAATATTTGCTTTAAAG
		00295780-EGFP_Rv	AAGTTCTTCACCCTTAGAAACCATGGATCCGCAATAAGACTTTTTTTTTTGAAAATTTATTG
	3' flanking	Neo4-00295780_Fw	CCCGGGGGATCTGAATTCGATATCAAGCTTTATAGATAGATATATATGTGCATACTTTAC
		3Am-00295780_Rv	GCGAGCACAGAATTAATACGACTTTCTATATTCCAACTCTCTTTGGACATATC
TTHERM_00034970	coding	5Am-00034970_Fw	GCTGATGGCGATGAATGAACACTGTACATGAACTTAAAGAATGCACTTGACGAC
		00034970-EGFP_Rv	AAGTTCTTCACCCTTAGAAACCATGGATCCCTTTTCTTTTTAGTTATTGAGTGATTTTTG
	3' flanking	Neo4-00034970_Fw	CCCGGGGGATCTGAATTCGATATCAAGCTTTGCTTATTGGATAAAATATTAATCCATAGC
		3Am-00034970_Rv	GCGAGCACAGAATTAATACGACTATAATTAGCTTGTCCAATTTCTTTACAAAC
TTHERM_00036890	coding	5Am-00036890_Fw	GCTGATGGCGATGAATGAACACTGCTCGAACTCTAATCAGATTCATTTAGTGTG
		00036890-EGFP_Rv	AAGTTCTTCACCCTTAGAAACCATGGATCCTTCTAATTCTATTTAATTAATCTAATTTTC
	3' flanking	Neo4-00036890_Fw	CCCGGGGGATCTGAATTCGATATCAAGCTTCCACTTTACAAACATTCATATTTAGCTTTC
		3Am-00036890_Rv	GCGAGCACAGAATTAATACGACTTCAAAACTTATAAAATCCAGCTGAAATATC
TTHERM_00046930	coding	5'Am-00046930_Fw	GCTGATGGCGATGAATGAACACTGACAGACAACAGTTGTGCTGAAAAAGCTGTTT
		00046930-EGFP_Rv2	AAGTTCTTCACCCTTAGAAACCATGGATCCTAAATTTTAGGAATAATTCTAATTCAAAGA
	3' flanking	Neo4-00046930_3'FLNK_Fw3	CCCGGGGGATCTGAATTCGATATCAAGCTTTGGTTATATCATTGCATTTTATCTTTCTAA
		3'Am-00046930_Rv3	GCGAGCACAGAATTAATACGACTTGATATGGGAGTGGGCACCTATTCTTTCGT
TTHERM_00049220	coding	5Am-00049220_Fw	GCTGATGGCGATGAATGAACACTGTGAAATATCAGTGAGGAGAATGAGCAACAC
		00049220-EGFP_Rv	AAGTTCTTCACCCTTAGAAACCATGGATCCTTTGCTTAATTTTGGATTGTATTAGTCAAT
	3' flanking	Neo4-00049220_Fw	CCCGGGGGATCTGAATTCGATATCAAGCTTTTAAAATATATAGAATGGATTGATTACGAG
		3Am-00049220_Rv	GCGAGCACAGAATTAATACGACTTACAAGTCTCCAACAACTCACTATGGCTGA
TTHERM_000549648	coding	5Am-00549670_Fw	GCTGATGGCGATGAATGAACACTGAGCATTTGTTGTTGTTGCTGCTGCTAGTTC
		00549670-EGFP_Rv	AAGTTCTTCACCCTTAGAAACCATGGATCCATTAATTAATTATTAAATATACTGG
	3' flanking	Neo4-00549670_Fw	CCCGGGGGATCTGAATTCGATATCAAGCTTTTCAGTTTAATTTACATTCTTTTTAAGTTG
		3Am-00549670_Rv	GCGAGCACAGAATTAATACGACTTTTACAAGTTATGGAAACAAATAGATGCTC
TTHERM_00079530	coding	5'Am-00079530_Fw	GCTGATGGCGATGAATGAACACTGCACTTCTGTCGTGAAATTGCTCATCAAGCT
		00079530-EGFP_Rv	AAGTTCTTCACCCTTAGAAACCATGGATCCATTCAAGATAAACTTTTAGAATTTATCTCT
	3' flanking	Neo4-00079530_3'FLNK_Fw	CCCGGGGGATCTGAATTCGATATCAAGCTTCTATATAAATTGTGATTGACTTTTAATTAG
		3'Am-00079530_Rv	GCGAGCACAGAATTAATACGACTCATCCATTCCTATAAAGCTGATGAAGTGCT
TTHERM_00086720	coding	5'Am-00086720_Fw	GCTGATGGCGATGAATGAACACTGGTCAACTTCAATTTAGAGATCCAGTTCTGG
		00086720-EGFP_Rv	AAGTTCTTCACCCTTAGAAACCATGGATCCGATATTTATTCTATTTTCTTTCCCGTTTACC
	3' flanking	Neo4-00086720_3'FLNK_Fw	CCCGGGGGATCTGAATTCGATATCAAGCTTCTCTTCATTCTATATATAAATAAAATCAACT
		3'Am-00086720_Rv	GCGAGCACAGAATTAATACGACTAAAAAATCTATTAGAATATACTATCAGCCT
TTHERM_00091510	coding	5Am-00091510_Fw	GCTGATGGCGATGAATGAACACTGGTAAAAGTACTATGACGACTTGCTACCTGT
		00091510-EGFP_Rv	AAGTTCTTCACCCTTAGAAACCATGGATCCTTATTTAGGAGCCATAATGTTTAAGAGC
	3' flanking	Neo4-00091510_Fw	CCCGGGGGATCTGAATTCGATATCAAGCTTTGTTAATTAAATTTAGTAGGTTTTAACTGT
		3Am-00091510_Rv	GCGAGCACAGAATTAATACGACTGTGTATTTAGCTATAAGTATTAGCGTTGA
TTHERM_00092800	coding	5Am-00092800_Fw	GCTGATGGCGATGAATGAACACTGAACAACTGGTACCAACAGGGAAAGTGTATC
		00092800-EGFP_Rv	AAGTTCTTCACCCTTAGAAACCATGGATCCAAGTATTTGGCTATTTAGTTTACTC
	3' flanking	Neo4-00092800_Fw	CCCGGGGGATCTGAATTCGATATCAAGCTTTTCAAATACTACTACTATTACAAATACTAG
		3Am-00092800_Rv	GCGAGCACAGAATTAATACGACTAGTTTAGAAGTGTTTGTTTTAATTACTTAG
TTHERM_00112710	coding	5Am-00112710_Fw	GCTGATGGCGATGAATGAACACTGAGCAGTAGGAAGAAGATAAGGTGATTGGCT
		00112710-EGFP_Rv	AAGTTCTTCACCCTTAGAAACCATGGATCCATTTTATTCAATATTATTTACTTAGATTTTTTGAG
	3' flanking	Neo4-00112710_Fw	CCCGGGGGATCTGAATTCGATATCAAGCTTCAAATAGTTTTGTTCTAATAGAGACAATAG
		3Am-00112710_Rv	GCGAGCACAGAATTAATACGACTCTATAAATATTTTCATTCTATCAACACCTA
TTHERM_00112830	coding	5Am-00112830_Fw	GCTGATGGCGATGAATGAACACTGTGAAAATAACTCATTGTACTAATACTAGGA
		00112830-EGFP_Rv	AAGTTCTTCACCCTTAGAAACCATGGATCCAATTAAAATGACATAGTATTCATC
	3' flanking	Neo4-00112830_Fw	CCCGGGGGATCTGAATTCGATATCAAGCTTTTTTGAAAAATTATGAAATTATATAAAGGC
		3Am-00112830_Rv	GCGAGCACAGAATTAATACGACTGGAATAACATGGATAAAAGTATTACCATGG
TTHERM_00113310	coding	5Am-00113310_Fw	GCTGATGGCGATGAATGAACACTGCAGACTCAGAATACATGATTTAGGCTATTG
		00113310-EGFP_Rv	AAGTTCTTCACCCTTAGAAACCATGGATCCATTATTATCTATAAGTTCTTTGAATTTCTG

	3' flanking	Neo4-00113310_Fw	CCCGGGGGATCTGAATTCGATATCAAGCTTACGAATTAATTTCTCCTAGTTTTTTCTTAC
		3Am-00113310_Rv	GCGAGCACAGAATTAATACGACTATTTCATAAAAAATGTTAGCTGTAGTAATG
TTHERM_00128920	coding	5Am-00128920_Fw	GCTGATGGCGATGAATGAACACTGGGTAAGAAAAGCTAATAAGCTCAAGGTGAC
		00128920-EGFP_Rv	AAGTTCTTCACCCTTAGAAACCATGGATCCATCTTGAGTTTAACTATGTTCATGTTCAGA
	3' flanking	Neo4-00128920_Fw	CCCGGGGGATCTGAATTCGATATCAAGCTTTTCTTTAAAATCTAAAGAAAAGTATCTTGC
		3Am-00128920_Rv	GCGAGCACAGAATTAATACGACTCAAAACATTTAATAAATGTCTCAAGATGCC
TTHERM_00133710	coding	5Am-00133710_Fw	GCTGATGGCGATGAATGAACACTGGTTAACATACAAGACACTCCAATCTTTCTC
		00133710-EGFP_Rv	AAGTTCTTCACCCTTAGAAACCATGGATCCAAAAGCACTCTTTCTGAAGCTAAAACAACT
	3' flanking	Neo4-00133710_Fw	CCCGGGGGATCTGAATTCGATATCAAGCTTATAAAGAACTTACTTAATTATCAGGTTAAC
		3Am-00133710_Rv	GCGAGCACAGAATTAATACGACTGTTTAAAGTAACCTTCATCCATCATACCAA
TTHERM_00133730	coding	5Am-00133730_Fw2	GCTGATGGCGATGAATGAACACTGAACCTAAAGTAATCACAATTGTTTCTTCC
		00133730-EGFP_Rv2	AAGTTCTTCACCCTTAGAAACCATGGATCCATTATTTTAAGTTTAGGTTGCCTTTTTCC
	3' flanking	Neo4-00133730_Fw2	CCCGGGGGATCTGAATTCGATATCAAGCTTTTTTAGTTATATTTTTGTGGATTGTAC
		3Am-00133730_Rv2	GCGAGCACAGAATTAATACGACTCAAATAGAATATGATGATATCAAATTACTG
TTHERM_00155590	coding	5'Am-00155590_Fw	GCTGATGGCGATGAATGAACACTGGAAGAAGCTCTAAGTAAATGCATGCAGAAAC
		00155590-EGFP_Rv	AAGTTCTTCACCCTTAGAAACCATGGATCCTTGTTCAGCATTCATTTATTGTAAGCCATT
	3' flanking	Neo4-00155590_3'FLNK_Fw	CCCGGGGGATCTGAATTCGATATCAAGCTTACCTATCTATATTTTTCCACTAATTACCCA
		3'Am-00155590_Rv	GCGAGCACAGAATTAATACGACTGTATTCTAATAACTATCCTATCAGTCAGTC
TTHERM_00185640	coding	5Am-00185640_Fw2	GCTGATGGCGATGAATGAACACTGTTCACTCACTCAATCTTCGAGAGTTCTAAG
		00185640-EGFP_Rv	AAGTTCTTCACCCTTAGAAACCATGGATCCGTTTGAGCTATTAGTAATTTATTTTAAGTT
	3' flanking	Neo4-00185640_Fw	CCCGGGGGATCTGAATTCGATATCAAGCTTAAACGAACTTTTTACTTCTAACAAACCAAC
		3Am-00185640_Rv	GCGAGCACAGAATTAATACGACTACCTTTTGGAATTGAAAGCTCAAATTCTGC
TTHERM_00189440	coding	5'Am-00189440_Fw	GCTGATGGCGATGAATGAACACTGCTGCATCTCCATTTGATTATGTAGATAATC
		00189440-EGFP_Rv	AAGTTCTTCACCCTTAGAAACCATGGATCCAAATGATTATTTGCTATTATTTGCAGAATG
	3' flanking	Neo4-00189440_3'FLNK_Fw	CCCGGGGGATCTGAATTCGATATCAAGCTTTTGTATTTATTTGTGTGTATGTGTGTTTGA
		3'Am-00189440_Rv	GCGAGCACAGAATTAATACGACTAGTTATTTGAGGGTGGTTACCATCAAATGC
TTHERM_00193969	coding	5Am-00193970_Fw	GCTGATGGCGATGAATGAACACTGGTTTAACATTCCTTCGTTTCTACTAAACCT
		00193970-EGFP_Rv	AAGTTCTTCACCCTTAGAAACCATGGATCCAATTTCCTAATCTAAGTCTTTCACTTCTAAG
	3' flanking	Neo4-00193970_Fw	CCCGGGGGATCTGAATTCGATATCAAGCTTTGTTTGTTATCTTTTATAAAAGTATTCTTG
		3Am-00193970_Rv	GCGAGCACAGAATTAATACGACTAAGCCAATTTAAATTGTCCATAGTTGGTCT
TTHERM_00197670	coding	5Am-00197670-2_Fw	GCTGATGGCGATGAATGAACACTGAATTAACCAAGAAGACCAATCATCCTTGCT
		00197670-2-EGFP_Rv	AAGTTCTTCACCCTTAGAAACCATGGATCCTTTGTAAACCTTCTTTTCTTATTATTTAAG
	3' flanking	Neo4-00197670-2_Fw	CCCGGGGGATCTGAATTCGATATCAAGCTTGTTGGATTTTTGATTGAATTTTAGAAAATC
		3Am-00197670-2_Rv	GCGAGCACAGAATTAATACGACTAAAGTTAGTTTCTGACTAATTATTAGTAGT
TTHERM_00204150	coding	5Am-00204150_Fw	GCTGATGGCGATGAATGAACACTGTGATTGATATAAAGATATATAGAAAATGGA
		00204150-EGFP_Rv	AAGTTCTTCACCCTTAGAAACCATGGATCCTTAGATAAATAATTTTTATGGAACATTATC
	3' flanking	Neo4-00204150_Fw	CCCGGGGGATCTGAATTCGATATCAAGCTTTTCTATATTGGAATCTGCTACAATTCTAAG
		3Am-00204150_Rv	GCGAGCACAGAATTAATACGACTATTAATCTAATTCAAAAATTAGACCTATTC
TTHERM_00219320	coding	5'Am-00219320_Fw	GCTGATGGCGATGAATGAACACTGCGATAAATTAGCAAAAGATTAATTTGAGCA
		00219320-EGFP_Rv2	AAGTTCTTCACCCTTAGAAACCATGGATCCATAAATTATATGATTATTAAATTTTCTTTT
	3' flanking	Neo4-00219320__Fw2	CCCGGGGGATCTGAATTCGATATCAAGCTTACAAAATATTCATCGATAATCAGAAACTTC
		3'Am-00219320_Rv2	GCGAGCACAGAATTAATACGACTAAGTTGATCCTGAGACAAATTATCGAAGCT
TTHERM_00221110	coding	5Am-00221110_Fw	GCTGATGGCGATGAATGAACACTGCAACTAGATGACCTCTTAAATGAAAAATCC
		00221110-EGFP_Rv	AAGTTCTTCACCCTTAGAAACCATGGATCCTTTGCTTGGTTCGCTGCCGTCCTATTAATTTG
	3' flanking	Neo4-00221110_Fw	CCCGGGGGATCTGAATTCGATATCAAGCTTTGTTTCTTGGCAAAACAATATTTTCTATTG
		3Am-00221110_Rv	GCGAGCACAGAATTAATACGACTTCACAAAAACGAAATATATCTGGTTTATAG
TTHERM_00237610	coding	5Am-00237610_Fw	GCTGATGGCGATGAATGAACACTGCAACAGAAAAAATGATGGAAAAGCTGACTG
		00237610-EGFP_Rv	AAGTTCTTCACCCTTAGAAACCATGGATCCAAGAAGCTTCTCAATTCTTGATTTAAATCT
	3' flanking	Neo4-00237610_Fw	CCCGGGGGATCTGAATTCGATATCAAGCTTTCTATTAAAGCTTATTTTATATTTCGATGA
		3Am-00237610_Rv	GCGAGCACAGAATTAATACGACTTCATCTGTTTATAATCGAAGTTAATTTACT
TTHERM_00267890	coding	5Am-00267890_Fw	GCTGATGGCGATGAATGAACACTGGTCACTCAAACATCTCAAGGCTAATTTGCT
		00267890-EGFP_Rv	AAGTTCTTCACCCTTAGAAACCATGGATCCTTATGGATTTCCTTTATAATTAGT
	3' flanking	Neo4-00267890_Fw	CCCGGGGGATCTGAATTCGATATCAAGCTTAAGTATCTATTTACCTATCAATCAATCTCT
		3Am-00267890_Rv	GCGAGCACAGAATTAATACGACTCATGCTTGATAATTGATTATTAACAATCTC
TTHERM_00289290	coding	5Am-00289290_Fw	GCTGATGGCGATGAATGAACACTGCTTGACATCAGATGTAGCTCATGAGCTCAG
		00289290-EGFP_Rv	AAGTTCTTCACCCTTAGAAACCATGGATCCATTATTAAAGTAGCTTTTAGTTGCGTCTTAG
	3' flanking	Neo4-00289290_Fw	CCCGGGGGATCTGAATTCGATATCAAGCTTCGAGATAAATAAATATCTATGAACTCAAAG
		3Am-00289290_Rv	GCGAGCACAGAATTAATACGACTTGGTTTATTCTTCATATTTATGTGTAAGAG
TTHERM_00299879	coding	5Am-00299870_Fw	GCTGATGGCGATGAATGAACACTGGAAAAGTAGTCAATTCTTTGCGAACCCTGT
		00299870-EGFP_Rv	AAGTTCTTCACCCTTAGAAACCATGGATCCTTCATAGGCAGGTATTTTTTGGCAAAAAGA
	3' flanking	Neo4-00299870_Fw	CCCGGGGGATCTGAATTCGATATCAAGCTTGCTTATCCAGAAATTCATCCAAGTTTTTAG
		3Am-00299870_Rv	GCGAGCACAGAATTAATACGACTGAAGGAGTTGTCATATTAGATGAAGCTTAC
TTHERM_00301910	coding	5Am-00301910_Fw	GCTGATGGCGATGAATGAACACTGATGATAGAAAATTTTGACAAATTAATTTAG
		00301910-EGFP_Rv	AAGTTCTTCACCCTTAGAAACCATGGATCCTTCGTCAAATTCTTCTTCATCTTTTTC
	3' flanking	Neo4-00301910_Fw	CCCGGGGGATCTGAATTCGATATCAAGCTTTTTTGAGATGTGATGTATTCTTTCATGGGA
		3Am-00301910_Rv	GCGAGCACAGAATTAATACGACTACAAATATAGAGATTTATAGAAAGATCAAG
TTHERM_00309960*	coding	5Am-00309960_Fw2	GCTGATGGCGATGAATGAACACTGAGTTGACACCTAAAATAAGTAATTAAGGTG
		00309960-EGFP_Rv3	AAGTTCTTCACCCTTAGAAACCATGGATCCTATAATTAAGGTTGAAATGAATTATTAATC
	3' flanking	Neo4-00309960_Fw2	CCCGGGGGATCTGAATTCGATATCAAGCTTTGTTATTCATTTTGTAAGTTTTATTTACTC
		3Am-00309960_Rv2	GCGAGCACAGAATTAATACGACTTTTGCACACTTTTTTGATACATAAATTCTC
TTHERM_00341280	coding	5Am-00341280_Fw2	GCTGATGGCGATGAATGAACACTGGCAAGTTATACAAAACTCAGTTTATCC
		00341280-EGFP_Rv2	AAGTTCTTCACCCTTAGAAACCATGGATCCAGATTATTATGATTAGTAAAAACTGTTGC
	3' flanking	Neo4-00341280_Fw2	CCCGGGGGATCTGAATTCGATATCAAGCTTACTAAATGATGCAAAATAAAATTGTCTTC
		3Am-00341280_Rv2	GCGAGCACAGAATTAATACGACTAATCTAAAACATTTAACAACAGTATCTAG
TTHERM_00370840	coding	5Am-00370840_Fw	GCTGATGGCGATGAATGAACACTGTTATTTGATTGAAACATTTCCTTCTCTTGT
		00370840-EGFP_Rv	AAGTTCTTCACCCTTAGAAACCATGGATCCGTATTCATTAACAGCAAAAAATTCCTGT
	3' flanking	Neo4-00370840_Fw	CCCGGGGGATCTGAATTCGATATCAAGCTTCAGTAAATTATTGATTTCAGTGATATTAAG
		3Am-00370840_Rv	GCGAGCACAGAATTAATACGACTTTACCCTTATAATATAGATTTCAGTTGTTC
TTHERM_00402050	coding	5'Am-00402050_Fw	GCTGATGGCGATGAATGAACACTGTAGTTAAATAGCTCAGAAATCAGAAGAGTT
		00402050-EGFP_Rv	AAGTTCTTCACCCTTAGAAACCATGGATCCTTAAAACAATTTTCTATAAAATGTATTTATTGATTG
	3' flanking	Neo4-00402050_3'FLNK_Fw	CCCGGGGGATCTGAATTCGATATCAAGCTTCTTTATAATAAACTTACAACACAACTTCTA

		3'Am-00402050_Rv	GCGAGCACAGAATTAATACGACTCTTCAGTGTGTTAGACTGACGTGATAACCA
TTHERM_00412010a*	coding	5Am-00412010_Fw2	GCTGATGGCGATGAATGAACACTGGAATAAAAGTCTTTTGTTGCTTGAGGCAACG
		00412010-EGFP_Rv	AAGTTCTTCACCCTTAGAAACCATGGATCCTTAAAAAAAGCATGATTATGGTGGGGATAA
	3' flanking	Neo4-00412010_Fw	CCCGGGGGATCTGAATTCGATATCAAGCTTTATTATATTATTTTTCAATATCAGCCTGTA
		3Am-00412010_Rv	GCGAGCACAGAATTAATACGACTATATATGAAAATAGATTACGTTAGAGCCTA
TTHERM_00412010b*	coding	5Am-00412010_Fw2	GCTGATGGCGATGAATGAACACTGGAATAAAAGTCTTTTGTTGCTTGAGGCAACG
		00412010-EGFP_Rv-2	AAGTTCTTCACCCTTAGAAACCATGGATCCTATTTGATATAATAAAAAGCTGACATAGA
	3' flanking	Neo4-00412010_Fw	CCCGGGGGATCTGAATTCGATATCAAGCTTTATTATATTATTTTTCAATATCAGCCTGTA
		3Am-00412010_Rv	GCGAGCACAGAATTAATACGACTATATATGAAAATAGATTACGTTAGAGCCTA
TTHERM_00420400*	coding	5Am-00420400_Fw2	GCTGATGGCGATGAATGAACACTGAGAACTTAACTCTGAGGTTACTATGCTAAG
		00420400-EGFP_Rv_2	AAGTTCTTCACCCTTAGAAACCATGGATCCAAATTTCTTCTTTTTCTCTGTGGTGGAATA
	3' flanking	Neo4-00420400_Fw	CCCGGGGGATCTGAATTCGATATCAAGCTTATGTTACAAATTTTTATACAGTTATTTAGC
		3Am-00420400_Rv	GCGAGCACAGAATTAATACGACTAGTTACTGTAATATTAGTTCTAGAGGGAGA
TTHERM_00433790	coding	5Am-00433790_Fw	GCTGATGGCGATGAATGAACACTGTTTGAGTGCTGTAAATATCGATCCTAACCA
		00433790-EGFP_Rv	AAGTTCTTCACCCTTAGAAACCATGGATCCTTTATTTTATTTATATTATGACAAAAATCC
	3' flanking	Neo4-00433790_Fw	CCCGGGGGATCTGAATTCGATATCAAGCTTTTTTAATCATACATGAACACAGAAACAAATTTTAC
		3Am-00433790_Rv	GCGAGCACAGAATTAATACGACTATTGATCCATTGAAAAATAAATAGGAATCG
TTHERM_00439300	coding	5Am-00439300_Fw	GCTGATGGCGATGAATGAACACTGGATAGAGGAAAAATAAGTGAGTGAGGATTC
		00439300-EGFP_Rv	AAGTTCTTCACCCTTAGAAACCATGGATCCATCTCCGTATTCGTACTGATCCTCATCATC
	3' flanking	Neo4-00439300_Fw	CCCGGGGGATCTGAATTCGATATCAAGCTTTAGTTTCTACTTTTAAAGTTATATTTCTTG
		3Am-00439300_Rv	GCGAGCACAGAATTAATACGACTGTTTTATATTTAATGTAAGCTCACTTAGCTG
TTHERM_00442420	coding	5Am-00442420_Fw	GCTGATGGCGATGAATGAACACTGTGGAACAATTTAAGTTTGGCAGCTATCAGA
		00442420-EGFP_Rv	AAGTTCTTCACCCTTAGAAACCATGGATCCCTTATTAGGTATAAATGAGAATTTAATTAG
	3' flanking	Neo4-00442420_Fw	CCCGGGGGATCTGAATTCGATATCAAGCTTTTTTCAGTCCGCTTCATATATCAGTGTATC
		3Am-00442420_Rv	GCGAGCACAGAATTAATACGACTGCATTAAATAGATTAGGTAGCTTGCTTATC
TTHERM_00460720	coding	5Am-00460720_Fw	GCTGATGGCGATGAATGAACACTGTGATAATGAAAAGTAATCTCAGAGCAGC
		00460720-EGFP_Rv	AAGTTCTTCACCCTTAGAAACCATGGATCCAATTATTTTTTTAATAATTTATGTAATATC
	3' flanking	Neo4-00460720_Fw	CCCGGGGGATCTGAATTCGATATCAAGCTTTACTGAGTTTGATTTTTCTTAGCTGTTAG
		3Am-00460720_Rv	GCGAGCACAGAATTAATACGACTTTAGCCTTACTCCAAACTTTAACTTGTG
TTHERM_00471000	coding	5Am-00471000_Fw	GCTGATGGCGATGAATGAACACTGGTACTTCATAAATTAGGGTATTTTATAGTC
		00471000-EGFP_Rv	AAGTTCTTCACCCTTAGAAACCATGGATCCCATTTATTTTGATAAAATATTTTTATTC
	3' flanking	Neo4-00471000_Fw	CCCGGGGGATCTGAATTCGATATCAAGCTTTACATGTTTATTTGTTTTATTGGTTGGTTG
		3Am-00471000_Rv	GCGAGCACAGAATTAATACGACTGGCTTGAAGGAGATGACTCATTCAATGCTG
TTHERM_00471720	coding	5Am-00471720_Fw	GCTGATGGCGATGAATGAACACTGAAGGTACAAAAGTTGATATCAGACTGGATG
		00471720-EGFP_Rv2	AAGTTCTTCACCCTTAGAAACCATGGATCCAATGATATTTTATTTGCTTTTTGAAAAAGG
	3' flanking	Neo4-00471720_Fw	CCCGGGGGATCTGAATTCGATATCAAGCTTAAGATTTACAAGTTGTTTATTTTACAC
		3Am-00471720_Rv	GCGAGCACAGAATTAATACGACTCTATTGTACAATTTTTCTTTTACTATTAGC
TTHERM_00471730	coding	5Am-00471730_Fw	GCTGATGGCGATGAATGAACACTGGAGCGCACTTTATGAATTATTCTCTCGTG
		00471730-EGFP_Rv	AAGTTCTTCACCCTTAGAAACCATGGATCCATCATCATTTTCATCATCTTACTATTATC
	3' flanking	Neo4-00471730_Fw	CCCGGGGGATCTGAATTCGATATCAAGCTTCCTCAATTTAATATAAATCAATTCAATTTC
		3Am-00471730_Rv	GCGAGCACAGAATTAATACGACTCTTAATATTAAATCATCATTTATGACATAG
TTHERM_00474360	coding	5Am-00474360_Fw2	GCTGATGGCGATGAATGAACACTGCTCAACAGATTAAATGCCCAGCTAAAGAGC
		00474360-EGFP_Rv	AAGTTCTTCACCCTTAGAAACCATGGATCCATTAGTATATAGGTTGTCTTAGTAATCATCAAC
	3' flanking	Neo4-00474360_Fw	CCCGGGGGATCTGAATTCGATATCAAGCTTATGAATTGTTCATTTTTTATCTATTACCTC
		3Am-00474360_Rv	GCGAGCACAGAATTAATACGACTTATCTTATTAACGTTTCTAATATTAATATC
TTHERM_00474920	coding	5Am-00474920_Fw	GCTGATGGCGATGAATGAACACTGCGAAAATAATATGAATCCTACTAGAGGAGA
		00474920-EGFP_Rv	AAGTTCTTCACCCTTAGAAACCATGGATCCGCTTTGGATATTTTCACAACAGAAAGTG
	3' flanking	Neo4-00474920_Fw	CCCGGGGGATCTGAATTCGATATCAAGCTTGAGTTTATTTTAAGTATTCAAATGAACTTG
		3Am-00474920_Rv	GCGAGCACAGAATTAATACGACTTTGGAAAGTAAATAACATTTGAAGAGAGAG
TTHERM_00487030	coding	5Am-00487030_Fw	GCTGATGGCGATGAATGAACACTGGTAAGAAACACCATGCAGTCAAGTGCTTTG
		00487030-EGFP_Rv	AAGTTCTTCACCCTTAGAAACCATGGATCCGCTCTTTATCTTTATTTATTCTGCAACTTC
	3' flanking	Neo4-00487030_Fw	CCCGGGGGATCTGAATTCGATATCAAGCTTGTGTCCTATATATATTCTATCTATCCACTC
		3Am-00487030_Rv	GCGAGCACAGAATTAATACGACTATAAGGAAAGATTTGGATAATTATTACATG
TTHERM_00497670	coding	5Am-00497670_Fw	GCTGATGGCGATGAATGAACACTGGTAATAAACTAAATATTGGATGTAAGGCCA
		00497670-EGFP_Rv	AAGTTCTTCACCCTTAGAAACCATGGATCCATAAAAACTATCTACATTAAATTATTTAAACC
	3' flanking	Neo4-00497670_Fw	CCCGGGGGATCTGAATTCGATATCAAGCTTGCATCTTAAGAGAAAAGAGTTATACATTAC
		3Am-00497670_Rv	GCGAGCACAGAATTAATACGACTTAATACGTAAATGTTGAAGGTGTTGGAACT
TTHERM_00499370	coding	5Am-00499370_Fw	GCTGATGGCGATGAATGAACACTGATAAAGCAAATGACCTATCTTTTCCT
		00499370-EGFP_Rv2	AAGTTCTTCACCCTTAGAAACCATGGATCCATTTAAAACGTCTTCTTATTTTGC
	3' flanking	Neo4-00499370_Fw2	CCCGGGGGATCTGAATTCGATATCAAGCTTCATAAGTAATAGTTTTTCAAGAATTAGCAG
		3Am-00499370_Rv	GCGAGCACAGAATTAATACGACTAGTTGAAATTTTTCCTAATCCTAAGACCT
TTHERM_00501010	coding	5Am-00501010_Fw	GCTGATGGCGATGAATGAACACTGATTTCATAAATAGAGTAGTTGGCTCTTCCT
		00501010-EGFP_Rv	AAGTTCTTCACCCTTAGAAACCATGGATCCTTAGTTATGTTCTAATTTATATGAGACTGT
	3' flanking	Neo4-00501010_Fw	CCCGGGGGATCTGAATTCGATATCAAGCTTTTAACACAAACTTAATTAACCAACCTGATG
		3Am-00501010_Rv	GCGAGCACAGAATTAATACGACTACAGGACATCCCCATATAGCTTTGCATGAT
TTHERM_00522820	coding	5'Am-00522820_Fw	GCTGATGGCGATGAATGAACACTGAATTTCTAAAACGTTCAAGAGCCTCAAAAGA
		00522820-EGFP_Rv2	AAGTTCTTCACCCTTAGAAACCATGGATCCTTCTTATTTGTGTAATTCACTATTTTGTTT
	3' flanking	Neo4-00522820_3'FLNK_Fw3	CCCGGGGGATCTGAATTCGATATCAAGCTTGAGCTTAAAAATTTGAAGTAAGCTAATAAT
		3'Am-00522820_Rv3	GCGAGCACAGAATTAATACGACTTACGATAAAGTTAATCGCCTGTCCAAGCAG
TTHERM_00526270a*	coding	5Am-00526270_Fw	GCTGATGGCGATGAATGAACACTGACGATGATGATGAAGAATTAGATGATGATG
		00526270-EGFP_Rv	AAGTTCTTCACCCTTAGAAACCATGGATCCCTTCTTCTACAGTAAGTTTTATTTTTTTTATTG
	3' flanking	Neo4-00526270_Fw	CCCGGGGGATCTGAATTCGATATCAAGCTTATCATATTCATAATAAAATCTTTTTTTC
		3Am-00526270_Rv	GCGAGCACAGAATTAATACGACTATCATAAAGTAAACTCTTAGGGAGACTCCA
TTHERM_00526270b*	coding	5Am-00526270-2_Fw	GCTGATGGCGATGAATGAACACTGTAAGGAGGTAAACCAAACAAAGCTCACTAC
		00526270-2-EGFP_Rv	AAGTTCTTCACCCTTAGAAACCATGGATCCAATACAATTCTGATTTTATAATAAGTTACT
	3' flanking	Neo4-00526270-2_Fw	CCCGGGGGATCTGAATTCGATATCAAGCTTTATTGTGATTTGCTCAATAAATTGTCAGTG
		3Am-00526270-2_Rv	GCGAGCACAGAATTAATACGACTAAACAAGGGAAAATTAGGATTAATATTGTG
TTHERM_00529550	coding	5Am-00529550_Fw	GCTGATGGCGATGAATGAACACTGTAATAATGCTAAGCCACTCAAAAGTTAGCT
		00529550-EGFP_Rv	AAGTTCTTCACCCTTAGAAACCATGGATCCTTTATTCTAATTATTGATCACCTGTCTTG
	3' flanking	Neo4-00529550_Fw	CCCGGGGGATCTGAATTCGATATCAAGCTTAGAATGGCATAAATACTCTAAGAAAAAGGA
		3Am-00529550_Rv	GCGAGCACAGAATTAATACGACTTTAATTGTGATATAATCTACTACGAAGGTG

TTHERM_00540090	coding	5Am-00540090_Fw	GCTGATGGCGATGAATGAACACTGACGCAGCACTAATTTTACTAGGTTTTCAAG
		00540090-EGFP_Rv	AAGTTCTTCACCCTTAGAAACCATGGATCCGACTCTTGAAGAGGAATCTGGCTTTTCGAG
	3' flanking	Neo4-00540090_Fw	CCCGGGGGATCTGAATTCGATATCAAGCTTGATGCTAGATTTTCTTAACTAGCTTTAG
		3Am-00540090_Rv	GCGAGCACAGAATTAATACGACTTTTACTTGATTTGCTTTTCACCATTTTCTC
TTHERM_00564480	coding	5Am-00564480_Fw2	GCTGATGGCGATGAATGAACACTGCTTCAAATTCCAATGATGATAACGATGACAG
		00564480-EGFP_Rv	AAGTTCTTCACCCTTAGAAACCATGGATCCATTATACTTCTTGCTTAATTTTTAATCTAAG
	3' flanking	Neo4-00564480_Fw	CCCGGGGGATCTGAATTCGATATCAAGCTTGTATTATATTTCAACTTACCAGTGTTTATGG
		3Am-00564480_Rv	GCGAGCACAGAATTAATACGACTACCACTAAAAGAGCCATCCGAGTCCTAATC
TTHERM_00569460	coding	5Am-00569460_2_Fw	GCTGATGGCGATGAATGAACACTGATTAACATTAAAAGCTATCAAAACCTAGCT
		00569460-EGFP_Rv	AAGTTCTTCACCCTTAGAAACCATGGATCCTATTGAAAACTTCTTAAAGTTCTCATAAGA
	3' flanking	Neo4-00569460_Fw	CCCGGGGGATCTGAATTCGATATCAAGCTTAAAGATTCAATCTATGACTCTTGCTGCAGT
		3Am-00569460_Rv	GCGAGCACAGAATTAATACGACTCTCCAAAAGAGTAAGTGACTATTATTTAGC
TTHERM_00572190	coding	5'Am-00572190_Fw	GCTGATGGCGATGAATGAACACTGGAGGAAAACAAGCAGTTTGTTGAGACGTAT
		00572190-EGFP_Rv	AAGTTCTTCACCCTTAGAAACCATGGATCCATTAAAATATGTTTCGATGATCGATTACAG
	3' flanking	Neo4-00572190_3'FLNK_Fw	CCCGGGGGATCTGAATTCGATATCAAGCTTCTGTATAGATTTAATTAAGATGTTAAACTA
		3'Am-00572190_Rv	GCGAGCACAGAATTAATACGACTGCCATTGACATGCCCTTACTTTTACTTTCT
TTHERM_00616290	coding	5Am-00616290_Fw	GCTGATGGCGATGAATGAACACTGGGTGGTAGTAGAGGAGTAATTACAAGATCA
		00616290-EGFP_Rv	AAGTTCTTCACCCTTAGAAACCATGGATCCACTCTTTACAATTTTTCTTTTTTATATTTTTTATTTTG
	3' flanking	Neo4-00616290_Fw	CCCGGGGGATCTGAATTCGATATCAAGCTTAATACTTCATGTAAAATATTGCTGTAC
		3Am-00616290_Rv	GCGAGCACAGAATTAATACGACTAACTGCTTACTTATCTTTTAGATAAGC
TTHERM_00622830	coding	5Am-00622830_Fw	GCTGATGGCGATGAATGAACACTGCACATACCTTGAGAAAATGAGCAAAGAATC
		00622830-EGFP_Rv	AAGTTCTTCACCCTTAGAAACCATGGATCCTGGCTAGCTAATATTCCTTTCGCTTATATTC
	3' flanking	Neo4-00622830_Fw	CCCGGGGGATCTGAATTCGATATCAAGCTTATCATTTATGTATGTATGTAAAGCTCTATC
		3Am-00622830_Rv	GCGAGCACAGAATTAATACGACTAGCAATTTGAATAAAATTCTAAACAGATGC
TTHERM_00637050	coding	5Am-00637050_Fw	GCTGATGGCGATGAATGAACACTGATAGTTAGAGAGGAATTAGTAATCTAAGCT
		00637050-EGFP_Rv	AAGTTCTTCACCCTTAGAAACCATGGATCCTGCTTCAACAATTTCACTATGGTTTGGAG
	3' flanking	Neo4-00637050_Fw	CCCGGGGGATCTGAATTCGATATCAAGCTTAGAAATTTTTCTTCCAATTCTCCCTAAATC
		3Am-00637050_Rv	GCGAGCACAGAATTAATACGACTATGTCAAATCTTCTCTCCTAAATTAATC
TTHERM_00649180	coding	5Am-00649180_Fw	GCTGATGGCGATGAATGAACACTGAACGATAAGGGTGATATCGATGTTGATAGC
		00649180-EGFP_Rv2	AAGTTCTTCACCCTTAGAAACCATGGATCCGAAGGTAATGGATTCATCGTTATCATTAGG
	3' flanking	Neo4-00649180_Fw	CCCGGGGGATCTGAATTCGATATCAAGCTTTATGTATCACAAATTCCATCTTCTAACACA
		3Am-00649180_Rv	GCGAGCACAGAATTAATACGACTGAAGAAATTATGCATGTTTTGTGAAAATCA
TTHERM_00664050	coding	5'Am-00664050_Fw	GCTGATGGCGATGAATGAACACTGCGAAGACAAACAACAGAGGATAGTATGGGA
		00664050-EGFP_Rv	AAGTTCTTCACCCTTAGAAACCATGGATCCGTATAGTCTACGATAATGTTCATTTATGCC
	3' flanking	Neo4-00664050_3'FLNK_Fw	CCCGGGGGATCTGAATTCGATATCAAGCTTTTTTGTTTGTTTATTTGTTGTCAATTTCTG
		3'Am-00664050_Rv	GCGAGCACAGAATTAATACGACTTAAGTGCGTGCTGGAATGAAATTGAAATAG
TTHERM_00693080	coding	5Am-00693080_Fw	GCTGATGGCGATGAATGAACACTGTTCTTATTGACTGGCTTACTGAAGTTTCCA
		00693080-EGFP_Rv	AAGTTCTTCACCCTTAGAAACCATGGATCCAATATTGGTTAGTTGTTAAGATTATTATTAG
	3' flanking	Neo4-00693080_Fw	CCCGGGGGATCTGAATTCGATATCAAGCTTAATAGAGCCATAAAACTATACTTATTGTTG
		3Am-00693080_Rv	GCGAGCACAGAATTAATACGACTCAATAGGATATTATAACTGTGTGCTCTTAG
TTHERM_00704020*	coding	5Am-00704020_Fw	GCTGATGGCGATGAATGAACACTGCATCCTCATTCAGTAATCATTTAATCTTAC
		00704020-EGFP_Rv	AAGTTCTTCACCCTTAGAAACCATGGATCCTTTTTTCTTTTTACTAAACTTTGCAAAAGC
	3' flanking	Neo4-00704020_Fw	CCCGGGGGATCTGAATTCGATATCAAGCTTACATATTCACTAAATTGAAGTCAAAATACT
		3Am-00704020_Rv	GCGAGCACAGAATTAATACGACTATTCTTCATAAACTTAACACATTTATCAAC
TTHERM_00706330	coding	5Am-00706330_Fw	GCTGATGGCGATGAATGAACACTGTTCAAAGCCTATATCAAAGGGAGTAATAGA
		00706330-EGFP_Rv	AAGTTCTTCACCCTTAGAAACCATGGATCCGTTAATTAATAAAACCTCCAATTCTTAATTTTC
	3' flanking	Neo4-00706330_Fw	CCCGGGGGATCTGAATTCGATATCAAGCTTCTCATTAATAAGTGCTTGATTGATTTATGA
		3Am-00706330_Rv	GCGAGCACAGAATTAATACGACTTGCAAAACACACAGAAAGTATGCTATTTGC
TTHERM_00728900	coding	5Am-00728900_Fw	GCTGATGGCGATGAATGAACACTGTGAATAATTTTGAAACATTATGGAGAAATC
		00728900-EGFP_Rv	AAGTTCTTCACCCTTAGAAACCATGGATCCAACTTATGCTTATTTTGAATCTAATTATG
	3' flanking	Neo4-00728900_Fw	CCCGGGGGATCTGAATTCGATATCAAGCTTTTGAAGAGAGTGGCAGCAAGATTCATCA
		3Am-00728900_Rv2	GCGAGCACAGAATTAATACGACTCTTTCACATTCTACTTTAGAGGAAGTATTC
TTHERM_00775940	coding	5Am-00775940-1_Fw	GCTGATGGCGATGAATGAACACTGCTATATTTGGTGTAGGGATTAGTTTGCTCG
		00775940-1-EGFP_Rv	AAGTTCTTCACCCTTAGAAACCATGGATCCCTATTTCAAGCTACCAAAACCTTGCTTAAT
	3' flanking	Neo4-00775940-1_Fw	CCCGGGGGATCTGAATTCGATATCAAGCTTATGAGCAAGGACTCTTATATCTATCTATAC
		3Am-00775940-1_Rv	GCGAGCACAGAATTAATACGACTACTACTGTCTAAAACAATGTAGATTTATGG
TTHERM_00775949	coding	5Am-00775940-2_2_Fw	GCTGATGGCGATGAATGAACACTGTACTTCCTAAAAAGCACTTAAAATCAGGCT
		00775940-2-EGFP_Rv	AAGTTCTTCACCCTTAGAAACCATGGATCCCAACGTGTTTAGAAAGCTTAAAAGTAATAAAC
	3' flanking	Neo4-00775940-2_Fw	CCCGGGGGATCTGAATTCGATATCAAGCTTTTCATTCTAATTTAAACCTCAGAATTACAC
		3Am-00775940-2_Rv	GCGAGCACAGAATTAATACGACTTCACTTCTTATTCGTTTGCAAACATTCGTG
TTHERM_00825660	coding	5Am-00825660_Fw	GCTGATGGCGATGAATGAACACTGGAATTCGATGATGATGAAGAAGAAGTAGAG
		00825660-EGFP_Rv	AAGTTCTTCACCCTTAGAAACCATGGATCCATTCTTTTTAGTTTTCTTTTAGGATCTG
	3' flanking	Neo4-00825660_Fw	CCCGGGGGATCTGAATTCGATATCAAGCTTTCTATTCATATTATTTGTTTATGTTCG
		3Am-00825660_Rv	GCGAGCACAGAATTAATACGACTCATAACTTAAAACAAGTAACAATGAGTC
TTHERM_00849260	coding	5'Am-00849260_Fw	GCTGATGGCGATGAATGAACACTGGTGAATAATTTAATAATGGTTATCCTCGCT
		00849260-EGFP_Rv	AAGTTCTTCACCCTTAGAAACCATGGATCCTTCTTGCTTCTACTAAATTTAAATAGTATC
	3' flanking	Neo4-00849260_Fw	CCCGGGGGATCTGAATTCGATATCAAGCTTCTAAGTGGATATTTCAAATTACATATCATG
		3'Am-00849260_Rv	GCGAGCACAGAATTAATACGACTATTAATACAATAATATGCTTTGCGTCCACT
TTHERM_00974120	coding	5Am-00974120_Fw	GCTGATGGCGATGAATGAACACTGTGAAATAGTTTAATAATAGGTCTGTGTGTG
		00974120-EGFP_Rv	AAGTTCTTCACCCTTAGAAACCATGGATCCTTGTTTTAAGTATATATCATACTATTTG
	3' flanking	Neo4-00974120_Fw	CCCGGGGGATCTGAATTCGATATCAAGCTTGCTTAAAAGCAATGATTTCAAGATCTCTGC
		3Am-00974120_Rv	GCGAGCACAGAATTAATACGACTGATAATAGAAATAGATTTATATACCCACTG
TTHERM_01014530	coding	5Am-01014530_Fw	GCTGATGGCGATGAATGAACACTGAGAAAAGGAACTTCCTCCATATTGCAAGGA
		01014530-EGFP_Rv	AAGTTCTTCACCCTTAGAAACCATGGATCCTTAAGGGGATTGTTGAGAGTTTGATTTCTG
	3' flanking	Neo4-01014530_Fw	CCCGGGGGATCTGAATTCGATATCAAGCTTATTAAACTATCAAATTAGATTCATTCTCA
		3Am-01014530_Rv	GCGAGCACAGAATTAATACGACTTGAGAACGAACTTGAATACCAGTCGGT
TTHERM_01014770	coding	5Am-01014770_Fw	GCTGATGGCGATGAATGAACACTGACAATCTACCAATTGGTAAAGTGACTATGG
		01014770-EGFP_Rv	AAGTTCTTCACCCTTAGAAACCATGGATCCATCCTTTTTGAGCATTTTATCTGGGTTTCC
	3' flanking	Neo4-01014770_Fw	CCCGGGGGATCTGAATTCGATATCAAGCTTTCAACTTATTTTGATATTATGTCTGAATTG
		3Am-01014770_Rv	GCGAGCACAGAATTAATACGACTGTTGATTTGTAAAAATTTGATCAAAAATAC
TTHERM_01085480	coding	5Am-01085480_Fw	GCTGATGGCGATGAATGAACACTGGATATAGCCCAACGAAGAATCTTTTAATAC

		01085480-EGFP_Rv	AAGTTCTTCACCCTTAGAAACCATGGATCCCACAAAGTATTTTTTGTTTGATCTCTTAAT
	3' flanking	Neo4-01085480_Fw	CCCGGGGGATCTGAATTCGATATCAAGCTTTTATCTTGGAATTACTGTGGAATTTCTCAG
		3Am-01085480_Rv	GCGAGCACAGAATTAATACGACTACAAAATACTCAGGATGAAGAGATTAGTTC
TTHERM_01132870	coding	5Am-01132870_Fw	GCTGATGGCGATGAATGAACACTGGAGGGAAATGATATTGATTAAATTTTGGCA
		01132870-EGFP_Rv2	AAGTTCTTCACCCTTAGAAACCATGGATCCGCTTTTTTAATCTTTTTTTGTTGATGATC
	3' flanking	Neo4-01132870_Fw2	CCCGGGGGATCTGAATTCGATATCAAGCTTAGTTTATAAATAAAAATTCTTCCTTTCATC
		3Am-01132870_Rv2	GCGAGCACAGAATTAATACGACTGATAACTTATTCATATCAGATTTCACTGTG
TTHERM_01276320	coding	5'Am-01276320_Fw	GCTGATGGCGATGAATGAACACTGGAGATTTCCTAAGTTTTATTATGAAAATACATTTATGGGTC
		01276320-EGFP_Rv2	AAGTTCTTCACCCTTAGAAACCATGGATCCGCTTTGTAATCTCTAAGGACATATAGATTT
	3' flanking	Neo4-01276320_3'FLNK_Fw3	CCCGGGGGATCTGAATTCGATATCAAGCTTGAAAACCTTTTAAAAATTGATTTTTGTGAG
		3'Am-01276320_Rv3	GCGAGCACAGAATTAATACGACTCTAATCAATACTTTGATAGGTACATTAGAGACT
TTHERM_01285910	coding	5Am-01285910_Fw	GCTGATGGCGATGAATGAACACTGAGACGAGAGCTTTTTAAATTATCAAAGCCA
		01285910-EGFP_Rv	AAGTTCTTCACCCTTAGAAACCATGGATCCATTAAAGTCAATCAATTCATATAAAATATTCG
	3' flanking	Neo4-01285910_Fw	CCCGGGGGATCTGAATTCGATATCAAGCTTTGATAATATTCAATGTAATATTTTAGTTCG
		3Am-01285910_Rv	GCGAGCACAGAATTAATACGACTAATGTAGTAGACTTTCCTACATTACGCTTG
TTHERM_01337400	coding	5'Am-01337400_Fw	GCTGATGGCGATGAATGAACACTGGATTGTCCCTGAATAAATAGATGTGGACTG
		01337400-EGFP_Rv	AAGTTCTTCACCCTTAGAAACCATGGATCCTTTAGCTATTTATTACTATTATTTCTATATTAG
	3' flanking	Neo4-01337400_Fw	CCCGGGGGATCTGAATTCGATATCAAGCTTTATATCTCTTCATAACTTAAATTGCTATTC
		3'Am-01337400_Rv	GCGAGCACAGAATTAATACGACTTTATCAGAGAGGATCCTGAGGTTGTCAAAG
TTHERM_01358410	coding	5Am-01358410_Fw	GCTGATGGCGATGAATGAACACTGATTTCCATAAGCTGAATGCGATGCGTTCTC
		01358410-EGFP_Rv	AAGTTCTTCACCCTTAGAAACCATGGATCCGTTGTTTTATTAACAAACCCATGGGTGGTT
	3' flanking	Neo4-01358410_Fw	CCCGGGGGATCTGAATTCGATATCAAGCTTTTGTAATTTATTTTATCTCCTATGATTTTG
		3Am-01358410_Rv	GCGAGCACAGAATTAATACGACTTTAGCATTCGCTATTTAAACTTAATATTTG
TTHERM_01367700	coding	5'Am-01367700_Fw	GCTGATGGCGATGAATGAACACTGGATTAATAAGCTGTTAAAAGTGAAAGCAAA
		01367700-EGFP_Rv2	AAGTTCTTCACCCTTAGAAACCATGGATCCTTTTTTAATAATTTTAAAGTGTTTAATAGG
	3' flanking	Neo4-01367700_3'FLNK_Fw	CCCGGGGGATCTGAATTCGATATCAAGCTTTTGTAAATTCTTTTGGTTTTTTAATGGAGT
		3'Am-01367700_Rv	GCGAGCACAGAATTAATACGACTTGAACAGTGTTTTTTATTCCTATTAATGGA

Overlapping and adapter sequences are underlined and dotted underlined, respectively.

b) Primers for the overlapping PCR

name	sequence (5' -> 3')
5’RACE Outer	GCTGATGGCGATGAATGAACACTG
3’RACE Outer	GCGAGCACAGAATTAATACGACT

Primers used for the other experiments

name	sequence (5' -> 3')
00237610_KO5Fw	AACAGCTATTTTGAATACATTAGTGGAG
00237610_KO5Rv	GTCTATCGAATTCCTGCAGCCCATAAATTTAATGAATCTGAATGCTTTC
00237610_KO3Fw	CTGGAAAAATGCAGCCCTCTATTAAAGCTTATTTTATATTTCGATGA
00237610_Fw1	AATTGTCAGAAAAAATGAAAAAAATTATC
00237610_Rv4	GTTAATTAAAATAAATTGATTAATAATAC
00237610_Fw9	GTTAAGCTTAATATAAAATATACGATCC
BTU1_5Fw	AATATGTGAAAATATCAAGCGAACTGAC
BTU1_5bsrRv	CCATACTTTGAAGATATCTGTCGACCTTTTCATCACCCAAATAAATACACGCA
BTU1_3bsrFw	TAATATTTTTTTTTGCCGCGGCCGCAATTCTTACTTCTACATGTTTCCTTTC
BTU1_3Rv	TGCTATAAGTCGCTTTTTAAAGAACTAAG
BX-5FNK_PDD1_FwL	AGGCTCGCCCTAGGTCACTCGAGCACATAAATTTAATCCAAATCATCAATCAC
SSB-5FNK_PDD1_Rv	CAGTGAGTCGACGAGTCCACTAGTACGTCGGGATCCTTTCTTTATTGAAATATTTATTGCTTTTTTAGC
SSS-3FNK_PDD1_Fw	CGACGTACTAGTGGACTCGTCGACTCACTGCCCGGGTTTTATCAAATTAAAATTTAAATAGGATTGATTG
KX-3FNK_PDD1_RvL	AGGCTCGCGGTACCAACCTCGAGTAGACCAATTATCGAGAATGTTGATAATAGC
SpeI-PDD1_3UTR_Fw	CGTCGAACTAGTTAAATTATTTAGTGATTACGGCTTGATTAAGC
SalI-PDD1_3UTR_Rv	CGTCGAGTCGACTATTTTAATTGAATAAATTTTCATAAATTAAAATGTG
BamHI-PDD1ORF_Fw	AGTCAGGATCCATGTCTCAGAAAAAGAGTTTAAAATAAAAGAG
SpeI-PDD1ORF_Rv2	AACACTGACTAGTTCAATGAGTAAGTTATTAATTAGCTTGTC
5Am-Pdd1_Fw	GCTGATGGCGATGAATGAACACTGATGTCTCTTAGCCATAATGGGTAGATGTTG
EcoRI-opt.PDD1_Fw	AGCAGAATTCATGAGCCAAAAAAAATCCCTGAAACAAAAACG
opt.PDD1_W72,75A_Rv1	GGTTGAATCTTCGATCGGGGCGTTCTCGGCTTTCACCAGATACTC
opt.PDD1_W72,75A_Fw1	GAGTATCTGGTGAAAGCCGAGAACGCCCCGATCGAAGATTCAACC
PstI-TGA-opt.PDD1_Rv2	ATCGCTGCAGTCAATGGGTCAGTTGCTGATTTGCCTGGC
PstI-TGA-opt.PDD1_I478D_RvMAL	ATCGCTGCAGTCAATGGGTCAGTTGCTGATTTGCCTGGCGCAGCTGTGACTGATTGGAGTGTTGCAGCAGAAAGTCGTCCAGGACTTGTGG
ERI-opt.PDD1_HNG1_Fw	AGCAGAATTCAAAAAACAGAAAGCCAACGTGATGCCTCAACC
PstI-TGA-opt.PDD1_HING1_Rv	ATCGCTGCAGTCACAGTTCCGTTTCATCGGCATCGGCGTTATTCG
ERI-opt.PDD1_HNG2_Fw	AGCAGAATTCATCAAAGATGACGTGATCGCCTATGAGGAC
PstI-TGA-opt.PDD1_HING2_Rv	ATCGCTGCAGTCAAATTTCAGTCAGTTCAATCGGGCGACGTTTC
EcoRI-opt.PDD1_FwpGEX	CACGAATTCTGATGAGCCAAAAAAAATCCCTGAAACAAAAACG
XhoI-opt.PDD1_RvpGEX	CATCTCGAGTATGGGTCAGTTGCTGATTTGCCTGGC
ERI-opt.MIM22_FwGEX	CAGCGAATTCTGATGTCTCAAAAAAAAAGCCTG
XhoI-opt.MIM22_RvGEX	CAGTCTCGAGTGTGCGTCAACTGCTGGTTGG
T7-opt.EGFP_Fw1	GTAATACGACTCACTATAGGGAGAATGGTTTCTAAGGGTGAAGAAC
opt.EGFP_Rv1	CTTATATAATTCATCCATACCAAGAGTAATACC
T7-CAM-IES_Fw1	GTAATACGACTCACTATAGGGAGAAATTTACAAAAGTTTGAATAATGAATTTTAGCAC
CAM-IES_Rv1	TTATTTTAAGATTTATTTTCAATTTGATTCAAG

A map and the sequence of pPPLNP1

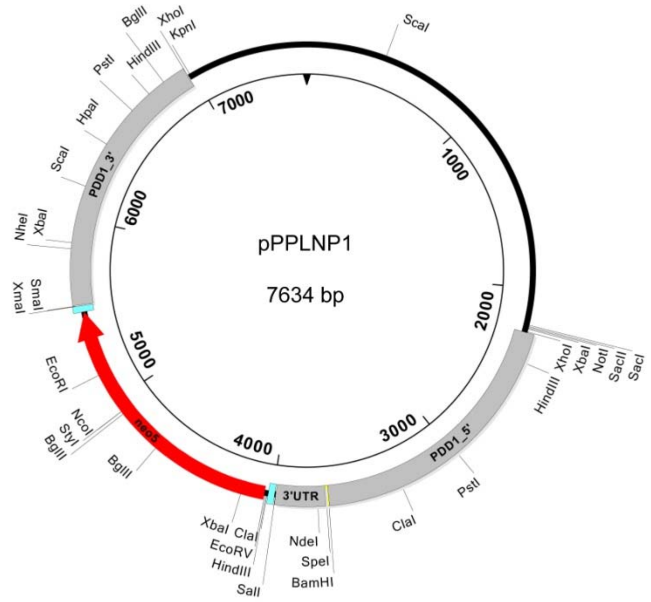

>pPPLNP1
GCACTTTTCGGGGAAATGTGCGCGGAACCCCTATTTGTTTATTTTTCTAAATACATTCAAATATGTATCCGCTCATGAGACAATAACCCTGATAAATGCTTCAATAATATTGAAAAAGGAAGAGTATGAGTATTCAACATT
TCCGTGTCGCCCTTATTCCCTTTTTTGCGGCATTTTGCCTTCCTGTTTTTGCTCACCCAGAAACGCTGGTGAAAGTAAAAGATGCTGAAGATCAGTTGGGTGCACGAGTGGGTTACATCGAACTGGATCTCAACAGCGGTA
AGATCCTTGAGAGTTTTCGCCCCGAAGAACGTTTTCCAATGATGAGCACTTTTAAAGTTCTGCTATGTGGCGCGGTATTATCCCGTATTGACGCCGGGCAAGAGCAACTCGGTCGCCGCATACACTATTCTCAGAATGACT
TGGTTGAGTACTCACCAGTCACAGAAAAGCATCTTACGGATGGCATGACAGTAAGAGAATTATGCAGTGCTGCCATAACCATGAGTGATAACACTGCGGCCAACTTACTTCTGACAACGATCGGAGGACCGAAGGAGCTAA
CCGCTTTTTTGCACAACATGGGGGATCATGTAACTCGCCTTGATCGTTGGGAACCGGAGCTGAATGAAGCCATACCAAACGACGAGCGTGACACCACGATGCCTGTAGCAATGGCAACAACGTTGCGCAAACTATTAACTG
GCGAACTACTTACTCTAGCTTCCCGGCAACAATTAATAGACTGGATGGAGGCGGATAAAGTTGCAGGACCACTTCTGCGCTCGGCCCTTCCGGCTGGCTGGTTTATTGCTGATAAATCTGGAGCCGGTGAGCGTGGGTCTC
GCGGTATCATTGCAGCACTGGGGCCAGATGGTAAGCCCTCCCGTATCGTAGTTATCTACACGACGGGGAGTCAGGCAACTATGGATGAACGAAATAGACAGATCGCTGAGATAGGTGCCTCACTGATTAAGCATTGGTAAC
TGTCAGACCAAGTTTACTCATATATACTTTAGATTGATTTAAAACTTCATTTTTAATTTAAAAGGATCTAGGTGAAGATCCTTTTTGATAATCTCATGACCAAAATCCCTTAACGTGAGTTTTCGTTCCACTGAGCGTCAG
ACCCCGTAGAAAAGATCAAAGGATCTTCTTGAGATCCTTTTTTTCTGCGCGTAATCTGCTGCTTGCAAACAAAAAAACCACCGCTACCAGCGGTGGTTTGTTTGCCGGATCAAGAGCTACCAACTCTTTTTCCGAAGGTAA
CTGGCTTCAGCAGAGCGCAGATACCAAATACTGTCCTTCTAGTGTAGCCGTAGTTAGGCCACCACTTCAAGAACTCTGTAGCACCGCCTACATACCTCGCTCTGCTAATCCTGTTACCAGTGGCTGCTGCCAGTGGCGATA
AGTCGTGTCTTACCGGGTTGGACTCAAGACGATAGTTACCGGATAAGGCGCAGCGGTCGGGCTGAACGGGGGGTTCGTGCACACAGCCCAGCTTGGAGCGAACGACCTACACCGAACTGAGATACCTACAGCGTGAGCTAT
GAGAAAGCGCCACGCTTCCCGAAGGGAGAAAGGCGGACAGGTATCCGGTAAGCGGCAGGGTCGGAACAGGAGAGCGCACGAGGGAGCTTCCAGGGGGAAACGCCTGGTATCTTTATAGTCCTGTCGGGTTTCGCCACCTCT
GACTTGAGCGTCGATTTTTGTGATGCTCGTCAGGGGGGCGGAGCCTATGGAAAAACGCCAGCAACGCGGCCTTTTTACGGTTCCTGGCCTTTTGCTGGCCTTTTGCTCACATGTTCTTTCCTGCGTTATCCCCTGATTCTG
TGGATAACCGTATTACCGCCTTTGAGTGAGCTGATACCGCTCGCCGCAGCCGAACGACCGAGCGCAGCGAGTCAGTGAGCGAGGAAGCGGAAGAGCGCCCAATACGCAAACCGCCTCTCCCCGCGCGTTGGCCGATTCATT
AATGCAGCTGGCACGACAGGTTTCCCGACTGGAAAGCGGGCAGTGAGCGCAACGCAATTAATGTGAGTTAGCTCACTCATTAGGCACCCCAGGCTTTACACTTTATGCTTCCGGCTCGTATGTTGTGTGGAATTGTGAGCG
GATAACAATTTCACACAGGAAACAGCTATGACCATGATTACGCCAAGCTCGAAATTAACCCTCACTAAAGGGAACAAAAGCTGGAGCTCCACCGCGGTGGCGGCCGCTCTAGAACTAGGTCACTCGAGCACATAAATTTAA
TCCAAATCATCAATCACAAAATAAATAGAAATATTTATTTTATACAAACAGCTGAAATAAAGAAAATATTTTACATTAGCTGTTTAACTCTATTCTAAAATTCAATGCAAATTTAATGCCTTTGCTGAAAATAAATTATAA
GCTTTTTGATAAATATCGCTAAAATGCTGATTGATAATACCATTTTTGATTTACTTTTTAAATCTGTTTAATAAAAATATAAAAAGTTGCTCAAAAATCTGATTTTTTTTTGATATTGAAATCAGATTAAAATCTAAAATT
AAGGCTAACAAATTTATTATTATTATTCAAAAGAACCCATAATTTATTACATTATAAGAAAGTTTAAATATTGGAAGGCCTTTGCCGTAATGAAAAACTTTATCATCAGTAGGTAATCCTTACCTATATATTTCTAATGAT
GAAGGAGATGGCCATATCCCTCGGGATTGAAAATCATGAAAAACTGGCTGCAATGCTGATCAAAGAAATATAAATTTAAGGATATACCGTACAAATTAAATAGCAAAACAAGCTACTCTATTATAAATTTTTCGAAAGAAA
ATATATAGAGTGTAGCTTAAGCTATAATTATATATATTTAATTATGCATATTTACTAAATTAATACTGCTATAAAATTCCCTATCACGAAAAGTCCAAGTGATGGAATAAAGAATTTACCTTATACAGTTAAAATATTTTA
GTTACCATTGCGCACTGCAGCAAATAAACATGATTGTTGAGGTTTTCTCAATAATTTGTTTGTTTTGGTAATCTGACATAACAACTCATATAAAAAAAACAAAACACTTCTCTTTTTTTCTTGATTAAAAAAAATTTAAAT
TTTCAATCCCTATTTCAACGATAGGTAAATAAAAAGAAATTTATTTATGCTACAAATACAAAAAATATTACAAAAAGAATTTAAATAAATTACTAAGTCGGTATTGTCTTAGTCATTCATTTATTTATTTATTTTTCAAAG
TTTAAAAAAGAATAAAATCAATAAAAAATAAAGCATTCATCTTTAAATAAAATATGATTAATTCAACATCGATTTATTTATTTAGATTTGTATTCATAAAATCTTATTGTTTTTTAGATTGATTAGAATAAATAATTAATA
TTTTAATCCTACCTACTAATAAATTTCTAAAATTTTAATTGATTTTGTTTGTCTATTTTTGATATTTGAGATTTCATCCGAATTATTTGAGTCTTTTTCACTAAGTTTAATTAGGTAAACAACTTTAAACATTTAGAATTT
AATTTAATTAAAACTATTGATTAATGCTTTGATGTTTACTGTGAAATCACTCTAATAATTTGTAAATAATTATTTTAAATCCAACCAAATATTTTGTTAATTTTATAAAAATAATAAATTTAAAAATTCTCCTAATTAGTA
AGCTAAAAAAGCAATAAATATTTCAATAAAGAAAGGATCCcgacgtACTAGTTAAATTATTTAGTGATTACGGCTTGATTAAGCTCTTAAAACATATGATGAACTCAATAAATTTTCTTATTAGAAACCAAAAAAATACTT
GGAAAAAAATTTTTAAAATAAATATATTTCTCATGATAAAAATATATTTTTTTGAAAAATATTGCTGATTATTTATTTATTAATTATTACATTTTACATATTTTGTAACGATTGAGTTTTTAGTTTAAAGAGTTTTAATTT
CACATTTTAATTTATGAAAATTTATTCAATTAAAATAGTCGACATAACTTCGTATAATGTATGCTATACGAAGTTATGGTATCGATAAGCTTGATATCTTCAAAGTATGGATTAATTATTTCAAATTATTAGAAGGTAATT
AATCTGCATAAATTCAAAACTATAAAAATAAAACATTAAAATTAATTCAACCTTATTGAAGCATCAAAATCTGAATCTCTAGAAAGACTGATTCTGATTGGATAATTTTTCGGCGCTAAGGATTTTGGATTAAAGAAAATT
AGATTTAATTATTAATCATGATTTGAATAGGATAGCAAGAATATTTGTTTGGTTTAAAAGGGAAAGCGGGTAATTATCAAAAATTTATAAATAATTTTAAAACAATAAATAGAAAAACAAATAAGATTATAAAAACTTACA
AAAATGATTGAACAAGATGGTTTACACGCTGGTTCTCCCGCCGCTTGGGTCGAAAGACTTTTCGGTTATGACTGGGCTCAACAAACCATCGGTTGCTCTGATGCCGCCGTCTTCCGTCTTTCTGCTCAAGGTCGTCCTGTT
CTTTTCGTCAAGACCGACCTTTCTGGTGCCCTTAATGAACTTCAAGATGAAGCTGCCCGTCTTTCTTGGCTTGCCACCACCGGTGTTCCTTGCGCTGCTGTCCTTGACGTTGTCACTGAAGCCGGTAGAGACTGGCTTCTT
TTAGGTGAAGTCCCCGGTCAAGATCTTCTTTCTTCTCACCTTGCTCCTGCCGAAAAAGTTTCTATCATGGCTGATGCTATGCGTCGTCTTCATACCCTTGATCCCGCTACCTGCCCTTTCGACCACCAAGCCAAACATCGT
ATCGAACGTGCTCGTACTCGTATGGAAGCCGGTCTTGTCGATCAAGATGATCTTGACGAAGAACATCAAGGTCTTGCCCCTGCCGAACTTTTCGCCAGACTTAAGGCCCGTATGCCCGACGGTGAAGATCTTGTCGTCACC
CATGGTGATGCCTGCTTACCCAATATCATGGTTGAAAATGGTCGTTTTTCTGGTTTCATCGACTGTGGTCGTCTTGGTGTCGCCGACCGTTATCAAGATATTGCCTTAGCTACCCGTGATATTGCTGAAGAACTTGGTGGT
GAATGGGCTGACCGTTTCCTTGTCCTTTACGGTATCGCCGCTCCCGATTCTCAACGTATCGCCTTCTATCGTCTTCTTGACGAATTCTTCTGAGATCCTTAAATTAAAAATTCAATATATATTTACAAACTTTCATATAAA
ATAAATATATTATATAAAATTAATTTTTAGTGTATTATATTAACATTAAAGCACCAAAAAAACGTGTTAATATACTACTATAAAATATAATTTATTCCAAATTGACTAAAATCATTATTTTACAACTCATTTGTATATATA
TTTTATGTCAATTATTTTTTTTAACTTTCTAAAAAAAAAAAATTCCTCTTCACATACATGTTAGCTCTTAAAAATTTGTCTGCAAATCCAATAATAATATTTTTTTTTGCCATTAAATTTTCAAATTTTTACTGGAAAAATG
CAGCATAACTTCGTATAATGTATGCTATACGAAGTTATCCCGGGTTTTATCAAATTAAAATTTAAATAGGATTGATTGATTTAAAAAAGATTAAATTAGGAAAATGGTTTGATTTATTTAAAACATAATCGAAACATAAAA
AATTAATATAGTATTATTCCATGTATTTATAAATTAGACTCAATAGATTTAGTTTTATGGTGTAGCTTCTTCTTAAACATCTTAGATTATTTGTTAATATAGTTAAACTCCTATTTTTTAATCGCTAAATTTATTACTTTT
TTACTTTTAGGCTTAACCTATAGATAATTAAGATAAATCATGATCGTGAGAATCTAGCTAGCGAGATAGCTGAGCGATAGAAGAAACTCTAGAATTGTGTGATTAAATAAATTTTTCTCTATCTTCTTTTGTATTCAAATA
TTAGTTTTACTTATCTTCAGCTTAATCAAAATTTTATAGAGAATTTATTTGTGGTTCAAAGTATTCTTATTCTTCATGCACATGTGTAGGACTTACGTTATGGCCGTAATATTCGTCGTTAGTTGGGAGTTAGATTCTTGT
TTTTAGTTTAATTGCACTGCTTGGAAGGAATTTTTTGCTTTACCCTCCTAACTAACTTTAACTAGCTGCAAAATGAGTAAAAGTAGTTTTTTGAAAACTTTAAGTACTTTTTTATTGCTTTTATTACTCATGAGGTTATCT
ATAAACTGACTGCAAAATATTTTAATCTTAGTTAGCTTAGTTGAATGTTTTCAATTTTATATTTTAGCTGCTTTCACCTTATTACAAGACTTCCTCAGAGTATTGAGACTTGTTATCGAATGTTTTCAAATTAAATTCCTA
AGAAGATGGCTTCACTGCAAATTTAGGGTTATACATTTTTTAAATATCTGTCTAAGATGGAGGTTAACGAATCTTTGTTTAAGATTTTTCTAAATTGTTAATTGAAATTTAATTTTCACTTCCACTTTTGTTATTTCCTTA
TCCTTAATGCTAACCTGCAATTTTAGCTTAGTTGTTGATTTATTTATTCGAAAATGTAGCATAGTAACCTTATTACATATTTTAGTTAAAAGAAGTATTATTGTTTTCCTGACCTTCTTAATATTATTTTTCATTTACTGC
AGCACCATTACCATTTGCTTAATTTTAATACAAGTCTTCTTAAAATGAAACCTAGTTAAAATGCTCTCCCTTCTATCTTGGTTGATACCCTTAGCTTTTTAAGAATTTGATTTCATCTTAAAGCTTTTGATTTGCTAAACT
CTACTATTATAGACTTTCTTATAGCTATTGAACAGTTTGAAAATTAGCTGTTTAATCATTTTGCATTTTATTTTCCAGATCTGTTTATGCCTTTGCATTTTCATTTTCTTCTTATTCTTTGTTTTTTTTCTCTACTGCTTC
CTTTCTTTGCTGAAGAATCCTAGCAAAGCTATTATCAACATTCTCGATAATTGGTCTACTCGAGGTTGGTACCCAATTCGCCCTATAGTGAGTCGTATTACAATTCACTGGCCGTCGTTTTACAACGTCGTGACTGGGAAA
ACCCTGGCGTTACCCAACTTAATCGCCTTGCAGCACATCCCCCTTTCGCCAGCTGGCGTAATAGCGAAGAGGCCCGCACCGATCGCCCTTCCCAACAGTTGCGCAGCCTGAATGGCGAATGGACGCGCCCTGTAGCGGCG
CATTAAGCGCGGCGGGTGTGGTGGTTACGCGCAGCGTGACCGCTACACTTGCCAGCGCCCTAGCGCCCGCTCCTTTCGCTTTCTTCCCTTCCTTTCTCGCCACGTTCGCCGGCTTTCCCCGTCAAGCTCTAAATCGGGGGC
TCCCTTTAGGGTTCCGATTTAGTGCTTTACGGCACCTCGACCCCAAAAAACTTGATTAGGGTGATGGTTCACGTAGTGGGCCATCGCCCTGATAGACGGTTTTTCGCCCTTTGACGTTGGAGTCCACGTTCTTTAATAGTG
GACTCTTGTTCCAAACTGGAACAACACTCAACCCTATCTCGGTCTATTCTTTTGATTTATAAGGGATTTTGCCGATTTCGGCCTATTGGTTAAAAAATGAGCTGATTTAACAAAAATTTAACGCGAATTTTAACAAAATAT
TAACGCTTACAATTTAGGTG

PDD1 5' flanking genomic sequence	2244..3700
PDD1 3’ UTR	3719..3985
loxP	3992..4025
neo5	4047..5502
loxP	5504..5537
PDD1 3' flanking genomic sequence	5544..6967
pBlueScriptSK(+)	6977..2234

Phosphor-mimic *PDD1* genes for in vivo analyses

```
>MIM10
ATGTCTCAGAAAAAGAGTTTAAAATAAAAGAGAAAGTAAGACTATAGCTCTGATGAAGAAGAGGAAGAAGAGGATCAATATGAAGTTGAAAAGATCTTAGACTCTAGATTTAATCCTAAGACCAAGCAAAAAGAATATCTT
GTCAAATGGGAAAGTATTATTAAATATTTATCTATTTATCATATTAATTCTTTTTATATATATATAAAGACTGGCCTATTGAAGACTCTACCTGGGAACCTTACGAGCATCTTTCGAATGTCAAAGAAATAGTTTAGGCCT
TTGAAAAGAAGCAAAAAGCTAACGTTATGCCTCAACCTACTGGACCTATCACTAGAGTAAATGCCTAGAAAGACCCTTAAAAAAAGAACAGACTATCTTTGAATTCTGAAATCAGCAAATCTTTACCTCAAGAAGAAGAAA
TTTAGACATCTAAAGAAGACAGTAAAAAATAAGCAGTCAAAAAATTCCAACCAGCTTCAAGAAGAAAATCTATTAGTTCTTAAGAAGATGAAGACCTTTAAGCTGAAGAAGTTCCACAGTAGCCTGAAAGTAAAAAAGATA
AAAATGATGGTGCCTTTGAAGAACCCAACAATGCTGATGCAGACGAAGAAGAGCTTGTATTTGAAGAAATTGTTGACAAAAGAATTCTTGATGGATAAACAGAATATTTAATACGCTTCTAAAGTAATTTATTTATTTATT
TGTTAATTTTAAATAAACAATTTTAATTAAAATATTTTTAATTTATTTATTTAATTAATTAATTAAAATAGATGTCTCTTAGCCATAATGGGTAGATGTTGGACAATTGATAGCTATTAAAGATGATGTTATAGCTTATGA
AGATAAAATTGCTGCTTAGAGTTAGCTCAATAAGACTAAGAACTTGAAAGAAAATGCTAAACATTCAGAATAACAATAGCAAAGCGAAAATAATAACTTAGAGACGGAAGAAGTTGAAGAAGATAAAGATGAGAAAAAAAG
AAGTTTACTTGCGAATTCAAAGAGACCCACTGCTAAAAGATCATAGCCATTCAATTAAGATGAAGAGAAAGAAAAAGAAGTCACTTAGCCTACCTCGAATTTAGGAGAAAAAGGTCAATCTTAGTAAGTTGAAAAAGAACA
AGCAACAAACTCACAAACTTAACAGCCTCAAACAGCACACAGAAGTGGCTCAAGACTCGAATAGATTCAATCAAATGCCAATCAAGTTACTTAATAAGCTTAATAGCTATCAAACACCAACAATTCTTCCTCTACATCTCT
TGAAGTTTCATCAAAAATGCCTAGCTAAATGTCTTAAAAACGCAGACCTATTGAACTTACTGAAATTTAACAAGGTGATTTCAAGACCGATAATGTCGATAAGATTGAAATCCAAGGAGATTTTAATGACATAATGACTTC
AAGGTTTGAAGTTTTTTGGAAAATCAGATAAGACAATGTTACCCCTGCTTCCTAAGTCTATTCAGCCTCTTACCTGAGAAGATATGAGCCTTAAGTTCTCATCGATTTCCTCTTACAACATTCAAACTAATCTCAATTAAG
ACAAGCTAATTAATAACTTACTCATTGA

Intron    155..210, 688..776
```

```
>MIM14
ATGTCTCAGAAAAAGAGTTTAAAATAAAAGAGAAAGTAAGACTATAGCGAAGATGAAGAAGAGGAAGAAGAGGATCAATATGAAGTTGAAAAGATCTTAGACTCTAGATTTAATCCTAAGACCAAGCAAAAAGAATATCTT
GTCAAATGGGAAAGTATTATTAAATATTTATCTATTTATCATATTAATTCTTTTTATATATATATAAAGACTGGCCTATTGAAGACTCTACCTGGGAACCTTACGAGCATCTTTCGAATGTCAAAGAAATAGTTTAGGCCT
TTGAAAAGAAGCAAAAAGCTAACGTTATGCCTCAACCTACTGGACCTATCACTAGAGTAAATGCCTAGAAAGACCCTTAAAAAAAGAACAGACTAGAATTGAATTCTGAAATCGAAAAATCTTTACCTCAAGAAGAAGAAA
TTTAGACATCTAAAGAAGACAGTAAAAAATAAGCAGTCAAAAAATTCCAACCAGCTTCAAGAAGAAAATCTATTAGTTCTTAAGAAGATGAAGACCTTTAAGCTGAAGAAGTTCCACAGTAGCCTGAAAGTAAAAAAGATA
AAAATGATGGTGCCTTTGAAGAACCCAACAATGCTGATGCAGACGAAGAAGAGCTTGTATTTGAAGAAATTGTTGACAAAAGAATTCTTGATGGATAAACAGAATATTTAATACGCTTCTAAAGTAATTTATTTATTTATT
TGTTAATTTTAAATAAACAATTTTAATTAAAATATTTTTAATTTATTTATTTAATTAATTAATTAAAATAGATGTCTCTTAGCCATAATGGGTAGATGTTGGACAATTGATAGCTATTAAAGATGATGTTATAGCTTATGA
AGATAAAATTGCTGCTTAGAGTTAGCTCAATAAGACTAAGAACTTGAAAGAAAATGCTAAACATTCAGAATAACAATAGCAAAGCGAAAATAATAACTTAGAGACGGAAGAAGTTGAAGAAGATAAAGATGAGAAAAAAAG
AAGTTTACTTGCGAATTCAAAGAGACCCACTGCTAAAAGATCATAGCCATTCAATTAAGATGAAGAGAAAGAAAAAGAAGTCACTTAGCCTACCGAAAATTTAGGAGAAAAAGGTCAATCTTAGTAAGTTGAAAAAGAACA
AGCAACAAACTCACAAACTTAACAGCCTCAAACAGCACACAGAAGTGGCTCAAGACTCGAATAGATTCAATCAAATGCCAATCAAGTTACTTAATAAGCTTAATAGCTATCAAACACCAACAATTCTTCCTCTACATCTCT
TGAAGTTTCATCAAAAATGCCTAGCTAAATGTCTTAAAAACGCAGACCTATTGAACTTACTGAAATTTAACAAGGTGATTTCAAGACCGATAATGTCGATAAGATTGAAATCCAAGGAGATTTTAATGACATAATGACTTC
AAGGTTTGAAGTTTTTTGGAAAATCAGATAAGACAATGTTACCCCTGCTTCCTAAGTCTATTCAGCCTCTTACCTGAGAAGATATGAGCCTTAAGTTCTCATCGATTTCCTCTTACAACATTCAAACTAATCTCAATTAAG
ACAAGCTAATTAATAACTTACTCATTGA

Intron    155..210, 688..776
```

```
>MIM18
ATGTCTCAGAAAAAGAGTTTAAAATAAAAGAGAAAGTAAGACTATGAAGAAGATGAAGAAGAGGAAGAAGAGGATCAATATGAAGTTGAAAAGATCTTAGACTCTAGATTTAATCCTAAGACCAAGCAAAAAGAATATCTT
GTCAAATGGGAAAGTATTATTAAATATTTATCTATTTATCATATTAATTCTTTTTATATATATATAAAGACTGGCCTATTGAAGACTCTACCTGGGAACCTTACGAGCATCTTTCGAATGTCAAAGAAATAGTTTAGGCCT
TTGAAAAGAAGCAAAAAGCTAACGTTATGCCTCAACCTACTGGACCTATCACTAGAGTAAATGCCTAGAAAGACCCTTAAAAAAAGAACAGACTAGAATTGAATGAGGAAATCGAAAAATCTTTACCTCAAGAAGAAGAAA
TTTAGACATCTAAAGAAGACAGTAAAAAATAAGCAGTCAAAAAATTCCAACCAGCTTCAAGAAGAAAATCTATTAGTGAATAAGAAGATGAAGACCTTTAAGCTGAAGAAGTTCCACAGTAGCCTGAAAGTAAAAAAGATA
AAAATGATGGTGCCTTTGAAGAACCCAACAATGCTGATGCAGACGAAGAAGAGCTTGTATTTGAAGAAATTGTTGACAAAAGAATTCTTGATGGATAAACAGAATATTTAATACGCTTCTAAAGTAATTTATTTATTTATT
TGTTAATTTTAAATAAACAATTTTAATTAAAATATTTTTAATTTATTTATTTAATTAATTAATTAAAATAGATGTCTCTTAGCCATAATGGGTAGATGTTGGACAATTGATAGCTATTAAAGATGATGTTATAGCTTATGA
AGATAAAATTGCTGCTTAGAGTTAGCTCAATAAGGAAAAGAACTTGAAAGAAAATGCTAAACATTCAGAATAACAATAGCAAAGCGAAAATAATAACTTAGAGACGGAAGAAGTTGAAGAAGATAAAGATGAGAAAAAAAG
AAGTTTACTTGCGAATTCAAAGAGACCCACTGCTAAAAGATCATAGCCATTCAATTAAGATGAAGAGAAAGAAAAAGAAGTCACTTAGCCTACCGAAAATTTAGGAGAAAAAGGTCAATCTTAGTAAGTTGAAAAAGAACA
AGCAACAAACTCACAAACTTAACAGCCTCAAACAGCACACAGAAGTGGCTCAAGACTCGAATAGATTCAATCAAATGCCAATCAAGTTACTTAATAAGCTTAATAGCTATCAAACACCAACAATTCTTCCTCTACATCTCT
TGAAGTTTCATCAAAAATGCCTAGCTAAATGTCTTAAAAACGCAGACCTATTGAACTTACTGAAATTTAACAAGGTGATTTCAAGACCGATAATGTCGATAAGATTGAAATCCAAGGAGATTTTAATGACATAATGACTTC
AAGGTTTGAAGTTTTTTGGAAAATCAGATAAGACAATGTTACCCCTGCTTCCTAAGTCTATTCAGCCTCTTACCTGAGAAGATATGAGCCTTAAGTTCTCATCGATTTCCTCTTACAACATTCAAACTAATCTCAATTAAG
ACAAGCTAATTAATAACTTACTCATTGA

Intron    155..210, 688..776
```

```
>MIM22
ATGTCTCAGAAAAAGAGTTTAAAATAAAAGAGAAAGTAAGACTATGAAGAAGATGAAGAAGAGGAAGAAGAGGATCAATATGAAGTTGAAAAGATCTTAGACTCTAGATTTAATCCTAAGACCAAGCAAAAAGAATATCTT
GTCAAATGGGAAAGTATTATTAAATATTTATCTATTTATCATATTAATTCTTTTTATATATATATAAAGACTGGCCTATTGAAGACTCTACCTGGGAACCTTACGAGCATCTTTCGAATGTCAAAGAAATAGTTTAGGCCT
TTGAAAAGAAGCAAAAAGCTAACGTTATGCCTCAACCTACTGGACCTATCACTAGAGTAAATGCCTAGAAAGACCCTTAAAAAAAGAACAGACTAGAATTGAATGAGGAAATCGAAAAAGAATTACCTCAAGAAGAAGAAA
TTTAGACATCTAAAGAAGACAGTAAAAAATAAGCAGTCAAAAAATTCCAACCAGCTTCAAGAAGAAAATCTATTGAAGAATAAGAAGATGAAGACCTTTAAGCTGAAGAAGTTCCACAGTAGCCTGAAAGTAAAAAAGATA
AAAATGATGGTGCCTTTGAAGAACCCAACAATGCTGATGCAGACGAAGAAGAGCTTGTATTTGAAGAAATTGTTGACAAAAGAATTCTTGATGGATAAACAGAATATTTAATACGCTTCTAAAGTAATTTATTTATTTATT
TGTTAATTTTAAATAAACAATTTTAATTAAAATATTTTTAATTTATTTATTTAATTAATTAATTAAAATAGATGTCTCTTAGCCATAATGGGTAGATGTTGGACAATTGATAGCTATTAAAGATGATGTTATAGCTTATGA
AGATAAAATTGCTGCTTAGAGTTAGCTCAATAAGGAAAAGAACTTGAAAGAAAATGCTAAACATTCAGAATAACAATAGCAAAGCGAAAATAATAACTTAGAGACGGAAGAAGTTGAAGAAGATAAAGATGAGAAAAAAAG
AAGTTTACTTGCGAATGAAAAGAGACCCACTGCTAAAAGATCATAGCCATTCAATTAAGATGAAGAGAAAGAAAAAGAAGTCACTTAGCCTACCGAAAATTTAGGAGAAAAAGGTCAATCTTAGTAAGTTGAAAAAGAACA
AGCAACAAACTCACAAACTTAACAGCCTCAAACAGCACACAGAAGTGGCGAAAGACTCGAATAGATTCAATCAAATGCCAATCAAGTTACTTAATAAGCTTAATAGCTATCAAACACCAACAATTCTTCCTCTACATCTCT
TGAAGTTTCATCAAAAATGCCTAGCTAAATGTCTTAAAAACGCAGACCTATTGAACTTACTGAAATTTAACAAGGTGATTTCAAGACCGATAATGTCGATAAGATTGAAATCCAAGGAGATTTTAATGACATAATGACTTC
AAGGTTTGAAGTTTTTTGGAAAATCAGATAAGACAATGTTACCCCTGCTTCCTAAGTCTATTCAGCCTCTTACCTGAGAAGATATGAGCCTTAAGTTCTCATCGATTTCCTCTTACAACATTCAAACTAATCTCAATTAAG
ACAAGCTAATTAATAACTTACTCATTGA

Intron    155..210, 688..776
```

```
>MIM22+Ins6K
ATGTCTCAGAAAAAGAGTTTAAAATAAAAGAGAAAGTAAGACTATGAAGAAGATGAAGAAGAGGAAGAAGAGGATCAATATGAAGTTGAAAAGATCTTAGACTCTAGATTTAATCCTAAGACCAAGCAAAAAGAATATCTT
GTCAAATGGGAAAGTATTATTAAATATTTATCTATTTATCATATTAATTCTTTTTATATATATATAAAGACTGGCCTATTGAAGACTCTACCTGGGAACCTTACGAGCATCTTTCGAATGTCAAAGAAATAGTTTAGGCCT
TTGAAAAGAAGCAAAAAGCTAACGTTATGCCTCAACCTACTGGACCTATCACTAGAGTAAATGCCTAGAAAGACCCTTAAAAAAAGAACAGACTAGAATTGAATGAGGAAATCGAAAAAAAGGAATTACCTCAAGAAGAAG
AAATTTAGACATCTAAAGAAGACAGTAAAAAATAAGCAGTCAAAAAATTCCAACCAGCTTCAAGAAGAAAATCTATTGAAAAGGAATAAGAAGATGAAGACCTTTAAGCTGAAGAAGTTCCACAGTAGCCTGAAAGTAAAA
AAGATAAAAATGATGGTGCCTTTGAAGAACCCAACAATGCTGATGCAGACGAAGAAGAGCTTGTATTTGAAGAAATTGTTGACAAAAGAATTCTTGATGGATAAACAGAATATTTAATACGCTTCTAAAGTAATTTATTTA
TTTATTTGTTAATTTTAAATAAACAATTTTAATTAAAATATTTTTAATTTATTTATTTAATTAATTAATTAAAATAGATGTCTCTTAGCCATAATGGGTAGATGTTGGACAATTGATAGCTATTAAAGATGATGTTATAGC
TTATGAAGATAAAATTGCTGCTTAGAGTTAGCTCAATAAGGAAAAGAAGAACTTGAAAGAAAATGCTAAACATTCAGAATAACAATAGCAAAGCGAAAATAATAACTTAGAGACGGAAGAAGTTGAAGAAGATAAAGATGA
GAAAAAAAGAAGTTTACTTGCGAATAAGGAAAAGAGACCCACTGCTAAAAGATCATAGCCATTCAATTAAGATGAAGAGAAAGAAAAAGAAGTCACTTAGCCTACCGAAAAGAATTTAGGAGAAAAAGGTCAATCTTAGTA
AGTTGAAAAAGAACAAGCAACAAACTCACAAACTTAACAGCCTCAAACAGCACACAGAAGTGGCGAAAAGAGACTCGAATAGATTCAATCAAATGCCAATCAAGTTACTTAATAAGCTTAATAGCTATCAAACACCAACAA
TTCTTCCTCTACATCTCTTGAAGTTTCATCAAAAATGCCTAGCTAAATGTCTTAAAAACGCAGACCTATTGAACTTACTGAAATTTAACAAGGTGATTTCAAGACCGATAATGTCGATAAGATTGAAATCCAAGGAGATTT
TAATGACATAATGACTTCAAGGTTTGAAGTTTTTTGGAAAATCAGATAAGACAATGTTACCCCTGCTTCCTAAGTCTATTCAGCCTCTTACCTGAGAAGATATGAGCCTTAAGTTCTCATCGATTTCCTCTTACAACATTC
AAACTAATCTCAATTAAGACAAGCTAATTAATAACTTACTCATTGA

Intron    155..210, 694..782
```

```
>MIM22+Sub6K
ATGTCTCAGAAAAAGAGTTTAAAATAAAAGAGAAAGTAAGACTATGAAGAAGATGAAGAAGAGGAAGAAGAGGATCAATATGAAGTTGAAAAGATCTTAGACTCTAGATTTAATCCTAAGACCAAGCAAAAAGAATATCTT
GTCAAATGGGAAAGTATTATTAAATATTTATCTATTTATCATATTAATTCTTTTTATATATATATAAAGACTGGCCTATTGAAGACTCTACCTGGGAACCTTACGAGCATCTTTCGAATGTCAAAGAAATAGTTTAGGCCT
```

TTGAAAAGAAGCAAAAAGCTAACGTTATGCCTCAACCTACTGGACCTATCACTAGAGTAAATGCCTAGAAAGACCCTTAAAAAAAGAACAGACTAGAATTGAATGAGGAAATCGAAAAAGAATTACCTAAAGAAGAAGAAA
TTTAGACATCTAAAGAAGACAGTAAAAAATAAGCAGTCAAAAAATTCCAACCAGCTTCAAGAAGAAAATCTATTGAAGAAAAAGAAGATGAAGACCTTTAAGCTGAAGAAGTTCCACAGTAGCCTGAAAGTAAAAAAGATA
AAAATGATGGTGCCTTTGAAGAACCCAACAATGCTGATGCAGACGAAGAAGAGCTTGTATTTGAAGAAATTGTTGACAAAAGAATTCTTGATGGATAAACAGAATATTTAATACGCTTCTAAAGTAATTTATTTATTTATT
TGTTAATTTTAAATAAACAATTTTAATTAAAATATTTTTAATTTATTTATTTAATTAATTAATTAAAATAGATGTCTCTTAGCCATAATGGGTAGATGTTGGACAATTGATAGCTATTAAAGATGATGTTATAGCTTATGA
AGATAAAATTGCTGCTTAGAGTTAGCTCAAGAAGGAAAAGAACTTGAAAGAAAATGCTAAACATTCAGAATAACAATAGCAAAGCGAAAATAATAACTTAGAGACGGAAGAAGTTGAAGAAGATAAAGATGAGAAAAAAAG
AAGTTTACTTGCGAAGGAAAAGAGACCCACTGCTAAAAGATCATAGCCATTCAATTAAGATGAAGAGAAAGAAAAAGAAGTCACTTAGCCTACCGAAAAGTTAGGAGAAAAAGGTCAATCTTAGTAAGTTGAAAAAGAACA
AGCAACAAACTCACAAACTTAACAGCCTCAAACAGCACACAGAAGTGGCGAAAGACTCGAAAAGATTCAATCAAATGCCAATCAAGTTACTTAATAAGCTTAATAGCTATCAAACACCAACAATTCTTCCTCTACATCTCT
TGAAGTTTCATCAAAAATGCCTAGCTAAATGTCTTAAAAACGCAGACCTATTGAACTTACTGAAATTTAACAAGGTGATTTCAAGACCGATAATGTCGATAAGATTGAAATCCAAGGAGATTTTAATGACATAATGACTTC
AAGGTTTGAAGTTTTTTGGAAAATCAGATAAGACAATGTTACCCCTGCTTCCTAAGTCTATTCAGCCTCTTACCTGAGAAGATATGAGCCTTAAGTTCTCATCGATTTCCTCTTACAACATTCAAACTAATCTCAATTAAG
ACAAGCTAATTAATAACTTACTCATTGA

Intron   155..210, 688..776

Codon optimized *PDD1* genes for *E. coli* expression

>opt.WT_FL
ATGAGCCAAAAAAAATCCCTGAAACAAAAACGTAAACAAGACTATAGCAGCGACACCGAGGAAGAGGAGGAGGACCAATATGAAGTGGAAAAAATCCTGGATAGCCGCTTTAATCCGAAAACCAAACAAAAAGAGTATCTG
GTGAAATGGGAGAACTGGCCGATCGAAGATTCAACCTGGGAACCGTATGAACATCTGAGCAACGTGAAAGAAATCGTACAGGCCTTTGAGAAAAAACAGAAAGCCAACGTGATGCCTCAACCAACAGGTCCGATTACCCGT
GTTAATGCCCAGAAAGACCCACAGAAAAAAAACCGTCTGAGCCTGAATAGCAGTATCAGCAAATCTCTGCCACAAGAAGAGGAAATCCAAACCAGCAAAGAGGATAGCAAAAAACAAGCCGTCAAAAAATTCCAGCCGGCA
AGTCGTCGTAAAAGCATCTCTAGTCAGAGCGATAGTGATCTGCAAGCAGAAGAAGTGCCTCAGCAGCCAGAGTCTAAAAAAGACAAAAATGATGGCGCCTTTGAAGAACCGAATAACGCCGATGCCGATGAAACGGAACTG
GTGTTCGAGGAGATTGTGGATAAACGTATTCTGGATGGCCAGACCGAATATCTGATCCGCTTTCAGAACGTTAGTCAGCCACAGTGGGTTGATGTTGGCCAGCTGATTGCCATCAAAGATGACGTGATCGCCTATGAGGAC
AAAATCGCCGCCCAAAGTCAACTGAACAAAACGAAAAACCTGAAAGAGAACGCCAAACATTCTAGTCAACAGCAGCAGTCCGAGAACAATAATCTGGAGACTGAAGAGGTCGAAGAGGACAAAGACAGCAAAAAACGCTCC
CTGCTGGCAAATTCTAAACGCCCGACGGCGAAACGCTCTCAACCGTTCAACCAGGACGAGGAAAAAGAGAAATCCGTCACCCAGCCTACAAGCAACCTGGGATCGAAAGGTCAGAGTCAGCAAGTGGAGAAAGAACAAGCG
ACCAATAGCCAGACACAACAGCCACAGACAGCACATCGTAGCGGTAGTCGTCTGAGTCAAATCCAATCGAATGCTAACCAAGTGACACAACAGGCCCAACAACTGAGTAATACCAACAACAGCTCCAGCACTAGTCTGGAA
GTGTCCAGTAAAATGCCGTCACAGATGAGTCAGAAACGTCGCCCGATTGAACTGACTGAAATTCAACAGGGGGACTTTAAAACCGACAACGTGGATAAAATCGAGATCCAAGGCGACTTCAACGATATCATGACCTCCCGC
TTCGAAGTGTTTTGGAAAATCCGCCAGGACAATGTTACACCGGCAAGCCAGGTGTATTCTGCCTCTTATCTGCGTCGCTATGAACCACAAGTCCTGATCGACTTTCTGCTGCAACACTCCAATCAGTCACAGCTGCGCCAG
GCAAATCAGCAACTGACCCATTGA

>opt.W50/53A_FL
ATGAGCCAAAAAAAATCCCTGAAACAAAAACGTAAACAAGACTATAGCAGCGACACCGAGGAAGAGGAGGAGGACCAATATGAAGTGGAAAAAATCCTGGATAGCCGCTTTAATCCGAAAACCAAACAAAAAGAGTATCTG
GTGAAAGCCGAGAACGCCCCGATCGAAGATTCAACCTGGGAACCGTATGAACATCTGAGCAACGTGAAAGAAATCGTACAGGCCTTTGAGAAAAAACAGAAAGCCAACGTGATGCCTCAACCAACAGGTCCGATTACCCGT
GTTAATGCCCAGAAAGACCCACAGAAAAAAAACCGTCTGAGCCTGAATAGCAGTATCAGCAAATCTCTGCCACAAGAAGAGGAAATCCAAACCAGCAAAGAGGATAGCAAAAAACAAGCCGTCAAAAAATTCCAGCCGGCA
AGTCGTCGTAAAAGCATCTCTAGTCAGAGCGATAGTGATCTGCAAGCAGAAGAAGTGCCTCAGCAGCCAGAGTCTAAAAAAGACAAAAATGATGGCGCCTTTGAAGAACCGAATAACGCCGATGCCGATGAAACGGAACTG
GTGTTCGAGGAGATTGTGGATAAACGTATTCTGGATGGCCAGACCGAATATCTGATCCGCTTTCAGAACGTTAGTCAGCCACAGTGGGTTGATGTTGGCCAGCTGATTGCCATCAAAGATGACGTGATCGCCTATGAGGAC
AAAATCGCCGCCCAAAGTCAACTGAACAAAACGAAAAACCTGAAAGAGAACGCCAAACATTCTAGTCAACAGCAGCAGTCCGAGAACAATAATCTGGAGACTGAAGAGGTCGAAGAGGACAAAGACAGCAAAAAACGCTCC
CTGCTGGCAAATTCTAAACGCCCGACGGCGAAACGCTCTCAACCGTTCAACCAGGACGAGGAAAAAGAGAAATCCGTCACCCAGCCTACAAGCAACCTGGGATCGAAAGGTCAGAGTCAGCAAGTGGAGAAAGAACAAGCG
ACCAATAGCCAGACACAACAGCCACAGACAGCACATCGTAGCGGTAGTCGTCTGAGTCAAATCCAATCGAATGCTAACCAAGTGACACAACAGGCCCAACAACTGAGTAATACCAACAACAGCTCCAGCACTAGTCTGGAA
GTGTCCAGTAAAATGCCGTCACAGATGAGTCAGAAACGTCGCCCGATTGAACTGACTGAAATTCAACAGGGGGACTTTAAAACCGACAACGTGGATAAAATCGAGATCCAAGGCGACTTCAACGATATCATGACCTCCCGC
TTCGAAGTGTTTTGGAAAATCCGCCAGGACAATGTTACACCGGCAAGCCAGGTGTATTCTGCCTCTTATCTGCGTCGCTATGAACCACAAGTCCTGATCGACTTTCTGCTGCAACACTCCAATCAGTCACAGCTGCGCCAG
GCAAATCAGCAACTGACCCATTGA

>opt.I456D_FL
ATGAGCCAAAAAAAATCCCTGAAACAAAAACGTAAACAAGACTATAGCAGCGACACCGAGGAAGAGGAGGAGGACCAATATGAAGTGGAAAAAATCCTGGATAGCCGCTTTAATCCGAAAACCAAACAAAAAGAGTATCTG
GTGAAATGGGAGAACTGGCCGATCGAAGATTCAACCTGGGAACCGTATGAACATCTGAGCAACGTGAAAGAAATCGTACAGGCCTTTGAGAAAAAACAGAAAGCCAACGTGATGCCTCAACCAACAGGTCCGATTACCCGT
GTTAATGCCCAGAAAGACCCACAGAAAAAAAACCGTCTGAGCCTGAATAGCAGTATCAGCAAATCTCTGCCACAAGAAGAGGAAATCCAAACCAGCAAAGAGGATAGCAAAAAACAAGCCGTCAAAAAATTCCAGCCGGCA
AGTCGTCGTAAAAGCATCTCTAGTCAGAGCGATAGTGATCTGCAAGCAGAAGAAGTGCCTCAGCAGCCAGAGTCTAAAAAAGACAAAAATGATGGCGCCTTTGAAGAACCGAATAACGCCGATGCCGATGAAACGGAACTG
GTGTTCGAGGAGATTGTGGATAAACGTATTCTGGATGGCCAGACCGAATATCTGATCCGCTTTCAGAACGTTAGTCAGCCACAGTGGGTTGATGTTGGCCAGCTGATTGCCATCAAAGATGACGTGATCGCCTATGAGGAC
AAAATCGCCGCCCAAAGTCAACTGAACAAAACGAAAAACCTGAAAGAGAACGCCAAACATTCTAGTCAACAGCAGCAGTCCGAGAACAATAATCTGGAGACTGAAGAGGTCGAAGAGGACAAAGACAGCAAAAAACGCTCC
CTGCTGGCAAATTCTAAACGCCCGACGGCGAAACGCTCTCAACCGTTCAACCAGGACGAGGAAAAAGAGAAATCCGTCACCCAGCCTACAAGCAACCTGGGATCGAAAGGTCAGAGTCAGCAAGTGGAGAAAGAACAAGCG
ACCAATAGCCAGACACAACAGCCACAGACAGCACATCGTAGCGGTAGTCGTCTGAGTCAAATCCAATCGAATGCTAACCAAGTGACACAACAGGCCCAACAACTGAGTAATACCAACAACAGCTCCAGCACTAGTCTGGAA
GTGTCCAGTAAAATGCCGTCACAGATGAGTCAGAAACGTCGCCCGATTGAACTGACTGAAATTCAACAGGGGGACTTTAAAACCGACAACGTGGATAAAATCGAGATCCAAGGCGACTTCAACGATATCATGACCTCCCGC
TTCGAAGTGTTTTGGAAAATCCGCCAGGACAATGTTACACCGGCAAGCCAGGTGTATTCTGCCTCTTATCTGCGTCGCTATGAACCACAAGTCCTGGACGACTTTCTGCTGCAACACTCCAATCAGTCACAGCTGCGCCAG
GCAAATCAGCAACTGACCCATTGA

>opt.MIM14_FL
ATGTCTCAAAAAAAAAGCCTGAAGCAAAAACGCAAACAGGATTATAGCGAAGATGAGGAAGAGGAAGAAGAAGACCAGTATGAAGTGGAGAAAATCCTCGATTCACGGTTTAACCCCAAAACAAAACAGAAAGAGTATCTG
GTGAAGTGGGAAAACTGGCCCATTGAGGATAGCACATGGGAGCCATACGAACATTTATCTAATGTGAAGGAGATTGTGCAGGCGTTCGAAAAAAAACAAAAAGCGAATGTTATGCCCCAGCCCACGGGTCCGATTACGCGC
GTTAATGCGCAAAAGGACCCACAGAAGAAGAATCGCCTCGAACTCAACTCGGAGATTGAAAAATCACTCCCCCAAGAAGAAGAAATTCAGACGAGTAAAGAGGATTCTAAAAAACAGGCCGTCAAAAAGTTCCAGCCGGCT
TCGCGGCGCAAGAGTATTAGCTCCCAGGAAGATGAGGACCTGCAAGCAGAAGAAGTTCCGCAGCAGCCGGAAAGTAAAAAGGATAAAAATGATGGCGCATTTGAAGAACCTAACAACGCTGATGCAGACGAAGAGGAACTG
GTGTTCGAGGAAATTGTGGATAAACGCATTTTGGACGGTCAGACTGAGTACTTGATCCGTTTTCAGAATGTCAGCCAACCTCAGTGGGTTGACGTTGGTCAGCTGATTGCTATCAAAGACGATGTGATCGCGTACGAAGAT
AAAATCGCCGCACAGTCACAGCTGAATAAAACAAAAAACTTGAAGGAAAACGCTAAACATTCGGAGCAGCAGCAGCAGTCCGAAAACAACAATTTGGAAACTGAAGAAGTGGAGGAAGACAAAGATGAAAAGAAGCGCAGT
CTGCTGGCGAATAGCAAACGTCCGACTGCGAAGCGTTCACAACCGTTCAATCAGGATGAAGAAAAAGAAAAAGAAGTGACCCAGCCAACCGAAAATCTCGGCGAAAAAGGCCAATCGCAGCAGGTGGAAAAAGAACAGGCC
ACGAATAGCCAGACTCAGCAGCCGCAGACCGCCCATCGCAGTGGCTCGCGCCTTGAACAGATCCAATCGAACGCGAATCAGGTAACGCAGCAGGCCCAGCAGTTAAGCAACACCAATAATAGCTCTTCTACCAGCCTGGAG
GTTAGCTCAAAAATGCCTAGTCAGATGTCTCAGAAACGCCGCCCGATCGAACTGACCGAGATTCAGCAGGGGGATTTCAAGACGGACAATGTGGACAAAATTGAGATTCAGGGTGATTTCAATGATATCATGACGTCTCGC
TTTGAAGTTTTTTGGAAAATTCGCCAGGATAATGTAACGCCAGCGAGCCAAGTGTACAGTGCCAGCTACCTGCGCCGTTACGAACCTCAGGTCCTGATCGATTTCCTGTTACAACACTCTAATCAGTCTCAGCTGCGCCAG
GCGAATCAGCAACTTACGCACTGA

>opt.MIM22_FL
ATGTCTCAAAAAAAAAGCCTGAAACAAAAACGCAAGCAAGATTATGAAGAAGATGAAGAGGAAGAAGAGGAAGACCAGTATGAGGTGGAAAAAATCCTGGATAGTCGTTTCAATCCGAAAACAAAGCAGAAAGAATATCTT
GTGAAATGGGAAAATTGGCCCATCGAAGATAGCACGTGGGAACCTTACGAACATCTGTCGAATGTTAAAGAAATCGTCCAGGCTTTTGAAAAAAAACAGAAAGCCAATGTGATGCCTCAGCCTACCGGACCGATCACACGT
GTGAACGCGCAGAAAGATCCTCAGAAGAAAAATCGCCTGGAGCTGAATGAAGAAATCGAGAAAGAACTGCCGCAGGAGGAGGAAATTCAGACCAGCAAAGAAGATTCGAAAAAGCAGGCTGTGAAAAAGTTTCAACCGGCA
AGCCGTCGCAAAAGTATTGAAGAACAGGAAGATGAGGATTTGCAGGCGGAGGAAGTGCCCCAGCAACCTGAATCCAAAAAAGATAAAAACGATGGAGCGTTTGAAGAGCCAAACAATGCGGACGCAGACGAAGAGGAACTC
GTGTTCGAGGAGATTGTGGACAAACGCATTTTAGATGGTCAGACCGAATACCTCATTCGTTTTCAGAACGTTAGTCAGCCGCAATGGGTAGACGTAGGGCAGTTAATTGCCATTAAAGATGACGTGATCGCGTATGAAGAT
AAAATTGCCGCCCAAAGTCAGCTGAACAAGGAAAAAAACTTAAAAGAGAATGCTAAGCACAGCGAACAACAACAACAAAGCGAAAATAACAACCTCGAAACAGAGGAAGTGGAAGAAGATAAAGATGAAAAAAAACGCTCC
TTACTGGCAAACGAAAAACGCCCGACCGCGAAACGCTCCCAGCCCTTTAATCAGGACGAAGAGAAGGAGAAGGAAGTCACACAGCCCACGGAGAATTTAGGCGAAAAAGGTCAGAGCCAGCAGGTAGAAAAAGAACAGGCT
ACCAATTCTCAGACGCAGCAGCCGCAAACCGCCCACCGTTCCGGCGAACGTCTGGAGCAGATCCAGAGCAACGCTAATCAAGTTACGCAGCAGGCGCAGCAACTGTCTAATACCAACAATAGCAGCAGTACTTCGCTCGAA
GTGTCCAGCAAGATGCCGTCCCAGATGTCTCAAAAACGTCGTCCAATTGAACTTACGGAGATCCAACAGGGTGACTTTAAGACGGACAATGTGGATAAGATTGAAATTCAGGGGGATTTTAATGATATCATGACCTCTCGT
TTCGAGGTTTTTTGGAAAATCCGCCAAGACAATGTCACCCCCGCGTCGCAGGTTTACTCAGCCTCGTATCTTCGCCGTTACGAACCGCAAGTGCTTATTGATTTTCTGCTCCAGCACTCAAATCAATCGCAGCTTCGCCAA
GCCAACCAGCAGTTGACGCACTGA

>opt.MIM22+Ins6K_FL
ATGTCCCAAAAGAAATCGTTAAAGCAGAAACGTAAGCAGGATTATGAGGAAGATGAAGAAGAAGAAGAAGAGGACCAGTACGAAGTCGAGAAGATCCTGGATTCCCGCTTCAACCCGAAAACTAAGCAAAAAGAGTACCTG
GTTAAATGGGAAAACTGGCCAATTGAGGATTCGACTTGGGAACCGTATGAACATTTGTCAAATGTGAAAGAGATTGTACAGGCGTTTGAGAAAAAACAAAAAGCGAACGTTATGCCGCAGCCGACGGGCCCCATTACTCGT
GTAAATGCGCAGAAAGATCCACAGAAGAAAAATCGCCTGGAGCTGAATGAAGAGATCGAAAAAAAAGAATTACCTCAGGAAGAAGAAATTCAAACGAGCAAGGAAGACTCAAAAAAACAGGCCGTGAAGAAATTTCAGCCT
GCGAGCCGCCGTAAAAGTATCGAAAAAGAACAAGAAGATGAAGACCTGCAAGCCGAGGAAGTTCCGCAACAGCCGGAATCCAAGAAAGATAAAAATGACGGCGCCTTTGAAGAACCAAATAATGCCGATGCGGATGAGGAG
GAACTTGTGTTCGAAGAAATCGTTGATAAACGTATCCTGGACGGGCAAACGGAATATCTGATCCGCTTTCAGAATGTCTCTCAACCGCAGTGGGTGGACGTTGGTCAATTGATTGCCATTAAAGACGATGTCATTGCGTAT
GAGGATAAAATCGCCGCGCAATCGCAGCTGAACAAAGAAAAGAAGAACCTGAAGGAAAACGCTAAACACTCTGAACAACAGCAGCAGTCAGAGAATAACAATCTCGAAACCGAGGAAGTAGAAGAAGATAAAGATGAAAAA
AAACGGTCGCTCCTGGCTAATAAAGAAAAGCGCCCGACAGCAAAACGGAGCCAGCCTTTCAACCAGGATGAAGAAAAAGAAAAAGAAGTGACGCAGCCGACAGAAAAAAACCTGGGCGAAAAAGGACAATCGCAGCAGGTG
GAGAAAGAGCAGGCGACGAACAGTCAAACTCAGCAGCCTCAGACCGCACACCGCAGTGGAGAGAAGCGTCTGGAACAAATCCAAAGTAATGCAAATCAAGTGACCCAACAGGCTCAACAACTGAGCAATACGAACAATAGC
AGCTCCACCTCGTTGGAGGTCAGCTCAAAGATGCCTAGCCAGATGAGCCAAAAGCGTCGCCCGATTGAGCTGACCGAAATTCAGCAGGGTGATTTTAAGACCGACAATGTAGACAAGATCGAAATTCAGGGAGATTTTAAC
GATATTATGACTTCACGGTTCGAGGTCTTCTGGAAAATCCGTCAAGATAATGTAACCCCGGCCTCTCAGGTTTATTCGGCCAGCTATCTTCGTCGTTATGAACCCCAGGTCCTCATCGATTTTCTGCTCCAGCACTCGAAT
CAGTCTCAACTTCGCCAGGCCAACCAGCAGCTTACCCATTGA

>opt. WT_HNG1
AAAAAACAGAAAGCCAACGTGATGCCTCAACCAACAGGTCCGATTACCCGTGTTAATGCCCAGAAAGACCCACAGAAAAAAAACCGTCTGAGCCTGAATAGCAGTATCAGCAAATCTCTGCCACAAGAAGAGGAAATCCAA
ACCAGCAAAGAGGATAGCAAAAAACAAGCCGTCAAAAAATTCCAGCCGGCAAGTCGTCGTAAAAGCATCTCTAGTCAGAGCGATAGTGATCTGCAAGCAGAAGAAGTGCCTCAGCAGCCAGAGTCTAAAAAAGACAAAAAT
GATGGCGCCTTTGAAGAACCGAATAACGCCGATGCCGATGAAACGGAACTGTGA

>opt.WT_HNG2
ATCAAAGATGACGTGATCGCCTATGAGGACAAAATCGCCGCCCAAAGTCAACTGAACAAAACGAAAAACCTGAAAGAGAACGCCAAACATTCTAGTCAACAGCAGCAGTCCGAGAACAATAATCTGGAGACTGAAGAGGTC
GAAGAGGACAAAGACAGCAAAAAACGCTCCCTGCTGGCAAATTCTAAACGCCCGACGGCGAAACGCTCTCAACCGTTCAACCAGGACGAGGAAAAAGAGAAATCCGTCACCCAGCCTACAAGCAACCTGGGATCGAAAGGT
CAGAGTCAGCAAGTGGAGAAAGAACAAGCGACCAATAGCCAGACACAACAGCCACAGACAGCACATCGTAGCGGTAGTCGTCTGAGTCAAATCCAATCGAATGCTAACCAAGTGACACAACAGGCCCAACAACTGAGTAAT
ACCAACAACAGCTCCAGCACTAGTCTGGAAGTGTCCAGTAAAATGCCGTCACAGATGAGTCAGAAACGTCGCCCGATTGAACTGACTGAAATTTGA

A map and the sequence of pCAM_MDS-IES

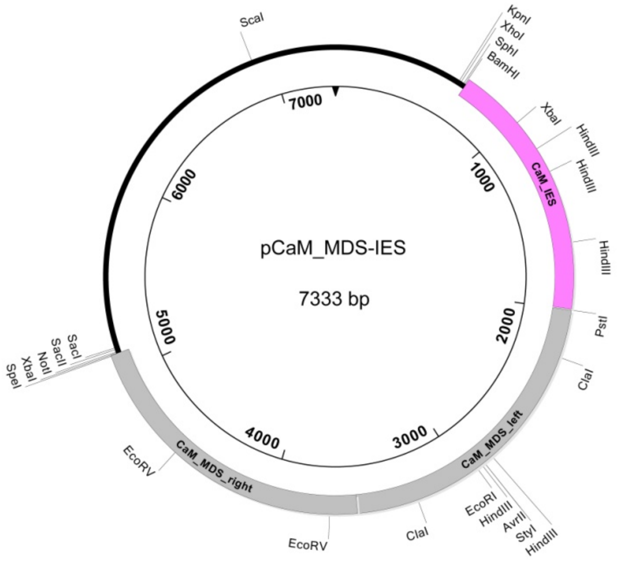


```
> pCAM_MDS-IES
CACCTAAATTGTAAGCGTTAATATTTTGTTAAAATTCGCGTTAAATTTTTGTTAAATCAGCTCATTTTTTAACCAATAGGCCGAAATCGGCAAAATCCCTTATAAATCAAAAGAATAGACCGAGATAGGGTTGAGTGTTGT
TCCAGTTTGGAACAAGAGTCCACTATTAAAGAACGTGGACTCCAACGTCAAAGGGCGAAAAACCGTCTATCAGGGCGATGGCCCACTACGTGAACCATCACCCTAATCAAGTTTTTTGGGGTCGAGGTGCCGTAAAGCACT
AAATCGGAACCCTAAAGGGAGCCCCCGATTTAGAGCTTGACGGGGAAAGCCGGCGAACGTGGCGAGAAAGGAAGGGAAGAAAGCGAAAGGAGCGGGCGCTAGGGCGCTGGCAAGTGTAGCGGTCACGCTGCGCGTAACCAC
CACACCCGCCGCGCTTAATGCGCCGCTACAGGGCGCGTCCCATTCGCCATTCAGGCTGCGCAACTGTTGGGAAGGGCGATCGGTGCGGGCCTCTTCGCTATTACGCCAGCTGGCGAAAGGGGGATGTGCTGCAAGGCGATT
AAGTTGGGTAACGCCAGGGTTTTCCCAGTCACGACGTTGTAAAACGACGGCCAGTGAATTGTAATACGACTCACTATAGGGCGAATTGGGTACCGGGCCCCCCCTCGAGACGACGCATGCCTACAGGATCCAATTTACAAA
AGTTTGAATAATGAATTTTAGCACATTTAAATATTATTATATCAGTAAATAAAATATTCTTAAATTCTATAAAATATTTCTTATATATTTTTAAATATTACCAAATCAAAAACTAATATTTTTTAACAATTCAAATTAAAA
AAAAAATTAATTGTTTTAATTTTATGATTTACTAAATTTCAATTTTTAATTTCTTAACCTTAGATGTTTTAAAATAAGATAGATTTCTTCATTAAAAAAATTTGATTAATTTACTAGGCACTAAAATTTTTTTATTTTTAAA
AAAACTATTTTTAAAATAGATTTTTCTAGATTTAAATTACAAATAAAGATAATTTTTTTAATCACAAATGTTCAATTAATAGAGTATTTTTATGAACTTAATTTAATTTTATTATAGCACTTTGTAATTTAAAAAAATAGT
CATCGCTATTTATTAGCTATAATTTATTTTCTGATATATTTTGAAGCTTTAAATAAAAAATAGTATGTCATCAAAATATTTATAAATTTTGCCGAAAAATAAAAAACATTGATCTAAATTTTCAAAATTTATGGTTTTCCA
GAATTTTCAATCTAAAAAATTCTGGAAATTAAGCTTCATTAATAATTGAATGAAAACATAAATTTTATGTTTAATTAAATTTTTCTTTTTAAATATAGGAAATGCTTCCAAATTTTATACTTTGAGTAGAGTTTTTGTAAT
TTTTATTAATTTTTTTGAATCCATGCACTATTAAAAATCTAAATAAATTATCATTATTTTTTTTTTTTTTTTTCTTTTTATAACTCTATTTTAAATTAATATTAAATTTTATTCTTCTTATTGATAAAATTATTTGATTGAT
AGTTCATTATCAGCCATCAAGCATTTTTAAAATAGCCAATTGCTTTAATTTTATAATTTTAAAAACATATTATTAAAAATAGTATGTATTTCTTTTTACAGTTAAGCATACTAAGCTTTATTTTAATATTAAATTTGTAAG
CAATCCTATTTAGAGAACAGTTAGTTGATAAAAATCAGTTAGGCTTTAGTTTTAAATTATTTAAGAAACATTTGCATTATAAAAACTTCTTTAAAAATTTTATTTTTAACCAAAATTTTTTTTTATGAAATGAAATATTTA
TAAAATCTTAATTTAATTAAATTCAATTTTGCATATTTTGATTAAATGAGATATTTATATGTTGTATAGCTTTATATTATGTGTTTTAACTGTATCAATCAATAAATAAATGAATTAAATTGAAATAACTTGAATCAAATT
GAAAATAAATCTTAAAATAACTGCAGCGAAGTTAGAGCGAACCTCATAGGTGACGGATTTTAGAAGACCCCAAAAAGAAACGGATGTAATACCAATTGATTGATTGATTGATTGATTGTTTGATTTATTTATTTATTTATT
TTAAATATATTTAAAAATCATAATGAATAGATGTTAGAATGAATGTTATTGGAGAGATTTCTCAAGCTATTAACTTTGACAATGATAACCTGTATGTATAAATTAATGATAAATTAACAGGATCGATCAGCTATATTTTAT
TTATTTATCCACTATAGTTATATTGAATGGGAGTTTTTTTTACCTCCTCACTGGTAGCCAGATTATGAGGATTTGCAAGAAAATATGCCAATTTTATCAGGACACATTAGCGAAGATATGAAAAGGGATTGGAATTTTTTG
TAAATATCTAAATTTAATTAATTAGCTATTTCATAATTGTTAAATATATTTAATTAGCTCATCCATAACCCAAATAGCTTCTCCTATTTAATTAAAAAACAAAGATGGTTAAAACGAACTCATAACTAACTTCTGCTTCCC
ATTTGATATTTAGGTTTAAACTAAACAATAAACATGTAAATCTATCTTATTAATTATTTTAGAGTATTAAAGAAATTAATTATATTTATTCAAATATTAGTTGAAGAATTATCCAAATTCCCTATATTATTCCTTACATTA
AGTTCAATAGATTCATGGGGTAGAAAAACCCTTTAAGGTAAATATATGAATTATTCTAATTGCATTTATCAATGTTATTTTTTCAATTATCATTAAGGATACACATATTGTGAAGTTCCAAGAAAACCAGGATACCATGAA
ATAACTGTTAAAGCTTGGGCTCCTGCTTAAAGTATTTACTAAAAAGTACATGAGTTTTTCCTAGGAGGACAAACTAAGCTTTATGATATAAAAAAAATAGCTTAGAATTCAATTTTAAATGATGAAGTAAATAAATATCTT
TTAGATAATTAAAAAGCTCTAAATAAATATTAAATACTAACTATTAACAGAATACATAAAGCGCTATTGATAGATTTAATCTAACTACTGAGAGTAAAGGAGCTGTTAAATTAATATTTAATGTTGCATTTTAGTCAAAGT
AATTAATTATAAATGATGTCAAAAACAAATATTTAGTTAGTTATTAAATCATGTTTAAATTAGAAAACGTAAAGAAGAATAAACTGCCATTTACCATGCAAAAAGATTAAATCTCAAAAGTAATAATAAAAGTTAATAGA
TCCATCTGAAATCGATTAGCAAGGATAACAAGATAACTCTTATGCACCTGTTATAAAGTAATATTTTTTATCAAGTTAATATATTTTAATTACAATAATTAAAAAGAAACGAGTATACAACTTCACTAAGATATACTACGT
AGAAATTAAATTGATATTTATGATTATGCTTAAACAATTAAATTGTTTTATCAACTAATTAACTAACTAACTAACTTATTCTATTAAAATTCTTTAAAATTATTGTATATTATTGTAAAATTTATTTATTTATCCAAATAA
ATATTTATTTTGTTAGTATTTTGTTTATCATTAACAAATTTTAGTTTTAAAAAATAAAATAATAGCAAAAAATAATAAATAAAATTAATTTATGTTATATAAAATTTGATTAAATAATATAAAAAAAAAAAAATTTATTTTT
TATTTTATTAATTTTTCAATTTAAATAAGATATCTTAGAAGATTCGTAGAAATATTAAAATTAATTATTATTGATATTATTTAGTAATTCGTTGAAAATCAACTATTTTATATTATTAAAAGACCAAAGAGTAAAATGAT
ATTTAGATAATTTGGTGCATTTTAAAGTAAAATTTAATTCTGCTTTTCTTCAAGCTCTCGTGGTTTTTTGAGAAGCGGTATAGGCTATAGTATAGATAATATTCCTGAATATGTGGATAAAGCTATTCAAAATTAATAAGT
TATAGATAATTTACAAAATTTGACAGATAAATATTTAATTGCAATTAGTAAGAATGACAAATAGTCTATATAGTAATTAACTAAAAAGAGTTTATGTGAAAAAACTAATTAGTGGATTGATGAAGTTTCCAAAAAGGGAGA
GAGCTTTTAGCTATTAGGTACAGATAATCAAAGTGTTTCTCTTTAATGTTTCACAAGTTATAGCTATATTTATGGATTGGCAAATAACTTGGATAACAAATAGTTCGAATCTCACTCAATTTATAATTTAAATAAAAACTT
TTTGGTGAAAAGGTTGTTATTCATAGAGGGTTTTAAAGCTCACCAATTATTAACAAAGAAATCTATTTTAGTAGAAGCATTTGTATACAACACTCTTAGACTTTCAATCAAAAAAGATACTAAGCAATAATAAGGCTAATA
AAATTTATAATATGCAGGTTATAAGATTTGTTTTGTTTGTGATGGAAAATACGAATAATAAACTCCCTATATATTGCTTGATAATGATAAAAAGTAATTGTACAGCCAAGAAGATAAGCAAAAGTCTTTCGAATTAAACTG
TTGGAAAATAGCAGATATCAACGATTGGATATTTGGTGATCTTTAGTAATAAGATTTTTAAAAATGGGCTTTCCAAGCAGTAATGGATTAATAAAATGAATAAATAGCTGACTTAAAATCAACTTAAACAAAATGATTATC
AATATTTTTATAAATTGTTTGTATATAAAATAAATTACTATAATTTAATTTATTATCAATTTTAAATATTTGATTTAAATATTTTATTAATTATTTAATATAATATAATTAAAAAGAAATTATTTTATTTTATTAAAAGCA
AATATTTTATTTAGATAATTTGTTAAATAATAAAAATGATTTAATTTAATTTAAATAAATAAACTAAATTATAAATTTAAGAATATTTAATGATTTAGTTTAATTTGAAAAAAATGTAAAAATATTTTTGATATTTTAAAA
TATTAATTTTACTGATAATCAATCTTAATCGTGTTATAAATTATTGAAGAAATTAATTTGTTTATTAAAAATATGTTTCAAAAATATTTTTTTCTATGAATAAAAAGTAGAATTGATTTATTTATTGATTTTGATATTAAA
TTAATATTTATTTTTAAATGCAAACTAGTTCTAGAGCGGCCGCCACCGCGGTGGAGCTCCAGCTTTTGTTCCCTTTAGTGAGGGTTAATTTCGAGCTTGGCGTAATCATGGTCATAGCTGTTTCCTGTGTGAAATTGTTAT
CCGCTCACAATTCCACACAACATACGAGCCGGAAGCATAAAGTGTAAAGCCTGGGGTGCCTAATGAGTGAGCTAACTCACATTAATTGCGTTGCGCTCACTGCCCGCTTTCCAGTCGGGAAACCTGTCGTGCCAGCTGCAT
TAATGAATCGGCCAACGCGCGGGGAGAGGCGGTTTGCGTATTGGGCGCTCTTCCGCTTCCTCGCTCACTGACTCGCTGCGCTCGGTCGTTCGGCTGCGGCGAGCGGTATCAGCTCACTCAAAGGCGGTAATACGGTTATCC
ACAGAATCAGGGGATAACGCAGGAAAGAACATGTGAGCAAAAGGCCAGCAAAAGGCCAGGAACCGTAAAAAGGCCGCGTTGCTGGCGTTTTTCCATAGGCTCCGCCCCCCTGACGAGCATCACAAAAATCGACGCTCAAGT
CAGAGGTGGCGAAACCCGACAGGACTATAAAGATACCAGGCGTTTCCCCCTGGAAGCTCCCTCGTGCGCTCTCCTGTTCCGACCCTGCCGCTTACCGGATACCTGTCCGCCTTTCTCCCTTCGGGAAGCGTGGCGCTTTCT
CATAGCTCACGCTGTAGGTATCTCAGTTCGGTGTAGGTCGTTCGCTCCAAGCTGGGCTGTGTGCACGAACCCCCCGTTCAGCCCGACCGCTGCGCCTTATCCGGTAACTATCGTCTTGAGTCCAACCCGGTAAGACACGAC
TTATCGCCACTGGCAGCAGCCACTGGTAACAGGATTAGCAGAGCGAGGTATGTAGGCGGTGCTACAGAGTTCTTGAAGTGGTGGCCTAACTACGGCTACACTAGAAGGACAGTATTTGGTATCTGCGCTCTGCTGAAGCCA
GTTACCTTCGGAAAAAGAGTTGGTAGCTCTTGATCCGGCAAACAAACCACCGCTGGTAGCGGTGGTTTTTTTGTTTGCAAGCAGCAGATTACGCGCAGAAAAAAAGGATCTCAAGAAGATCCTTTGATCTTTTCTACGGGG
TCTGACGCTCAGTGGAACGAAAACTCACGTTAAGGGATTTTGGTCATGAGATTATCAAAAAGGATCTTCACCTAGATCCTTTTAAATTAAAAATGAAGTTTTAAATCAATCTAAAGTATATATGAGTAAACTTGGTCTGAC
AGTTACCAATGCTTAATCAGTGAGGCACCTATCTCAGCGATCTGTCTATTTCGTTCATCCATAGTTGCCTGACTCCCCGTCGTGTAGATAACTACGATACGGGAGGGCTTACCATCTGGCCCCAGTGCTGCAATGATACCG
CGAGACCCACGCTCACCGGCTCCAGATTTATCAGCAATAAACCAGCCAGCCGGAAGGGCCGAGCGCAGAAGTGGTCCTGCAACTTTATCCGCCTCCATCCAGTCTATTAATTGTTGCCGGGAAGCTAGAGTAAGTAGTTCG
CCAGTTAATAGTTTGCGCAACGTTGTTGCCATTGCTACAGGCATCGTGGTGTCACGCTCGTCGTTTGGTATGGCTTCATTCAGCTCCGGTTCCCAACGATCAAGGCGAGTTACATGATCCCCCATGTTGTGCAAAAAAGCG
GTTAGCTCCTTCGGTCCTCCGATCGTTGTCAGAAGTAAGTTGGCCGCAGTGTTATCACTCATGGTTATGGCAGCACTGCATAATTCTCTTACTGTCATGCCATCCGTAAGATGCTTTTCTGTGACTGGTGAGTACTCAACC
AAGTCATTCTGAGAATAGTGTATGCGGCGACCGAGTTGCTCTTGCCCGGCGTCAATACGGGATAATACCGCGCCACATAGCAGAACTTTAAAAGTGCTCATCATTGGAAAACGTTCTTCGGGGCGAAAACTCTCAAGGATC
TTACCGCTGTTGAGATCCAGTTCGATGTAACCCACTCGTGCACCCAACTGATCTTCAGCATCTTTTACTTTCACCAGCGTTTCTGGGTGAGCAAAAACAGGAAGGCAAAATGCCGCAAAAAAGGGAATAAGGGCGACACGG
AAATGTTGAATACTCATACTCTTCCTTTTTCAATATTATTGAAGCATTTATCAGGGTTATTGTCTCATGAGCGGATACATATTTGAATGTATTTAGAAAAATAAACAAATAGGGGTTCCGCGCACATTTCCCCGAAAAGTG
C
```

```
CaM IES                    696..1994
CaM MDS left               2001..3558
CaM MDS right              3559..5105
pBlueScriptSK(+)           5100..695
```

Supplementary References

Aronica, L., Bednenko, J., Noto, T., DeSouza, L.V., Siu, K.W., Loidl, J., Pearlman, R.E., Gorovsky, M.A., and Mochizuki, K. (2008). Study of an RNA helicase implicates small RNA-noncoding RNA interactions in programmed DNA elimination in Tetrahymena. Genes Dev *22*, 2228-2241.

Busch, C.J., Vogt, A., and Mochizuki, K. (2010). Establishment of a Cre/loxP recombination system for N-terminal epitope tagging of genes in Tetrahymena. BMC Microbiol *10*, 191.

Cassidy-Hanley, D., Bowen, J., Lee, J.H., Cole, E., VerPlank, L.A., Gaertig, J., Gorovsky, M.A., and Bruns, P.J. (1997). Germline and somatic transformation of mating Tetrahymena thermophila by particle bombardment. Genetics *146*, 135-147.

Gorovsky, M.A., Yao, M.C., Keevert, J.B., and Pleger, G.L. (1975). Isolation of micro- and macronuclei of Tetrahymena pyriformis. Methods Cell Biol *9*, 311-327.

Iwamoto, M., Mori, C., Kojidani, T., Bunai, F., Hori, T., Fukagawa, T., Hiraoka, Y., and Haraguchi, T. (2009). Two distinct repeat sequences of Nup98 nucleoporins characterize dual nuclei in the binucleated ciliate tetrahymena. Curr Biol *19*, 843-847.

Kataoka, K., Schoeberl, U.E., and Mochizuki, K. (2010). Modules for C-terminal epitope tagging of Tetrahymena genes. J Microbiol Methods *82*, 342-346.

Keller, C., Adaixo, R., Stunnenberg, R., Woolcock, K.J., Hiller, S., and Buhler, M. (2012). HP1(Swi6) mediates the recognition and destruction of heterochromatic RNA transcripts. Mol Cell *47*, 215-227.

Liu, Y., Song, X., Gorovsky, M.A., and Karrer, K.M. (2005). Elimination of foreign DNA during somatic differentiation in Tetrahymena thermophila shows position effect and is dosage dependent. Eukaryot Cell *4*, 421-431.

Loidl, J., and Scherthan, H. (2004). Organization and pairing of meiotic chromosomes in the ciliate Tetrahymena thermophila. J Cell Sci *117*, 5791-5801.

Mochizuki, K. (2008). High efficiency transformation of Tetrahymena using a codon-optimized neomycin resistance gene. Gene *425*, 79-83.

Motl, J.A., and Chalker, D.L. (2011). Zygotic expression of the double-stranded RNA binding motif protein Drb2p is required for DNA elimination in the ciliate Tetrahymena thermophila. Eukaryot Cell *10*, 1648-1659.

Noto, T., Kurth, H.M., Kataoka, K., Aronica, L., DeSouza, L.V., Siu, K.W., Pearlman, R.E., Gorovsky, M.A., and Mochizuki, K. (2010). The Tetrahymena argonaute-binding protein Giw1p directs a mature argonaute-siRNA complex to the nucleus. Cell *140*, 692-703.

Schoeberl, U.E., Kurth, H.M., Noto, T., and Mochizuki, K. (2012). Biased transcription and selective degradation of small RNAs shape the pattern of DNA elimination in Tetrahymena. Genes Dev *26*, 1729-1742.

Taus, T., Kocher, T., Pichler, P., Paschke, C., Schmidt, A., Henrich, C., and Mechtler, K. (2011). Universal and confident phosphorylation site localization using phosphoRS. J Proteome Res *10*, 5354-5362.

Vogt, A., and Mochizuki, K. (2013). A domesticated PiggyBac transposase interacts with heterochromatin and catalyzes reproducible DNA elimination in Tetrahymena. PLoS Genet *9*, e1004032.

Woehrer, S.L., Aronica, L., Suhren, J.H., Busch, C.J., Noto, T., and Mochizuki, K. (2015). A Tetrahymena Hsp90 co-chaperone promotes siRNA loading by ATP-dependent and ATP-independent mechanisms. EMBO J *34*, 559-577.
